# Supplementary material for: Hydrophobic chirality amplification in confined water cages
Source: Nat Commun. 2019 Feb 20;10:851. doi: 10.1038/s41467-019-08792-z (PMC6382825; doi:10.1038/s41467-019-08792-z)
Supplement: Supplementary file 1 — Supplementary Information [file 41467_2019_8792_MOESM1_ESM.pdf]

Supplementary Information

Hydrophobic Chirality Amplification in Confined Water Cages

Song *et al.*

Supplementary Method

General Information

Chemicals
Chemicals were purchased from various companies (including Acros, Aldrich, TCI, Alfa Aesar and Fluka) as reagent grade and used without further purification.

Solvents
Dried solvents (including CH_2Cl_2, THF, toluene, and xylenes) were purchased from various companies (including Merck, Aldrich, and Alfa Aesar) and used without further purification. Additional solvents were purchased from various commercial suppliers.

Inert Gas
Dry argon was purchased from Hanmi Gas with >99.99% purity.

Glassware
All non-aqueous reactions were performed in flame-dried glassware under Ar. Solvents were removed under reduced pressure at 30 °C using a rotary evaporator and were dried under high vacuum (10^{-1} mbar).

Thin Layer Chromatography
Thin-layer chromatography (TLC) was performed using silica gel plates (Merck, Kieselgel 60 F254 0.25 mm).

Column chromatography
Column chromatography was carried out using Merck silica gel (60 Å, 230−400 mesh, particle size 0.040−0.063 mm) with technical grade solvents. Elution was accelerated using compressed air. Reverse phase column chromatography was carried out using Aldrich C_{18}-reversed phase silica gel (90 Å, 230−400 mesh, particle size 0.040−0.063 mm) with technical grade solvents.

Nuclear Magnetic Resonance Spectroscopy
^{1}H, ^{13}C and ^{19}F nuclear magnetic resonance (NMR) spectra were recorded on a Bruker Ascend™ 500 spectrometer in a suitable deuterated solvent. The solvent employed and respective measuring frequency are indicated for each experiment. Chemical shifts are reported with tetramethylsilane (TMS) serving as an internal reference for ^{1}H and ^{13}C NMR and with two or one digits after the comma. The resonance multiplicity is described as s (singlet), d (doublet), t (triplet), q (quartet), m (multiplet), and br s (broad singlet). All spectra were recorded at 298 K unless otherwise noted, processed with the MestReNova 6.0.2 suites of programs, and the coupling constants are reported as observed. The residual deuterated solvent signal relative to tetramethylsilane was used as the internal reference in the ^{1}H NMR spectra ($CDCl_3$ δ 7.26), and are reported as follows: chemical shift δ in ppm (multiplicity, coupling constant J in Hz, number of protons). ^{13}C NMR spectra are reported in ppm from tetramethylsilane (TMS), with the solvent resonance as the internal standard ($CDCl_3$ δ 77.2). All spectra are broadband decoupled unless otherwise noted. ^{19}F NMR (470.4 MHz) spectra were recorded using a Bruker Ascend™

500 spectrometer with benzotrifluoride ($C_6H_5CF_3$) as the external standard. All spectra are broadband decoupled unless otherwise noted.
* Probably due to the self-association phenomena of cinchona-derived catalysts (**CN-1** – **CN-5** and **CD-1** – **CD-5**), marked concentration dependencies were observed for the chemical shifts in the ^{1}H and ^{13}C NMR spectra. Thus, all NMR samples for catalyst were prepared by dissolving 5 mg of catalyst in 0.5 mL of $CDCl_3$.

Mass Spectrometry
High-resolution mass spectrometry (HRMS) was recorded using a Bruker Compact mass spectrometer.

Specific Rotations
Specific rotations ($[\alpha]_D^T$) were measured with a PerkinElmer Polarimeter 343 plus at room temperature with a sodium lamp (sodium D line, λ = 589 nm).

High Performance Liquid Chromatography
High performance liquid chromatography (HPLC) was performed on Varian Pro Star Series or an YL9100 Plus HPLC System instrument equipped with an isostatic pump using a chiral column (CHIRALPAK AD-H, CHIRALCEL OD-H, CHIRALPAK IA; 250 × 4.6 mm).

Infrared Spectroscopy
Infrared (IR) spectroscopy were recorded using a Bruker Vertex 70 spectrometer with the MIRacle Micro ATR accessory.

Log *P* Calculation
Calculated Log *P* values of catalysts were calculated by using ChemBioDraw Ultra 12.0 software (Wavefunction, Inc: Irvine, CA, 2014).

Magnetic stirrer
The stirring rate (rpm) was controlled using the CORNING PC-420D (see Supplementary Figure 1).

Syringe Pump
The injection speed (flow rate) was controlled by a KD Scientific Legato 200 syringe pump.

Microfluidic chip
Four injection channels were connected to a Dolomite 3D Flow Focusing Chip (100 μm ID, Hydrophilic or Hydrophobic).

Tubing
FEP tubing is used (250, 500, 750 and 1000 μm ID).

General Procedures and Analytical Data

Catalyst preparation

Procedure A (CN-2, CD-2)

$Pd(OAc)_2$ (0.076 g, 0.34 mmol, 0.1 equiv.), PPh_3 (0.18 g, 0.68 mmol, 0.2 equiv.), Et_3N (0.94 mL, 6.8 mmol. 2 equiv.), and bromobenzene (0.72 mL, 6.8 mmol, 2 equiv.) were added at room temperature to a solution of cinchonine or cinchonidine (1 g, 3.4 mmol, 1.0 equiv.) in dried toluene (25 mL). The resulting mixture was stirred at 110 °C for 72 hours. The reaction mixture was then cooled to room temperature and filtered through a celite pad. The filtrate was concentrated *in vacuo*. The residue was purified by reversed phase column chromatography on C_{18} silica gel with a gradient elution of $MeCN$-H_2O (1:9 to 3:7), affording the desired product as a white solid.

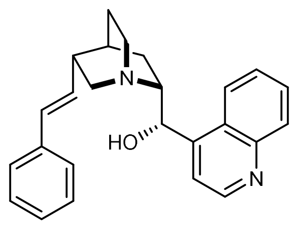

CN-2

white solid, isolated yield: 0.718 g, 57%; ^{1}H NMR (500 MHz, $CDCl_3$) δ 8.87 (d, J = 4.5 Hz, 1H), 8.14 – 8.09 (m, 1H), 7.98 – 7.94 (m, 1H), 7.68 – 7.62 (m, 2H), 7.45 – 7.40 (m, 1H), 7.38 – 7.34 (m, 2H), 7.33 – 7.27 (m, 2H), 7.23 – 7.19 (m, 1H), 6.47 – 6.35 (m, 2H), 5.75 (d, J = 4.2 Hz, 1H), 3.46 – 3.35 (m, 1H), 3.15 – 3.08 (m, 1H), 3.00 – 2.85 (m, 2H), 2.81 – 2.72 (m,1H), 2.42 – 2.34 (m, 1H), 2.17 – 2.08 (m, 1H), 1.83 (s, 1H), 1.61 – 1.45 (m, 2H), 1.24 – 1.15 (m, 1H); ^{13}C NMR (125 MHz, $CDCl_3$) δ 150.20, 148.98, 148.20, 137.59, 132.38, 130.35, 130.11, 129.02, 128.54, 127.09, 126.63, 126.07, 125.69, 122.96, 118.31, 71.77, 60.16, 50.03, 49.98, 39.62, 28.62, 26.29, 21.26; HRMS (m/z, ESI) calcd. for $[C_{25}H_{26}N_2O+H]^+$: 371.2118; found: 371.2122.

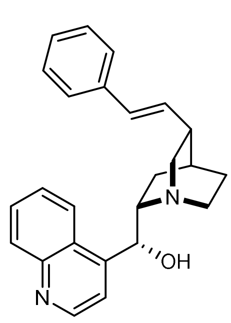

CD-2

white solid, isolated yield: 0.831 g, 66%; ^{1}H NMR (500 MHz, $CDCl_3$) δ 8.84 (d, J = 4.5 Hz, 1H), 8.13 – 8.07 (m, 1H), 8.04 – 8.00 (m, 1H), 7.70 – 7.65 (m, 1H), 7.60 – 7.57 (m, 1H), 7.48 – 7.41 (m, 1H), 7.28 – 7.21 (m, 4H), 7.18 – 7.14 (m, 1H), 6.33 (d, J = 15.8 Hz, 1H), 6.12 (dd, J = 15.8, 8.2 Hz, 1H), 5.69 (d, J = 4.1 Hz, 1H), 3.53 – 3.39 (m, 1H), 3.25 – 3.12 (m, 2H), 2.75 – 2.63 (m, 2H), 2.49 – 2.40 (m, 1H), 1.92 – 1.87 (m, 1H), 1.85 – 1.72 (m, 2H), 1.67 – 1.61 (m, 1H), 1.58 – 1.50 (m, 1H); ^{13}C NMR (125 MHz, $CDCl_3$) δ 150.19, 149.07, 148.24, 137.27, 133.50, 130.33, 130.08, 129.07, 128.48, 128.29, 127.11, 126.71, 126.62, 125.99, 125.71, 123.01, 118.21, 71.78, 60.60, 57.56, 43.15, 39.58, 28.31, 27.53, 21.87; HRMS (m/z, ESI) calcd. for $[C_{25}H_{26}N_2O+H]^+$: 371.2118; found: 371.2117.

Procedure B (CN-3, CD-3)

The **CN-2** or **CD-2** (0.090 g, 0.243 mmol) was dissolved in MeOH (30 mL). Pd/C (10%, 0.015 g) was added and the reaction mixture was stirred under H_2 at RT for 24 h. The mixture was then filtered through a celite pad, the filter bed was washed with MeOH, and the filtrate was concentrated. The residue was purified by column chromatography on silica gel with a gradient elution of EtOAc-MeOH (1:9 to 4:6), affording the desired product as a pale yellow solid.

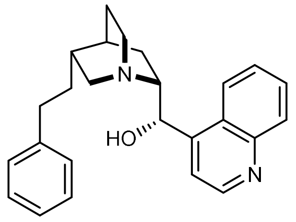

CN-3

Pale yellow solid, isolated yield: 0.049 g, 54%; ^{1}H NMR (500 MHz, $CDCl_3$) δ 8.90 (d, J = 4.4 Hz, 1H), 8.12 (d, J = 8.0 Hz, 1H), 7.99 (d, J = 8.3 Hz, 1H), 7.71 – 7.67 (m, 1H), 7.61 (d, J = 4.4 Hz, 1H), 7.51 – 7.49 (m, 1H), 7.29 (t, J = 7.5 Hz, 2H), 7.22 – 7.14 (m, 3H), 5.74 (d, J = 3.2 Hz, 1H), 3.14 – 3.11 (m, 2H), 2.98 – 2.83 (m, 2H), 2.81 – 2.70 (m, 1H), 2.62 – 2.49 (m, 2H), 2.01 – 1.93 (m, 1H), 1.75 – 1.70 (m, 3H), 1.58 – 1.45 (m, 3H), 1.29 – 1.11 (m, 2H); ^{13}C NMR (126 MHz, $CDCl_3$) δ 150.23, 148.63, 148.28, 142.38, 130.45, 129.03, 128.38, 128.34, 126.65, 125.81, 125.73, 123.02, 118.30, 71.72, 60.20, 51.15, 50.15, 34.80, 34.27, 33.67, 26.86, 26.57, 21.07; HRMS (m/z, ESI) calcd. for $[C_{25}H_{36}N_2OS+H]^+$: 373.2274; found: 373.2276.

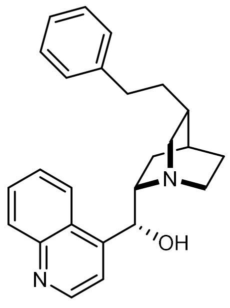

CD-3

Pale yellow solid, isolated yield: 0.065 g, 72%; ^{1}H NMR (500 MHz, $CDCl_3$) δ 8.85 (d, J = 4.5 Hz, 1H), 8.14 – 8.07 (m, 1H), 8.00 (d, J = 8.3 Hz, 1H), 7.69 – 7.63 (m, 1H), 7.57 (d, J = 4.5 Hz, 1H), 7.48 – 7.40 (m, 1H), 7.25 – 7.43 (m, 2H), 7.17 – 7.12 (m, 1H), 7.09 (d, J = 7.0 Hz, 2H), 5.65 (d, J = 4.0 Hz, 1H), 3.62 (br s, 1H), 3.49 – 3.38 (m, 1H), 3.16 – 3.10 (m, 1H), 3.06 (dd, J = 13.4, 9.3 Hz, 1H), 2.67 – 2.55 (m, 1H), 2.51 (t, J = 7.5 Hz, 2H), 2.45 – 2.38 (m, 1H), 1.82 – 1.70 (m, 3H), 1.63 – 1.47 (m, 4H), 1.46 – 1.34 (m, 1H); ^{13}C NMR (126 MHz, $CDCl_3$) δ 150.18, 149.12, 148.26, 142.16, 130.34, 129.05, 128.45, 128.32, 128.28, 127.71, 126.67, 125.77, 125.71, 123.03, 118.19, 72.02, 60.23, 58.57, 43.23, 36.73, 34.95, 33.72, 28.17, 25.88, 21.54; HRMS (m/z, ESI) calcd. for $[C_{25}H_{36}N_2OS+H]^+$: 373.2274; found: 373.2282.

Procedure C (CN-4, CN-5, CD-4, CD-5)

In a flame-dried Schlenk flask, anhydrous $CHCl_3$ (150 mL) was added to a mixture of cinchonine or cinchonidine (1 g, 3.7 mmol, 1 equiv.), azobisisobutyronitrile (AIBN) (0.167 g, 1.02 mmol, 0.3 equiv.) and the corresponding thiol (5 equiv.) under an argon atmosphere. The reaction mixture was stirred under reflux for 48 h; subsequently, the mixture was quenched with H_2O. The resulting mixture was then extracted with CH_2Cl_2. The combined organic layers were washed with brine, dried over anhydrous sodium sulfate, filtered, and concentrated in vacuo. The residue was purified by column chromatography on silica gel with a gradient elution of EtOAc-MeOH (1:9 to 4:6), affording the desired product as a white solid.

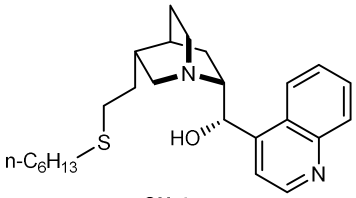

CN-4

New compound, white solid, isolated yield: 0.946 g, 62%; ^{1}H NMR (500 MHz, $CDCl_3$) δ 8.87 (d, J = 4.5 Hz, 1H), 8.11 (d, J = 7.9 Hz, 1H), 7.96 (d, J = 8.4 Hz, 1H), 7.71 – 7.64 (m, 1H), 7.60 (d, J = 4.5 Hz, 1H), 7.47 – 7.41 (m, 1H), 5.69 (d, J = 3.8 Hz, 1H), 3.70 (s, 1H), 3.12 – 3.00 (m, 2H), 2.90 – 2.80 (m, 2H), 2.78 – 2.68 (m, 1H), 2.52 – 2.40 (m, 3H), 2.00 – 1.93 (m, 1H), 1.84 (br s, 1H), 1.75 – 1.67 (m, 3H), 1.65 – 1.55 (m, 3H), 1.50 – 1.43 (m, 2H), 1.40 – 1.34 (m, 2H), 1.33 – 1.24 (m, 4H), 1.17 – 1.10 (m, 1H), 0.90 (t, J = 7.0 Hz, 3H); ^{13}C NMR (125 MHz, $CDCl_3$) δ 13C NMR (126 MHz, CDCl3) δ 150.18, 149.16, 148.22, 130.37, 129.00, 126.60, 125.68, 123.00, 118.18, 72.13, 60.03, 50.91, 50.21, 34.77, 32.39, 32.31, 31.48, 30.19, 29.65, 28.64, 27.08, 26.50, 22.58, 20.84, 14.06; HRMS (m/z, ESI) calcd. for $[C_{25}H_{36}N_2OS+H]^+$: 412.2548; found: 412.2550.

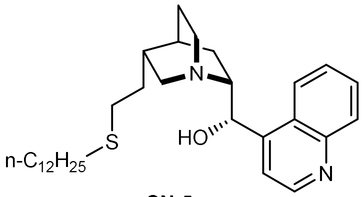

CN-5

New compound, white solid, isolated yield: 0.955 g, 52%; ^{1}H NMR (500 MHz, $CDCl_3$) δ 8.87 (d, J = 4.5 Hz, 1H), 8.16 – 8.09 (m, 1H), 7.98 (d, J = 8.2 Hz, 1H), 7.73 – 7.64 (m, 1H), 7.60 (d, J = 4.5 Hz, 1H), 7.51 – 7.44 (m, 1H), 5.69 (d, J = 4.2 Hz, 1H), 3.52 (br s, 1H), 3.13 – 3.02 (m, 2H), 2.94 – 2.85 (m, 2H), 2.75 – 2.68 (m, 1H), 2.49 – 2.44 (m, 3H), 2.01 – 1.78 (m, 2H), 1.75 – 1.65 (m, 3H), 1.62 – 1.55 (m, 3H), 1.54 – 1.45 (m, 2H), 1.44 – 1.37 (m, 2H), 1.35 – 1.20 (m, 15H), 0.88 (t, J = 7.0 Hz, 3H); ^{13}C NMR (125 MHz, $CDCl_3$) δ 150.19, 149.07, 148.23, 130.39, 129.02, 126.62, 125.68, 123.00, 118.19, 72.09, 60.04, 50.91, 50.20, 34.73, 32.37, 32.32, 31.92, 30.18, 29.69, 29.68, 29.65, 29.64, 29.57, 29.36, 29.30, 28.99, 27.05, 26.50, 22.69, 20.85, 14.12; HRMS (m/z, ESI) calcd. for $[C_{31}H_{48}N_2OS+H]^+$: 496.3487; found: 496.3489.

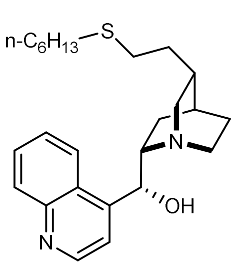

CD-4

New compound, white solid, isolated yield: 1.038 g, 68%; ^{1}H NMR (500 MHz, $CDCl_3$) δ 8.85 (d, J = 4.5 Hz, 1H), 8.12 – 7.09 (m, 1H), 8.00 (d, J = 8.2 Hz, 1H), 7.70 – 7.65 (m, 1H), 7.56 (d, J = 4.5 Hz, 1H), 7.48 – 7.43 (m, 1H), 5.63 (d, J = 4.2 Hz, 1H), 3.54 (br s, 1H), 3.50 – 3.35 (m, 1H), 3.15 – 3.03 (m, 2H), 2.68 – 2.59 (m, 1H), 2.48 – 2.38 (m, 4H), 1.81 – 1.70 (m, 3H), 1.66 (s, 1H), 1.59 – 1.38 (m, 7H), 1.35 – 1.19 (m, 6H), 0.87 (t, J = 7.0 Hz, 3H); ^{13}C NMR (125 MHz, $CDCl_3$) δ 150.15, 149.12, 148.24, 130.34, 129.06, 126.69, 125.66, 122.98, 118.17, 72.03, 60.18, 58.30, 43.20, 34.72, 34.66, 32.27, 31.42, 30.10, 29.58, 28.57, 28.10, 25.74, 22.53, 21.46, 14.02; HRMS

(m/z, ESI) calcd. for $[C_{25}H_{36}N_2OS+H]^+$: 412.2548; found: 412.2550.

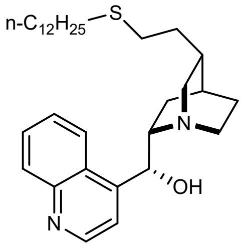

CD-5

New compound, white solid, isolated yield: 1.010 g, 55%; ^{1}H NMR (500 MHz, $CDCl_3$) δ 8.88 – 8.83 (m, 1H), 8.15 – 8.08 (m, 1H), 8.00 – 7.95 (m, 1H), 7.70 – 7.64 (m, 1H), 7.60 – 7.55 (m, 1H), 7.46 – 7.40 (m, 1H), 5.77 – 5.64 (m, 1H), 3.51 – 3.42 (m, 1H), 3.17 – 3.02 (m, 2H), 2.75 – 2.60 (m, 1H), 2.49 – 2.35 (m, 5H), 1.80 – 1.68 (m, 4H), 1.60– 1.40 (m, 7H), 1.35 – 1.20 (m, 18H), 0.88 (t, J = 7.0 Hz, 3H); ^{13}C NMR (125 MHz, $CDCl_3$) δ 150.17, 148.96, 148.26, 130.37, 129.06, 129.01, 126.71, 126.63, 125.63, 122.94, 118.20, 114.80, 71.78, 61.13, 60.16, 58.20, 56.66, 43.95, 43.20, 34.64, 34.61, 33.31, 32.28, 31.91, 30.08, 29.65, 29.63, 29.59, 29.52, 29.34, 29.23, 28.91, 27.99, 27.90, 27.72, 25.72, 22.69, 21.38, 14.12, 12.35; HRMS (m/z, ESI) calcd. for $[C_{31}H_{48}N_2OS+H]^+$: 496.3487; found: 496.3487.

Substrate preparation

N-Boc amidosulfones and *N*-Boc imines were prepared using the slightly modified previously documented procedure[1]

General procedure for the synthesis of *N*-Boc amidosulfones

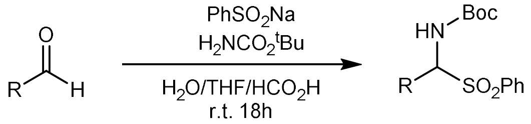

Water (100 mL), sodium benzenesulfinate (18.21 g, 110.9 mmol), and the appropriate aldehyde (113.1 mmol) followed by formic acid (24.3 mL, 643 mmol) were added to a solution of *tert*-butyl carbamate (13.00 g, 110.9 mmol) in THF (40 mL). The reaction mixture was stirred for 18 h at room temperature. The resultant white suspension was then filtered, washed with water, and then triturated with a mixture of hexane/dichloromethane (150/15 mL) overnight. The solid was then dried under reduced pressure.

General procedure for the synthesis of *N*-Boc imines

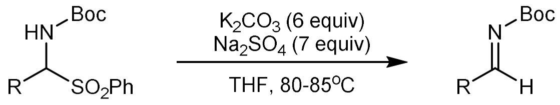

Under an argon atmosphere, one equivalent of α-amidosulfone (2 mmol) was refluxed in THF overnight in the presence of 1.60 g (12 mmol) of K_2CO_3 and ~2 g Na_2SO_4 (drying agent). Under argon atmosphere, the resulting mixture was filtered through a celite pad and the volatiles were evaporated. The resulting imine was transferred carefully under argon to a dried flask. Cautious handling of the imine is required to prevent decomposition and/or hydrolysis.

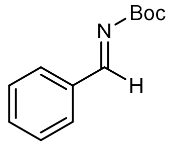

1a

^{1}H NMR (500 MHz, $CDCl_3$): *δ* 8.87 (s, 1H), 7.93–7.90 (m, 2H), 7.57–7.54 (m, 1H), 7.49–7.44 (m, 2H), 1.59 (s, 9H); ^{13}C NMR (125 MHz, $CDCl_3$): *δ* 169.71, 162.64, 134.06, 133.53, 130.21, 128.87, 82.28, 27.93.

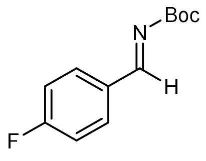

1b

^{1}H NMR (500 MHz, $CDCl_3$): δ 8.86 (s, 1H), 7.94 – 7.91 (m, 2H), 7.18 – 7.13 (m, 2H), 1.59 (s, 9H); ^{13}C NMR (125 MHz, $CDCl_3$): δ 168.35, 167.05, 165.01, 162.42, 132.52 (d, *J* = 9.3 Hz), 130.43 (d, *J* = 3.0 Hz), 116.24 (d, *J* = 22.1 Hz), 82.38, 27.90.

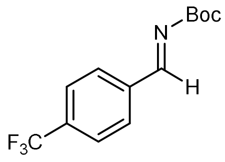

1c

^{1}H NMR (500 MHz, $CDCl_3$): δ 8.86 (s, 1H), 8.02 (d, J = 8.1 Hz, 2H), 7.73 (d, J = 8.2 Hz, 2H), 1.60 (s, 9H); ^{13}C NMR (125 MHz, $CDCl_3$): δ 167.41, 162.08, 137.16 (d, J = 0.7 Hz), 134.53 (q, J = 32.7 Hz), 130.16, 125.80 (q, J = 3.7 Hz), 123.56 (q, J = 272.7 Hz), 82.81, 27.83.

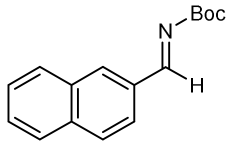

1d

^{1}H NMR (500 MHz, $CDCl_3$): δ 8.99 (s, 1H), 8.17 (s, 1H), 8.05 (dd, J = 8.6, 1.6 Hz, 1H), 7.86 – 7.73 (m, 4H), 7.55 – 7.45 (m, 2H), 1.61 (s, 9H).; ^{13}C NMR (125 MHz, $CDCl_3$): δ 169.85, 162.69, 135.97, 134.18, 132.73, 131.76, 129.18, 128.82, 128.60, 127.97, 126.88, 123.89, 82.20, 27.98.

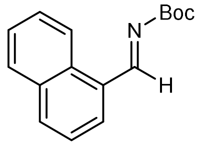

1e

^{1}H NMR (500 MHz, $CDCl_3$): δ 9.54 (s, 1H), 8.93 (d, J = 8.6 Hz, 1H), 8.19 (d, J = 7.2 Hz, 1H), 8.05 (d, J = 8.2 Hz, 1H), 7.92 (d, J = 8.1 Hz, 1H), 7.69 –7.65 (m, 1H), 7.58 – 7.53 (m, 2H), 1.63 (s, 9H).; ^{13}C NMR (125 MHz, $CDCl_3$): δ 167.91, 161.81, 133.23, 132.68, 130.94, 130.82, 128.20, 127.80, 127.05, 125.44, 124.03, 122.87, 81.14, 76.31, 76.05, 75.80, 26.93.

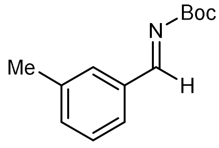

1f

^{1}H NMR (500 MHz, $CDCl_3$): δ 8.87 (s, 1H), 7.78 (s, 1H), 7.68 (d, J = 6.6 Hz, 1H), 7.40–7.33 (m, 2H), 2.40 (s, 3H), 1.59 (s, 9H); ^{13}C NMR (125 MHz, $CDCl_3$): δ 170.12, 162.67, 138.72, 134.45, 134.01, 130.18, 128.74, 128.03, 82.18, 77.35, 77.10, 76.84, 27.93, 21.16.

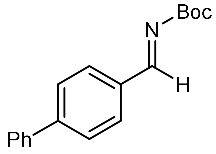

1g

^{1}H NMR (500 MHz, $CDCl_3$): δ 8.93 (s, 1H), 8.00–7.99 (m, 2H), 7.71–7.69 (m, 2H), 7.66–7.62 (m, 2H), 7.49–7.44 (m, 2H), 7.43–7.37 (m, 1H). 1.60 (s, 9H); ^{13}C NMR (125 MHz, $CDCl_3$): δ 169.50, 162.65, 146.23, 139.77, 132.93, 130.76, 128.94, 128.29, 127.48, 127.22, 82.22, 27.93.

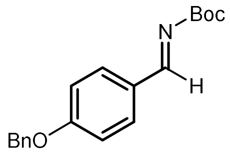

1h

^{1}H NMR (500 MHz, $CDCl_3$): δ 8.86 (s, 1H), 7.87 (d, *J* = 8.8 Hz, 2H), 7.46–7.35 (m, 5H), 7.01 (d, *J* = 8.8 Hz, 2H), 5.09 (s, 2H), 1.58 (s, 9H); ^{13}C NMR (125 MHz, $CDCl_3$): δ 190.76, 169.69, 163.31, 162.87, 136.08, 132.54, 132.01, 128.72, 128.30, 127.54, 127.45, 127.16, 115.21, 81.86, 70.21, 27.98.

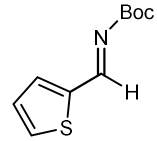

1i

^{1}H NMR (500 MHz, $CDCl_3$): δ 9.07 (s, 1H), 7.68–7.66 (m, 2H), 7.18–7.16 (m, 1H), 1.57 (s, 9H); ^{13}C NMR (125 MHz, $CDCl_3$): δ 163.41, 162.11, 140.21, 137.06, 134.17, 128.38, 82.08, 27.88.

General procedure for the asymmetric Mannich reaction under on-water conditions

Acetylacetone **2** or dimethylmalonate **4** (0.6 mmol) was added to the mixture of *N*-Boc imine **1** (0.3 mmol), catalyst (0.003 mmol, 1 mol%), toluene or *o*-xylene (4.5 mmol), and water or brine (2.0 mL). The reaction mixture was stirred vigorously with a magnetic bar at 1150 rpm and the temperature was set at 20 °C. After completion of the reaction (0.5 h - 1 h for **2** and 24 h - 48 h for **4**, respectively), the reaction mixture was extracted with ethyl acetate (3 x 5 mL). The combined organic layers were washed with brine, dried over anhydrous sodium sulfate, filtered, and concentrated in vacuo. The residue was purified by column chromatography on silica gel eluting with a hexanes/ethyl acetate mixture, affording the corresponding Mannich product **3** or **5**.

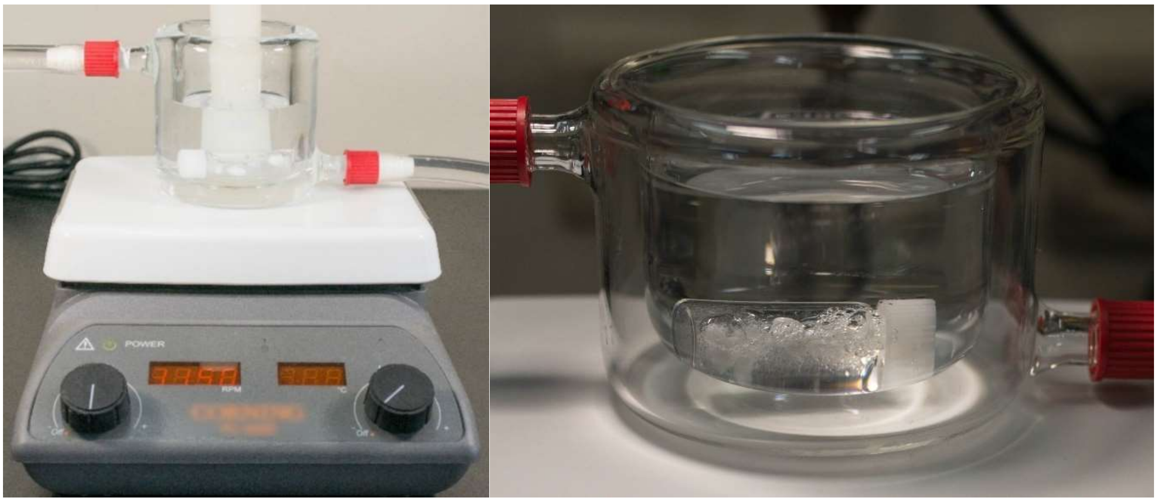

Supplementary Figure 1. Control of the stirring rate (rpm) using the CORNING PC-420D.

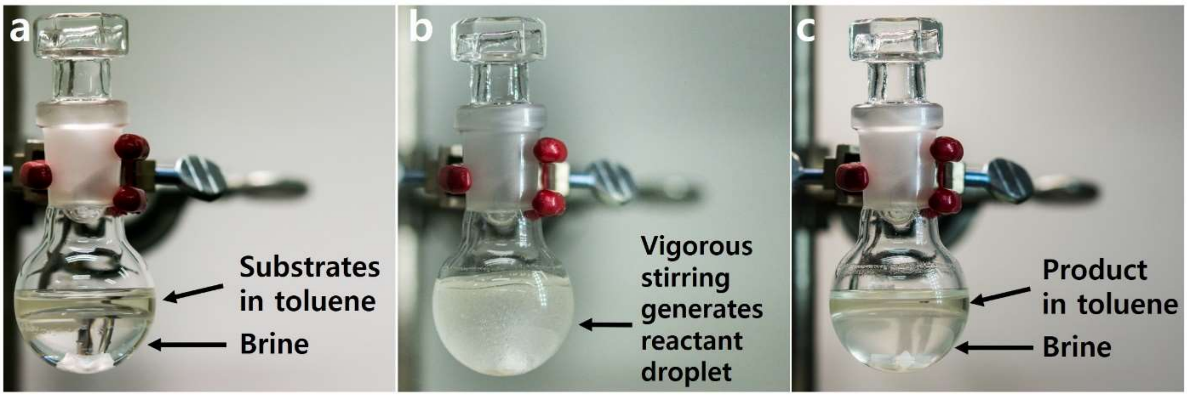

Supplementary Figure 2. Photos of asymmetric Mannich reaction under on-water conditions. **a**. before reaction; **b**. during reaction; **c**. after reaction.

Comment**:** As shown in the above pictures, the organic phase is dispersed, not solubilized, demonstrating an on-water system.

Compound 3a: Isolated yield: 85.2 mg (93%); white solid

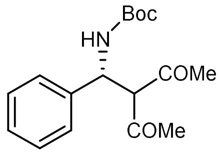

3a

^{1}H NMR (500 MHz, $CDCl_3$): δ 7.34 – 7.23 (m, 5H), 5.79 (br s, 1H), 5.50 (br s, 1H), 4.22 (br s, 1H), 2.19 (s, 3H), 2.11 (s, 3H), 1.40 (s, 9H); ^{13}C NMR (125 MHz, $CDCl_3$): δ 204.50, 202.50, 155.16, 139.85, 128.79, 127.73, 126.36, 80.10, 71.72, 53.77, 30.47, 30.08, 28.25.

HPLC analysis: Chiralpak AD-H, Hex/IPA = 95/5, 1.0 mL/min, 210 nm; t_R = 20.1 min (minor, *S*), 22.7 min (major, *R*)

The analytical data was identical to the reported value[2].

Compound 3b: Isolated yield: 90.2 mg (93%); white solid; Mp: 101 °C

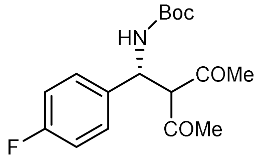

3b

^{1}H NMR (500 MHz, $CDCl_3$): δ 7.30–7.27 (m, 2H), 7.03–6.99 (m, 2H), 6.15 (br s, 1H), 5.46 (br s, 1H), 3.89 (br s, 1H), 3.74 (s, 3H), 3.65 (s, 3H), 1.42 (s, 9H); ^{13}C NMR (125 MHz, $CDCl_3$): δ 168.40, 167.48, 162.23 (d, J = 244.7 Hz), 155.13, 135.33, 1.09 (d, J = 8.1 Hz), 115.59 (d, J = 21.5 Hz), 80.03, 56.77, 53.01, 52.71, 28.35; IR (neat): ν 3376.32, 2968.76, 1759.90, 1736.14, 1684.91, 1515.42, 1284.26, 1167.91 cm^{-1}.

HPLC analysis: Chiralpak AD-H, Hex/IPA = 90/10, 1.0mL/min, 210 nm; t_R = 13.9 min (major, *R*), 17.4 min (minor, *S*)

The analytical data was identical to the reported value[2].

Compound 3c: New compound, isolated yield: 108.6 mg (97%); white solid; Mp: 161 °C

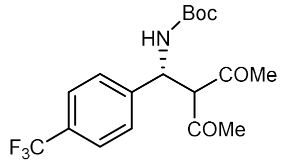

3c

^{1}H NMR (500 MHz, $CDCl_3$): δ 7.59 (d, J = 8.2 Hz, 2H), 7.42 (d, J = 8.2 Hz, 2H), 5.95 (s, 1H), 5.55 (s, 1H), 4.24 (d, J = 4.2 Hz, 1H), 2.25 (s, 3H), 2.11 (s, 3H), 1.40 (s, 9H); ^{13}C NMR (125 MHz, $CDCl_3$): δ 204.30, 202.07, 155.19, 143.99, 129.90 (q, J = 32.5 Hz), 126.86, 125.72 (q, J = 3.7 Hz), 123.91 (q, J = 270.4 Hz), 80.44, 71.01, 53.25, 30.62, 29.90, 28.20.; IR (neat): ν 3390.75, 2980.60, 1684.82, 1519.02, 1332.31, 1161.95, 1124.19 cm^{-1}; HRMS (m/z, ESI) calcd. for $[C_{18}H_{22}F_3NO_4+Na]^+$: 396.1393; found: 396.1394.

HPLC analysis: Chiralpak AD-H, Hex/IPA = 90/10, 1.0mL/min, 210 nm; t_R = 8.0 min (major, *R*), 10.3 min (minor, *S*)

Compound 5a: Isolated yield: 92.1 mg (91%); white solid

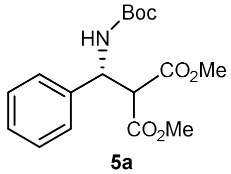

5a

^{1}H NMR (500 MHz, $CDCl_3$): δ 7.33–7.24 (m, 5H), 6.17 (br s, 1H), 5.50 (br s, 1H), 3.94 (br s, 1H), 3.73 (s, 3H), 3.62 (s, 3H), 1.42 (s, 9H); ^{13}C NMR (125 MHz, $CDCl_3$): δ 168.40, 167.58, 155.12, 139.43, 128.62, 127.67, 126.23, 79.74, 56.69, 53.41, 52.86, 52.53, 28.28.

HPLC analysis: Chiralpak IA, Hex/IPA = 90/10, 1.0 mL/min, 210 nm; t_R = 14.4 min (minor, *S*), 18.4 min (major, *R*)

The analytical data was identical to the reported value[3].

Compound 5b: Isolated yield: 99.3 mg (93%); white solid; Mp: 101 °C

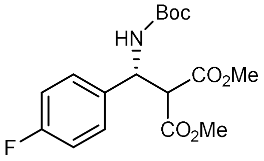

5b

^{1}H NMR (500 MHz, $CDCl_3$): δ 7.30–7.27 (m, 2H), 7.03–6.99 (m, 2H), 6.15 (br s, 1H), 5.46 (br s, 1H), 3.89 (br s, 1H), 3.74 (s, 3H), 3.65 (s, 3H), 1.42 (s, 9H); ^{13}C NMR (125 MHz, $CDCl_3$): δ 168.40, 167.48, 162.23 (d, J = 244.7 Hz), 155.13, 135.33, 128.09 (d, J = 8.1 Hz), 115.59 (d, J = 21.5 Hz), 80.03, 56.77, 53.01, 52.71, 28.35; IR (neat): ν 3376.32, 2968.76, 1759.90, 1736.14, 1684.91, 1515.42, 1284.26, 1167.91 cm^{-1}.
HPLC analysis: Chiralpak AD-H, Hex/IPA = 90/10, 1.0 mL/min, 210 nm; t_R = 19.3 min (major, *R*), 23.6 min (minor, *S*)

The analytical data was identical to the reported value[5].

Compound 5c: Isolated yield: 102.2 mg (84%); white solid

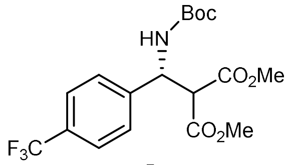

5c

^{1}H NMR (500 MHz, $CDCl_3$): δ 7.59 (d, J = 8.2 Hz, 2H), 7.44 (d, J = 8.2 Hz, 2H), 6.22 (br s, 1H), 5.54 (br s, 1H), 3.94 (s, 1H), 3.77 (s, 3H), 3.65 (s, 3H), 1.43 (s, 9H).; ^{13}C NMR (125 MHz, $CDCl_3$): δ 168.16, 167.22, 155.06, 143.54, 129.97 (q, J = 32.8 Hz), 126.73, 125.63 (q, J = 3.6 Hz), 123.97 (q, J = 272.0 Hz), 80.16, 56.26, 53.03, 52.69, 28.24.
HPLC analysis: Chiralpak AD-H, Hex/IPA = 90/10, 1.0 mL/min, 210 nm; t_R = 12.2 min (major, *R*), 20.5 min (minor, *S*)

The analytical data was identical to the reported value[5].

Compound 5d: Isolated yield: 109.3 mg (94%); white solid

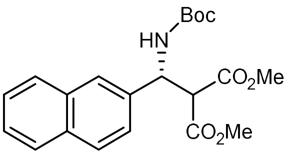

5d

^{1}H NMR (500 MHz, $CDCl_3$): δ 7.80–7.76 (m, 4H), 7.44–7.40 (m, 3H), 6.35 (br d, J = 8.6 Hz, 1H), 5.68 (br s, 1H), 4.06 (br s, 1H), 3.72 (s, 3H), 3.57 (s, 3H), 1.43 (s, 9H); ^{13}C NMR (125 MHz, $CDCl_3$): δ 168.46, 167.59, 155.21, 136.90, 133.25, 132.82, 128.51, 128.07, 127.63, 126.32, 126.12, 125.26, 124.30, 79.83, 56.66, 53.68, 52.91, 52.58, 28.33.
HPLC analysis: Chiralpak AD-H, Hex/IPA = 90/10, 1.0 mL/min, 210 nm; t_R = 24.5 min (minor, *S*), 29.3 min (major, *R*)

The analytical data was identical to the reported value[3].

Compound 5e: Isolated yield: 106.9 mg (92%); white solid

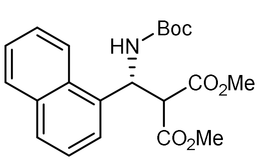

5e

^{1}H NMR (500 MHz, $CDCl_3$): δ 8.13 (d, J = 8.5 Hz, 1H), 7.85 (d, J = 8.1 Hz, 1H), 7.76 (d, J = 8.1 Hz, 1H), 7.58 (t, J = 7.1 Hz, 1H), 7.49 (m, 2H), 7.41 (t, J = 7.9 Hz, 1H), 6.60 (d, J = 9.2 Hz 1H), 6.30 (d, J = 5.9 Hz, 1H), 4.10 (br s, 1H), 3.80 (s, 3H), 3.54 (s, 3H), 1.43 (s, 9H); ^{13}C NMR (125 MHz, $CDCl_3$): δ 168.62, 167.81, 155.14, 134.82, 133.89, 130.05, 129.20, 128.59, 126.91, 125.83, 125.20, 123.67, 122.18, 79.84, 55.39, 53.05, 52.42, 50.18, 28.31.
HPLC analysis: Chiralpak AD-H, Hex/IPA = 90/10, 1.0 mL/min, 210 nm; t_R = 12.3 min (major, *R*), 13.1 min (minor, *S*)

The analytical data was identical to the reported value[4].

Compound 5f: Isolated yield: 94.9 mg (90%); white solid

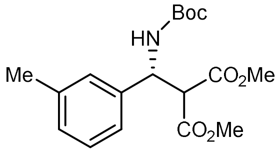

5f

^{1}H NMR (500 MHz, $CDCl_3$): δ 7.20 (t, J = 7.5 Hz, 1H), 7.13 – 7.02 (m, 3H), 6.13 (br s, 1H), 5.46 (br s, 1H), 3.92 (s, 1H), 3.75 (s, 3H), 3.64 (s, 3H), 2.33 (s, 3H), 1.42 (s, 9H).; ^{13}C NMR (125 MHz, $CDCl_3$): δ 168.45, 167.65, 155.13, 139.35, 138.27, 128.50, 128.43, 127.00, 123.14, 79.72, 56.72, 53.39, 52.87, 52.52, 28.30, 21.50.

HPLC analysis: Chiralpak AD-H, Hex/IPA = 90/10, 1.0 mL/min, 210 nm; t_R = 14.0 min (minor, *S*), 20.9 min (major, *R*)

The analytical data was identical to the reported value[3].

Compound 5g: New compound, isolated yield: 112.8 mg (91%); white solid

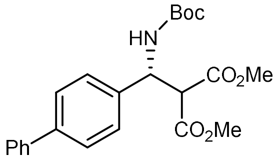

5g

^{1}H NMR (500 MHz, $CDCl_3$): δ 7.60 – 7.50 (m, 4H), 7.46 – 7.40 (m, 2H), 7.39 – 7.29 (m, 3H), 6.19 (br s, 1H), 5.54 (br s, 1H), 3.97 (s, 1H), 3.76 (s, 3H), 3.66 (s, 3H), 1.43 (s, 9H); ^{13}C NMR (125 MHz, $CDCl_3$): δ 168.44, 167.57, 155.15, 140.54, 138.45, 128.79, 127.40, 127.35, 127.05, 126.68, 79.87, 56.64, 53.24, 52.92, 52.60, 28.31; IR (neat): ν 3386.30, 2977.32, 2958.42, 2896.11, 1732.54, 1683.08, 1519.24, 1242.60, 1164.42 cm^{-1}; HRMS (m/z, ESI) calcd. for $[C_{23}H_{27}NO_6+Na]^+$: 436.1731; found: 436.1731.

HPLC analysis: Chiralpak AD-H, Hex/IPA = 90/10, 1.0 mL/min, 210 nm; t_R = 19.6 min (minor, *S*), 37.2 min (major, *R*)

Compound 5h: New compound, isolated yield: 80.2 mg (60%); white solid

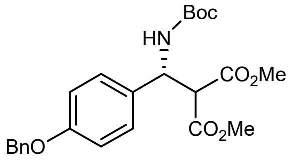

5h

^{1}H NMR (500 MHz, $CDCl_3$): δ 7.39 – 7.36 (m, 4H), 7.33 – 7.30 (m, 1H), 7.21 – 7.18 (m, 2H), 6.94 – 6.89 (m, 2H), 6.08 (s, 1H), 5.43 (s, 1H), 5.03 (s, 2H), 3.89 (s, 1H), 3.73 (s, 3H), 3.65 (s, 3H), 1.41 (s, 9H); ^{13}C NMR (125 MHz, $CDCl_3$): δ 168.46, 167.60, 158.21, 155.07, 136.87, 131.80, 128.61, 128.02, 127.51, 127.45, 114.90, 79.75, 70.02, 56.84, 52.86, 52.57, 28.30;

HRMS (m/z, ESI) calcd. for $[C_{24}H_{29}NO_7+Na]^+$: 466.1836; found: 466.1833.

HPLC analysis: Chiralpak AD-H, Hex/IPA = 80/20, 1.0 mL/min, 210 nm; t_R = 21.6 min (major, *R*), 31.0 min (minor, *S*)

Compound 5i: Isolated yield: 88.2 mg (86%); white solid

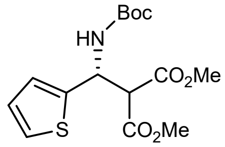

5i

^{1}H NMR (500 MHz, $CDCl_3$): δ 7.20–7.19 (m, 1H), 6.93–6.92 (m, 2H), 6.13 (br d, J = 8.1 Hz, 1H), 5.71 (br d, J = 4.9 Hz, 1H), 4.01 (br d, J = 3.5 Hz, 1H), 3.75 (s, 3H), 3.71 (s, 3H), 1.44 (s, 9H); ^{13}C NMR (125 MHz, $CDCl_3$): δ 168.26, 167.18, 154.93, 143.52, 126.94, 124.79, 124.42, 79.99, 58.60, 52.95, 52.69, 49.95, 28.28.

HPLC analysis: Chiralpak AD-H, Hex/IPA = 90/10, 1.0 mL/min, 210 nm; t_R = 15.3 min (minor, *S*), 21.6 min (major, *R*)

The analytical data was identical to the reported value[3].

General procedure for the asymmetric Mannich reaction under biphasic microfluidic conditions

Microfluidic device: The oil and aqueous phase were loaded into Hamilton glass syringes (Hamilton 1001TLL). FEP tubing (250 µm ID) was fitted over needles and used to transport the liquid to the droplet formation cross. Dolomite 3D Flow Focusing Chip (100 µm ID, hydrophilic or hydrophobic) was used for generating the plug flow of the organic phase in the aqueous phase. Additional FEP tubing (250, 500, 750 or 1000 µm ID, 15 m length) is connected to the exit line of the chip for the plug-flowing reaction mixture (See, Supplementary Figure 3).

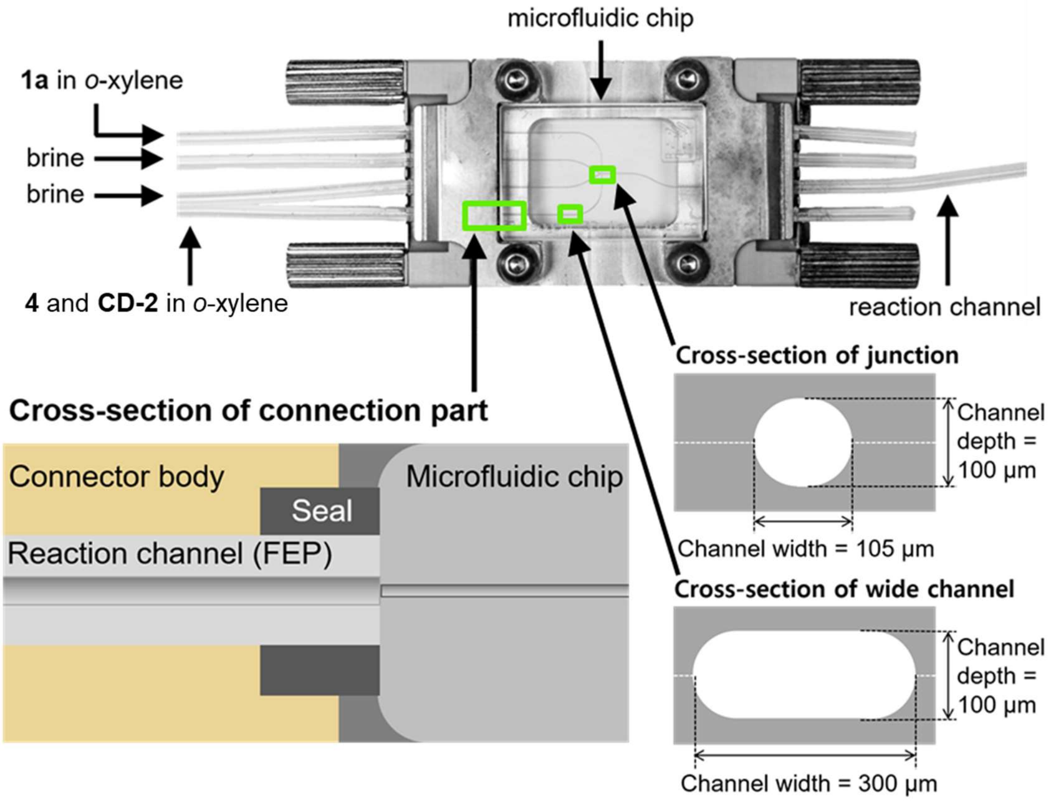

Supplementary Figure 3. Microfluidic device used in this study

Reactions in droplets: The organic solutions (**solution 1**: dimethyl malonate **4** (69 µL, 0.6 mmol) and catalyst **CD-2** or **CN-2** (1.1 mg, 0.003 mmol) in *o*-xylene (0.88 mL), the ultrasonification was used to assist the dissolution of catalyst in *o*-xylene; **solution 2**: phenyl *N*-Boc imine **1a** (61.6mg, 0.3 mmol) in *o*-xylene (0.88 mL)) and the aqueous solution (brine, 5.13 M) were loaded into separate syringes, then injected onto the microfluidic chip at different flow rates. The brine and organic solutions were brought together on the chip, then coflowed and compartmentalized into droplets by flow focusing of the aqueous phase with an organic phase. After the tube was filled with the reaction mixture, the flows of brine and organic solutions were stopped, and the outlet of the tube was then sealed tightly with vacuum grease. The other end of the tube was also sealed with vacuum grease. The biphasic plugs were kept inside the FEP tubing at 20 ^{o}C without any shaking. After 24-48 h, the reaction mixture was collected from the outlet

of the tubing by flushing with argon. The organic phase was then purified by chromatography to determine the conversions and *ee* values. The conversions of the reaction mixture were determined using ^{1}H NMR spectra by comparing the product peak integration with the reactant peak integration. The % *ee* of the product **5a** was determined by chiral HPLC.

A series of droplets of different sizes was formed in the FEP tubing by adjusting the flow rate ratio between the organic and aqueous phases. The droplet volumes were determined by dividing the organic flow rate (Q_o) by the droplet production frequency, measured using a high speed camera (Supplementary Table 3).
Images of droplets in the reaction channel were captured using a Nikon D 800 digital SLR camera equipped with an AF-S 60mm f/2.8 Macro lens.

Typical high pressure experimental procedure

The reaction was carried out according to the Hayashi procedure[6]. *N*-Boc-benzaldimine **1a** (50 mg, 0.24 mmol), **CN-2** (5.6 mg, 0.0024 mmol), dimethyl malonate (56 μL, 0.48 mmol), and anhydrous CH_2Cl_2 (4 mL) were added to a 4 mL Teflon tube at -20 °C and the tube was capped with exclusion of air. This tube was placed in an autoclave (Supplementary Figure 4), the inner capacity of which is 100 mL. The autoclave was completely filled with pre-cooled water (ca. 4 °C), sealed tightly, and left in a domestic electric refrigerator at -20 °C. It has been determined by Hayashi that under the above experimental conditions the internal pressure can reach about 200 MPa after 12 h. After 48 h, the autoclave was removed from the refrigerator, and the Teflon tube was removed from the autoclave. After removing the volatile organic solvent under reduced pressure, the residue was purified by column chromatography on silica gel (hexane:EtOAc =4:1) to obtain the Mannich adduct in a 94% yield (76.1 mg) with 89% *ee*.

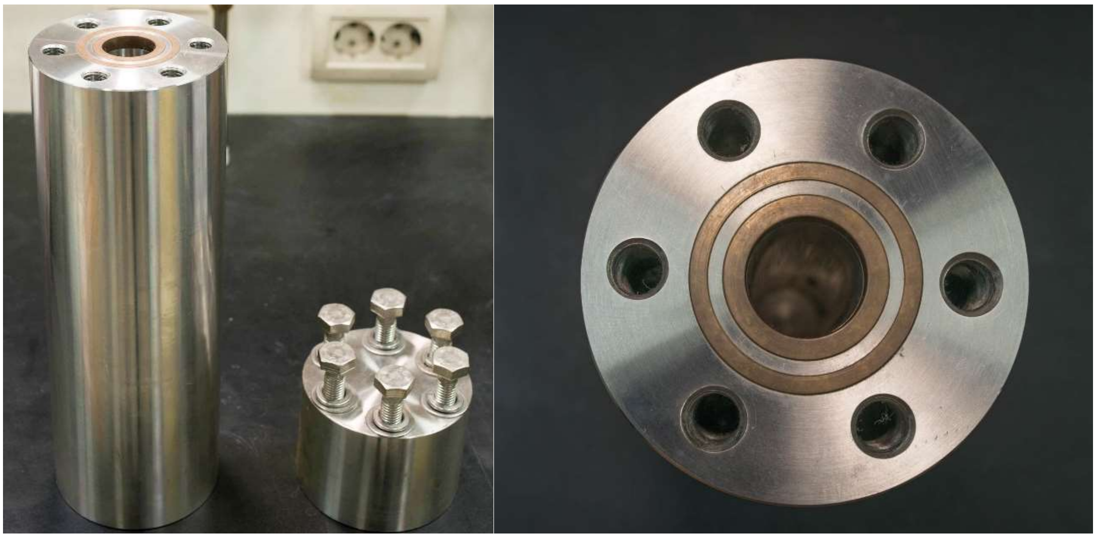

Supplementary Figure 4. An autoclave for water-freezing high pressure experiments

Supplementary Tables

Supplementary Table 1. Asymmetric Mannich reaction of imine (**1a**) with 2,4-pentanedione (**2**) or dimethyl malonate (**4**) catalyzed by **CN-1** in various reaction media[a]

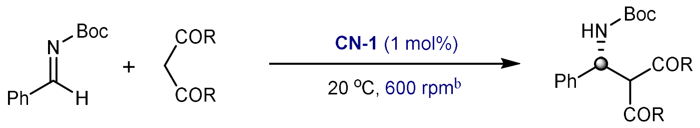

1a **2** (R = Me) **4** (R = OMe) (*R*)-**3a** (R = Me) (*R*)-**5a** (R = OMe)

Entry	Catalyst	R	Time (h)	Medium	Additive (eq.)	Conv. [c]/Yield (%)	*ee* (%)[d]
1	**CN-1**	Me	1	THF	-	>99[c]	1
2	**CN-1**	Me	1	CH_3CN	-	>99[c]	3
3	**CN-1**	Me	1	EtOH	-	>99[c]	1
4	**CN-1**	Me	1	formamide	-	>99[c]	4
5	**CN-1**	Me	1	CH_2Cl_2	-	81	22
6	**CN-1**	Me	1	*o*-xylene	-	91	43
7*	**CN-1**	Me	1	brine	-	84	55
8*	No cat	Me	0.5	brine	-	85	0
9	**CN-1**	Me	1	water	toluene (15)	85	84
10	**CN-1**	Me	1	brine	toluene (15)	89	84
11	**CN-1**	Me	1	brine	*o*-xylene (15)	90	84
12	**CN-1**	Me	1	$H_2O/LiClO_4$[d]	*o*-xylene (15)	63	15
13	**CN-1**	OMe	24	formamide	-	86	1
14	**CN-1**	OMe	24	CH_2Cl_2	-	84	32
15	**CN-1**	OMe	24	*o*-xylene	-	88	39
16*	**CN-1**	OMe	24	brine	-	90	42
17*	No cat	OMe	0.5	brine	-	87	0
18**	**CN-1**	OMe	24	water	toluene (15)	90[c]	87
19	**CN-1**	OMe	24	brine	toluene (15)	>99[c]/88	87
20	**CN-1**	OMe	24	brine	*o*-xylene (15)	>99[c]/91	85
21***	**CN-1**	OMe	24	$D_2O/NaCl$[e]	toluene (15)	>97[c]/87	87
22	**CN-1**	OMe	24	$H_2O/LiClO_4$[e]	toluene (15)	74	10

[a]General reaction conditions: **1a** (0.3 mmol), **2** or **4** (0.6 mmol), and catalyst **CN-1** (1 mol%) on 2.0 mL of media at r.t. [b]For stirring speed, see Supplementary Information for experimental details. [c]The conversion was determined by ^{1}H NMR integration. [d]Enantiomeric excess (% *ee*) was determined by high-performance liquid chromatography (HPLC). [d]Saturated solutions.

*__Comment:__ The organic phase is dispersed, not solubilized, therefore demonstrating an on-water system.
**__Comments:__ Using pure water instead of brine, only a slight difference in rates was observed. The reaction using brine proceeded slightly faster than using pure water.
***__Comments:__ We did not observe any kinetic solvent isotope effects, which strongly indicates that, under our on-water conditions, the interfacial hydrogen bonding, or a proton transfer process between the aqueous phase and organic reactants does not involved in the transition state. If our on-water catalysis results from the general acid mechanism, then the reaction ought to be slower on less acidic D_2O (entry 19 vs. entry 21).

Supplementary Table 2. Effect of catalyst hydrophobicity on enantioselectivity[a]

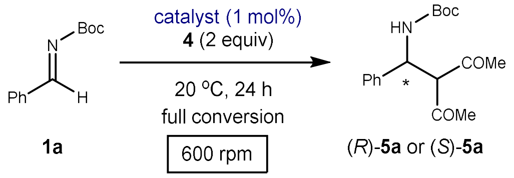

Entry	Catalyst	Medium	Additive (eq.)	*ee* (%)[b]
1	**CN-1**	brine	toluene (15)	87
2	**CN-2**	brine	toluene (15)	89
3	**CN-3**	brine	toluene (15)	89
4	**CN-4**	brine	toluene (15)	85
5	**CN-5**	brine	toluene (15)	86
6	**CD-1**	brine	toluene (15)	86
7	**CD-2**	brine	toluene (15)	87
8	**CD-3**	brine	toluene (15)	86
9	**CD-4**	brine	toluene (15)	85
10	**CD-5**	brine	toluene (15)	85
11	**CN-1**	CH_2Cl_2	-	32
12	**CN-2**	CH_2Cl_2	-	43
13	**CN-3**	CH_2Cl_2	-	14
14	**CN-4**	CH_2Cl_2	-	9
15	**CN-5**	CH_2Cl_2	-	8
16	**CD-1**	CH_2Cl_2	-	22
17	**CD-2**	CH_2Cl_2	-	27
18	**CD-3**	CH_2Cl_2	-	9
19	**CD-4**	CH_2Cl_2	-	4
20	**CD-5**	CH_2Cl_2	-	4

[a]General reaction conditions: **1a** (0.3 mmol), **4** (0.6 mmol), catalyst (1 mol%) brine (2.0 mL)/toluene (15 equiv) or CH_2Cl_2 (2.0 mL) at 20 °C. [b]Enantiomeric excess (% *ee*) was determined by high-performance liquid chromatography (HPLC).

Supplementary Table 3. Microfluidic asymmetric Mannich reaction with imine (**1a**) with dimethyl malonate (**4**) catalyzed by **CD-2** in various Q_w/Q_o ratio

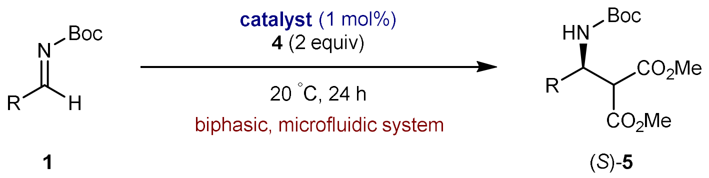

entry	substrate	catalyst	chip channel coating[a]	ID (μm)	solvent	$Q_w : Q_o$ (μL/min)	% *ee*	vol. of org. plug (nL)
1	**1a** (R=Ph)	**CD-2**	hydrophilic	250	brine/*o*-xylene	20:1	91.9	32
2	**1a** (R=Ph)	**CD-2**	hydrophilic	250	brine/*o*-xylene	20:20	91.7	48
3	**1a** (R=Ph)	**CD-2**	hydrophilic	250	brine/*o*-xylene	1:20	91.3	426
4	**1a** (R=Ph)	**CD-2**	hydrophilic	500	brine/*o*-xylene	20:1	92	83
5	**1a** (R=Ph)	**CD-2**	hydrophilic	500	brine/*o*-xylene	20:2	91.1	111
6	**1a (R=Ph)**	**CD-2**	Hydrophilic	500	H_2O/*o*-xylene	20:2	91.1	111
7	**1a** (R=Ph)	**CD-2**	hydrophilic	500	brine/*o*-xylene	20:20	90.7	219
8	**1a** (R=Ph)	**CD-2**	hydrophilic	500	brine/*o*-xylene	2:20	90.0	1429
9	**1a** (R=Ph)	**CD-2**	hydrophilic	500	brine/*o*-xylene	1:20	89.6	2000
10	**1a** (R=Ph)	**CD-2**	hydrophilic	1000	brine/*o*-xylene	20:1	88.3	333
11	**1a** (R=Ph)	**CD-2**	hydrophilic	1000	brine/*o*-xylene	20:20	88.1	1000
12	**1a** (R=Ph)	**CD-2**	hydrophilic	1000	brine/*o*-xylene	1:20	84.2	13333
13*	**1a** (R=Ph)	**CD-2**	hydrophilic	1000	brine/*o*-xylene	1:20	89.6	13333
14	**1a** (R=Ph)	**CN-2**	hydrophilic	500	NaCl/D_2O[b]/*o*-xylene	20:2	91.9	111
15	**1f** (R=3-MePh)	**CN-2**	hydrophilic	500	NaCl/D_2O[b]/*o*-xylene	20:2	94.8	111
16	**1a** (R=Ph)	**CN-2**	hydrophobic	500	brine/*o*-xylene	20:2	91.6	111

[a]Dolomite 3D flow focusing chip-100μm-hydrophilic channel coating (Part No. 3200435) and Dolomite 3D flow focusing chip-100μm-hydrophobic channel coating (Part No. 3200436) were used. [b]Saturated solutions.

***Comments:** The reaction was conducted under ca. 5 bar. The pressure within microfluidic system ($\o$ = 1000 μm, Q_w/Q_o = 1/20) was controlled by a back pressure regulator. A remarkably higher enantioselectivity was observed under high pressure conditions (from 84% *ee* under 1 bar (entry 12) to 90% *ee* under ca. 5 bar (entry 13)).

Supplementary Table 4. Kinetic study under the biphasic microfluidic condition

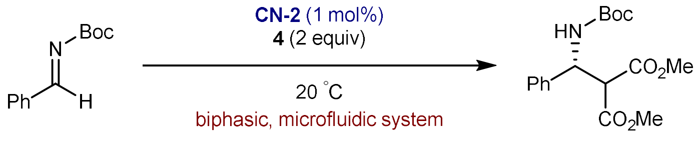

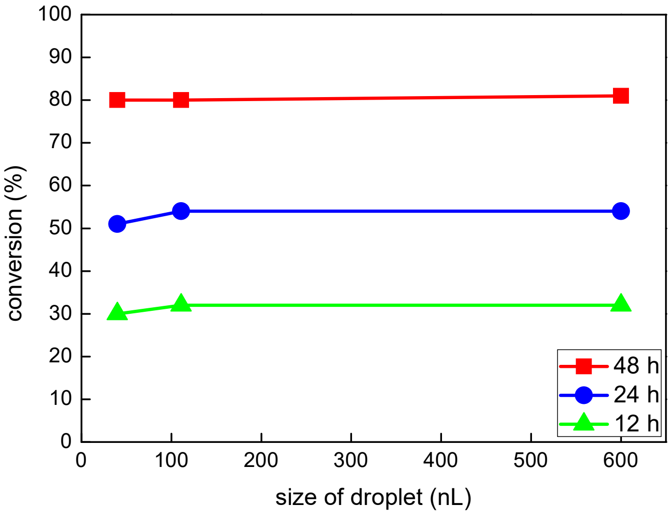

Time (h)	conversion in 40 nL (%)	conversion in 111 nL (%)	conversion in 600 nL (%)
12	30	32	32
24	51	54	54
48	80	80	81

Supplementary Figures

NMR Spectra

NMR Spectra of catalysts

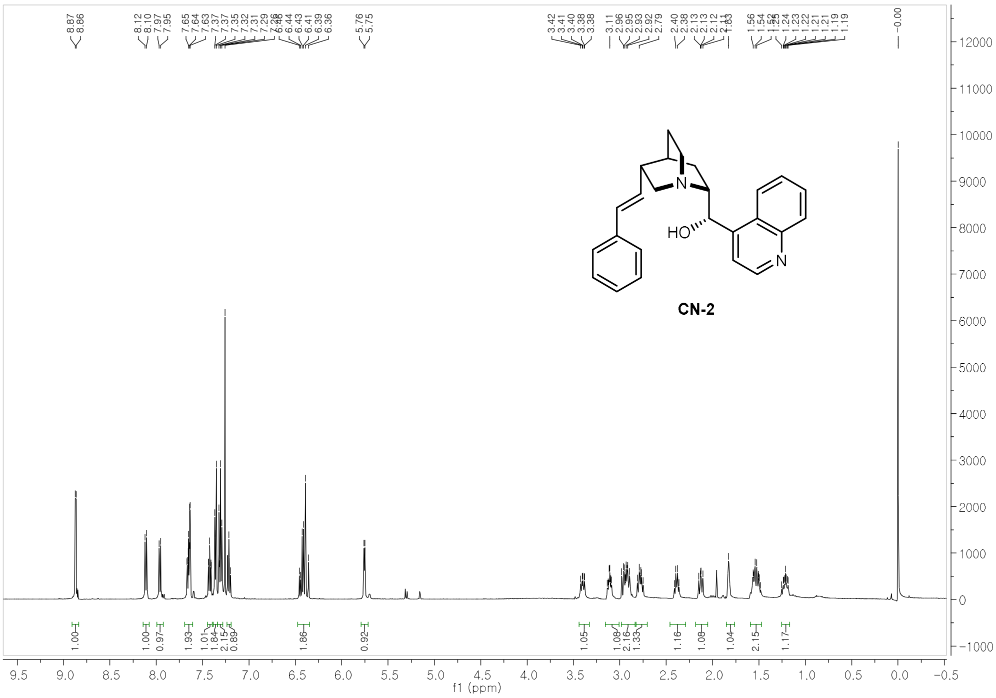

Supplementary Figure 5. ^{1}H NMR spectra of **CN-2**

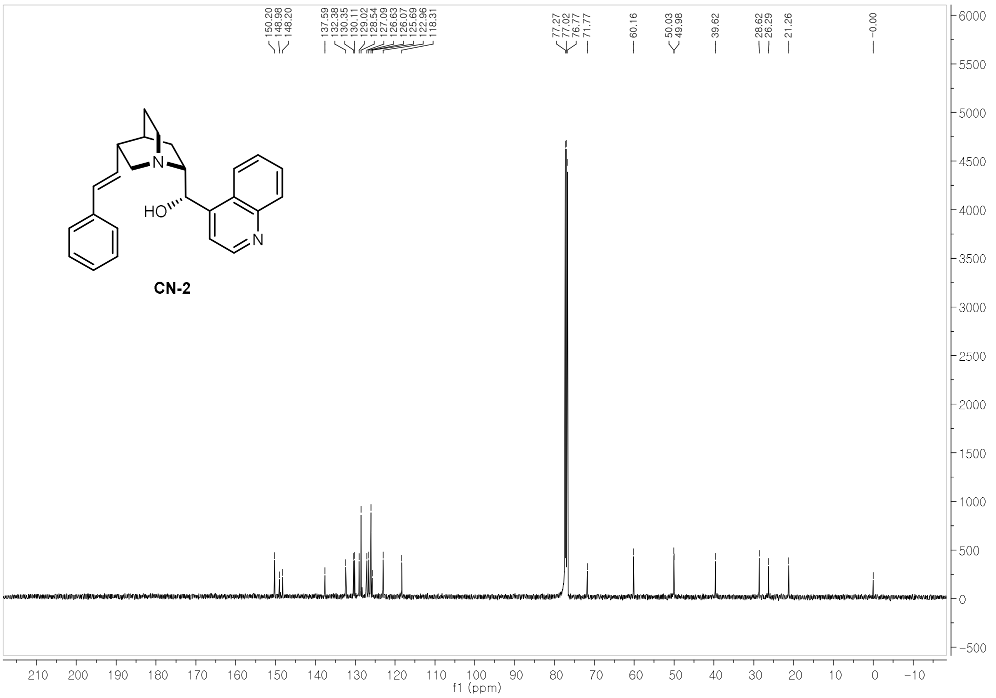

Supplementary Figure 6. ^{13}C NMR spectra of **CN-2**

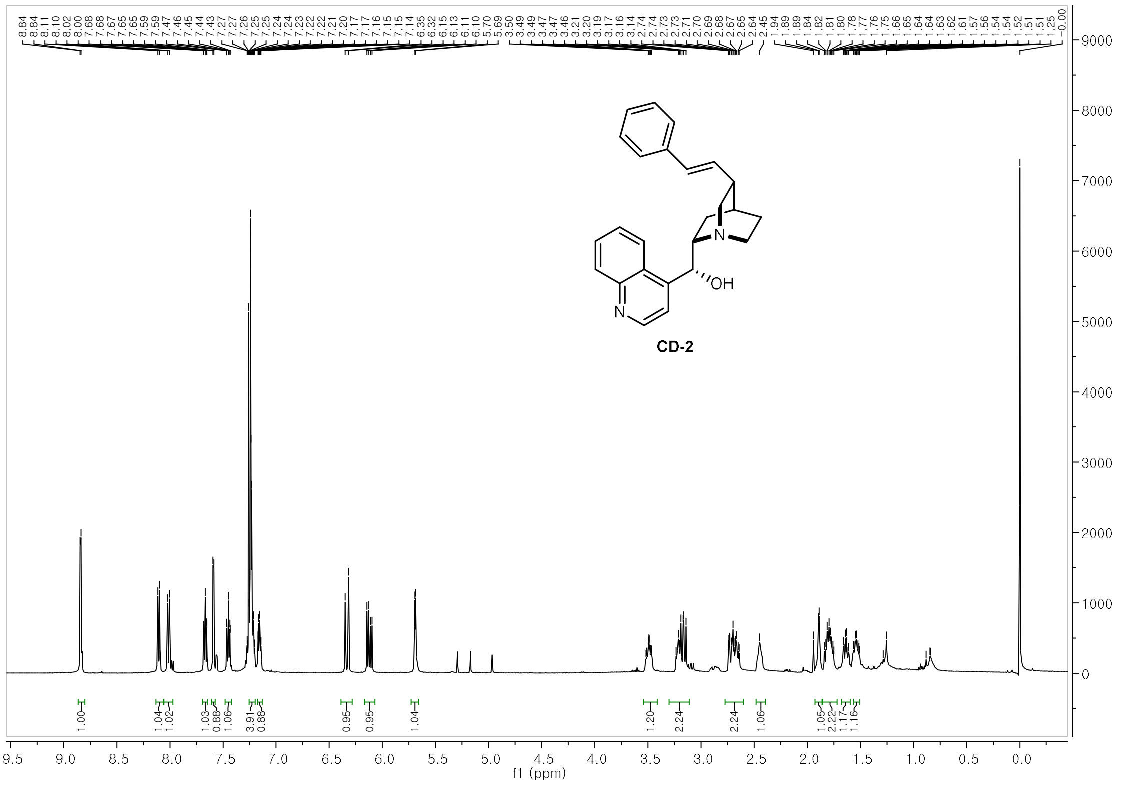

Supplementary Figure 7. ^{1}H NMR spectra of **CD-2**

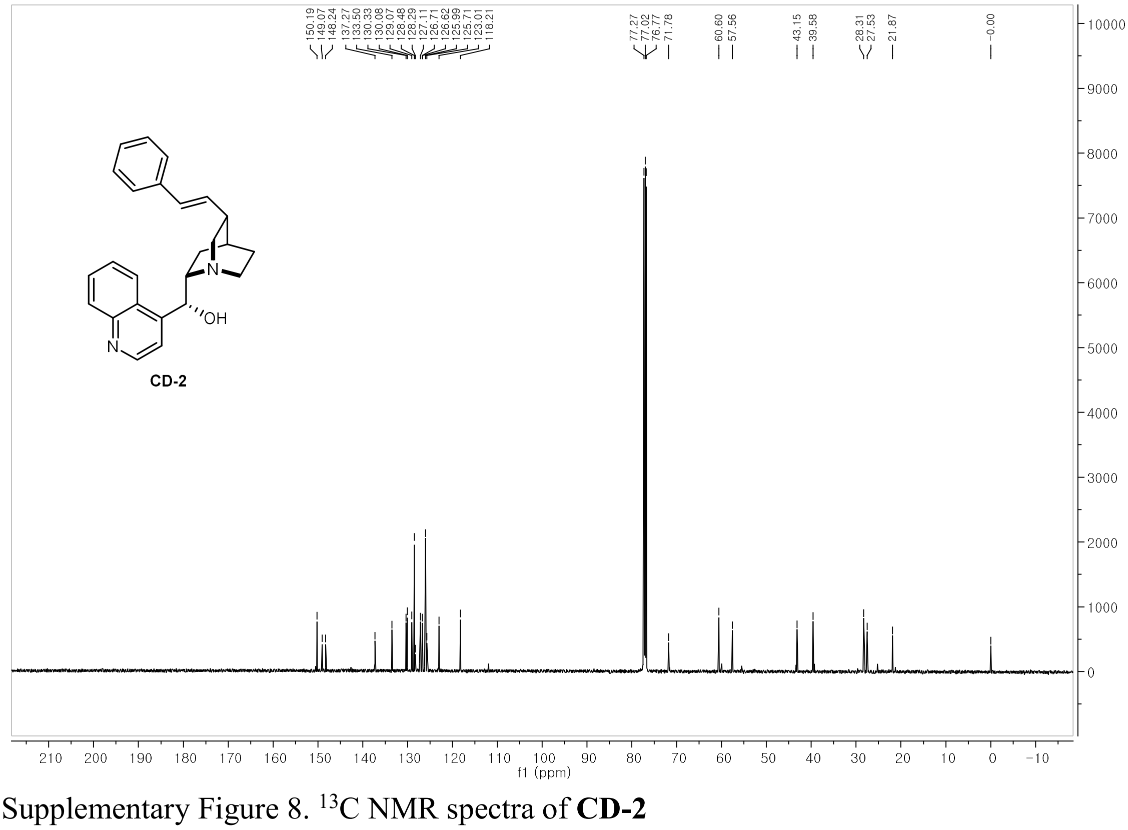

Supplementary Figure 8. ^{13}C NMR spectra of **CD-2**

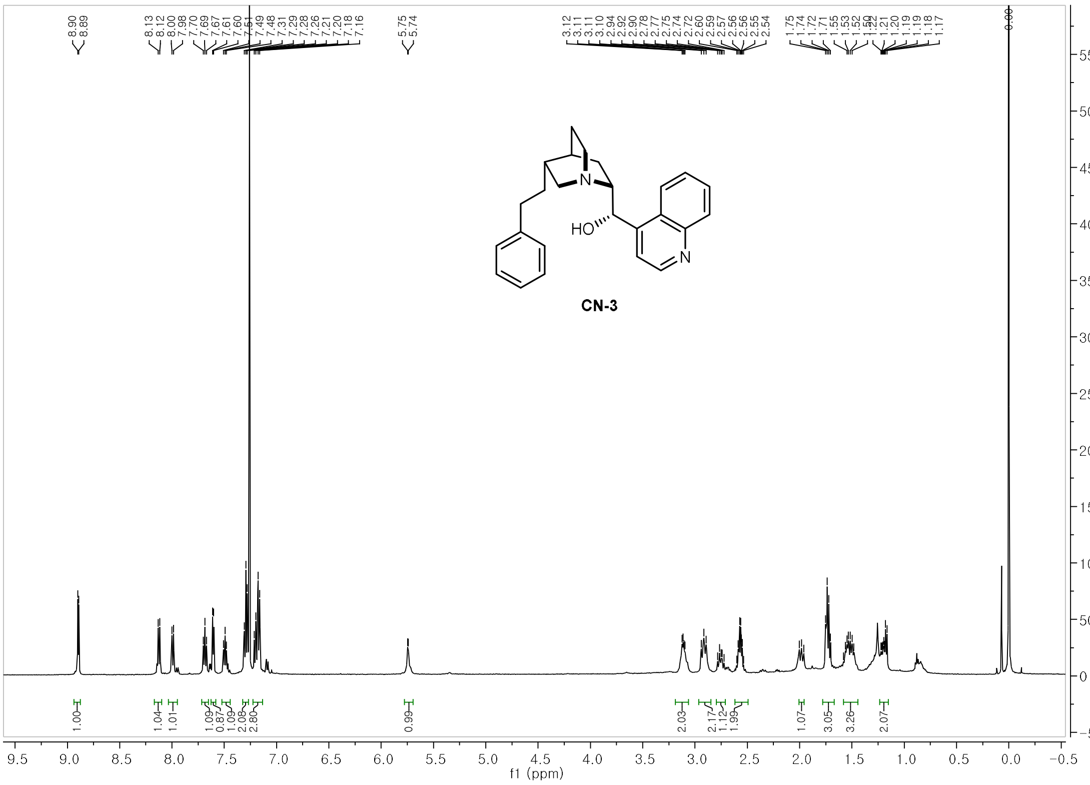

Supplementary Figure 9. ^{1}H NMR spectra of **CN-3**

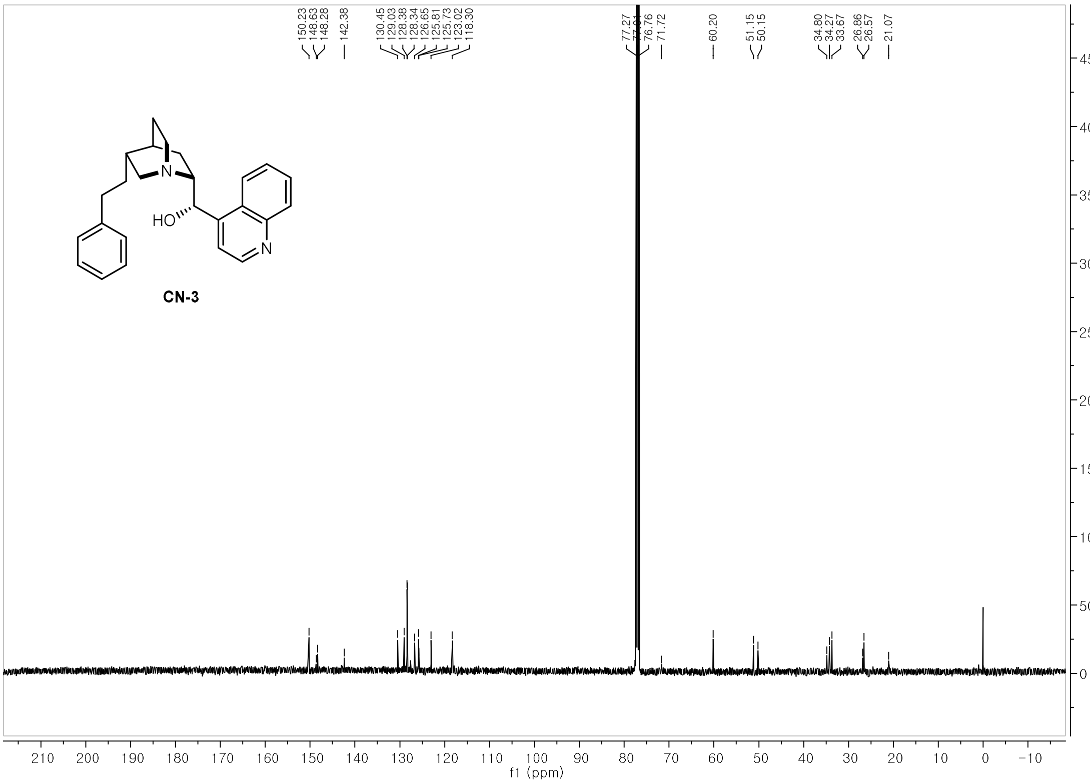

Supplementary Figure 10. ^{13}C NMR spectra of **CN-3**

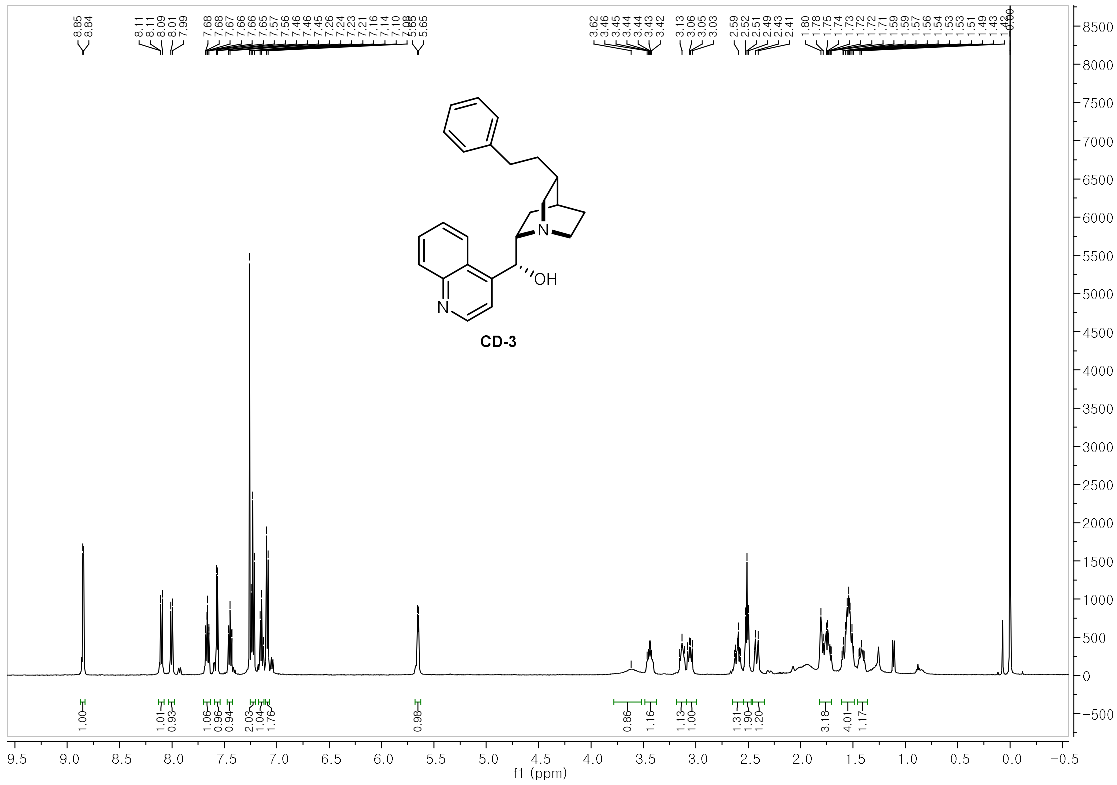

Supplementary Figure 11. ^{1}H NMR spectra of **CD-3**

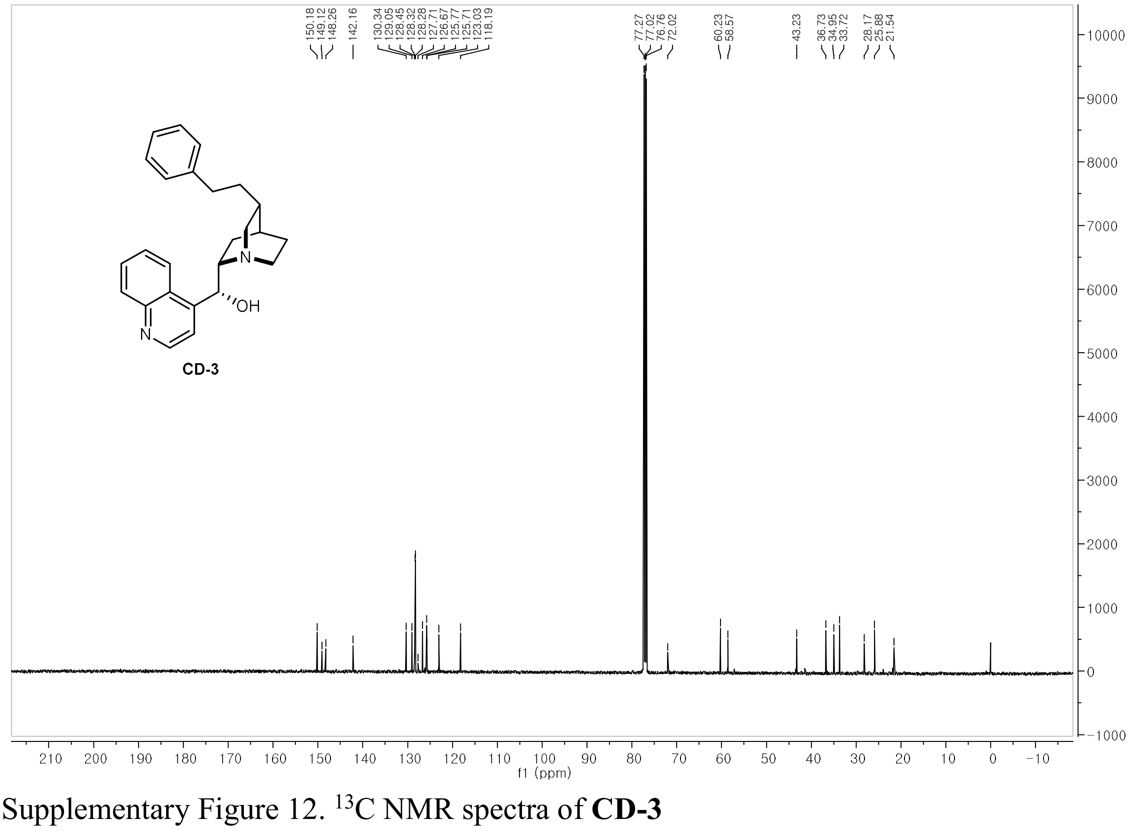

Supplementary Figure 12. ^{13}C NMR spectra of **CD-3**

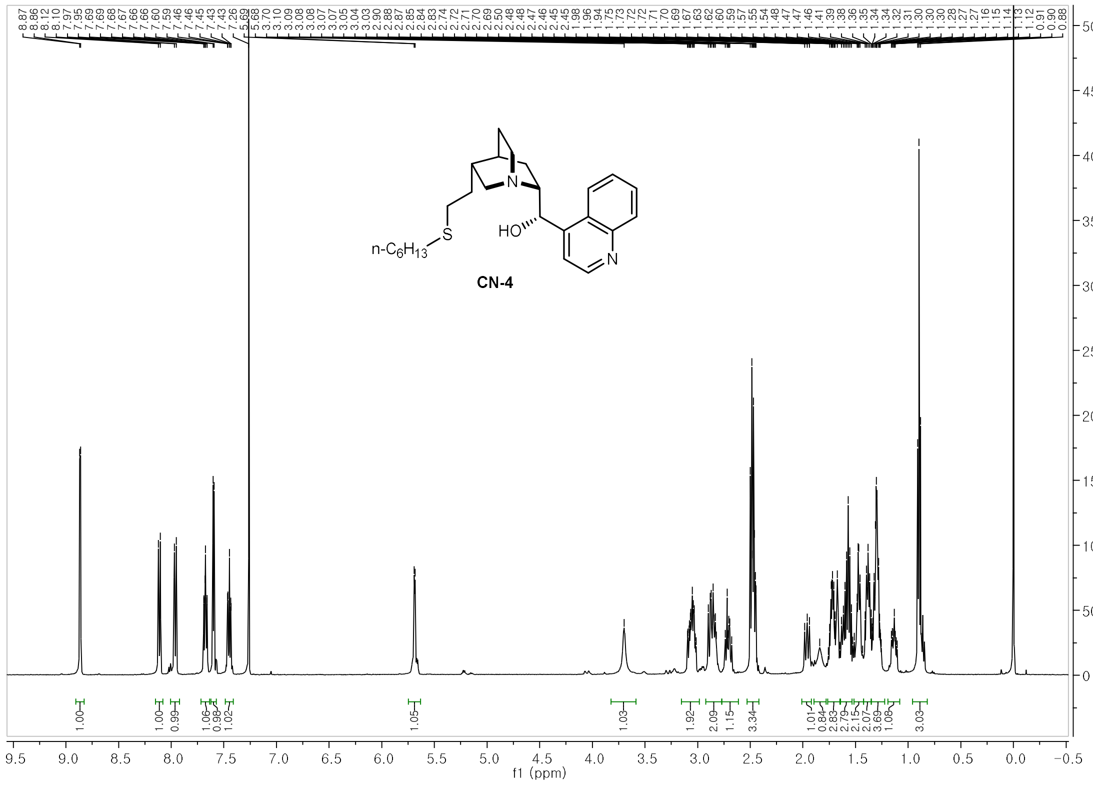

Supplementary Figure 13. ^{1}H NMR spectra of **CN-4**

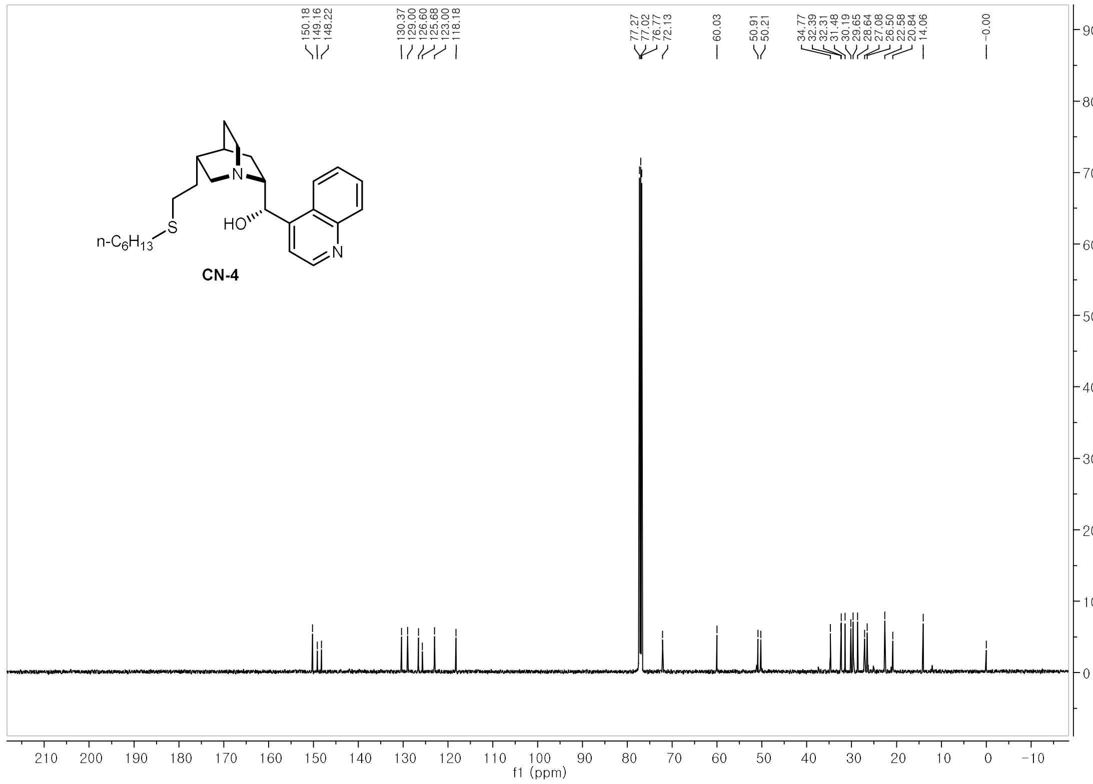

Supplementary Figure 14. ^{13}C NMR spectra of **CN-4**

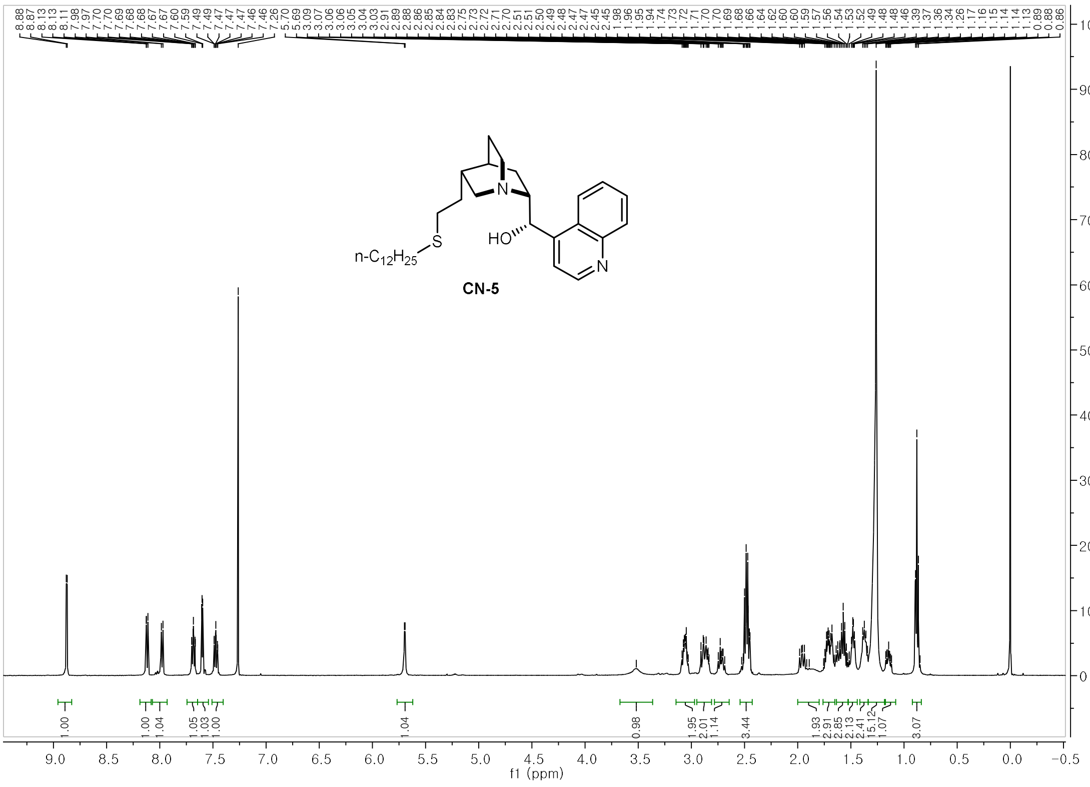

Supplementary Figure 15. ^{1}H NMR spectra of **CN-5**

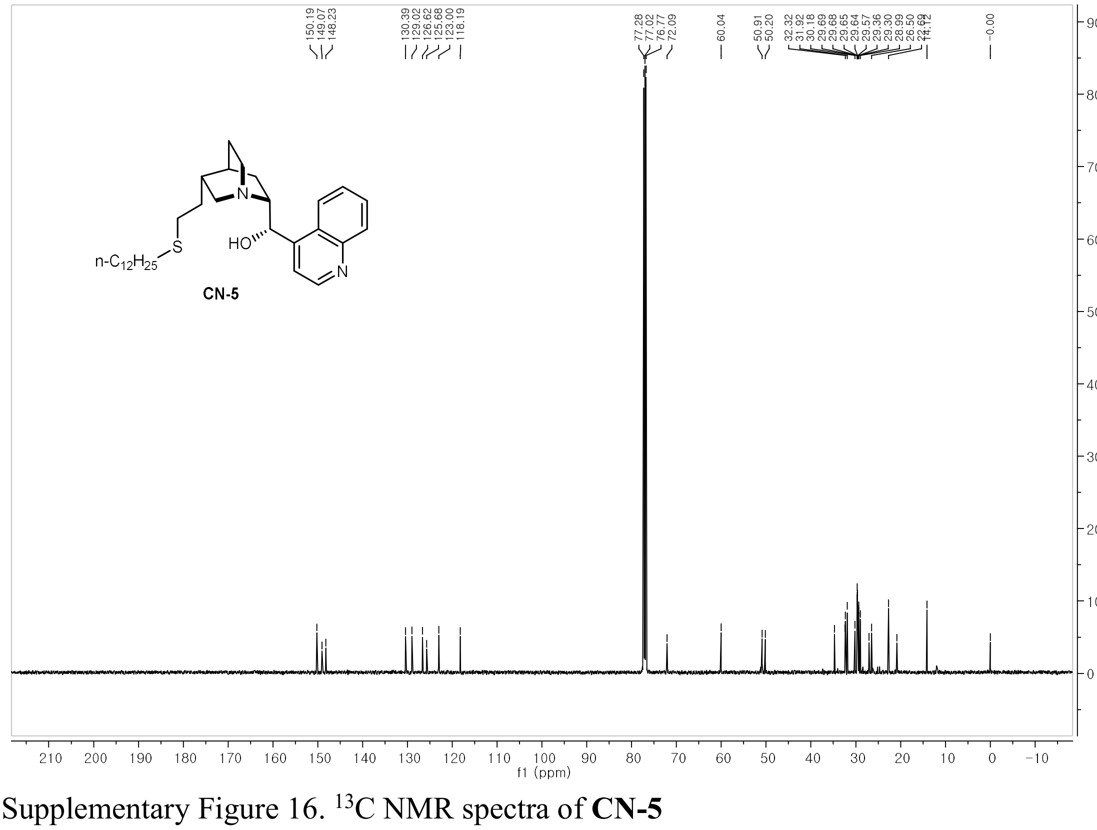

Supplementary Figure 16. ^{13}C NMR spectra of **CN-5**

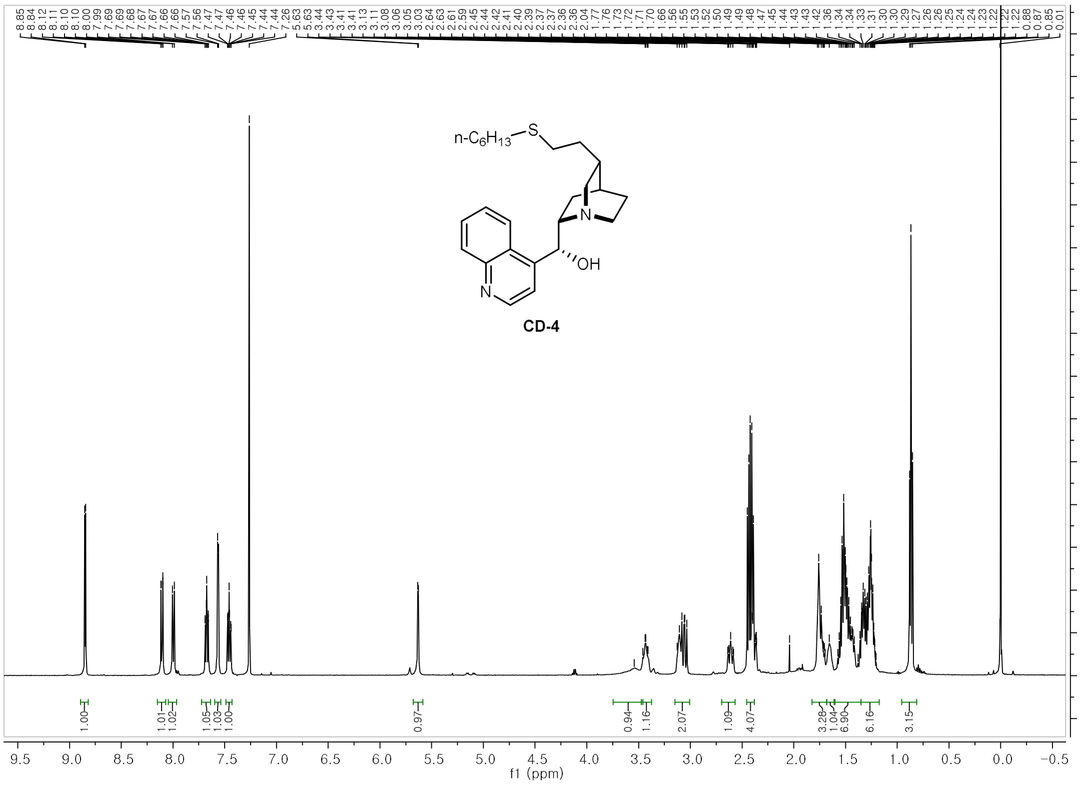

Supplementary Figure 17. ^{1}H NMR spectra of **CD-4**

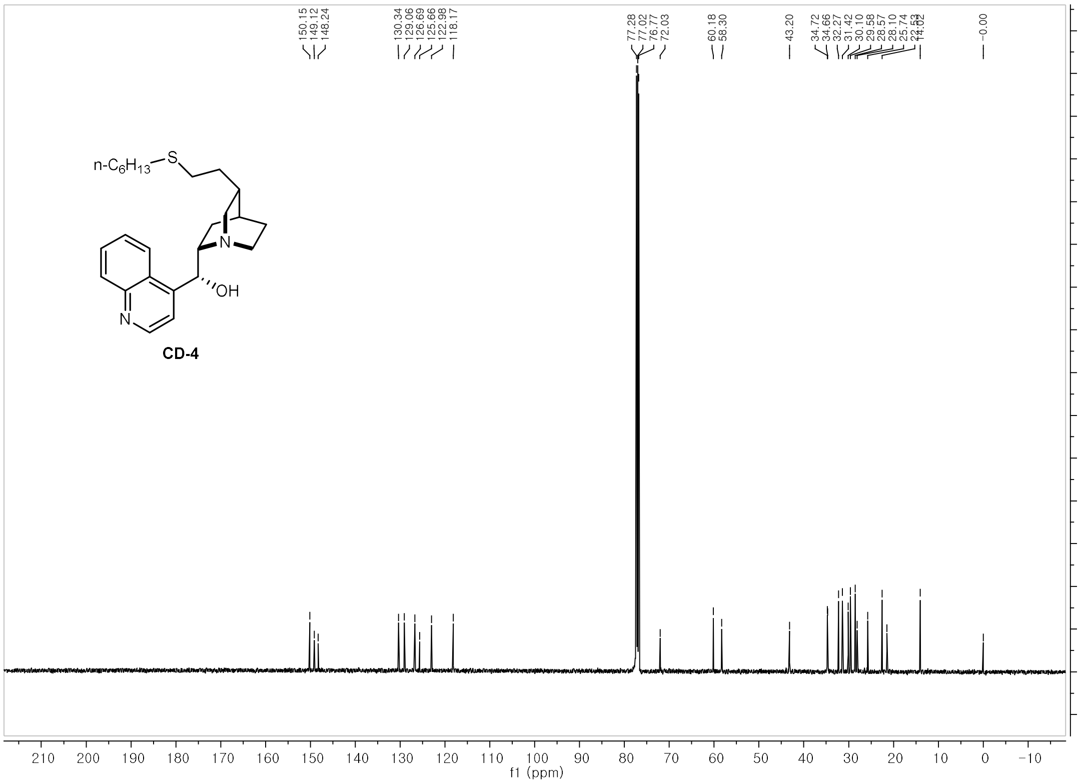

Supplementary Figure 18. ^{13}C NMR spectra of **CD-4**

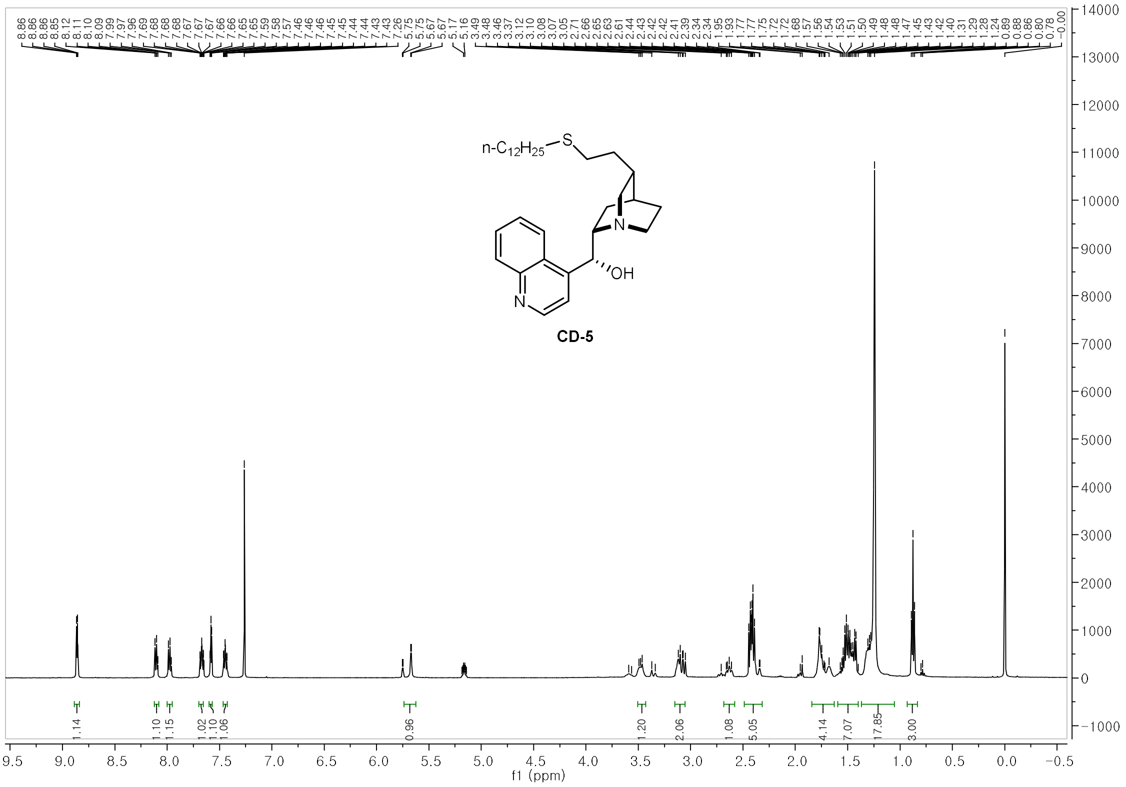

Supplementary Figure 19. ^{1}H NMR spectra of **CD-5**

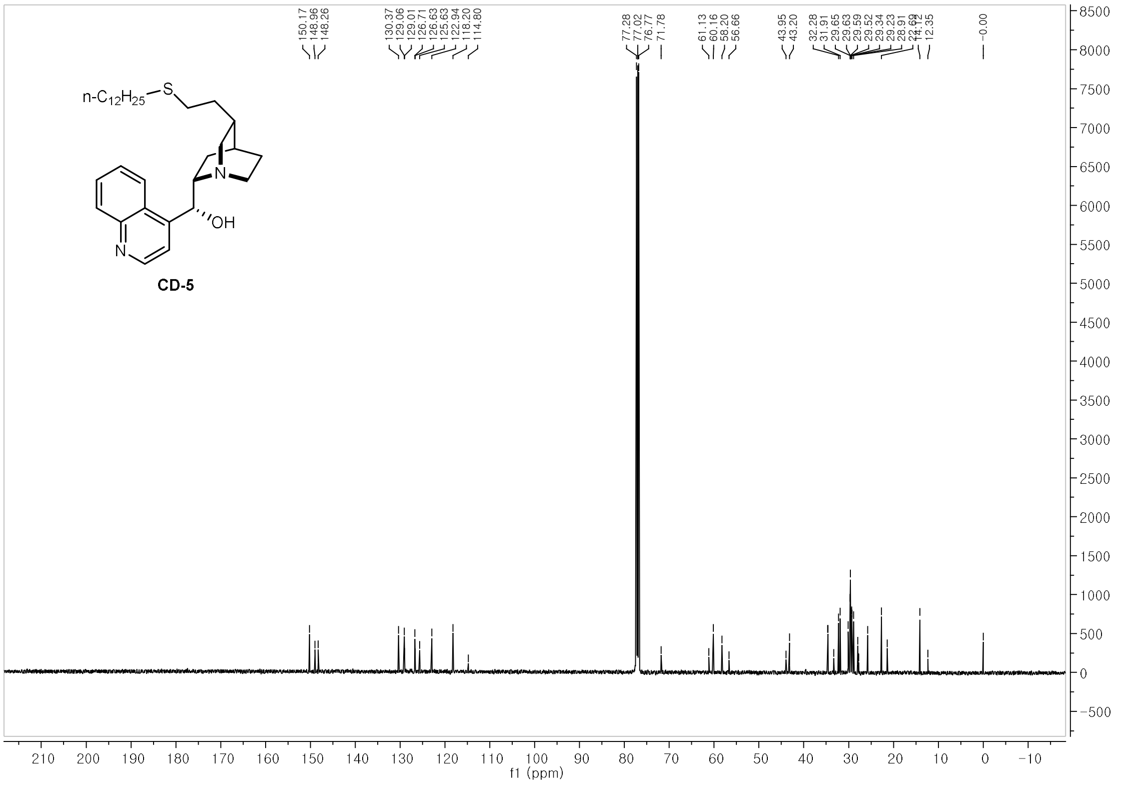

Supplementary Figure 20. ^{13}C NMR spectra of **CD-5**

NMR Spectra of substrates

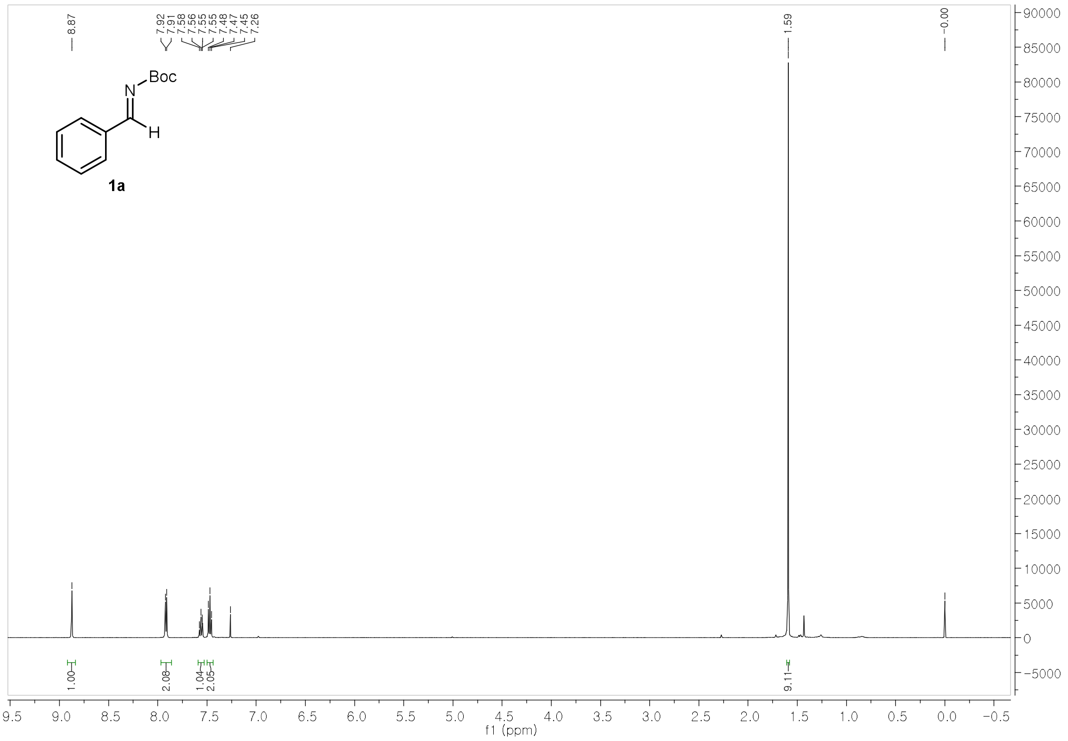

Supplementary Figure 21. ^{1}H NMR spectra of *N*-Boc imine **1a**

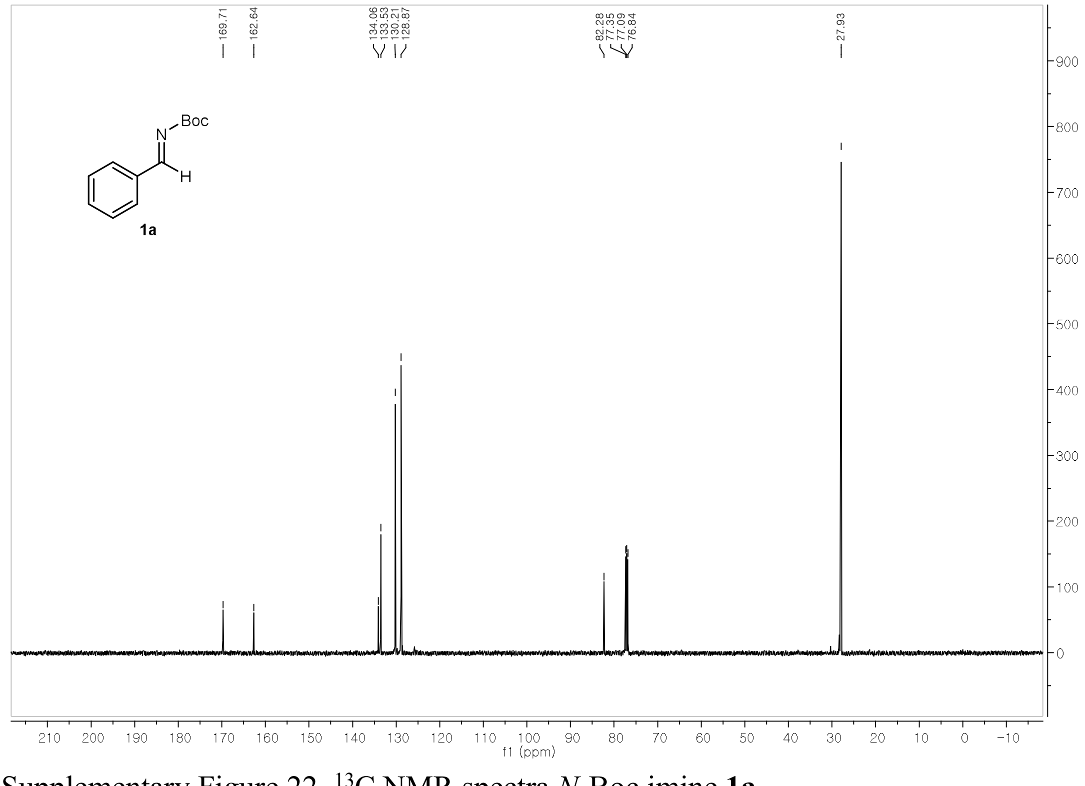

Supplementary Figure 22. ^{13}C NMR spectra *N*-Boc imine **1a**

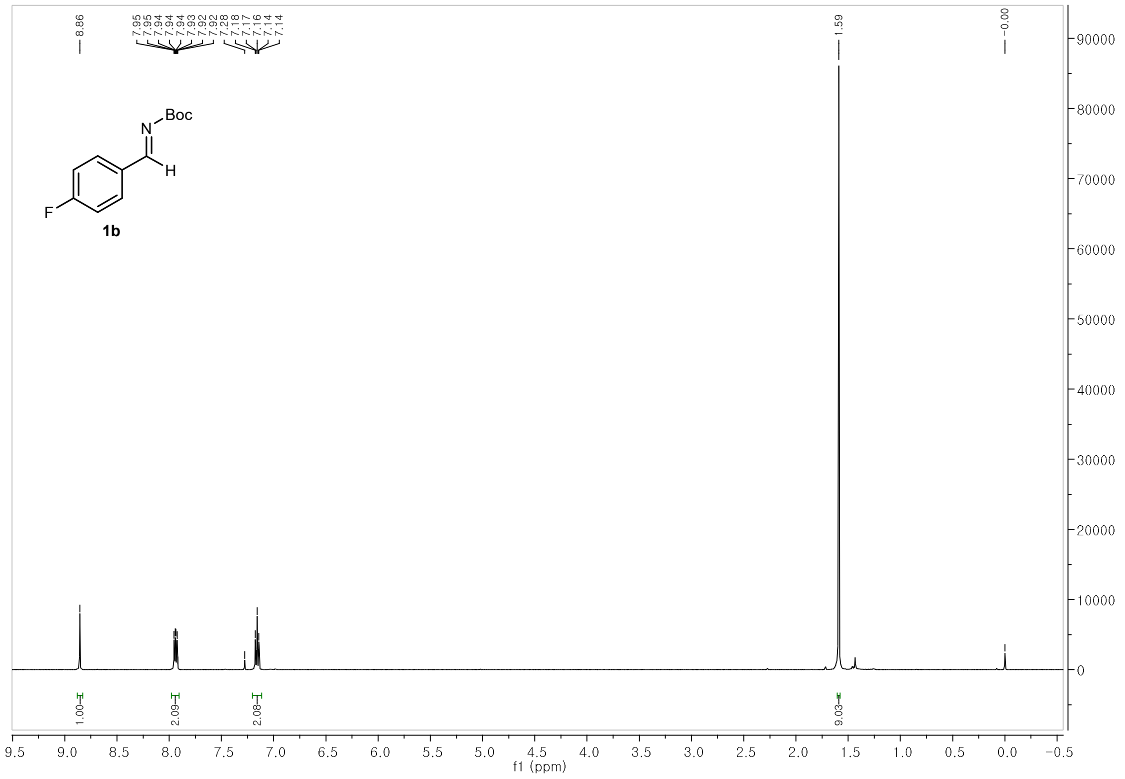

Supplementary Figure 23. ^{1}H NMR spectra of *N*-Boc imine **1b**

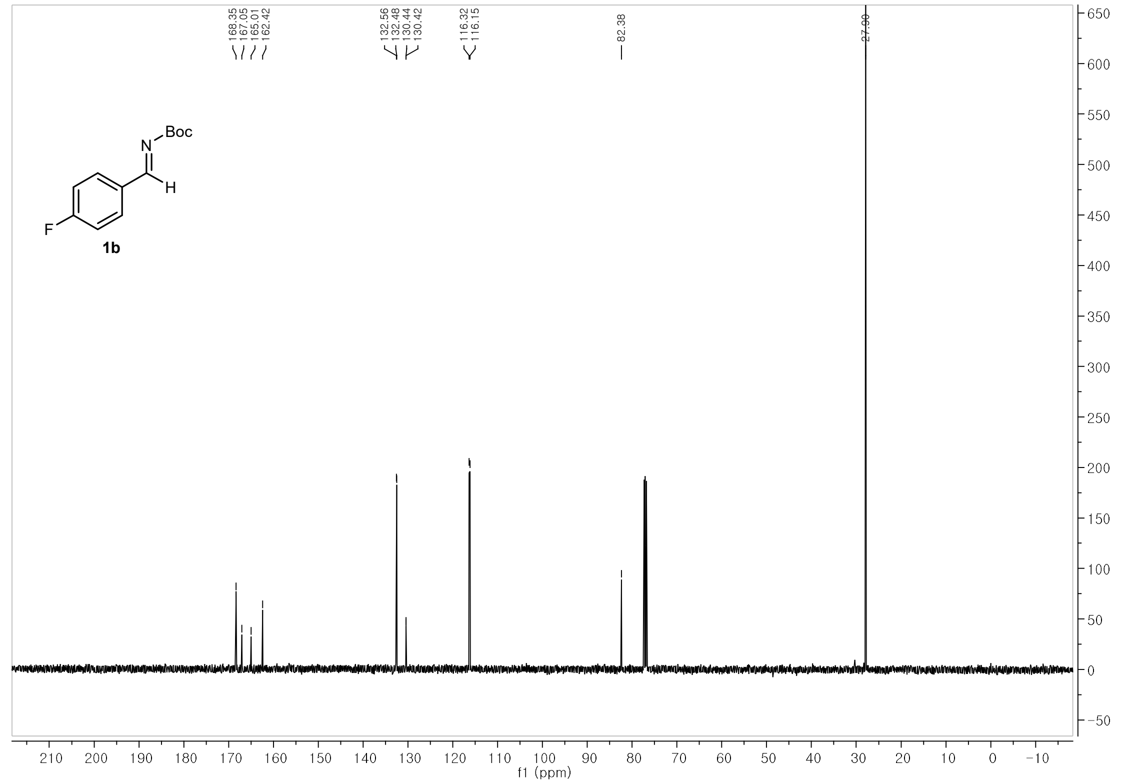

Supplementary Figure 24. ^{13}C NMR spectra *N*-Boc imine **1b**

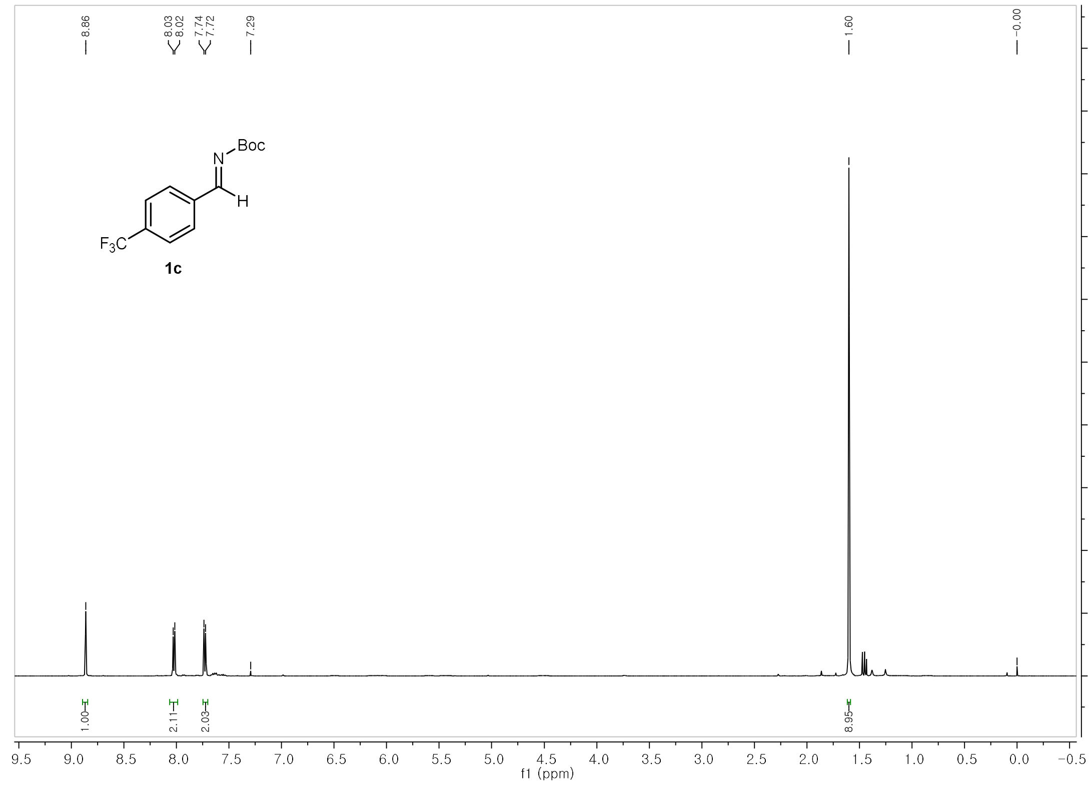

Supplementary Figure 25. ^{1}H NMR spectra of *N*-Boc imine **1c**

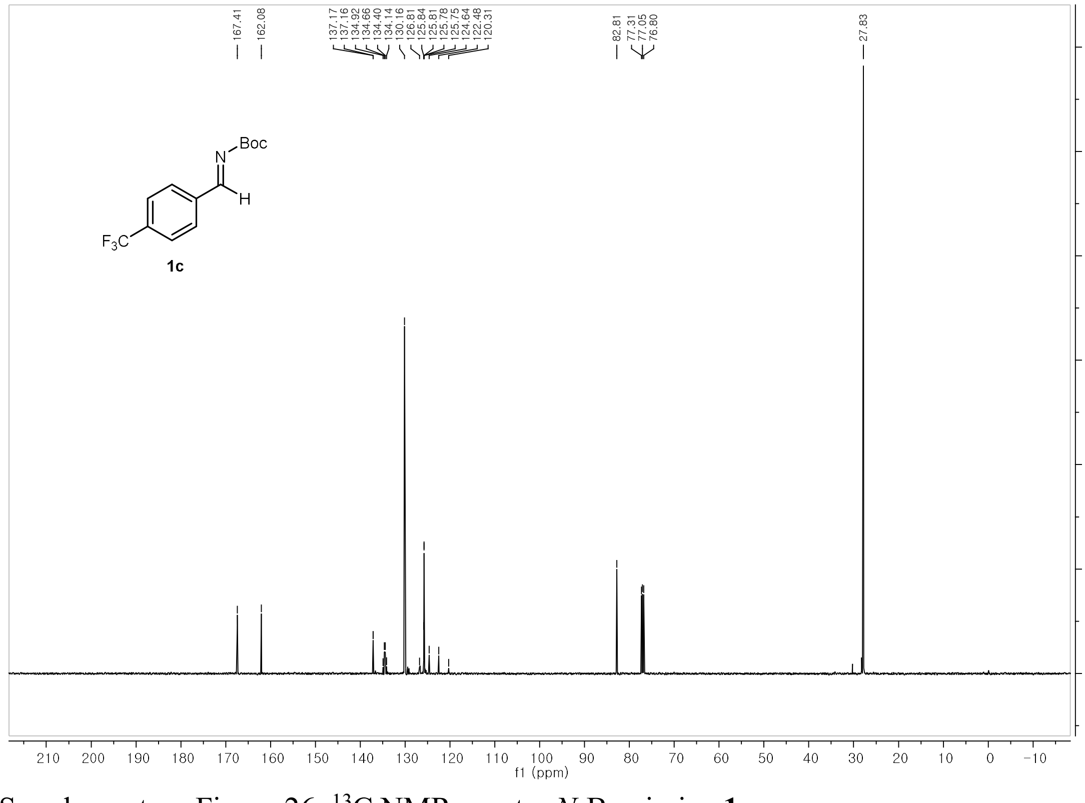

Supplementary Figure 26. ^{13}C NMR spectra *N*-Boc imine **1c**

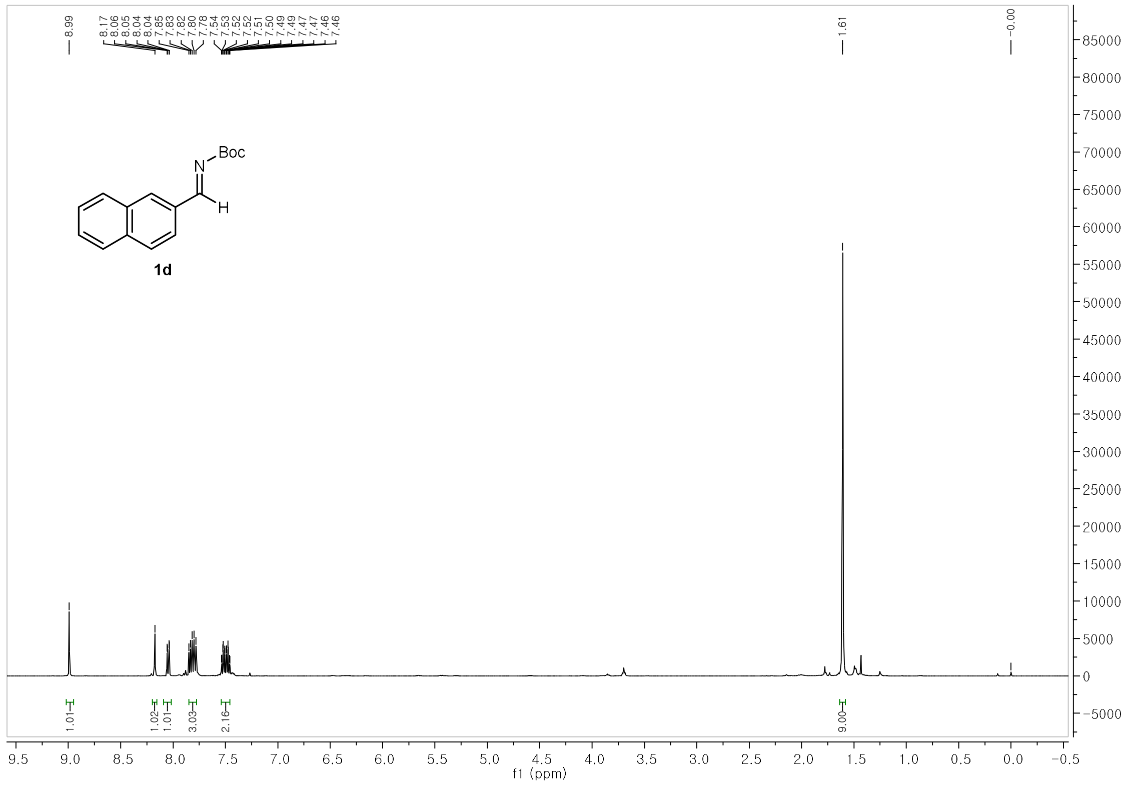

Supplementary Figure 27. ^{1}H NMR spectra of *N*-Boc imine **1d**

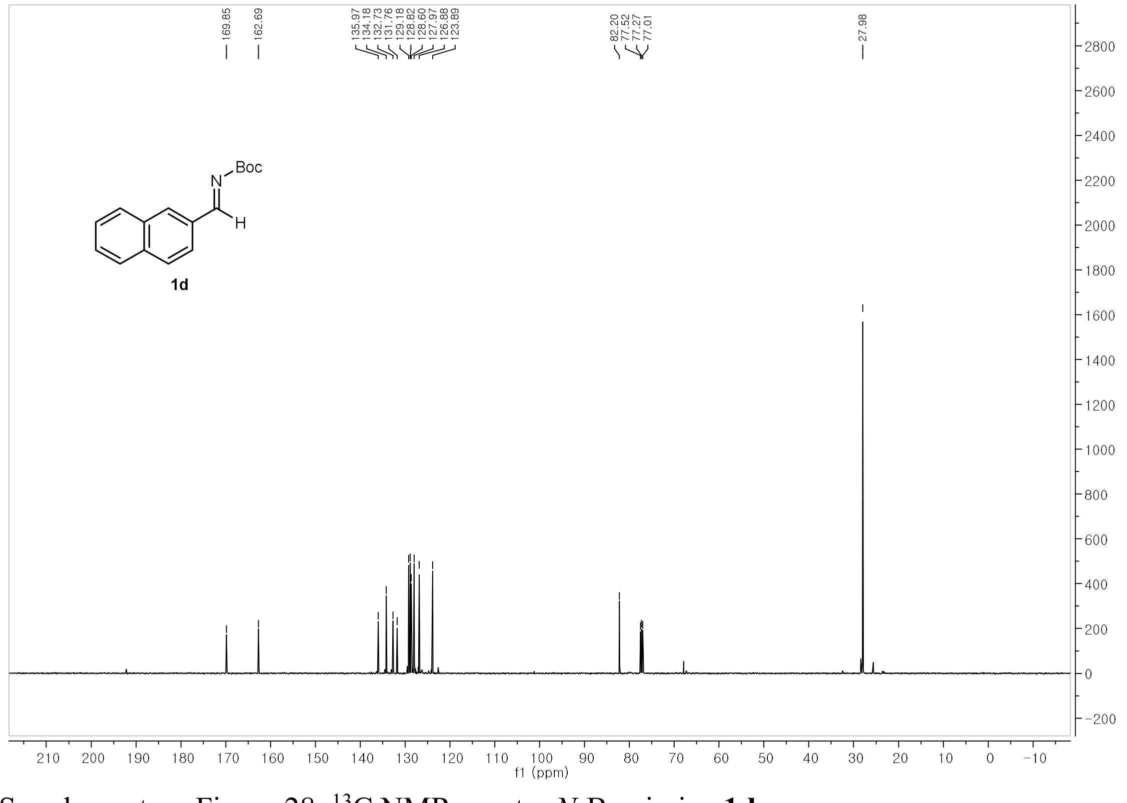

Supplementary Figure 28. ^{13}C NMR spectra *N*-Boc imine **1d**

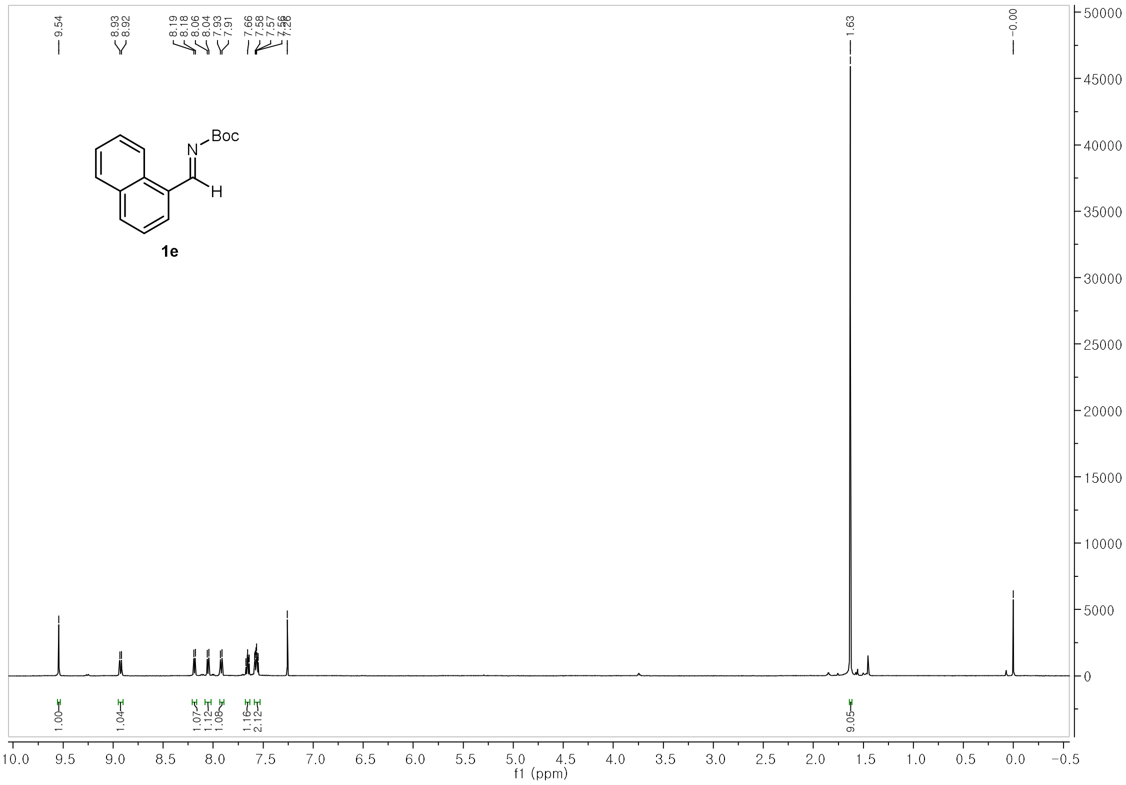

Supplementary Figure 29. ^{1}H NMR spectra of *N*-Boc imine **1e**

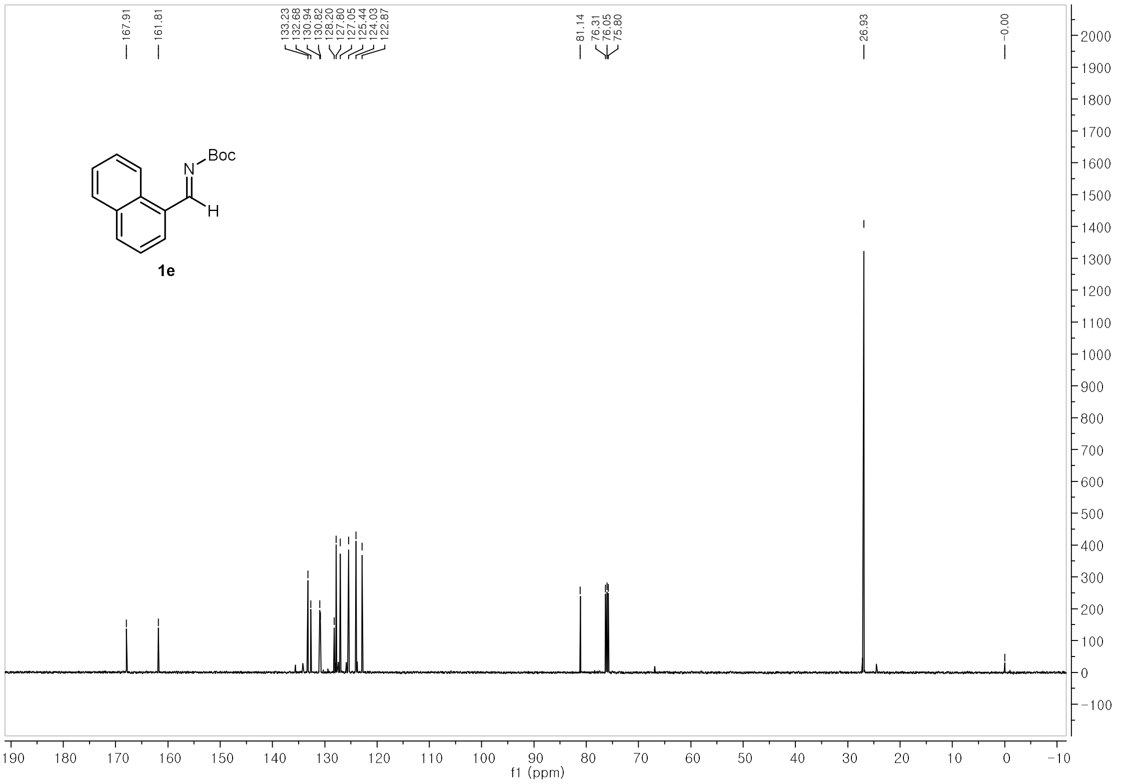

Supplementary Figure 30. ^{13}C NMR spectra *N*-Boc imine **1e**

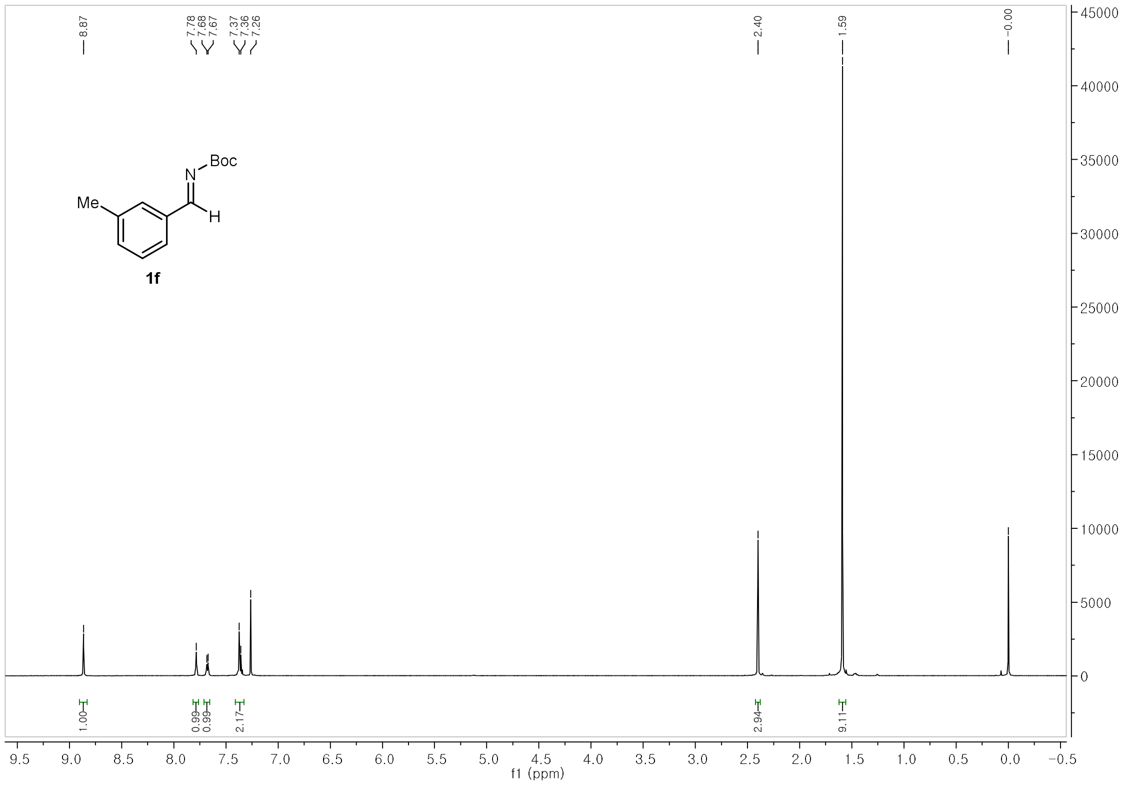

Supplementary Figure 31. ^{1}H NMR spectra of *N*-Boc imine **1f**

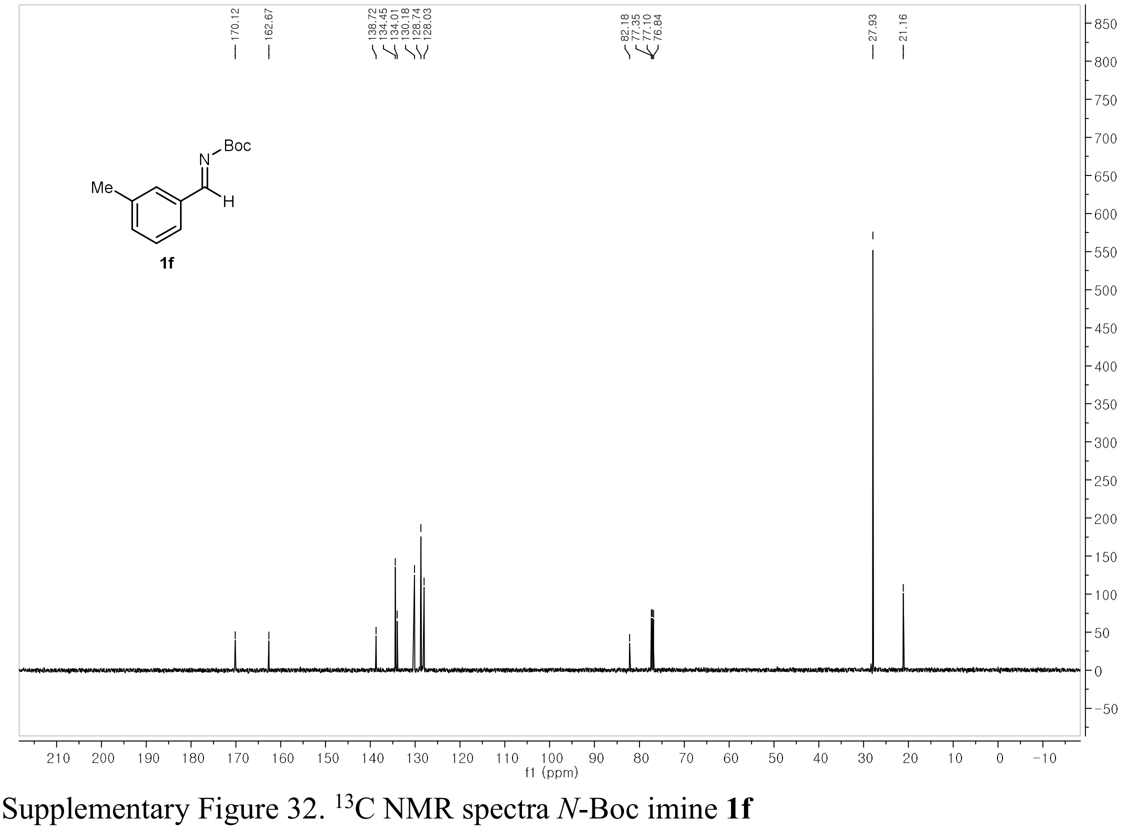

Supplementary Figure 32. ^{13}C NMR spectra *N*-Boc imine **1f**

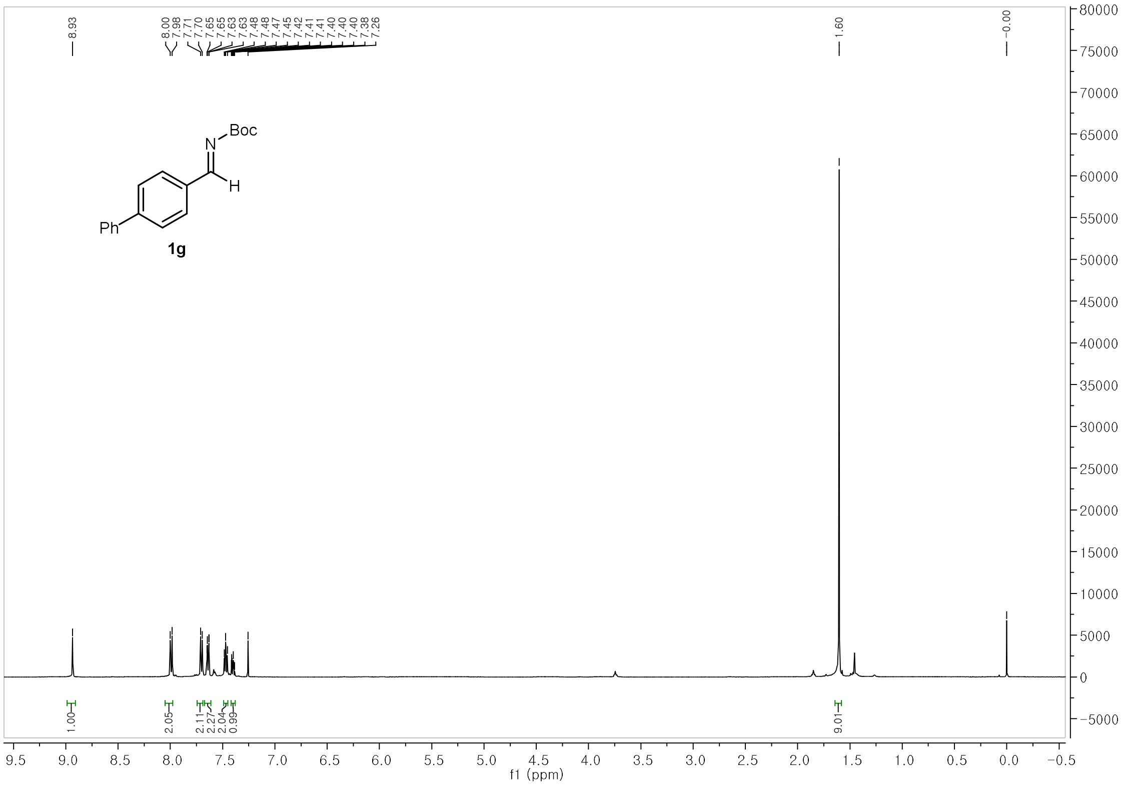

Supplementary Figure 33. ^{1}H NMR spectra of *N*-Boc imine **1g**

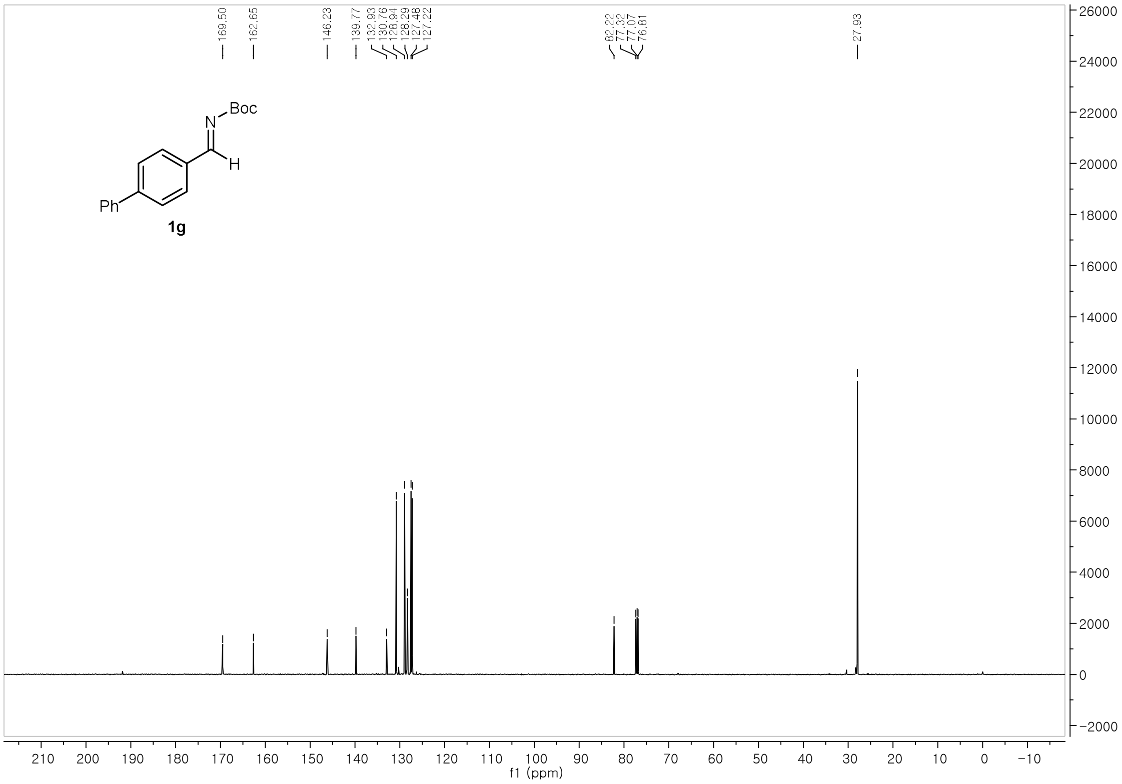

Supplementary Figure 34. ^{13}C NMR spectra *N*-Boc imine **1g**

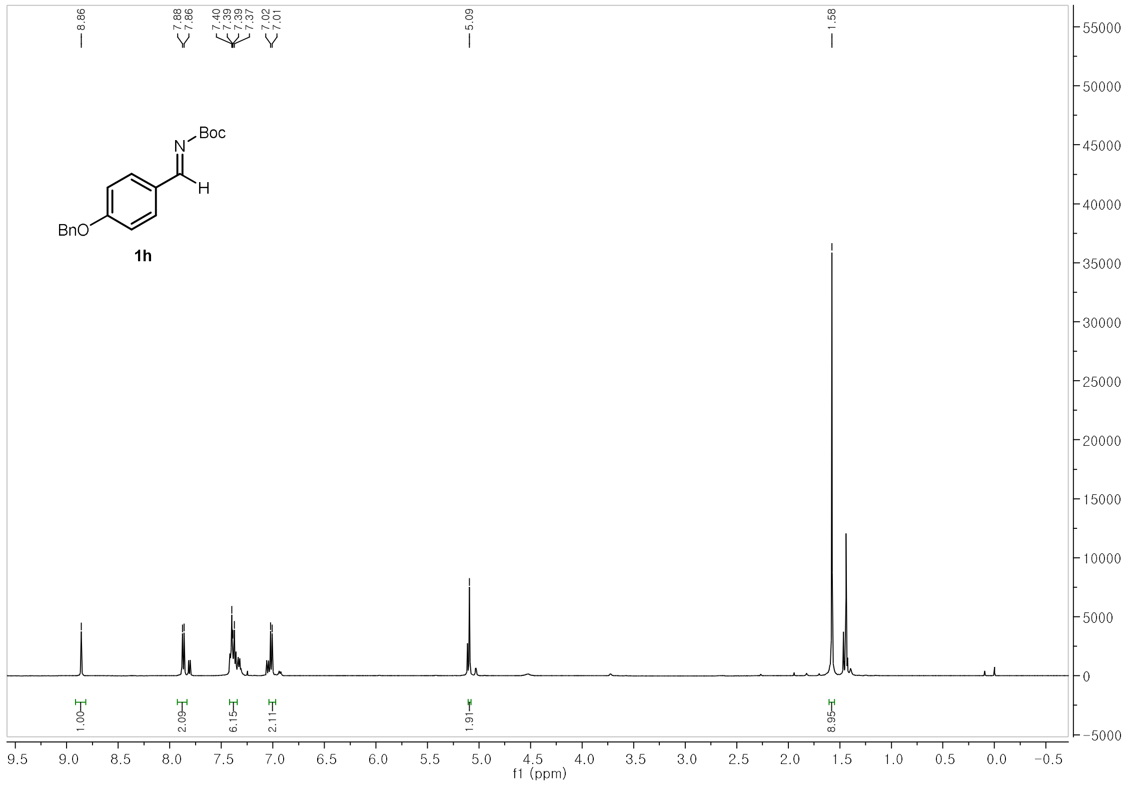

Supplementary Figure 35. ^{1}H NMR spectra of *N*-Boc imine **1h**

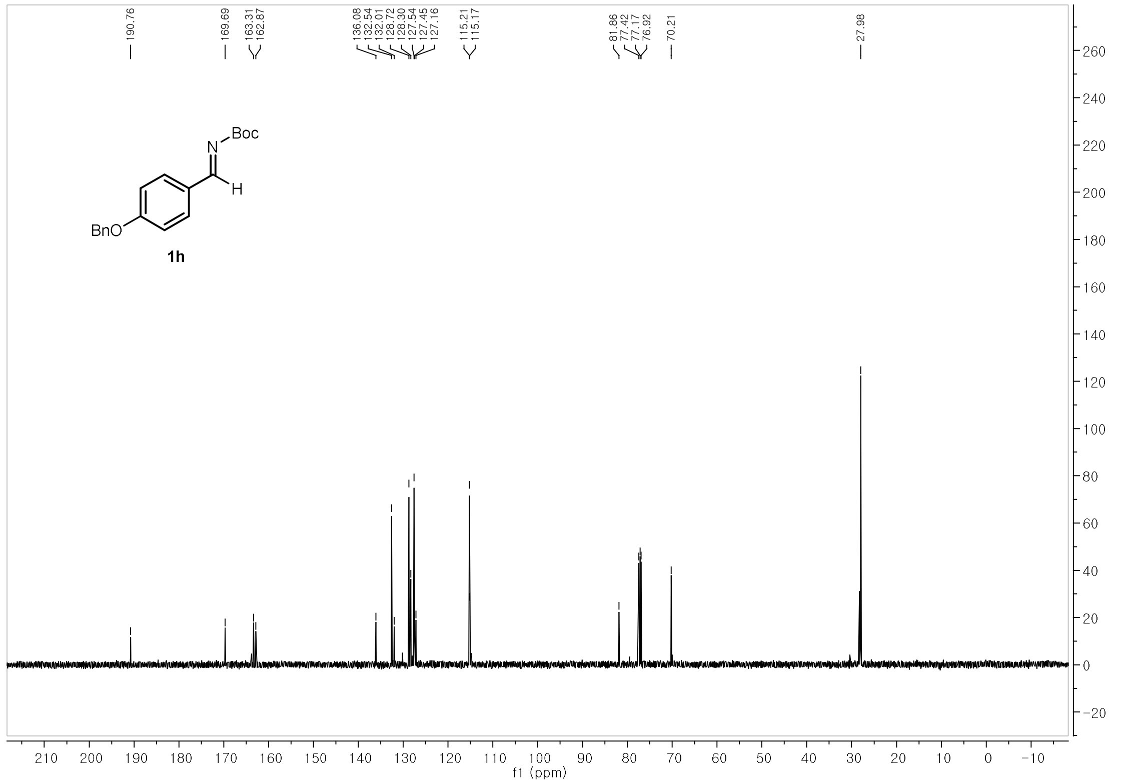

Supplementary Figure 36. ^{13}C NMR spectra *N*-Boc imine **1h**

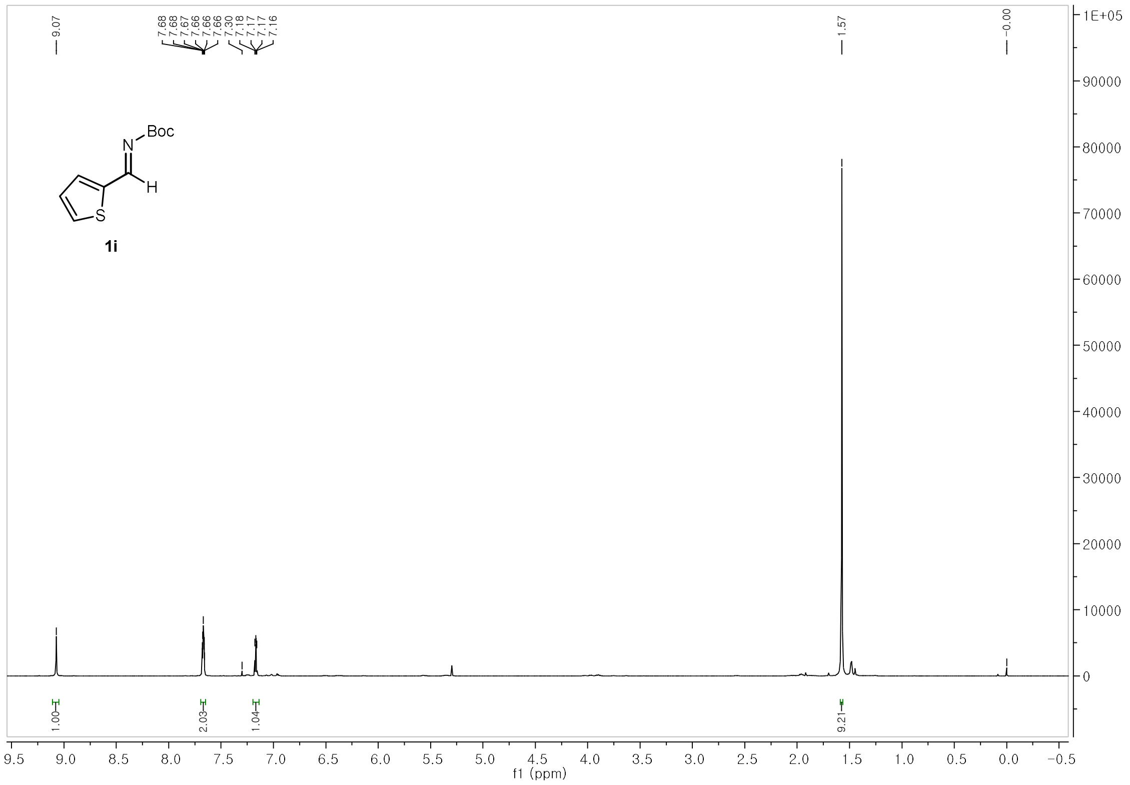

Supplementary Figure 37. ^{1}H NMR spectra of *N*-Boc imine **1i**

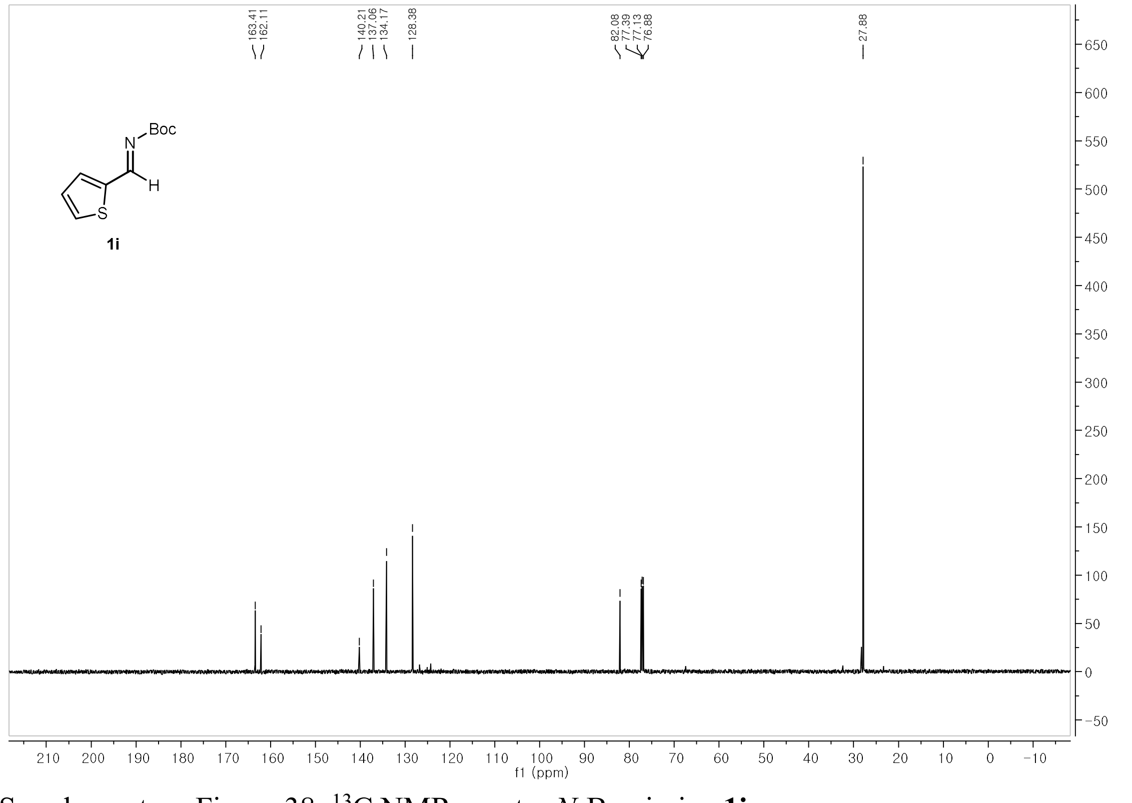

Supplementary Figure 38. ^{13}C NMR spectra *N*-Boc imine **1i**

NMR spectra of Mannich products

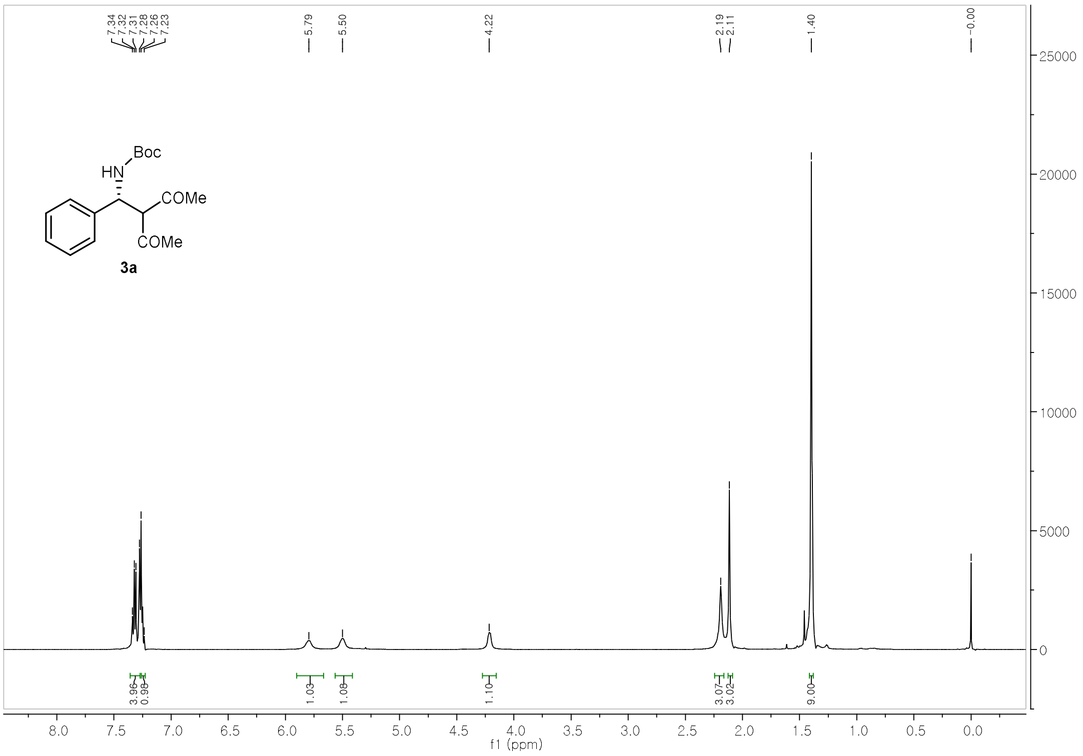

Supplementary Figure 39. ^{1}H NMR spectra of Mannich product **3a**

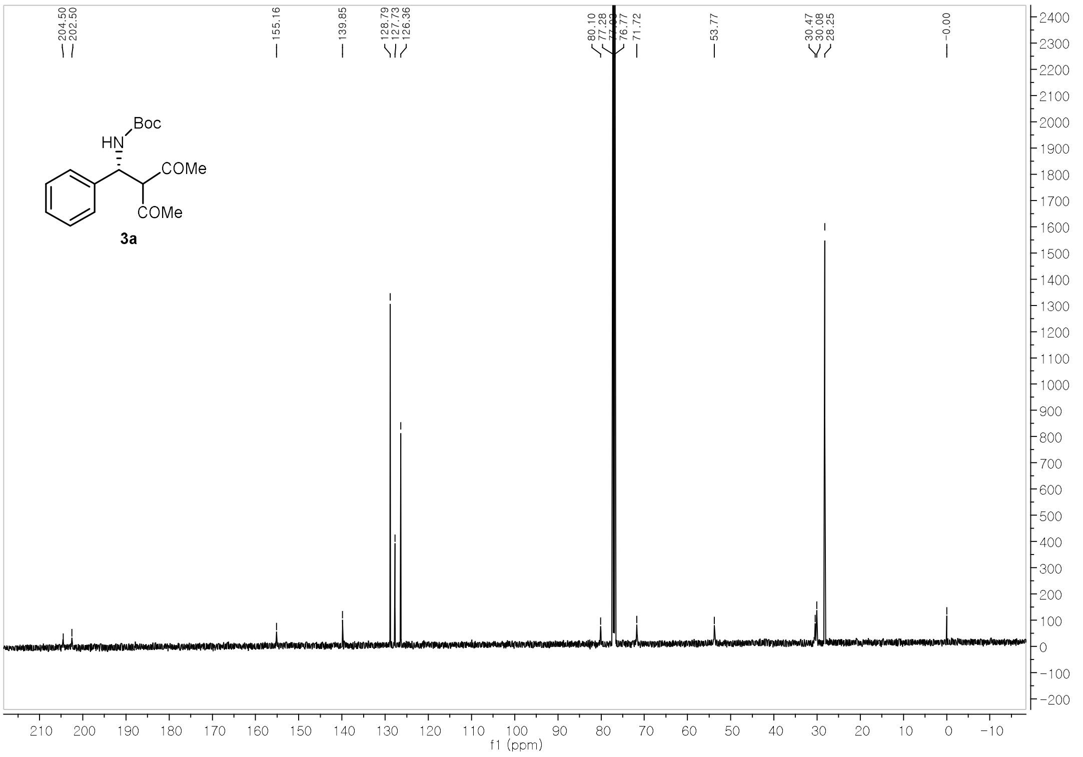

Supplementary Figure 40. ^{13}C NMR spectra of Mannich product **3a**

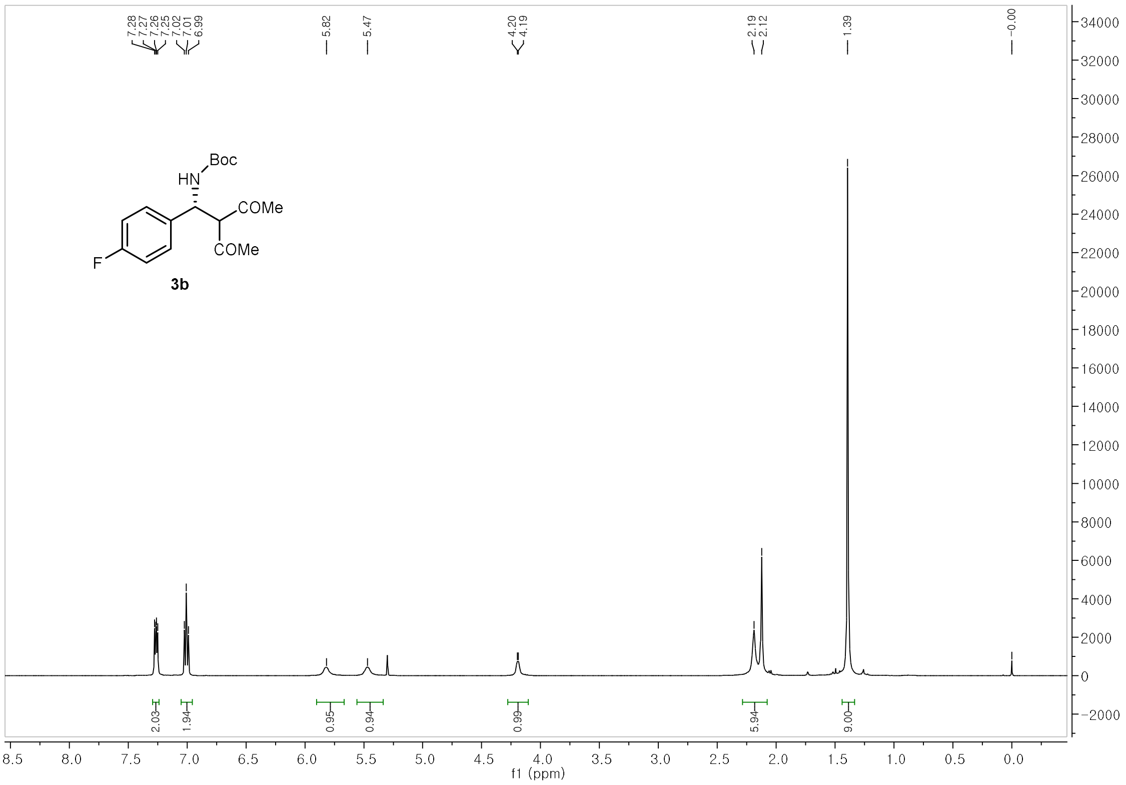

Supplementary Figure 41. ^{1}H NMR spectra of Mannich product **3b**

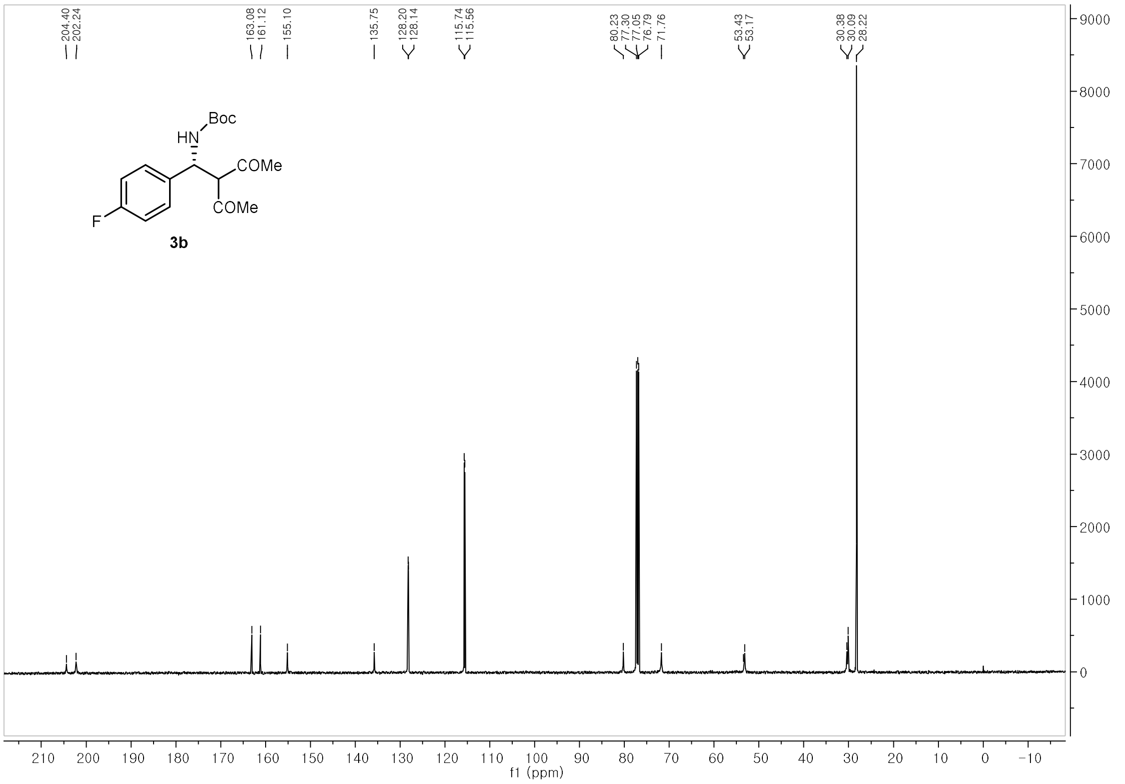

Supplementary Figure 42. ^{13}C NMR spectra of Mannich product **3b**

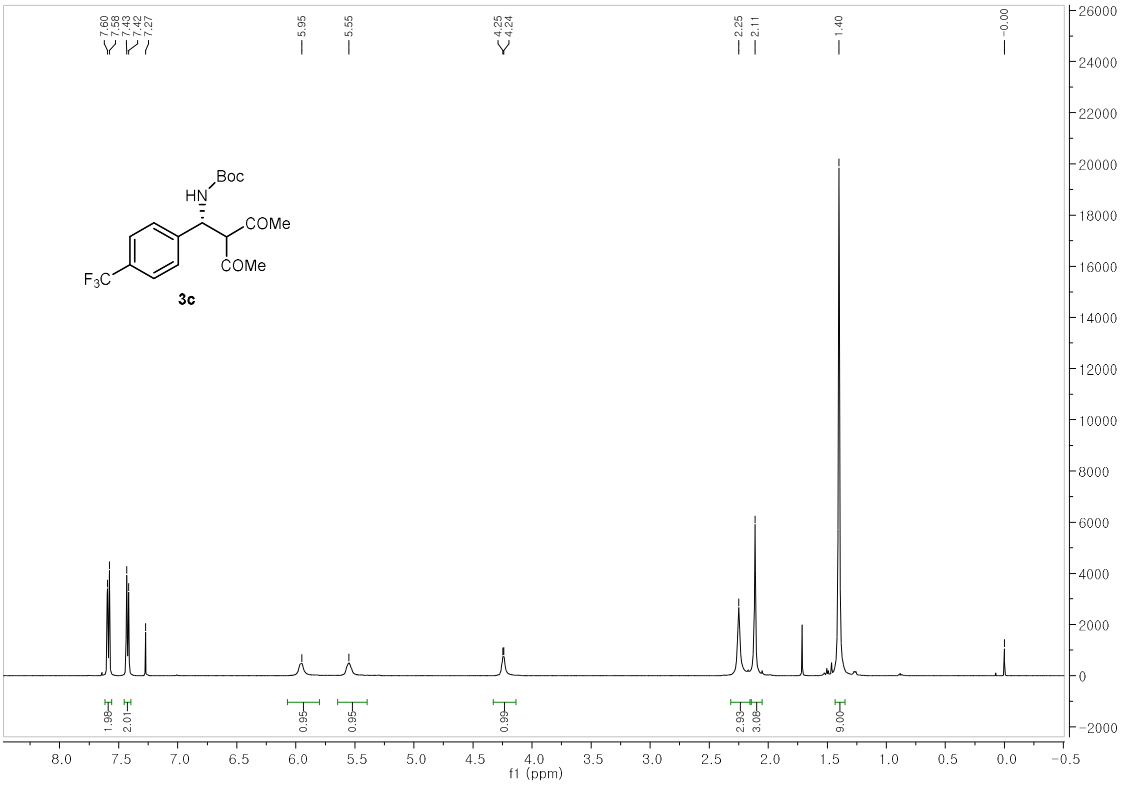

Supplementary Figure 43. ^{1}H NMR spectra of Mannich product **3c**

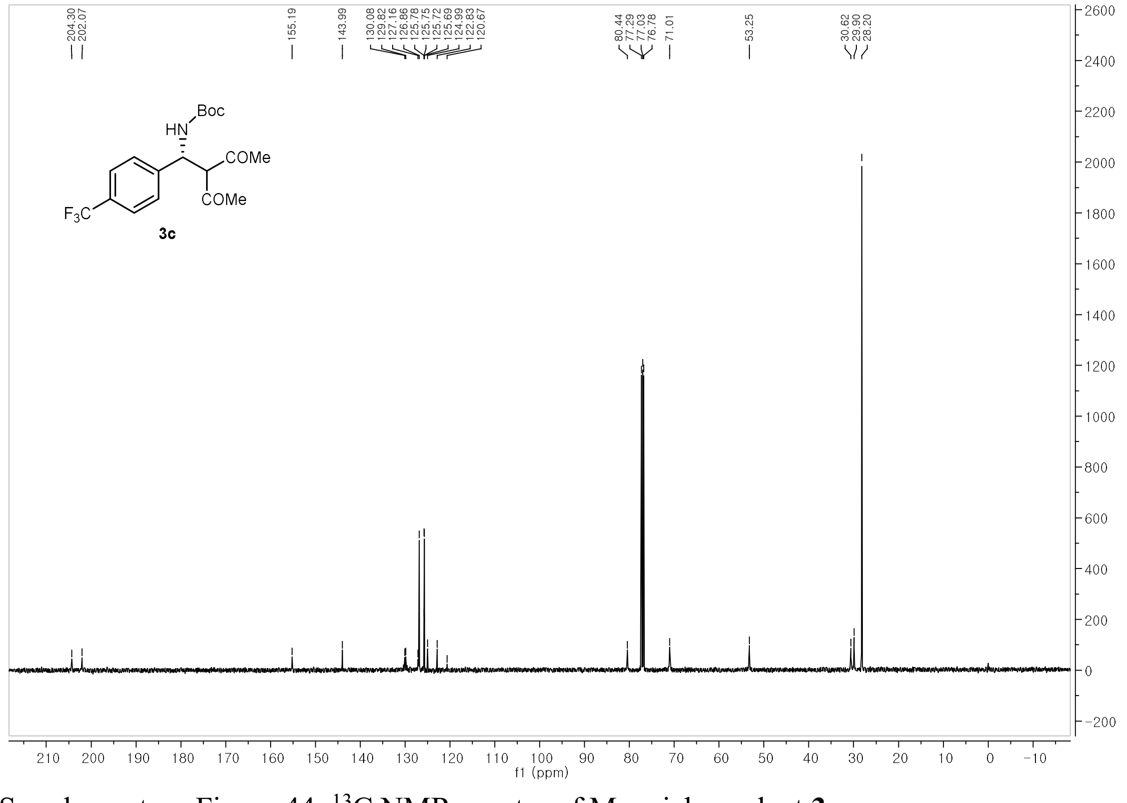

Supplementary Figure 44. ^{13}C NMR spectra of Mannich product **3c**

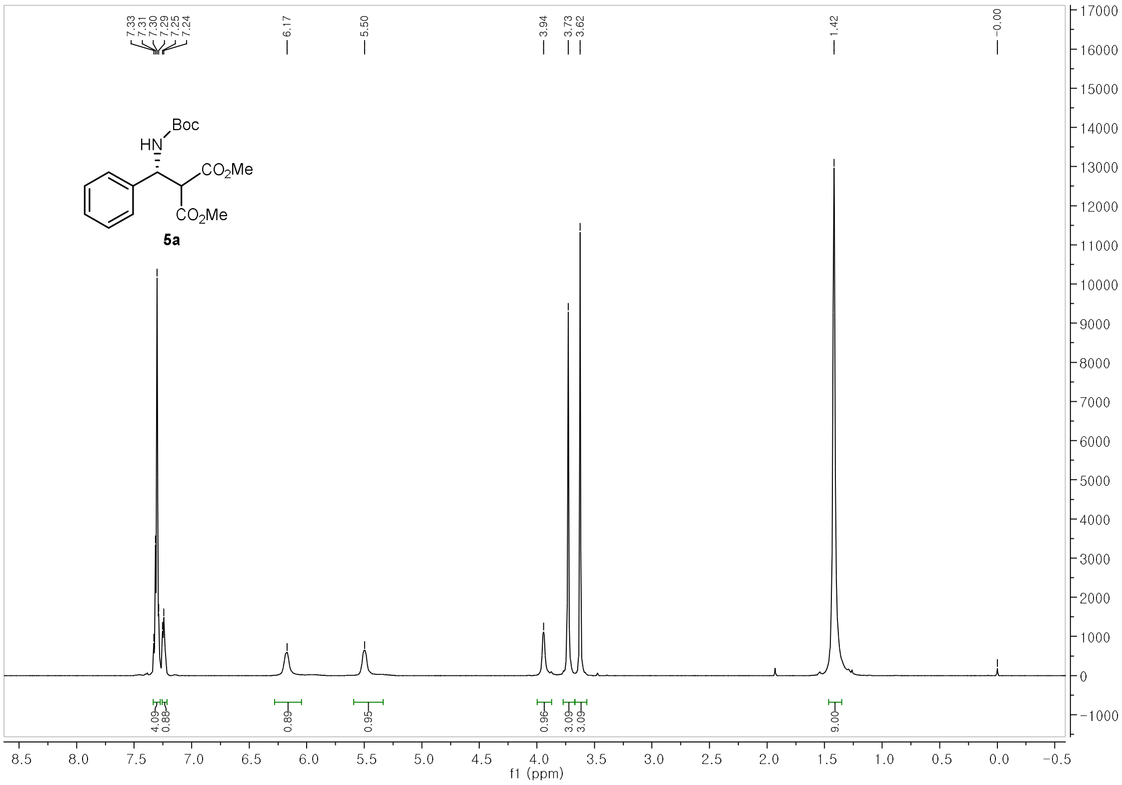

Supplementary Figure 45. ^{1}H NMR spectra of Mannich product **5a**

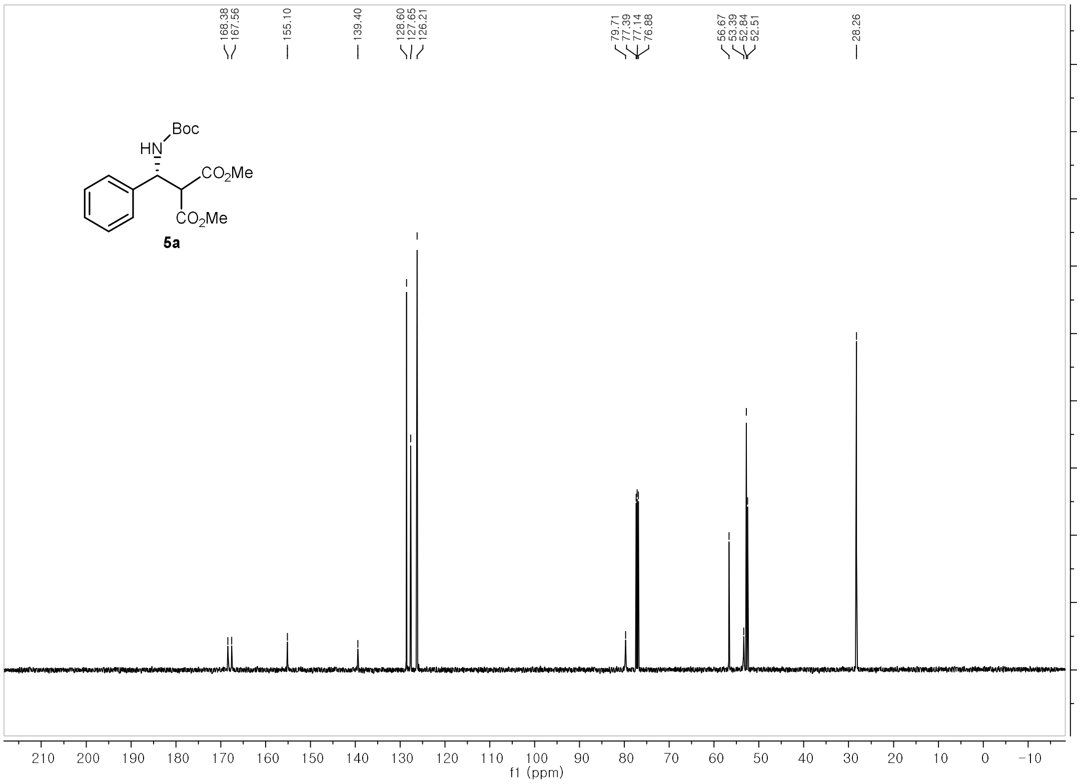

Supplementary Figure 46. ^{13}C NMR spectra of Mannich product **5a**

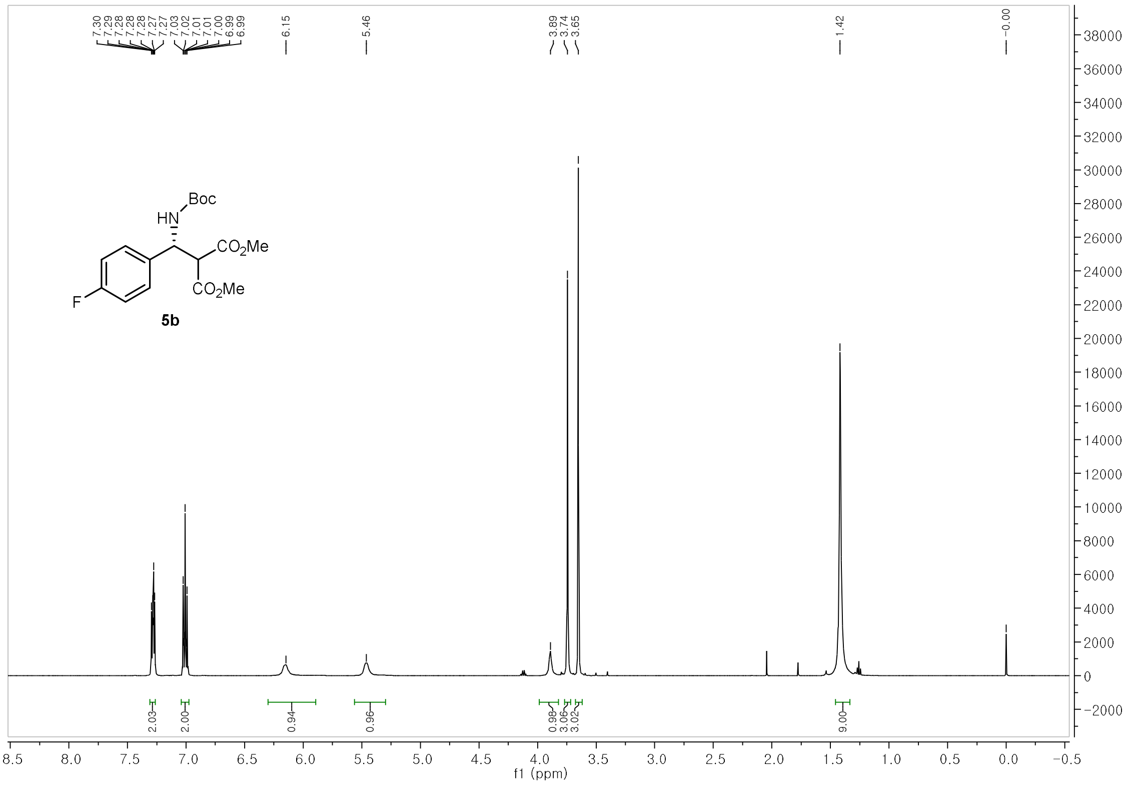

Supplementary Figure 47. ^{1}H NMR spectra of Mannich product **5b**

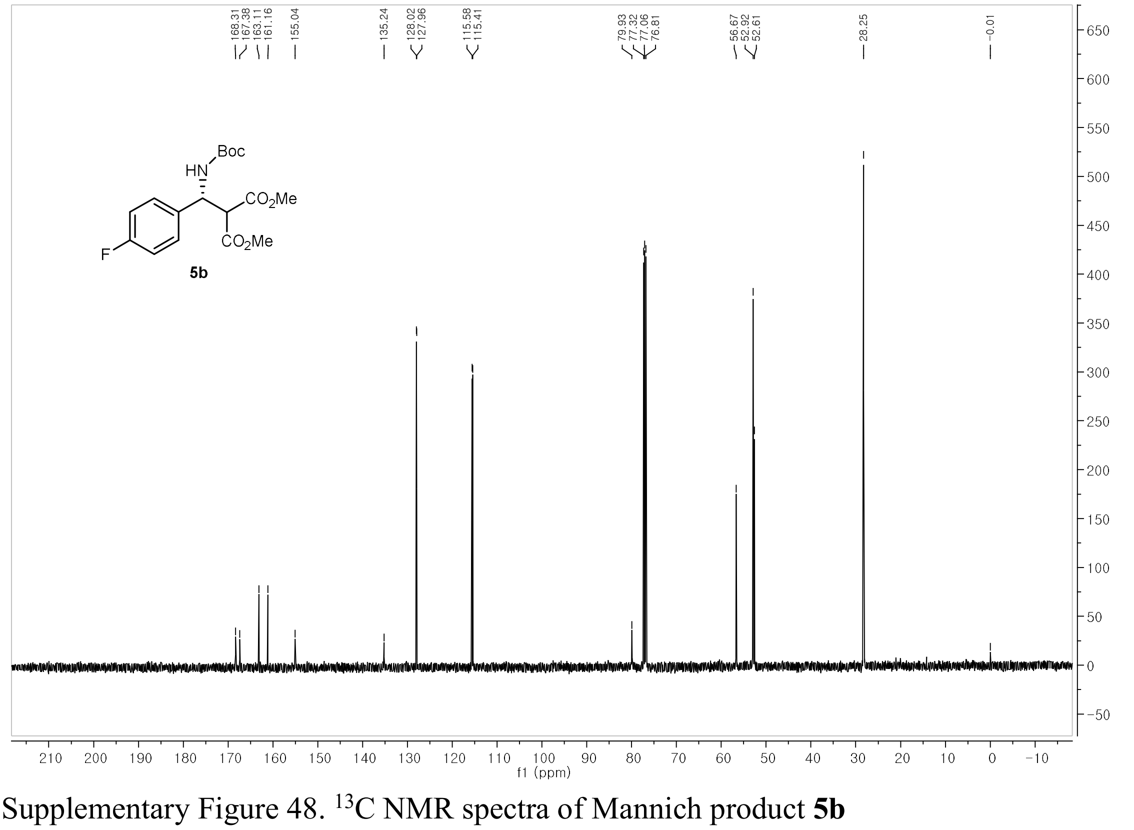

Supplementary Figure 48. ^{13}C NMR spectra of Mannich product **5b**

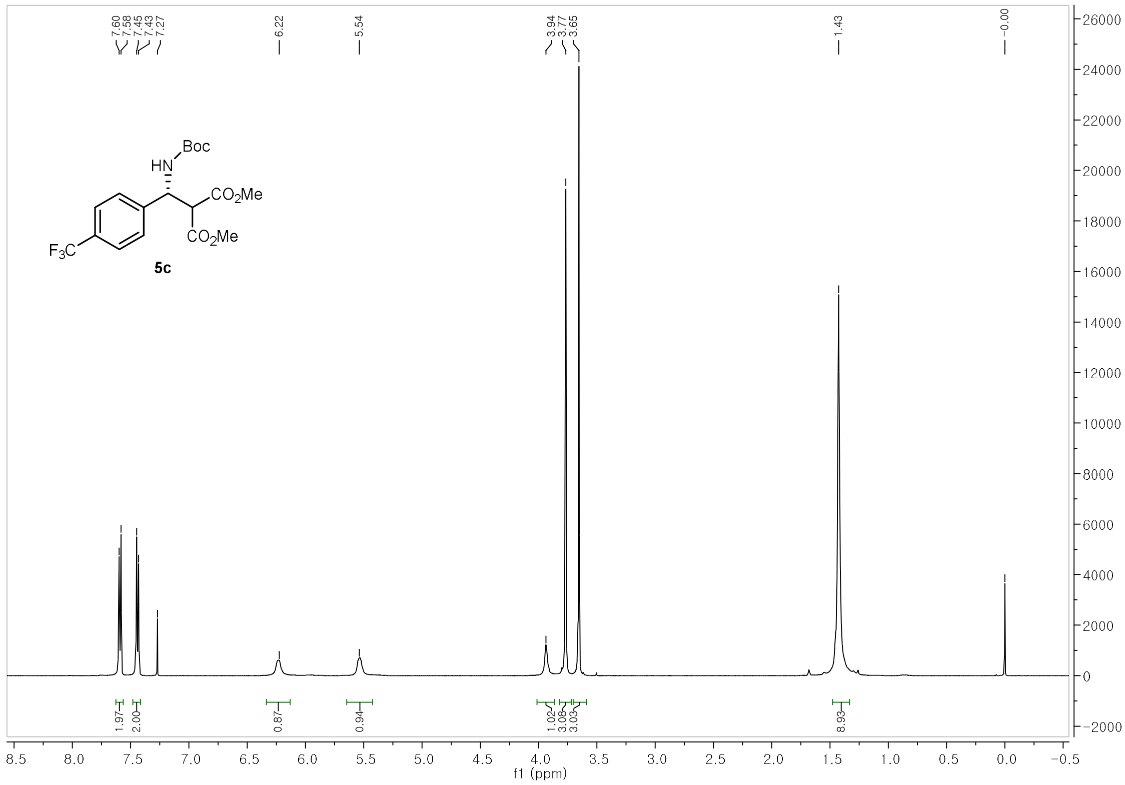

Supplementary Figure 49. ^{1}H NMR spectra of Mannich product **5c**

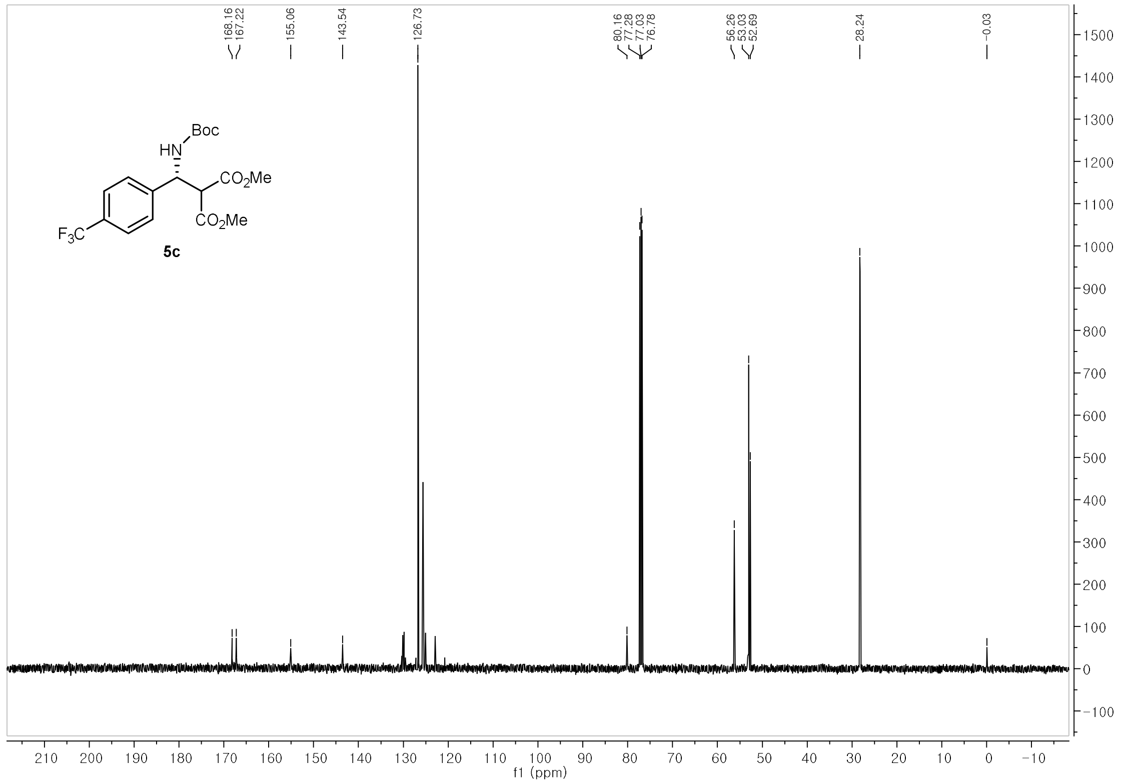

Supplementary Figure 50. ^{13}C NMR spectra of Mannich product **5c**

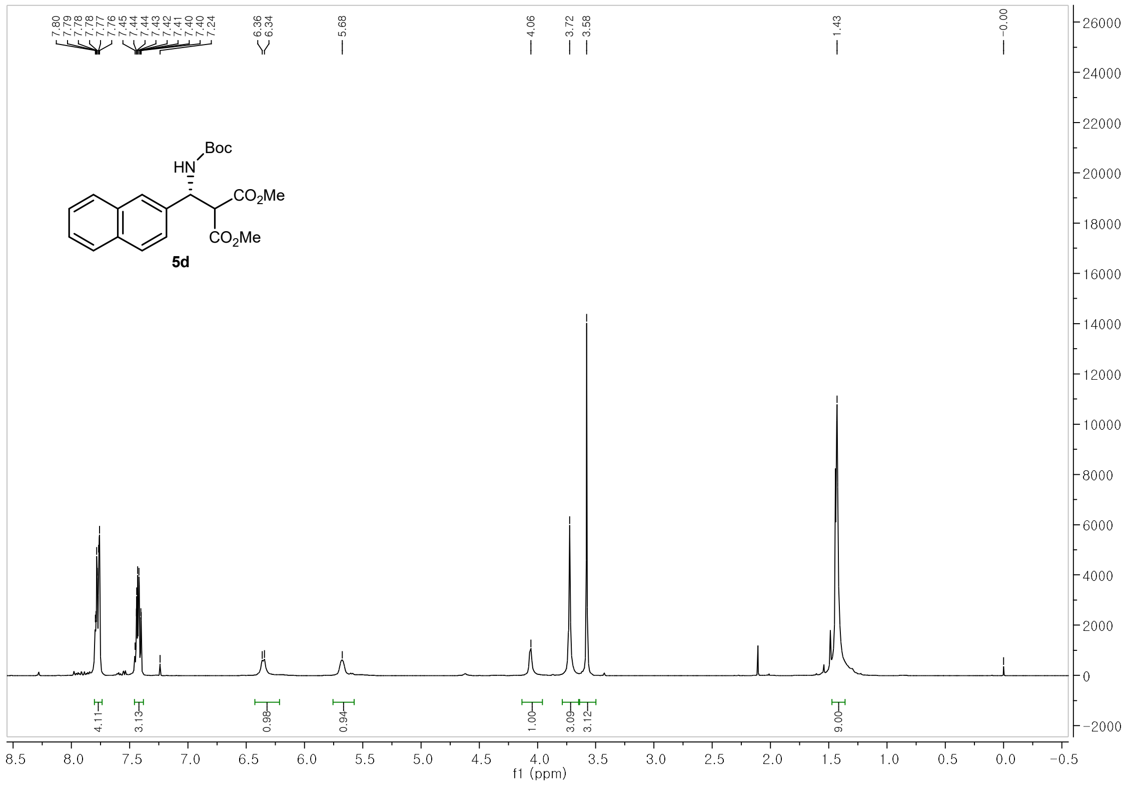

Supplementary Figure 51. ^{1}H NMR spectra of Mannich product **5d**

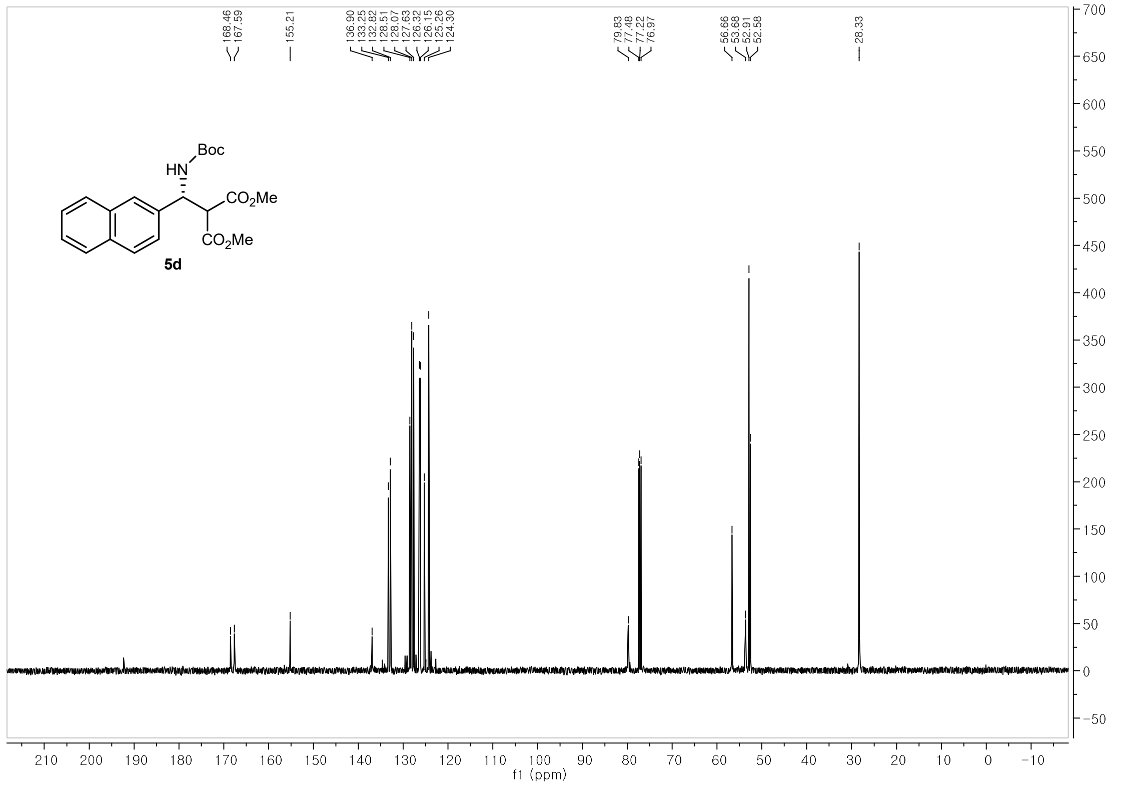

Supplementary Figure 52. ^{13}C NMR spectra of Mannich product **5d**

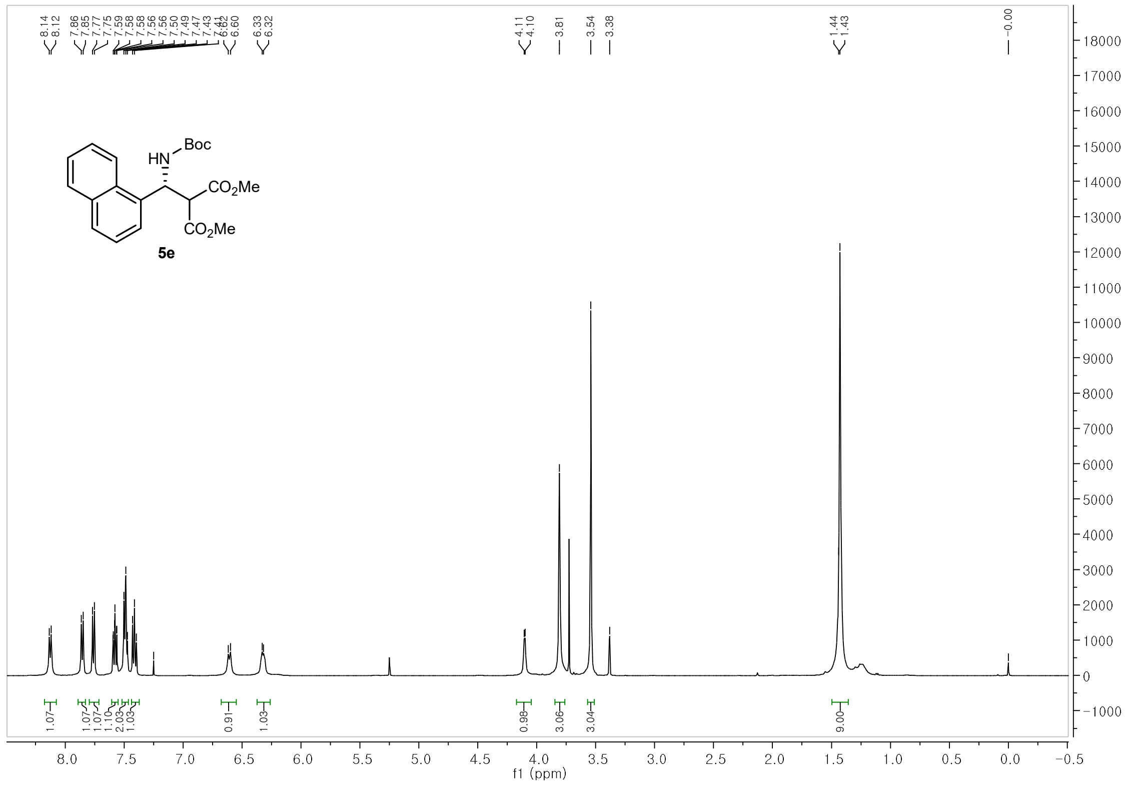

Supplementary Figure 53. ^{1}H NMR spectra of Mannich product **5e**

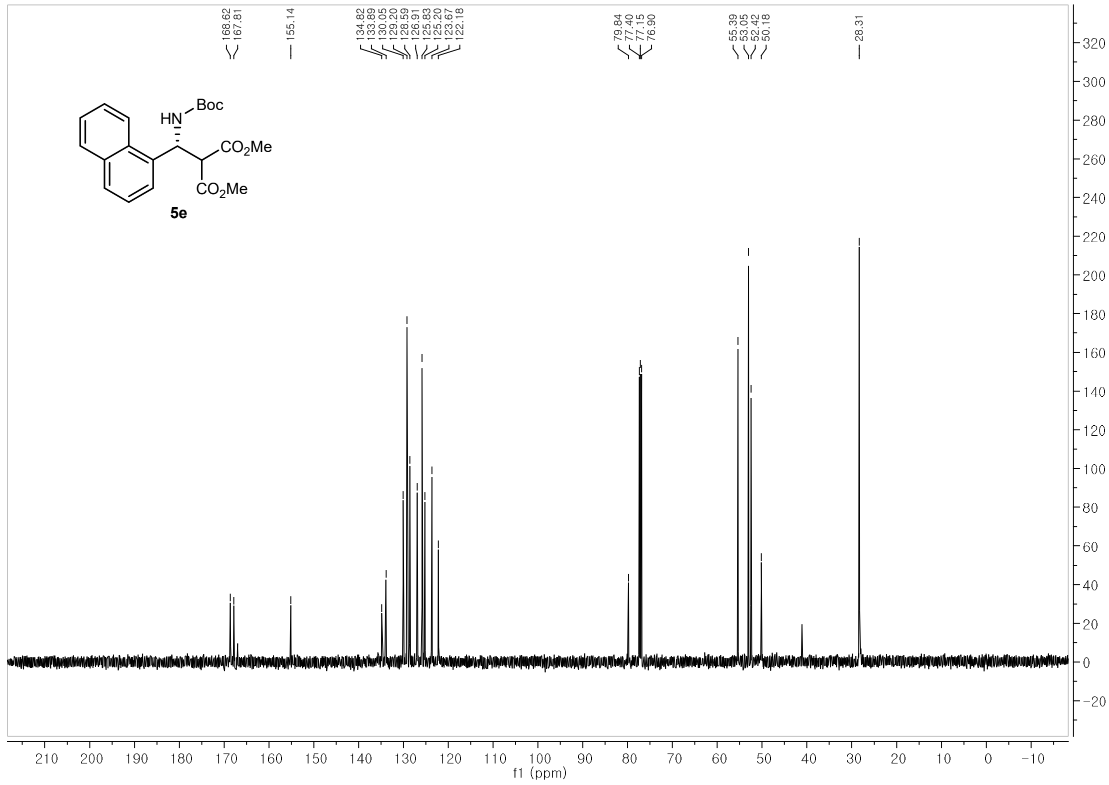

Supplementary Figure 54. ^{13}C NMR spectra of Mannich product **5e**

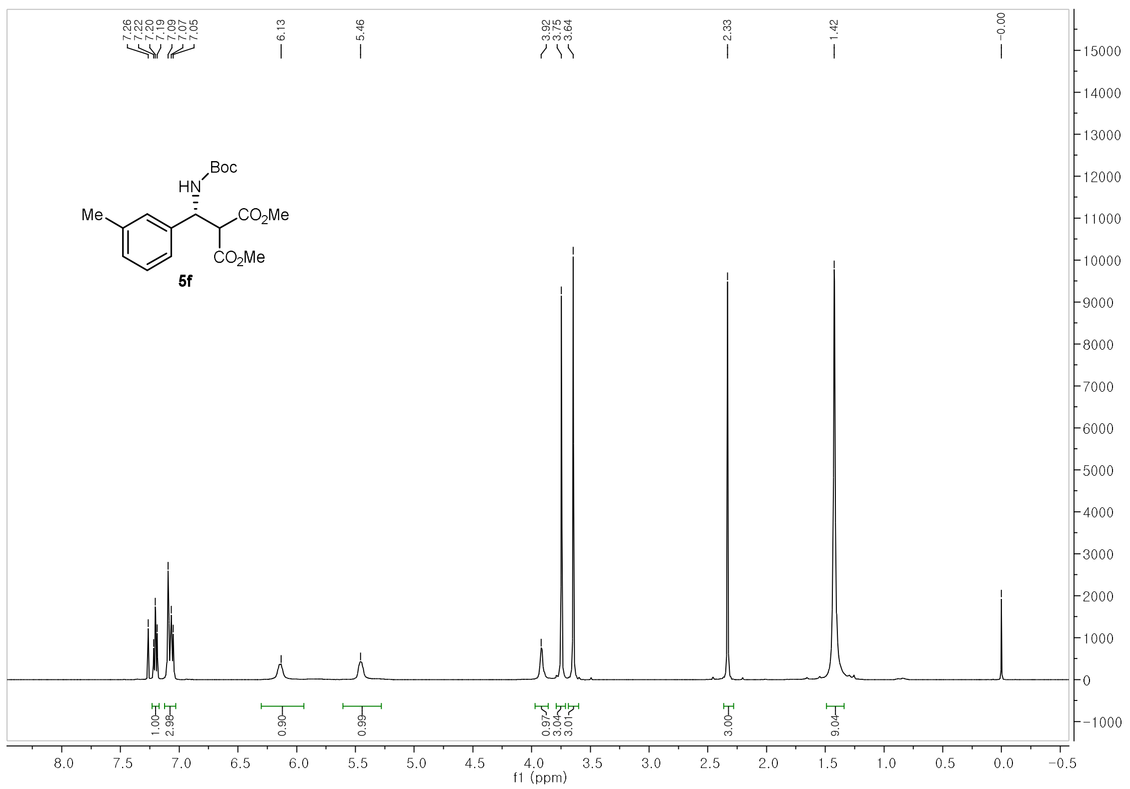

Supplementary Figure 55. ^{1}H NMR spectra of Mannich product **5f**

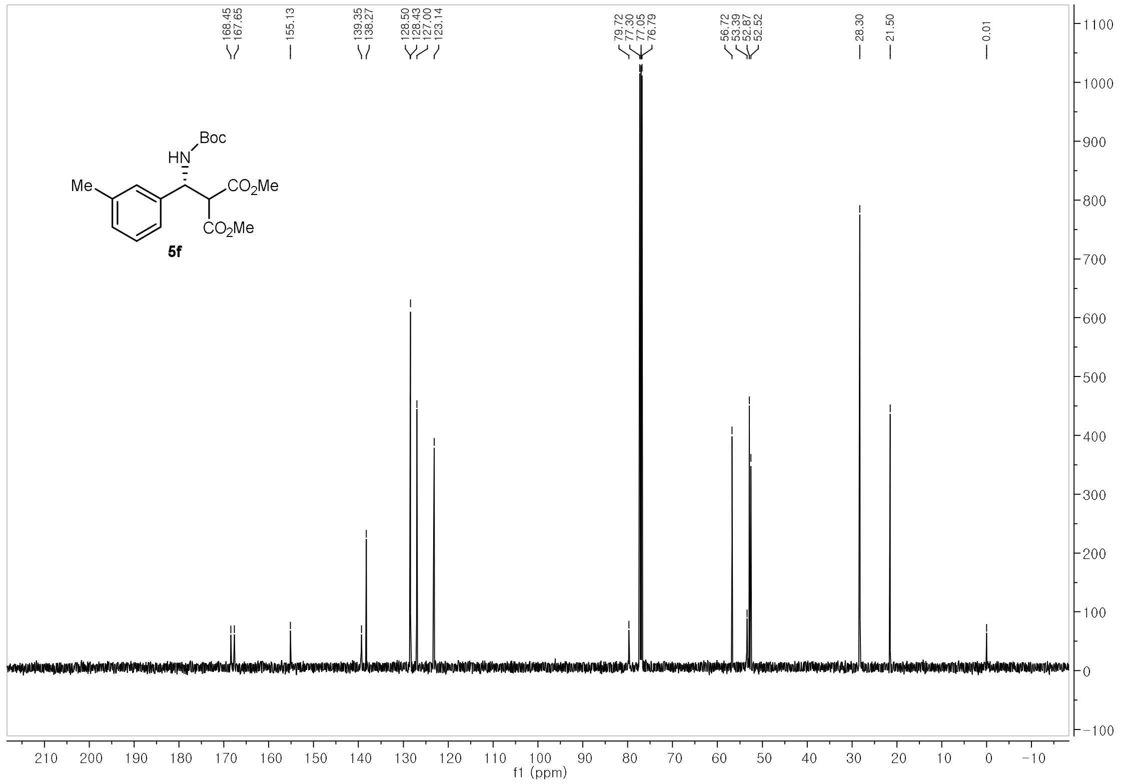

Supplementary Figure 56. ^{13}C NMR spectra of Mannich product **5f**

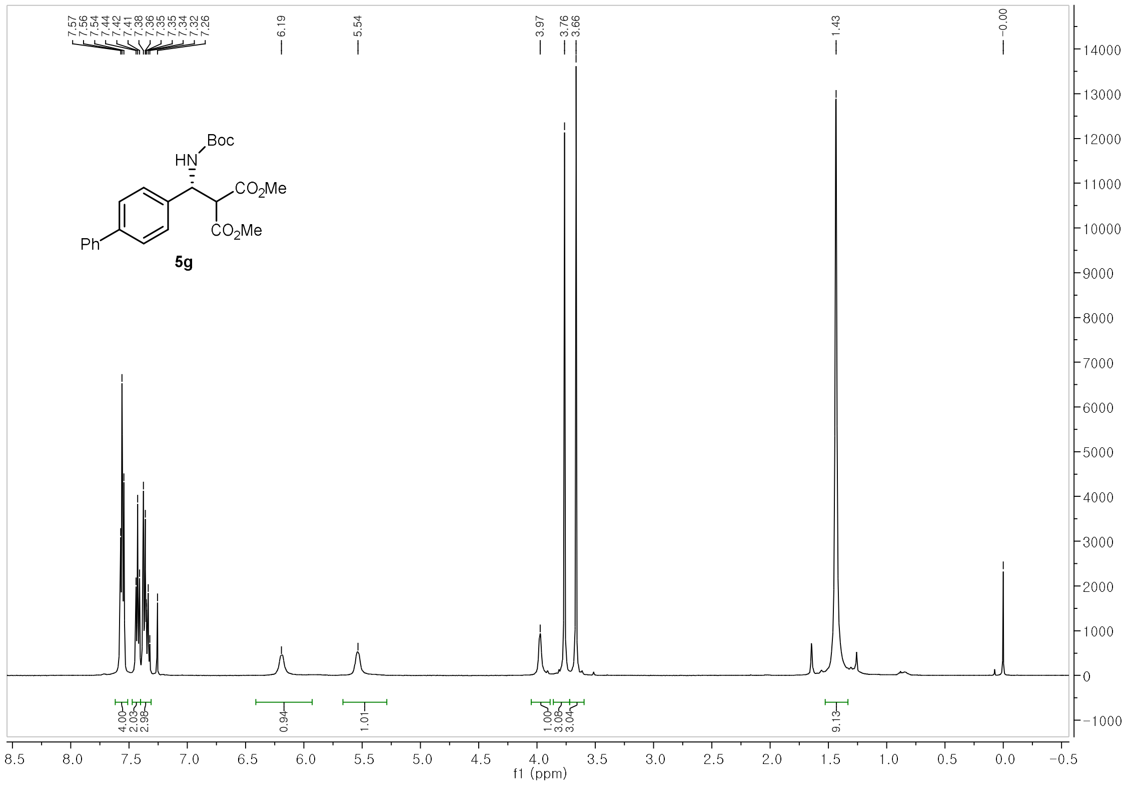

Supplementary Figure 57. ^{1}H NMR spectra of Mannich product **5g**

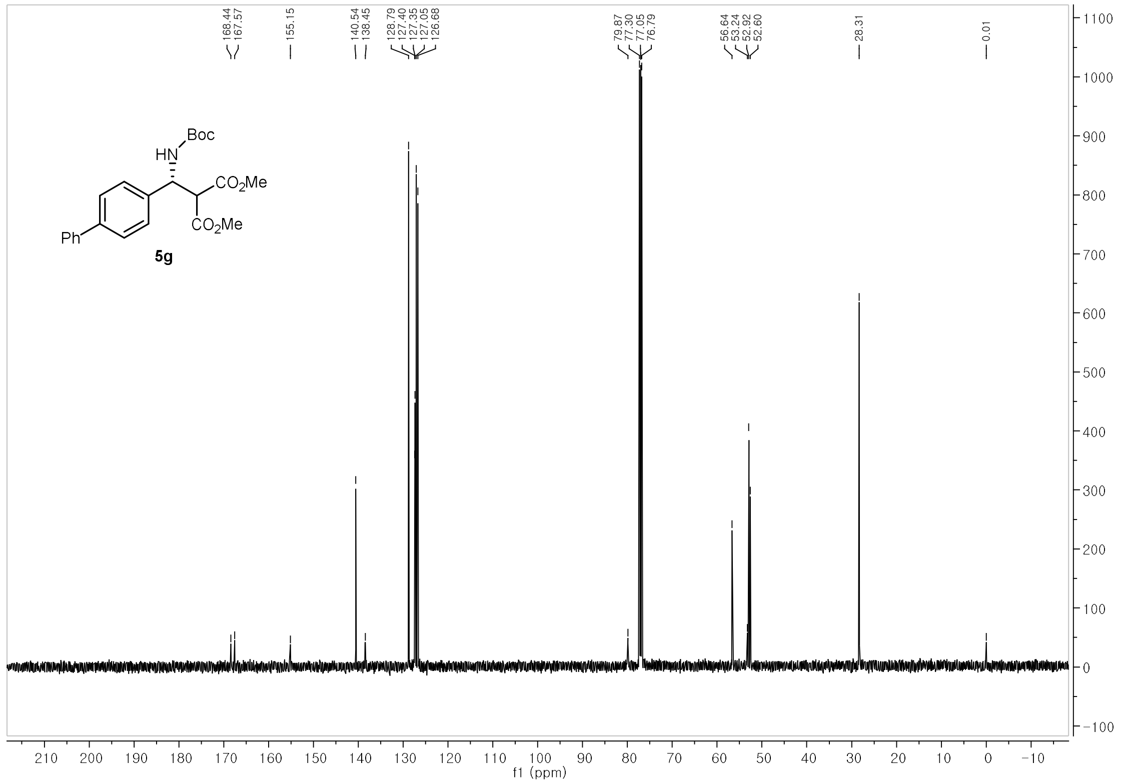

Supplementary Figure 58. ^{13}C NMR spectra of Mannich product **5g**

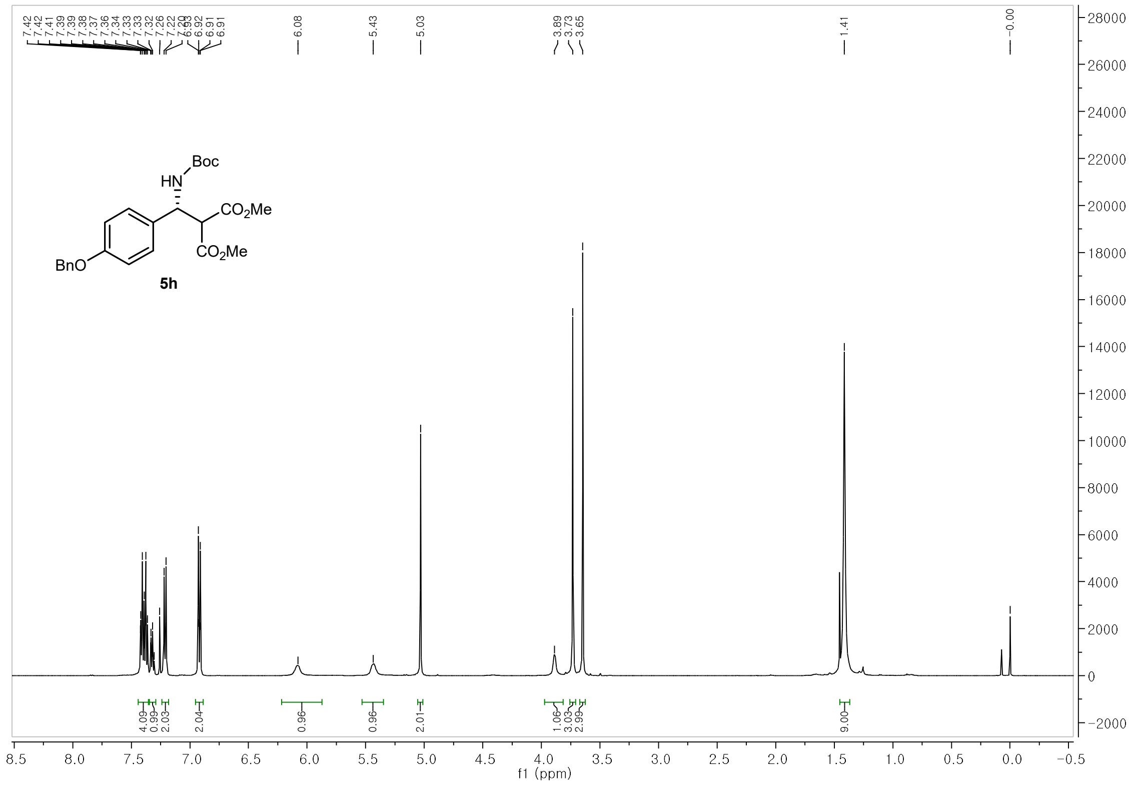

Supplementary Figure 59. ^{1}H NMR spectra of Mannich product **5h**

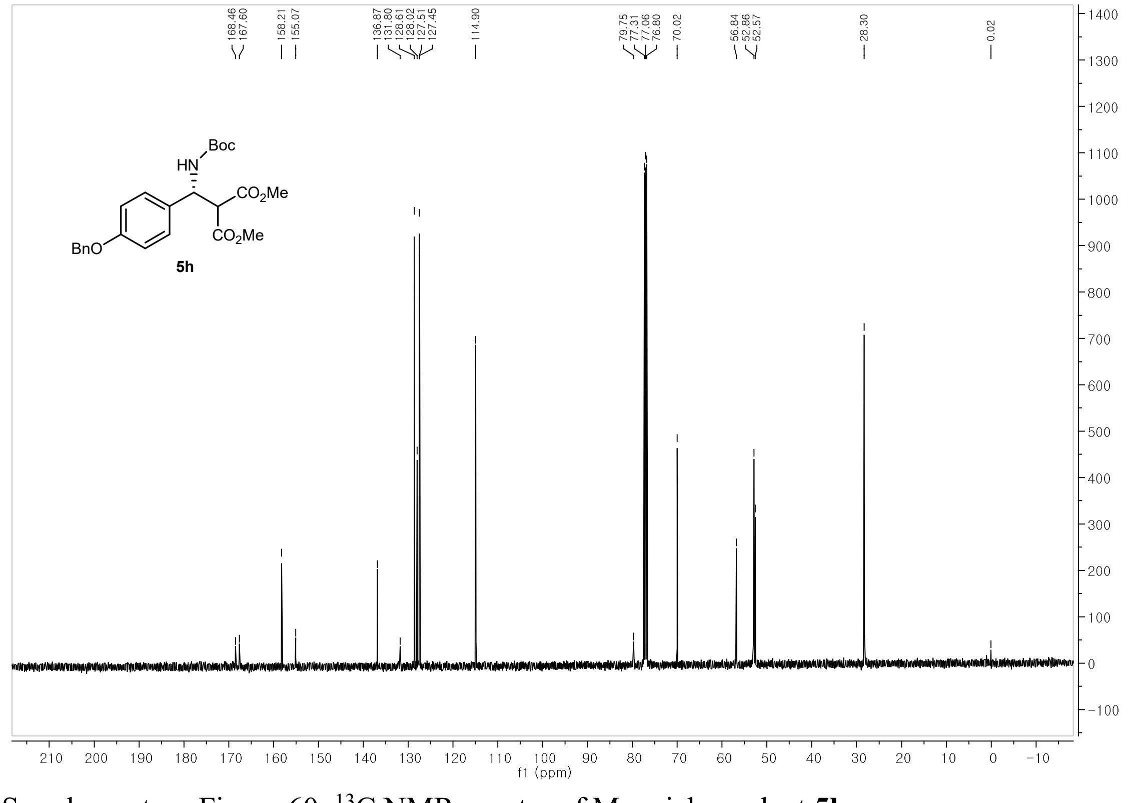

Supplementary Figure 60. ^{13}C NMR spectra of Mannich product **5h**

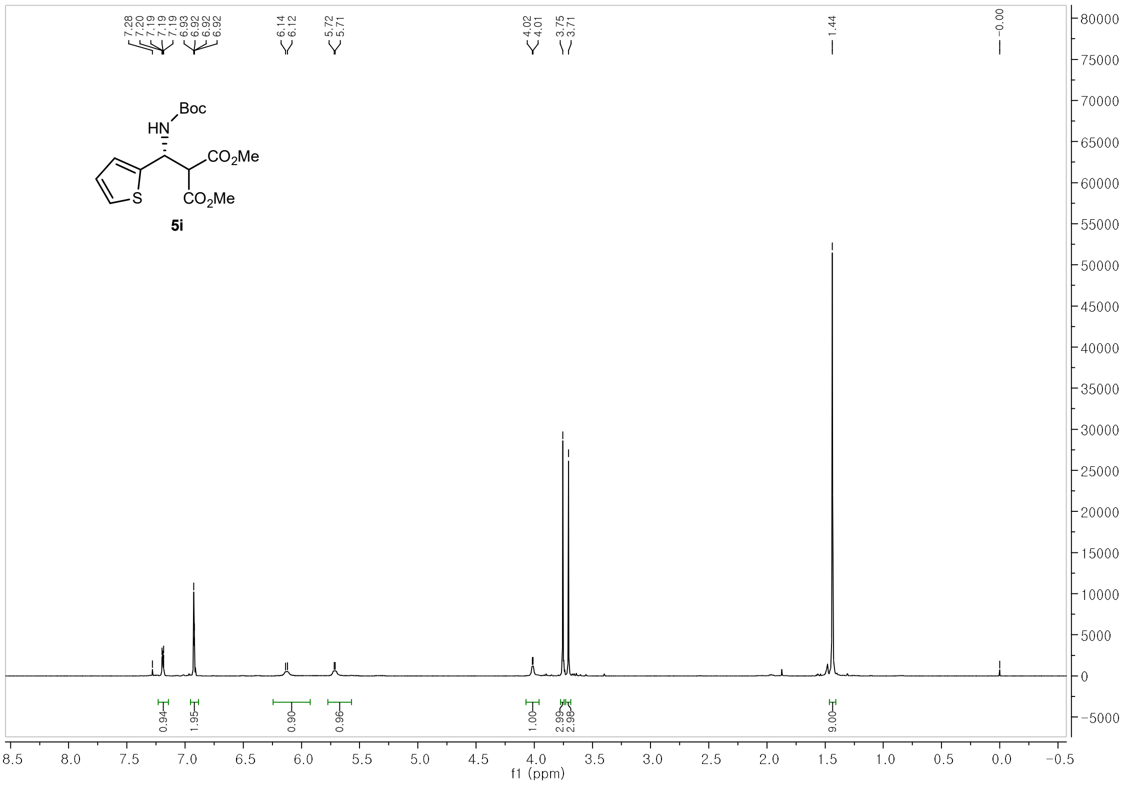

Supplementary Figure 61. ^{1}H NMR spectra of Mannich product **5i**

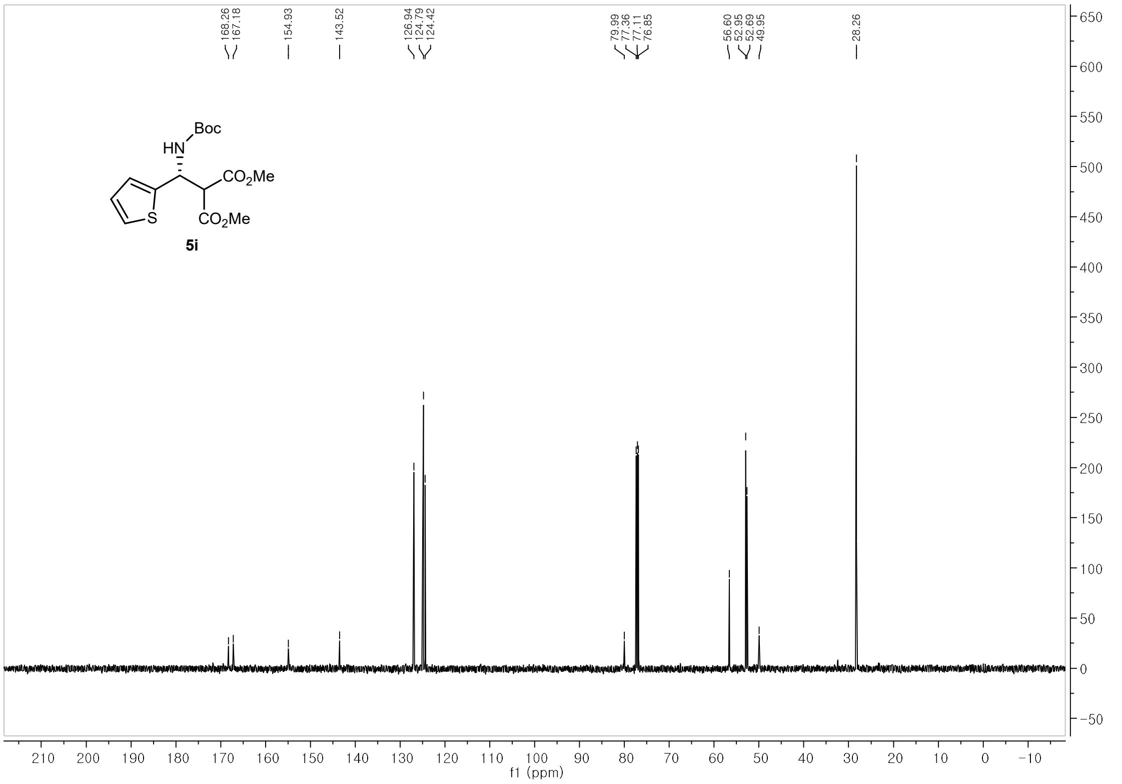

Supplementary Figure 62. ^{13}C NMR spectra of Mannich product **5i**

NMR Spectra of Supplementary Table 4

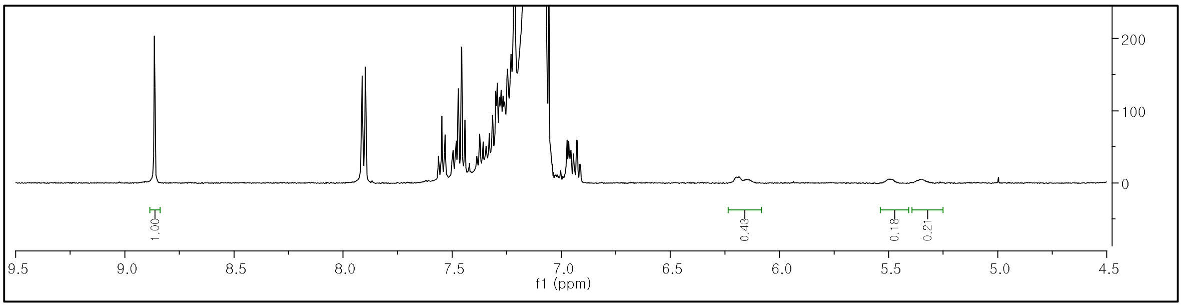

Supplementary Figure 63. ^{1}H NMR spectra of Kinetic study (12 hr, Size of droplet = 40 nL)

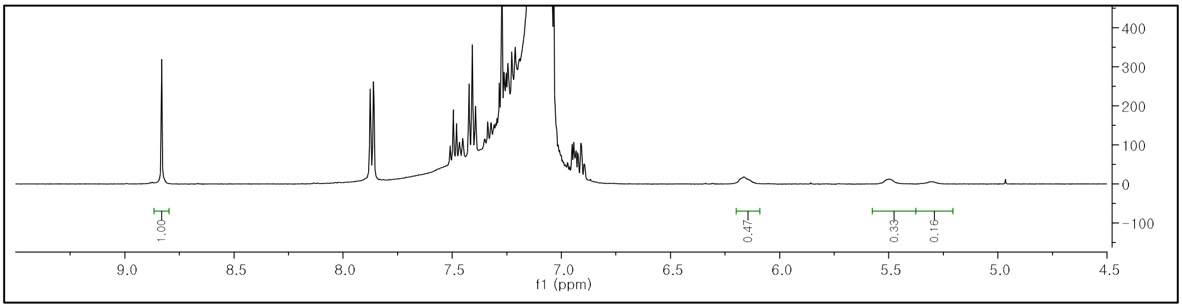

Supplementary Figure 64. ^{1}H NMR spectra of Kinetic study (12 hr, Size of droplet = 111 nL)

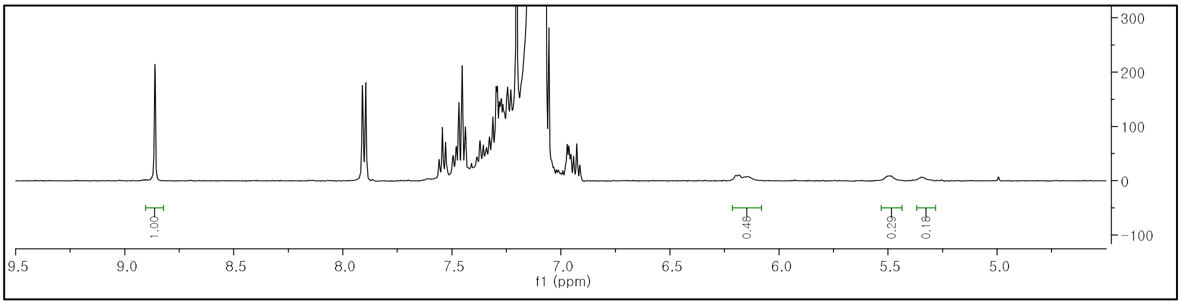

Supplementary Figure 65. ^{1}H NMR spectra of Kinetic study (12 hr, Size of droplet = 600 nL)

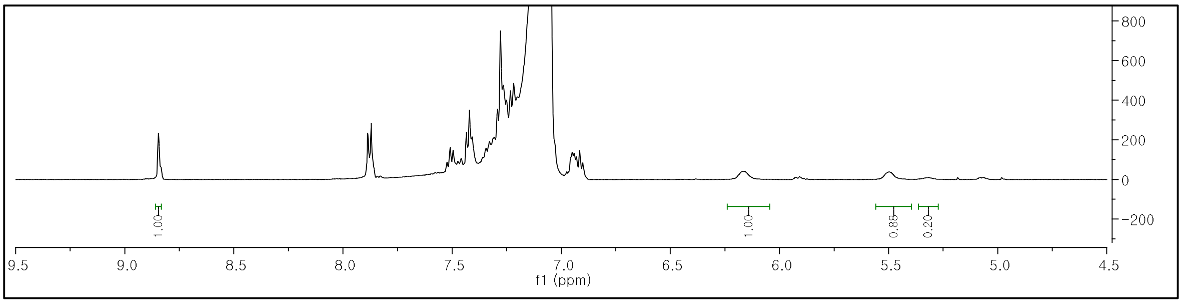

Supplementary Figure 66. ^{1}H NMR spectra of Kinetic study (24 hr, Size of droplet = 40 nL)

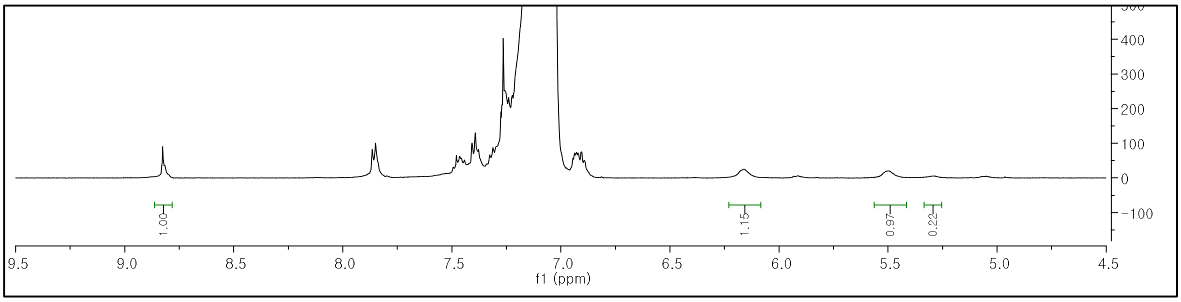

Supplementary Figure 67. ^{1}H NMR spectra of Kinetic study (24 hr, Size of droplet = 111 nL)

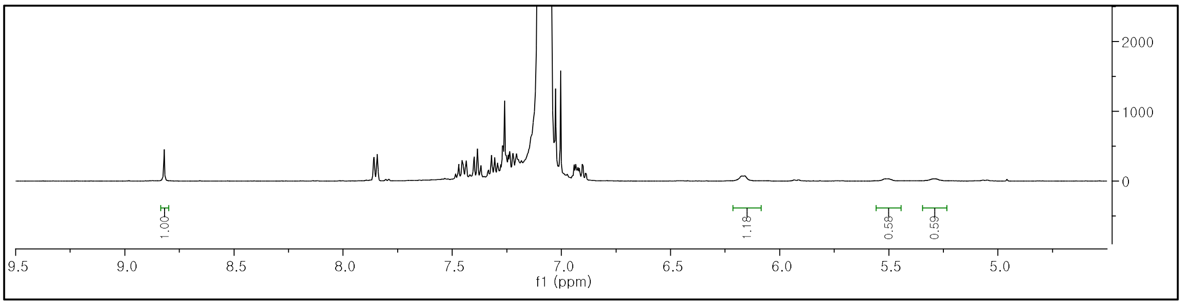

Supplementary Figure 68. ^{1}H NMR spectra of Kinetic study (24 hr, Size of droplet = 600 nL)

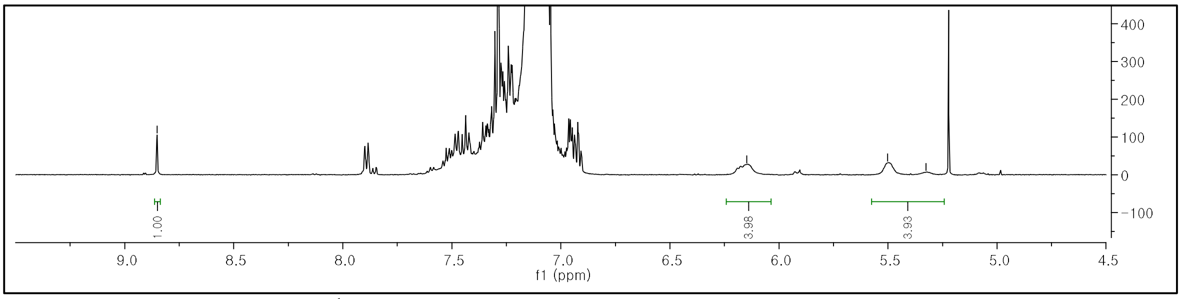

Supplementary Figure 69. ^{1}H NMR spectra of Kinetic study (48 hr, Size of droplet = 40 nL)

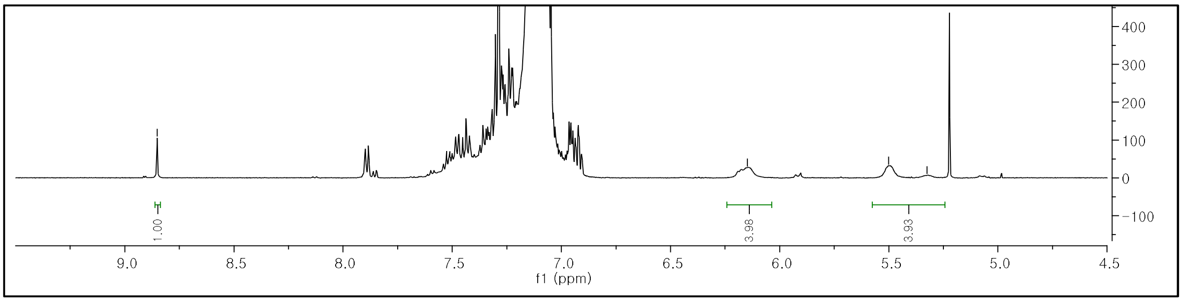

Supplementary Figure 70. ^{1}H NMR spectra of Kinetic study (48 hr, Size of droplet = 111 nL)

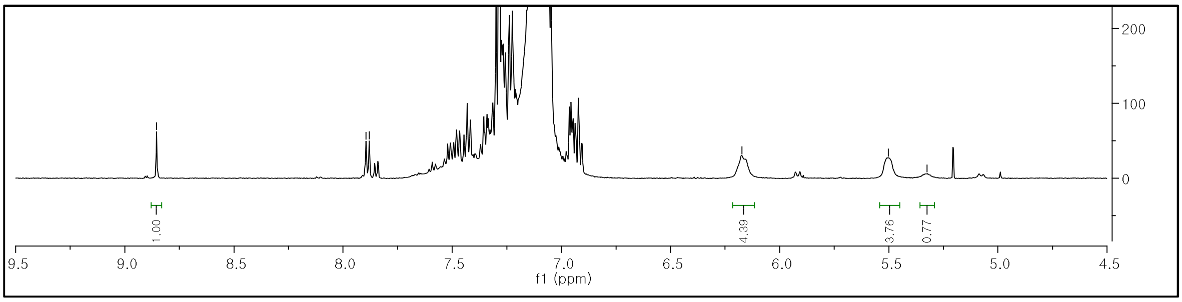

Supplementary Figure 71. ^{1}H NMR spectra of Kinetic study (48 hr, Size of droplet = 600 nL)

HPLC spectra

HPLC spectra of Fig. 1

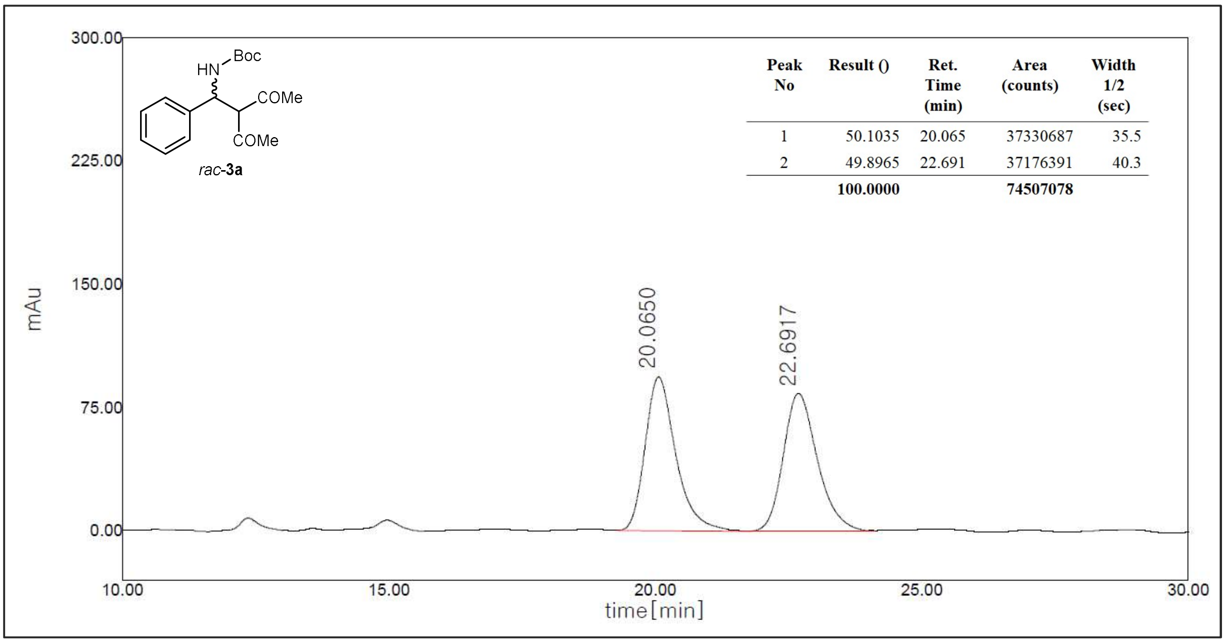

Supplementary Figure 72. HPLC spectra of *rac*-**3a**

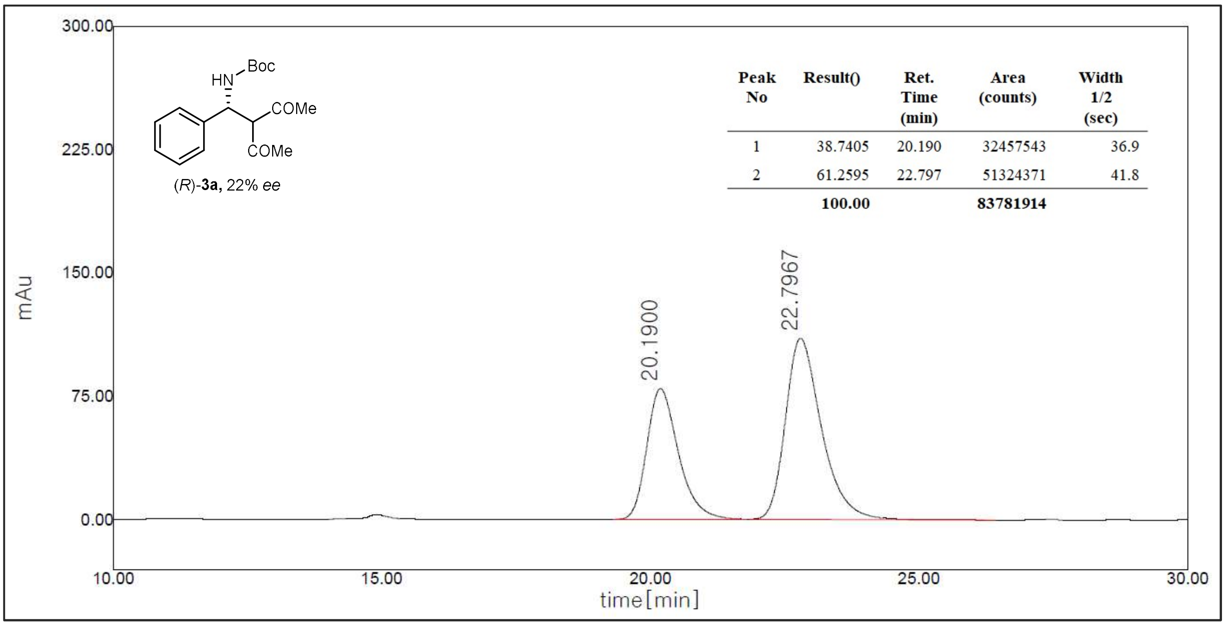

Supplementary Figure 73. HPLC spectra of **3a** (in dichloromethane)

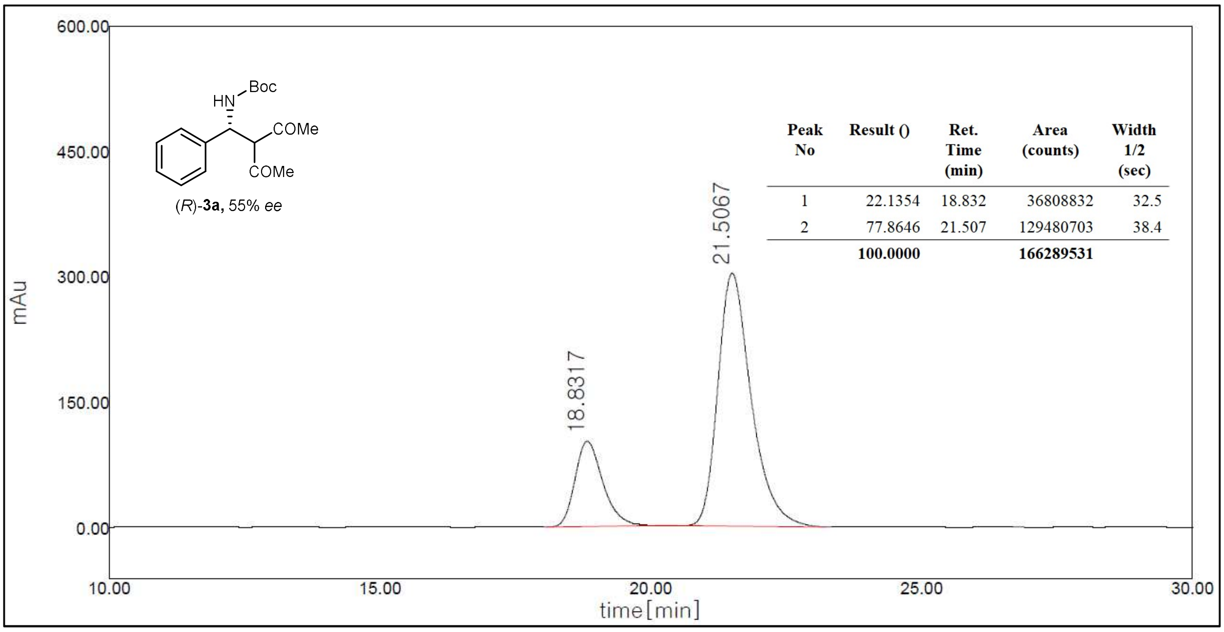

Supplementary Figure 74. HPLC spectra of **3a** (in brine)

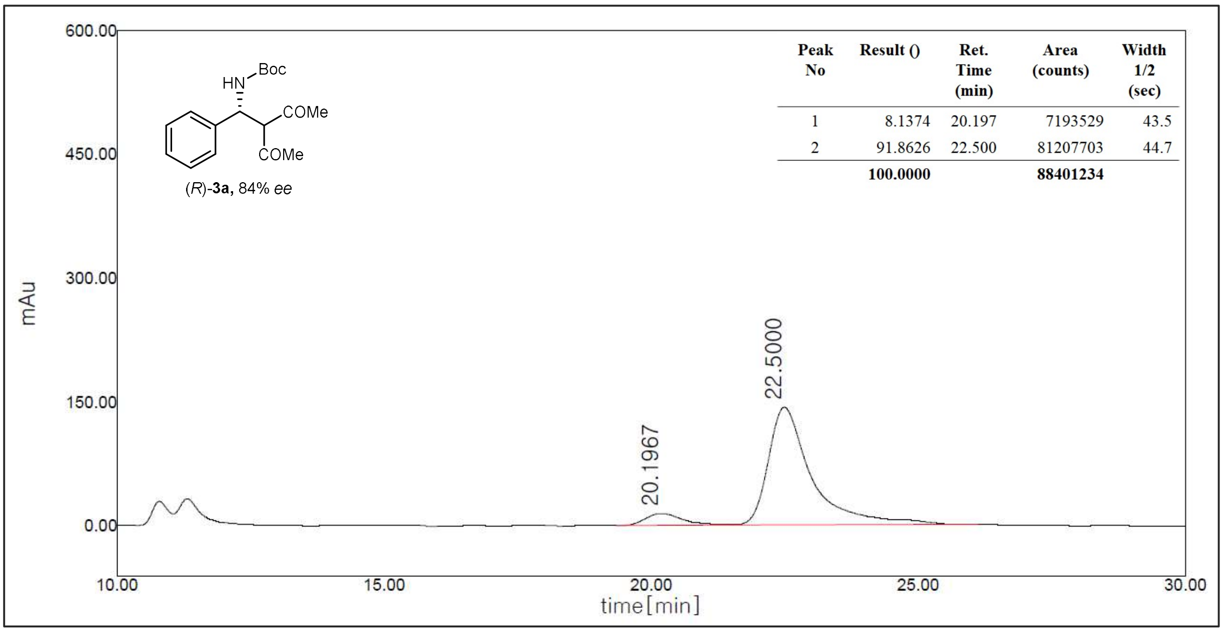

Supplementary Figure 75. HPLC spectra of **3a** (in H_2O/toluene)

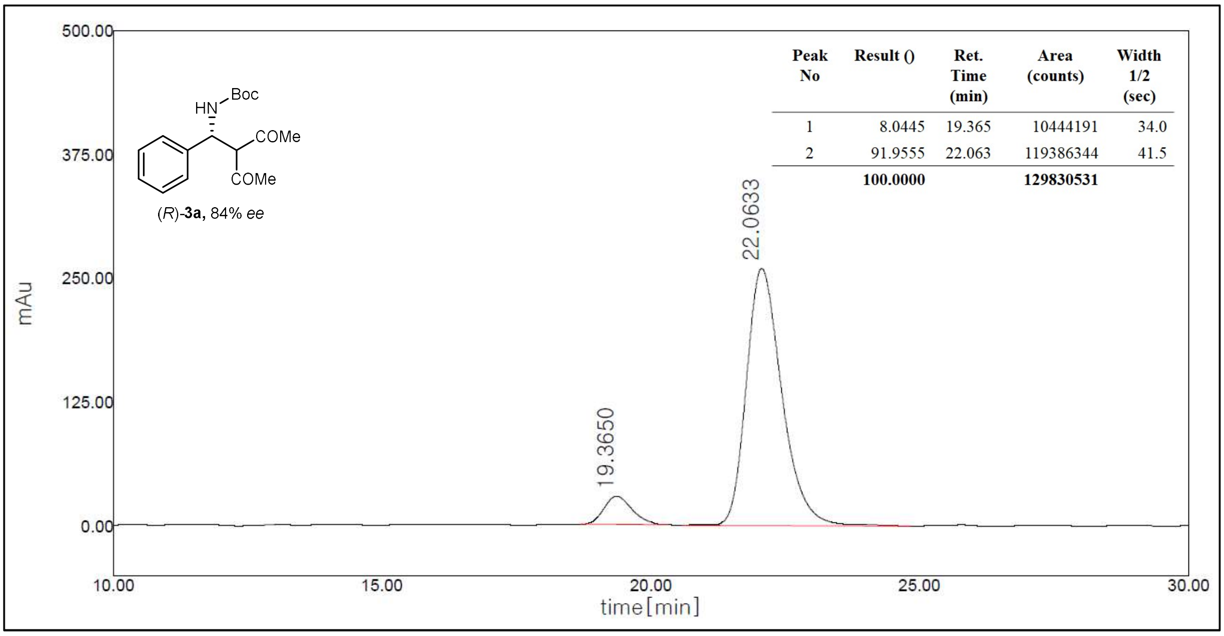

Peak No	Result ()	Ret. Time (min)	Area (counts)	Width 1/2 (sec)
1	8.0445	19.365	10444191	34.0
2	91.9555	22.063	119386344	41.5
	**100.0000**		**129830531**	

Supplementary Figure 76. HPLC spectra of **3a** (in brine/toluene)

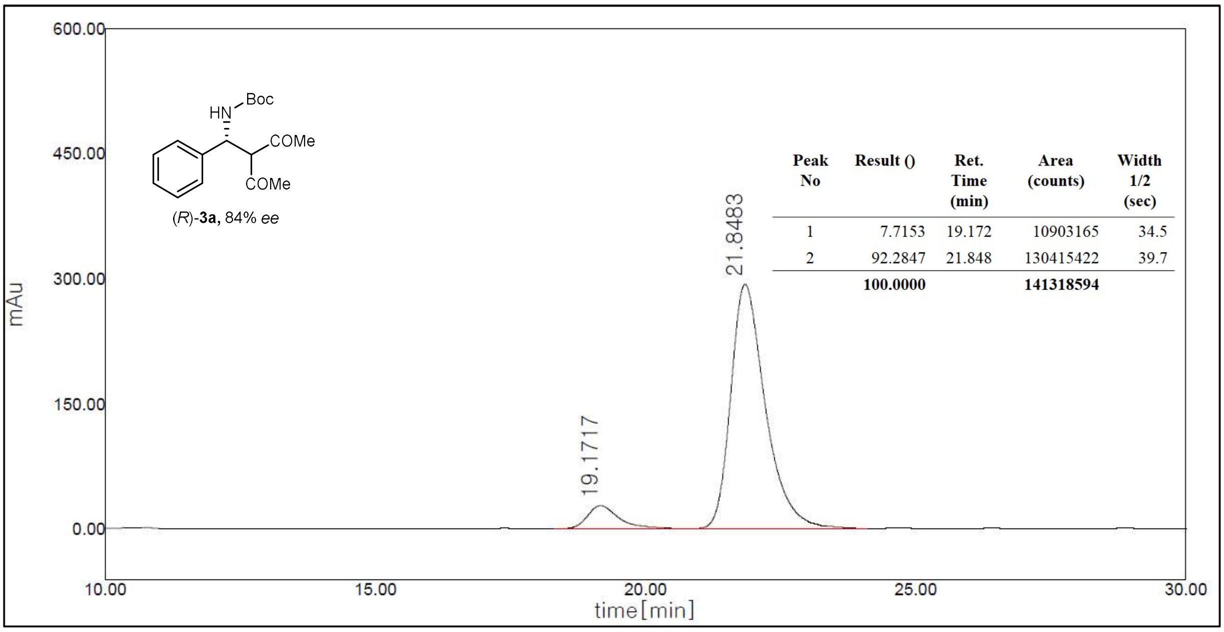

Peak No	Result ()	Ret. Time (min)	Area (counts)	Width 1/2 (sec)
1	7.7153	19.172	10903165	34.5
2	92.2847	21.848	130415422	39.7
	**100.0000**		**141318594**	

Supplementary Figure 77. HPLC spectra of **3a** (in brine/*o*-xylene)

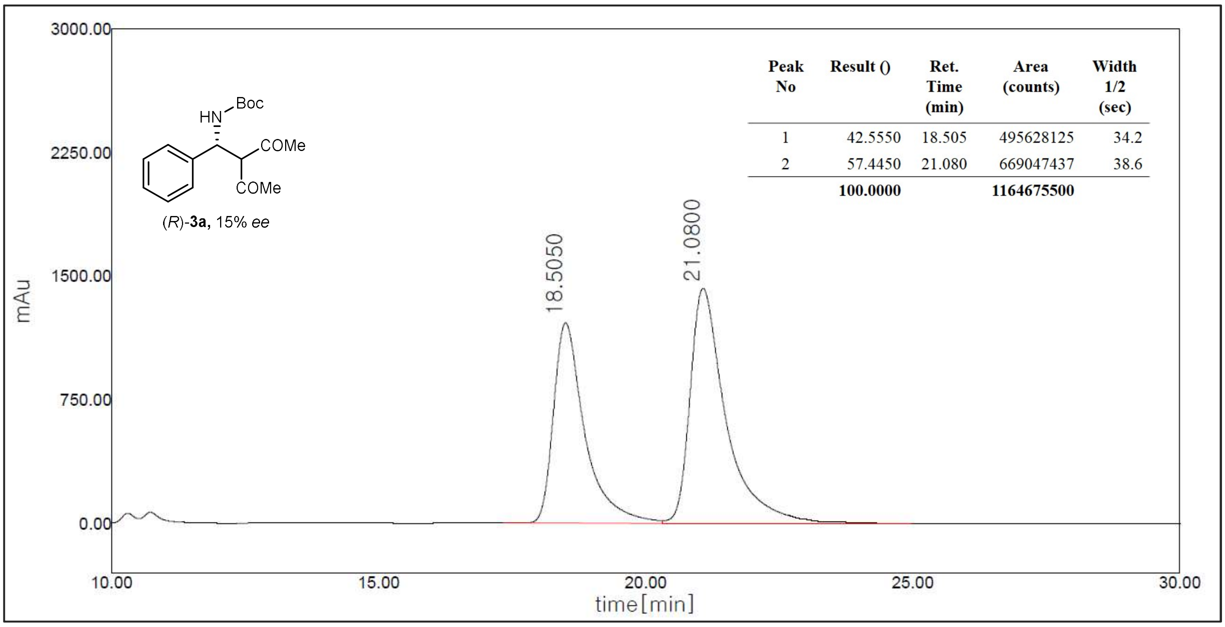

Supplementary Figure 78. HPLC spectra of **3a** (in aq. $LiClO_4$/*o*-xylene)

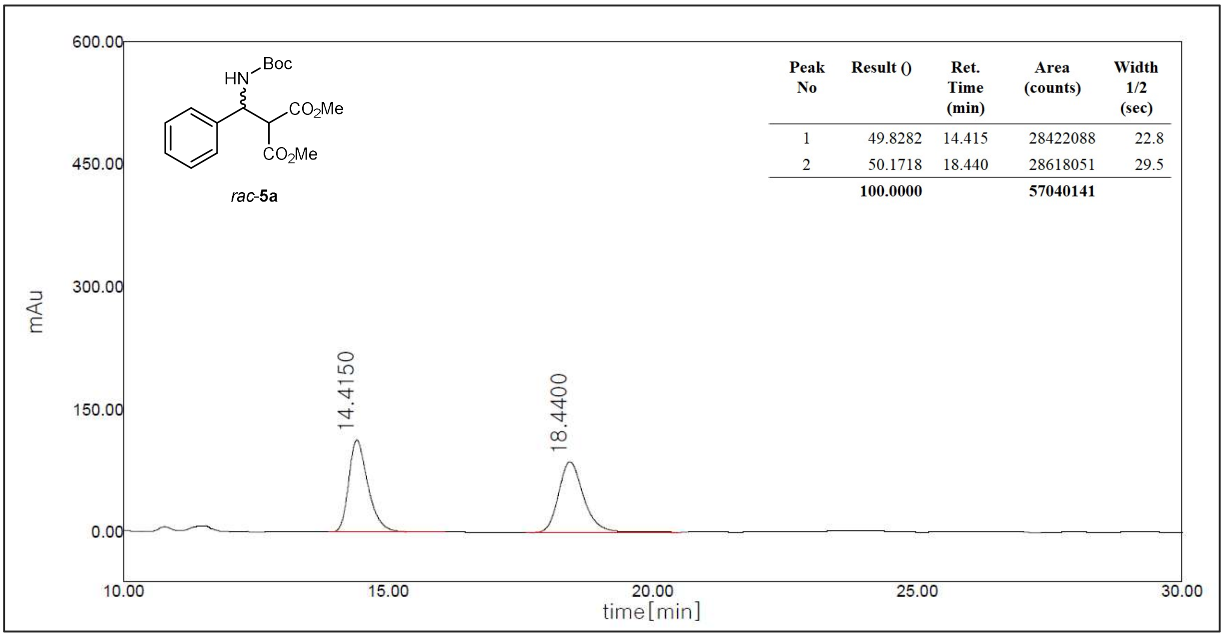

Peak No	Result ()	Ret. Time (min)	Area (counts)	Width 1/2 (sec)
1	49.8282	14.415	28422088	22.8
2	50.1718	18.440	28618051	29.5
	**100.0000**		**57040141**	

Supplementary Figure 79. HPLC spectra of *rac*-**5a**

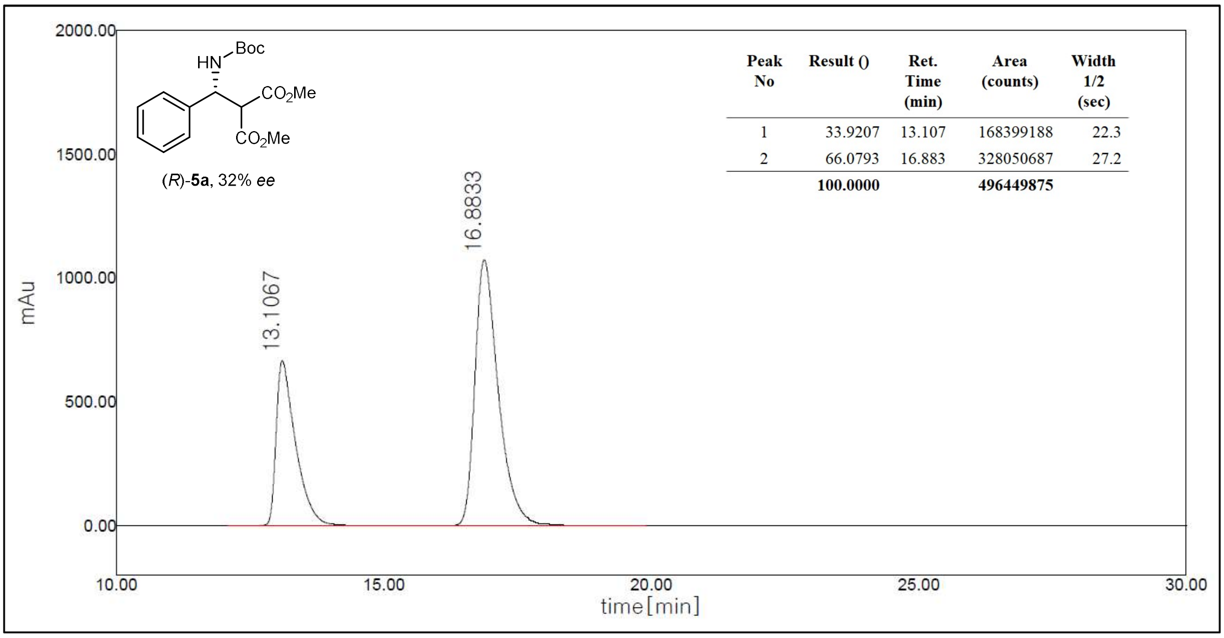

Peak No	Result ()	Ret. Time (min)	Area (counts)	Width 1/2 (sec)
1	33.9207	13.107	168399188	22.3
2	66.0793	16.883	328050687	27.2
	**100.0000**		**496449875**	

Supplementary Figure 80. HPLC spectra of **5a** (in dichloromethane)

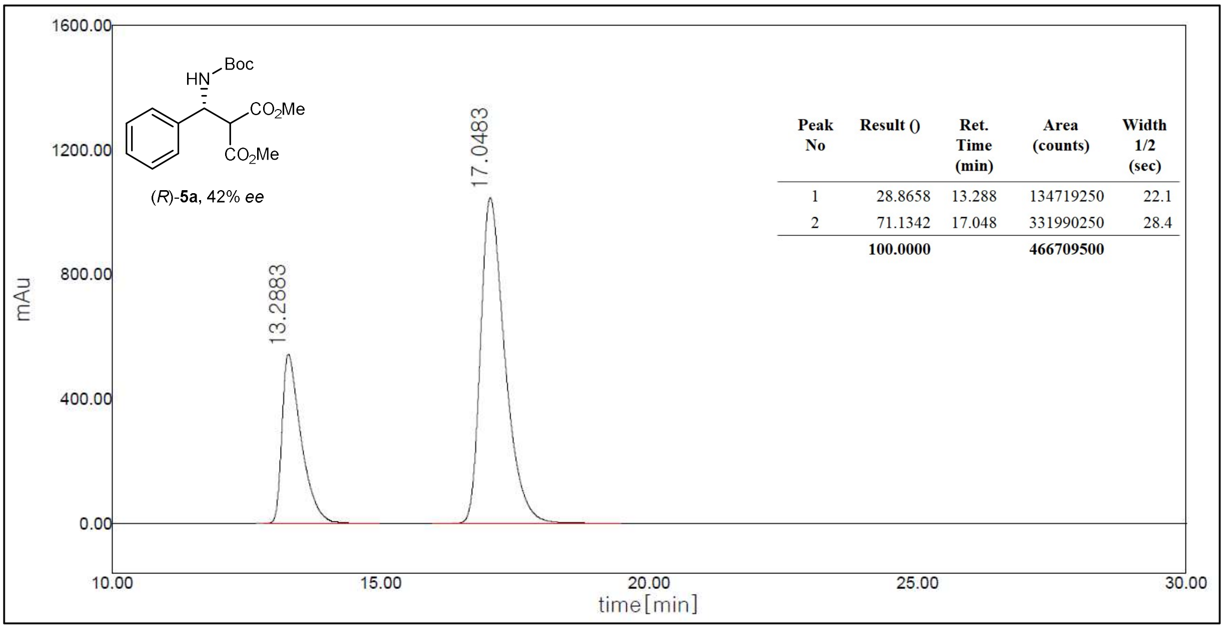

Peak No	Result ()	Ret. Time (min)	Area (counts)	Width 1/2 (sec)
1	28.8658	13.288	134719250	22.1
2	71.1342	17.048	331990250	28.4
	**100.0000**		**466709500**	

Supplementary Figure 81. HPLC spectra of **5a** (in brine)

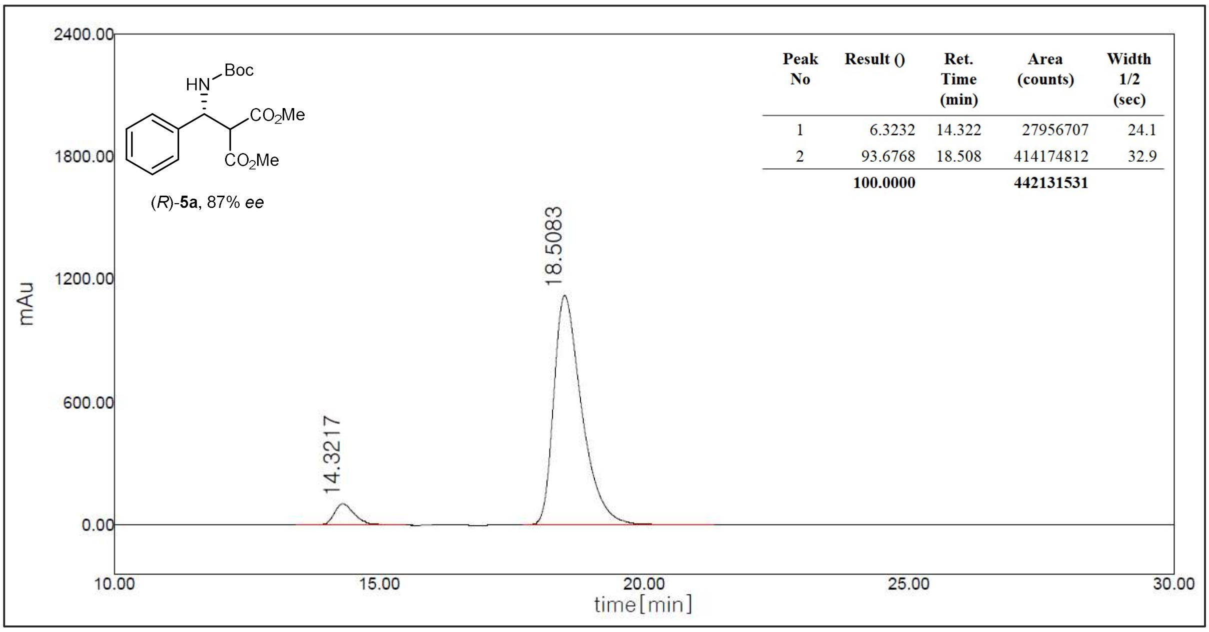

Peak No	Result ()	Ret. Time (min)	Area (counts)	Width 1/2 (sec)
1	6.3232	14.322	27956707	24.1
2	93.6768	18.508	414174812	32.9
	**100.0000**		**442131531**	

Supplementary Figure 82. HPLC spectra of **5a** (in H_2O/toluene)

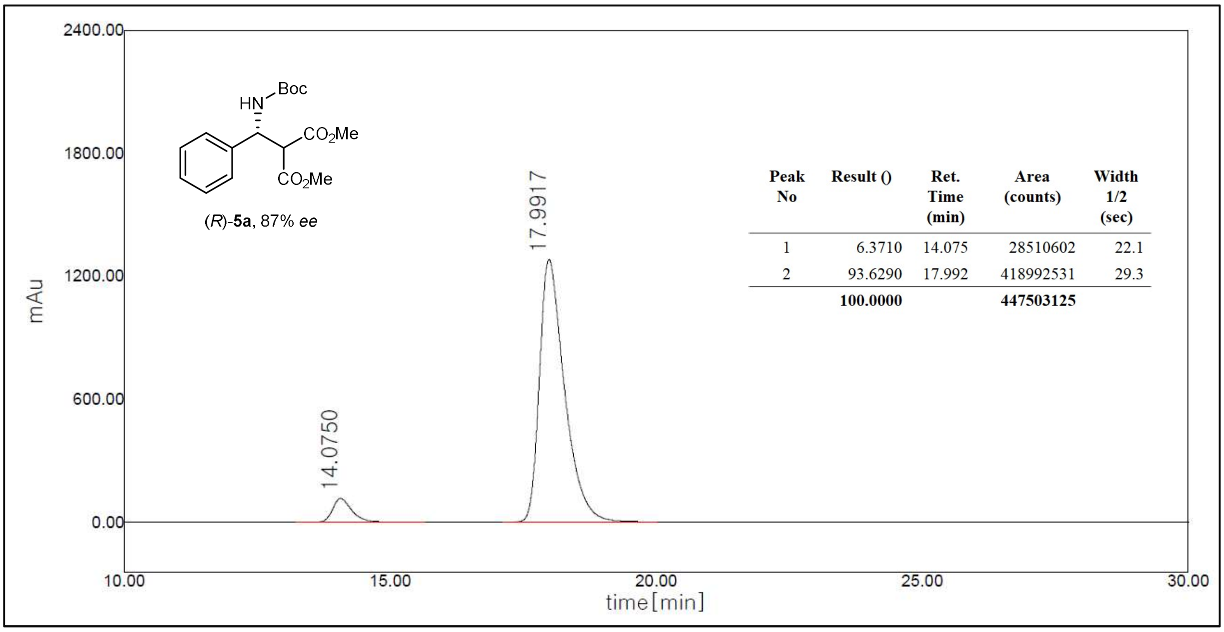

Peak No	Result ()	Ret. Time (min)	Area (counts)	Width 1/2 (sec)
1	6.3710	14.075	28510602	22.1
2	93.6290	17.992	418992531	29.3
	**100.0000**		**447503125**	

Supplementary Figure 83. HPLC spectra of **5a** (in brine/toluene)

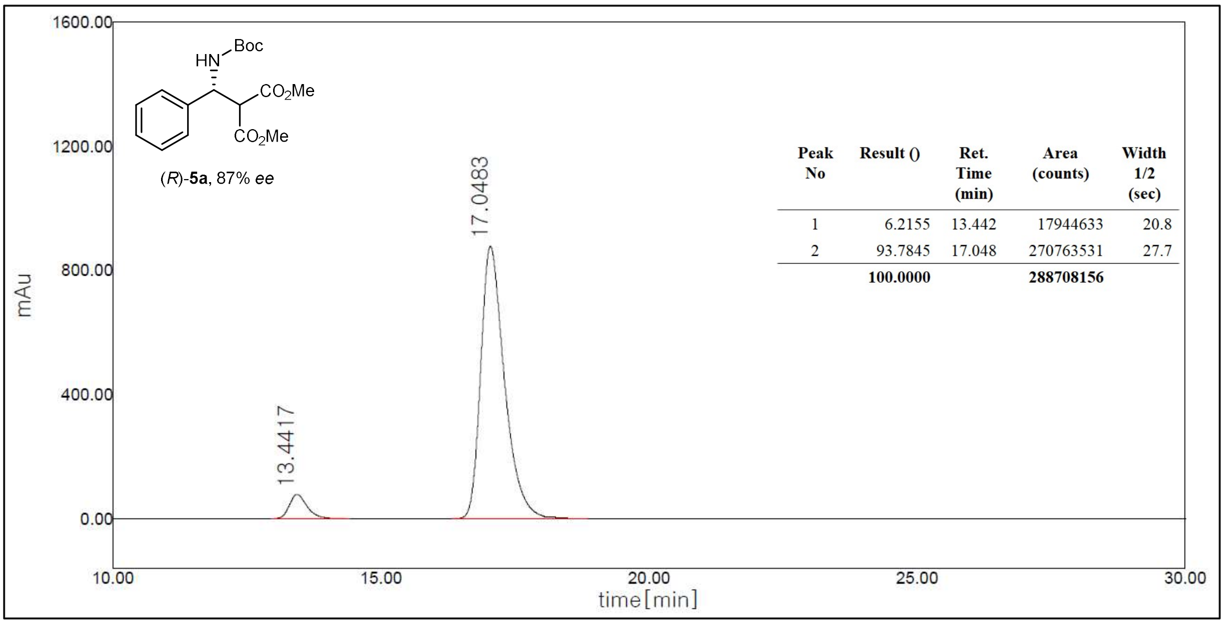

Peak No	Result ()	Ret. Time (min)	Area (counts)	Width 1/2 (sec)
1	6.2155	13.442	17944633	20.8
2	93.7845	17.048	270763531	27.7
	**100.0000**		**288708156**	

Supplementary Figure 84. HPLC spectra of **5a** (in brine/ *o*-xylene)

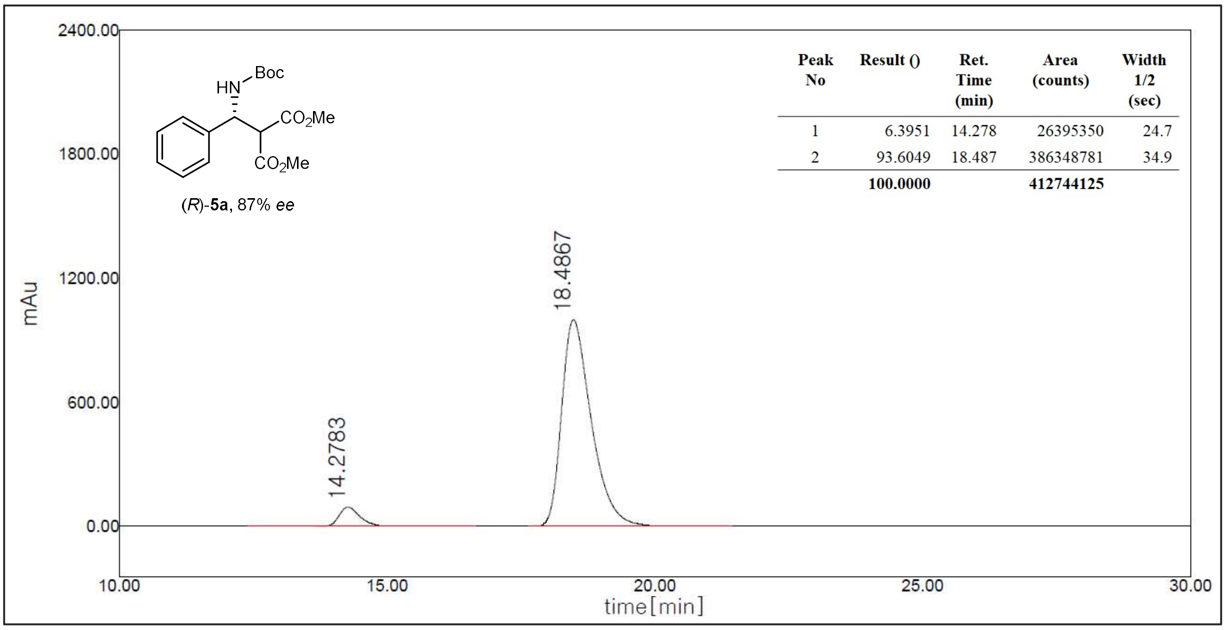

Supplementary Figure 85. HPLC spectra of **5a** (in D_2O/*o*-xylene)

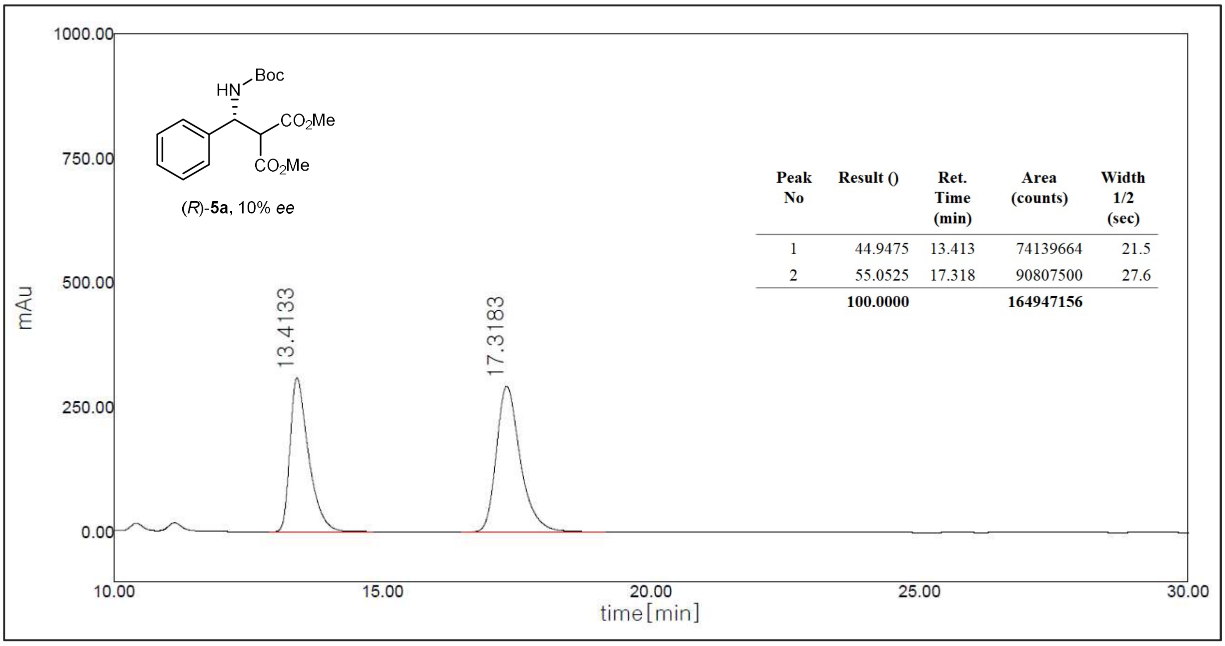

Supplementary Figure 86. HPLC spectra of **5a** (in aq. $LiClO_4$/*o*-xylene)

HPLC spectra of Fig. 2b

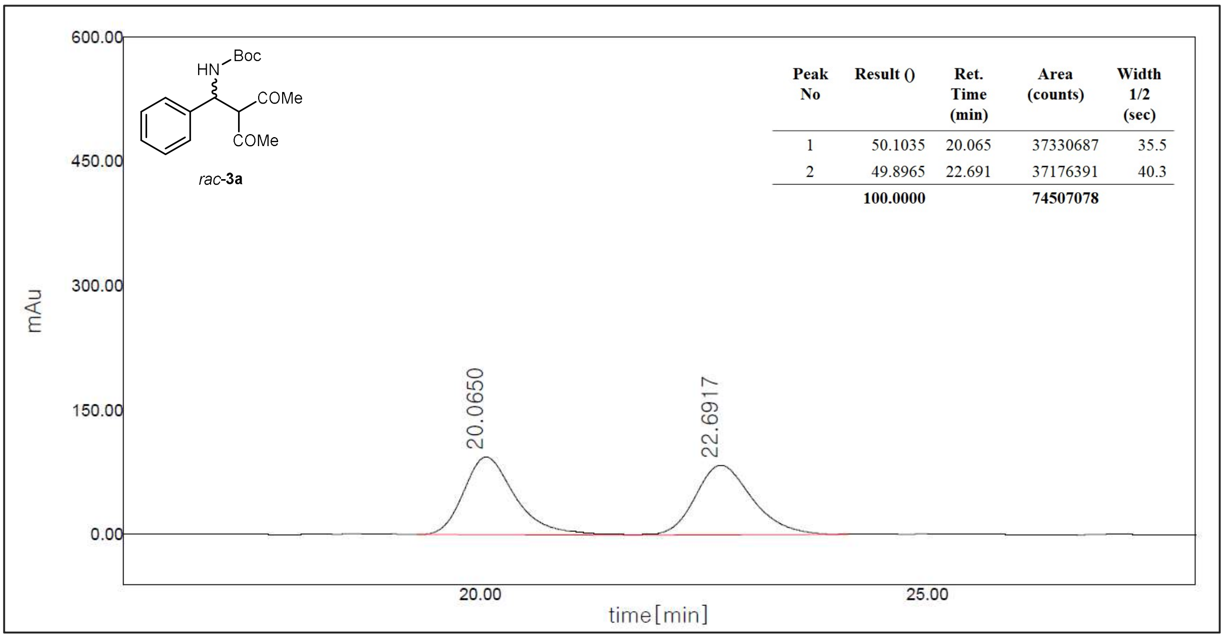

Peak No	Result ()	Ret. Time (min)	Area (counts)	Width 1/2 (sec)
1	50.1035	20.065	37330687	35.5
2	49.8965	22.691	37176391	40.3
	**100.0000**		**74507078**	

Supplementary Figure 87. HPLC spectra of *rac*-**3a**

With CN-derivatives/on water

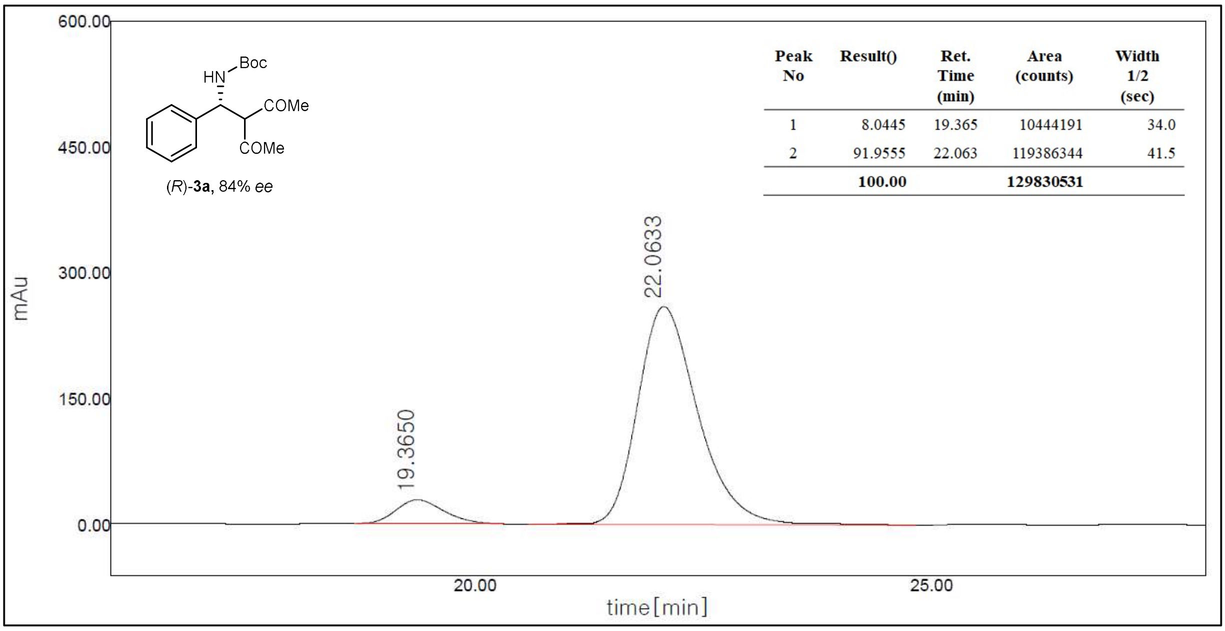

Peak No	Result()	Ret. Time (min)	Area (counts)	Width 1/2 (sec)
1	8.0445	19.365	10444191	34.0
2	91.9555	22.063	119386344	41.5
	**100.00**		**129830531**	

Supplementary Figure 88. HPLC spectra of **3a** (**CN-1** (Log *P* = 2.60), on water condition)

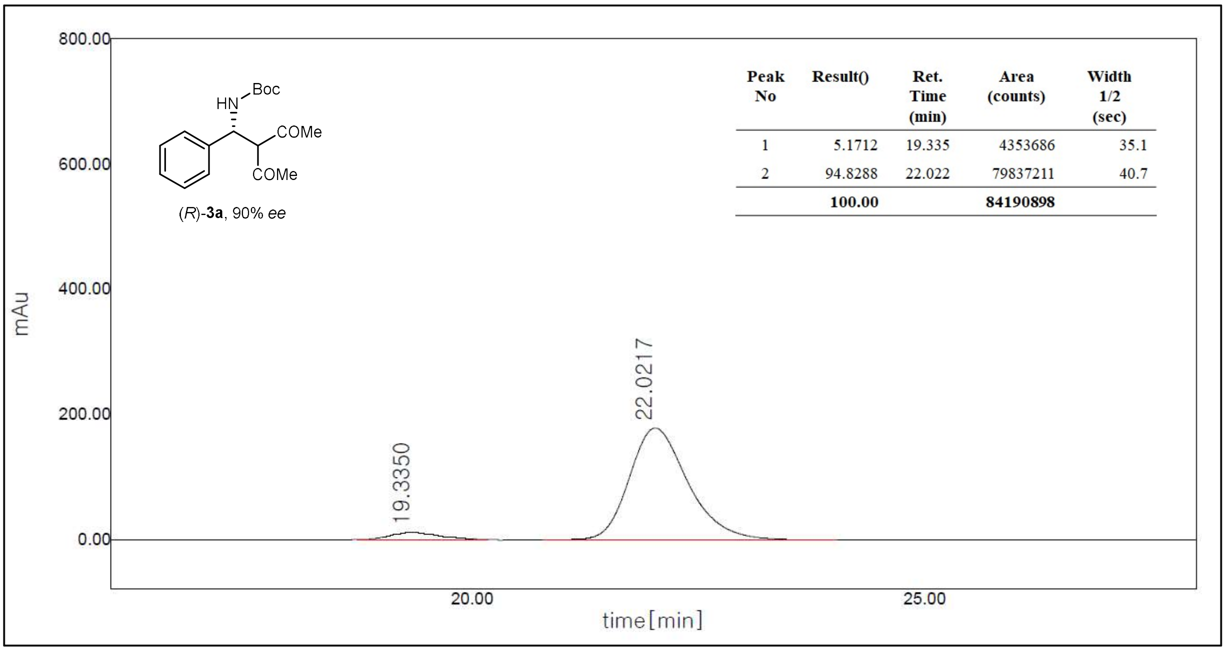

Supplementary Figure 89. HPLC spectra of **3a** (**CN-2** (Log *P* = 4.16), on water condition)

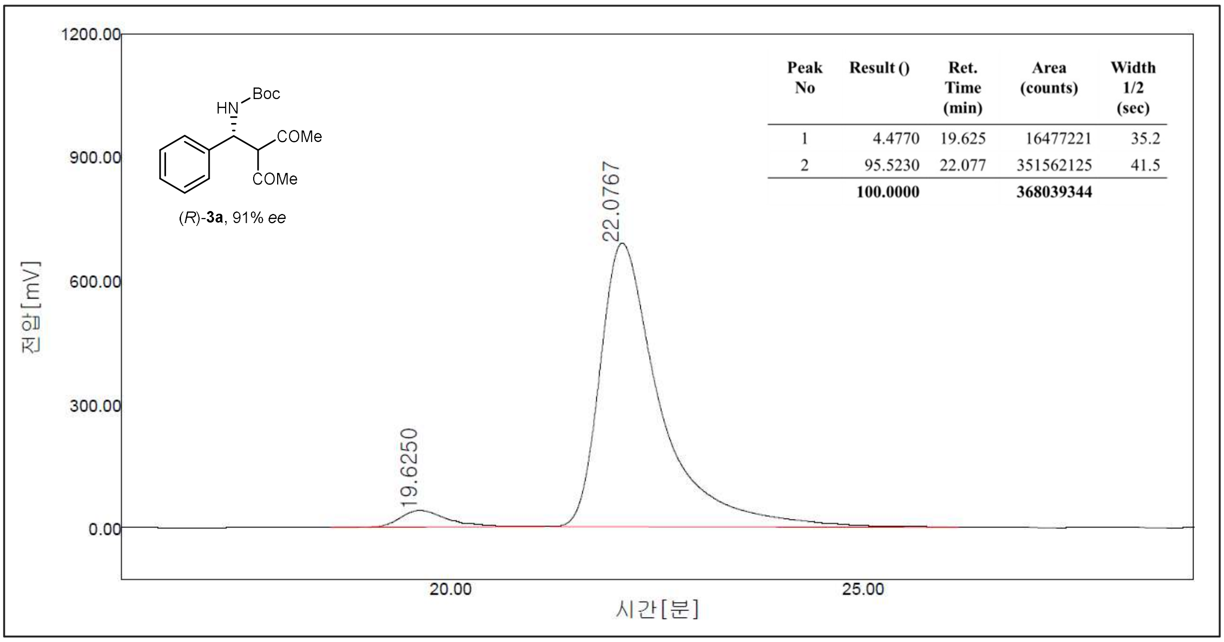

Supplementary Figure 90. HPLC spectra of **3a** (**CN-3** (Log *P* = 4.48), on water condition)

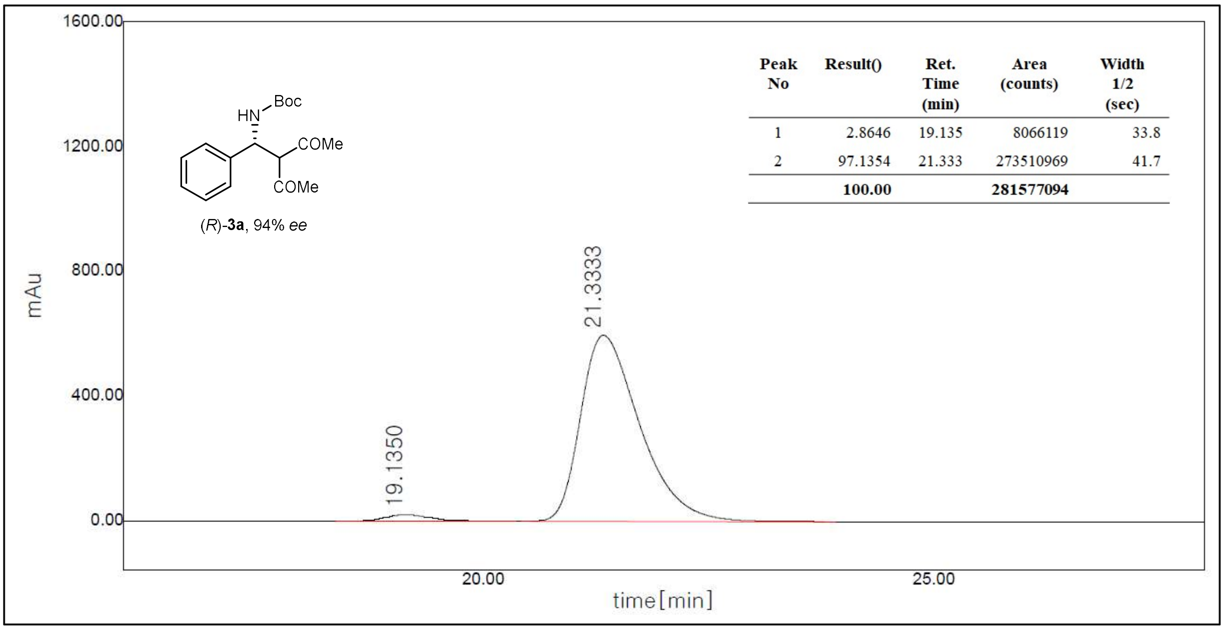

Peak No	Result()	Ret. Time (min)	Area (counts)	Width 1/2 (sec)
1	2.8646	19.135	8066119	33.8
2	97.1354	21.333	273510969	41.7
	**100.00**		**281577094**	

Supplementary Figure 91. HPLC spectra of **3a** (**CN-4** (Log *P* = 4.83), on water condition)

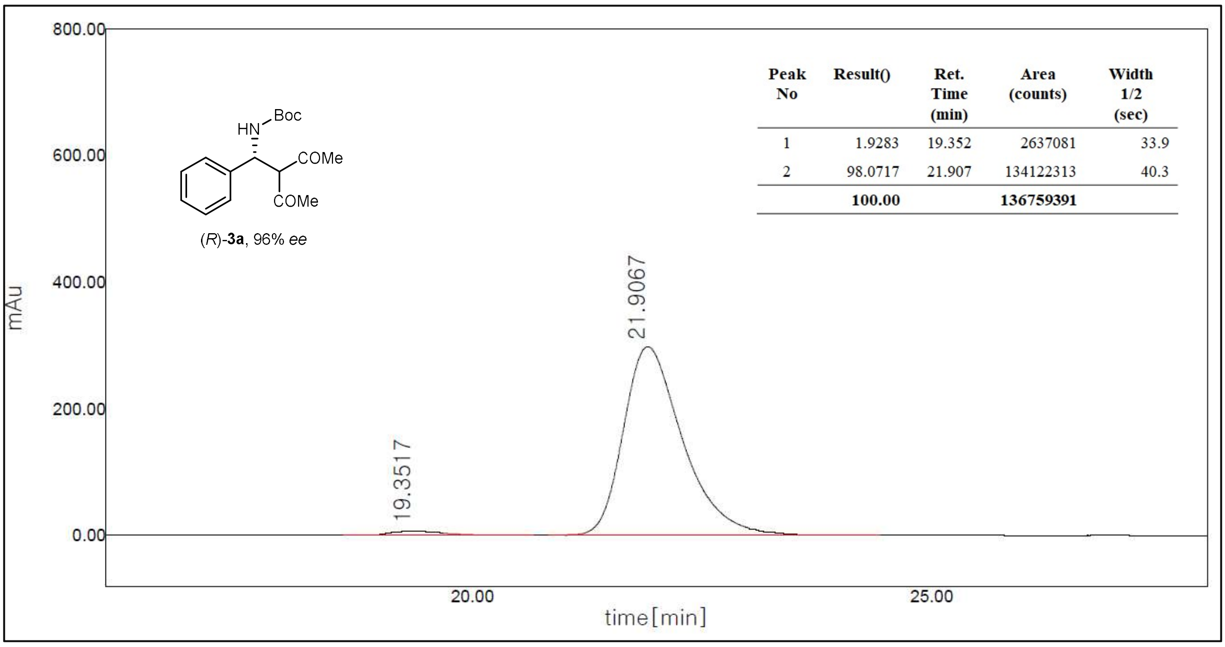

Peak No	Result()	Ret. Time (min)	Area (counts)	Width 1/2 (sec)
1	1.9283	19.352	2637081	33.9
2	98.0717	21.907	134122313	40.3
	**100.00**		**136759391**	

Supplementary Figure 92. HPLC spectra of **3a** (**CN-5** (Log *P* = 7.34), on water condition)

With CD-derivatives/on water

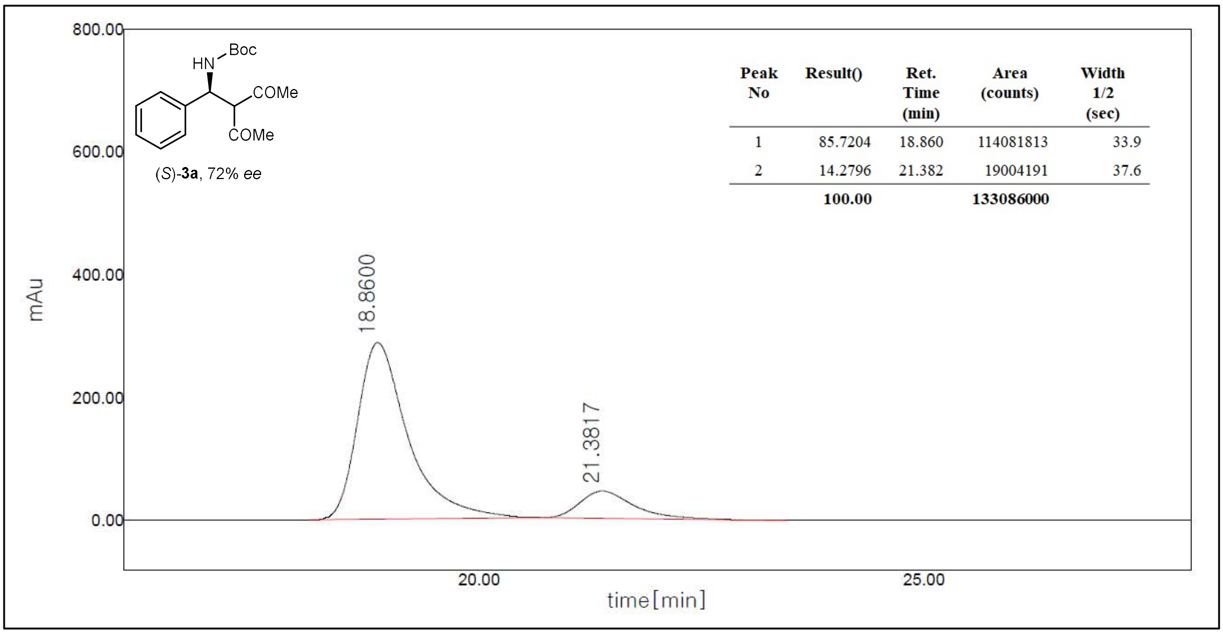

Peak No	Result()	Ret. Time (min)	Area (counts)	Width 1/2 (sec)
1	85.7204	18.860	114081813	33.9
2	14.2796	21.382	19004191	37.6
	**100.00**		**133086000**	

Supplementary Figure 93. HPLC spectra of **3a** (**CD-1** (Log *P* = 2.60), on water condition)

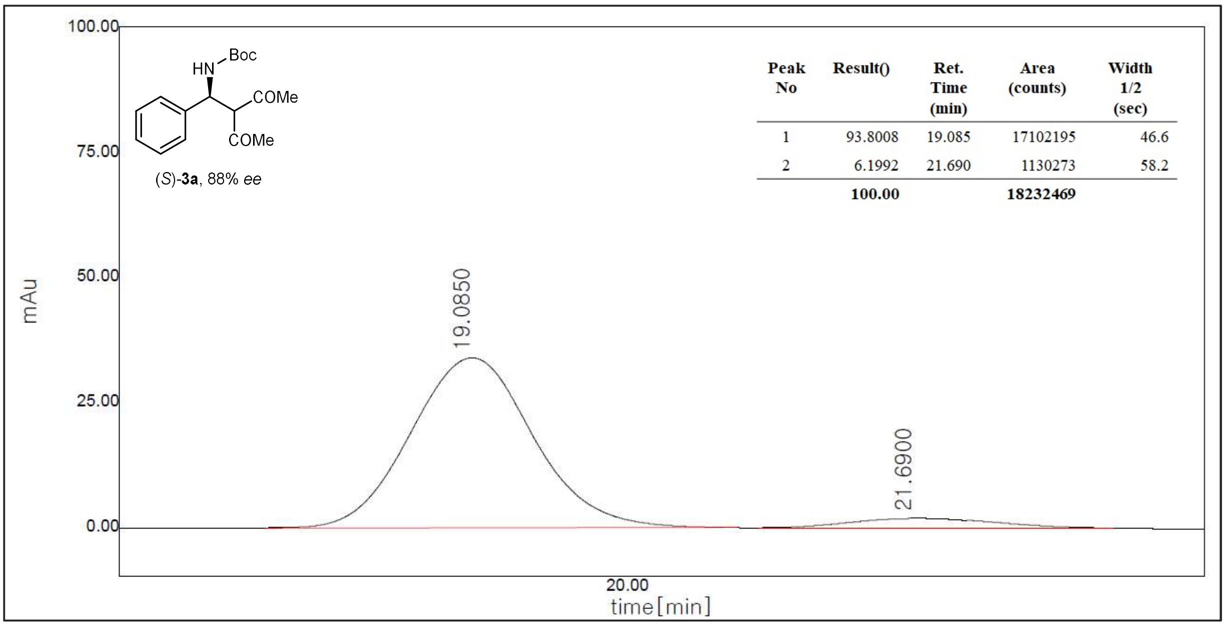

Peak No	Result()	Ret. Time (min)	Area (counts)	Width 1/2 (sec)
1	93.8008	19.085	17102195	46.6
2	6.1992	21.690	1130273	58.2
	**100.00**		**18232469**	

Supplementary Figure 94. HPLC spectra of **3a** (**CD-2** (Log *P* = 4.16), on water condition)

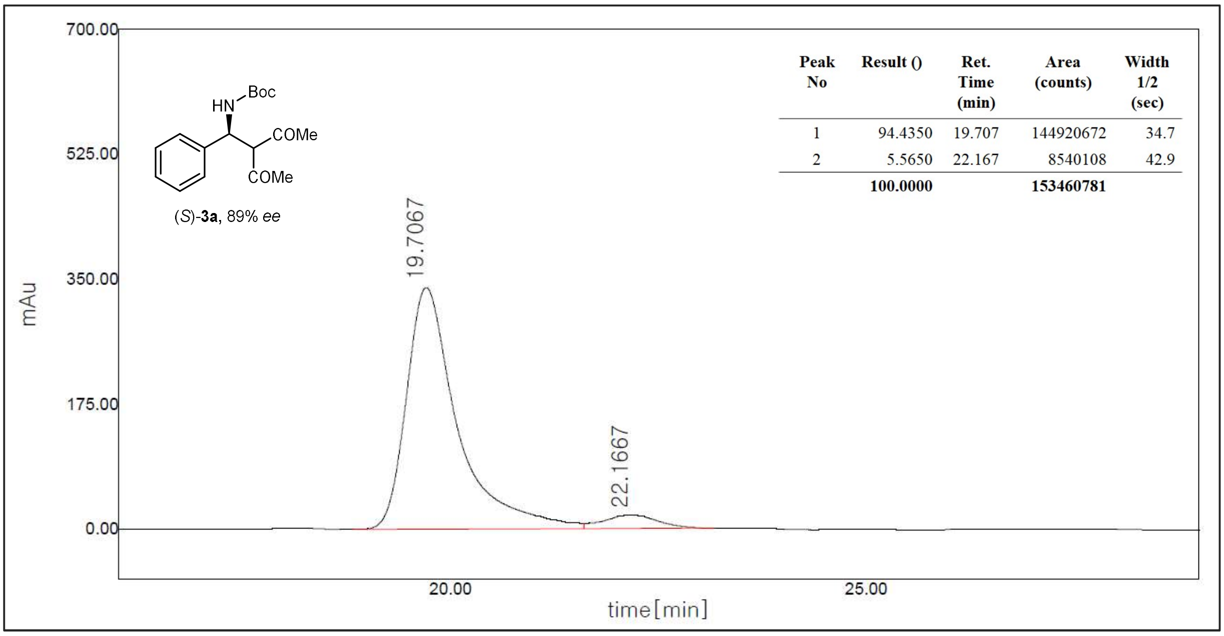

Peak No	Result ()	Ret. Time (min)	Area (counts)	Width 1/2 (sec)
1	94.4350	19.707	144920672	34.7
2	5.5650	22.167	8540108	42.9
	**100.0000**		**153460781**	

Supplementary Figure 95. HPLC spectra of **3a** (**CD-3** (Log *P* = 4.48), on water condition)

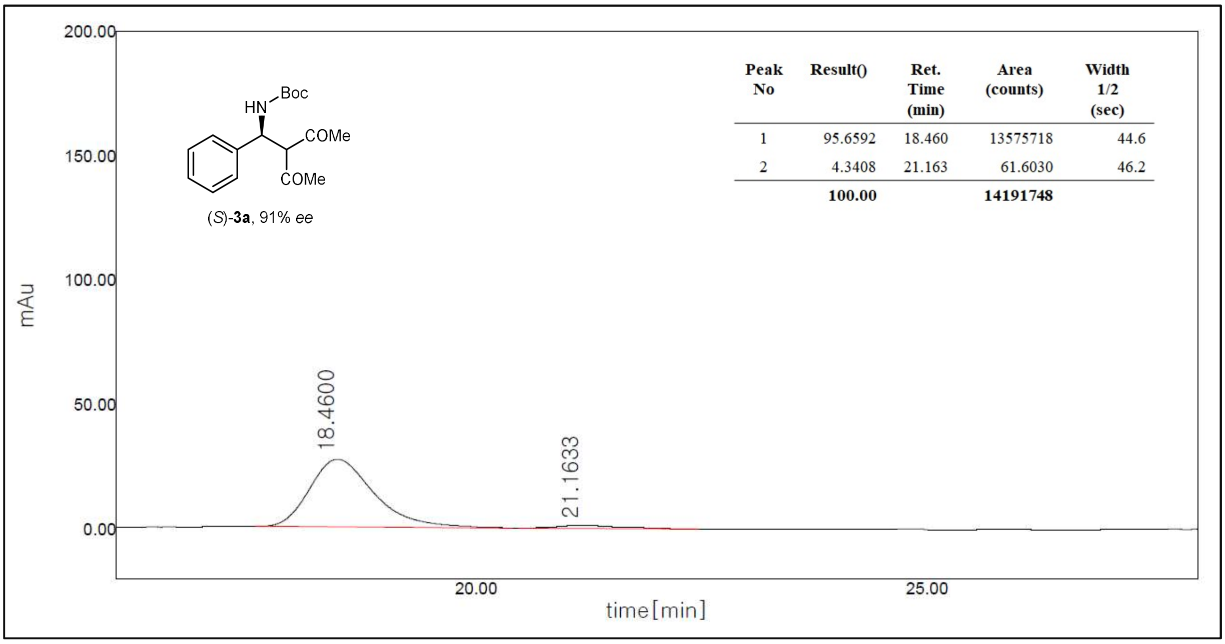

Peak No	Result()	Ret. Time (min)	Area (counts)	Width 1/2 (sec)
1	95.6592	18.460	13575718	44.6
2	4.3408	21.163	61.6030	46.2
	**100.00**		**14191748**	

Supplementary Figure 96. HPLC spectra of **3a** (**CD-4** (Log *P* = 4.83), on water condition)

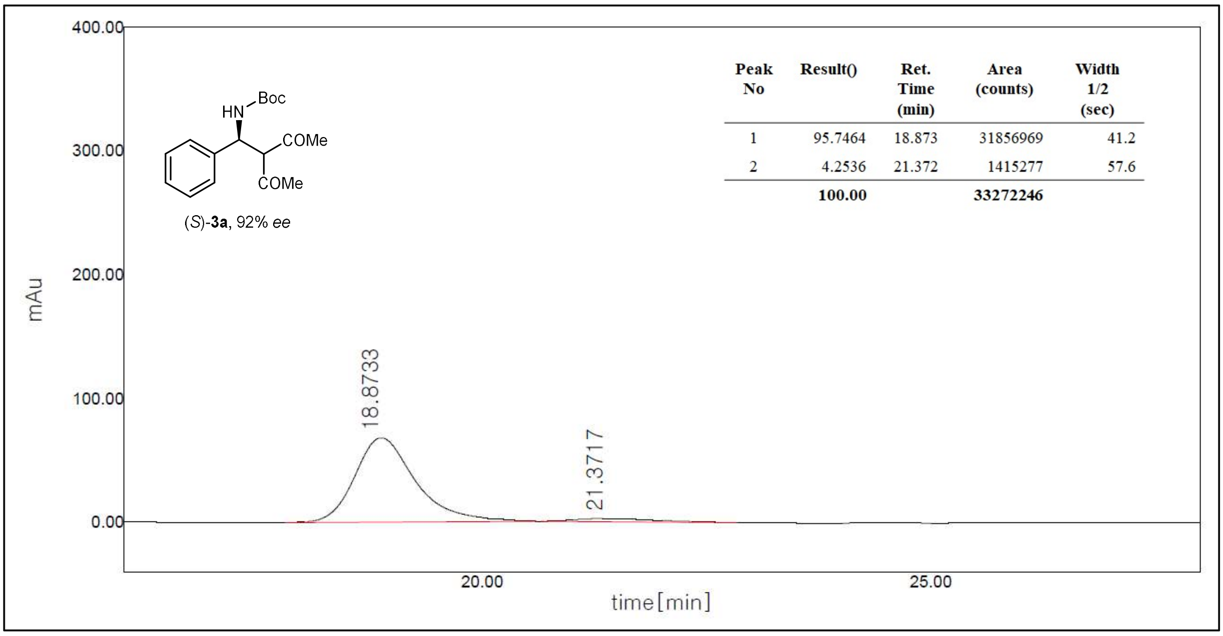

Peak No	Result()	Ret. Time (min)	Area (counts)	Width 1/2 (sec)
1	95.7464	18.873	31856969	41.2
2	4.2536	21.372	1415277	57.6
	**100.00**		**33272246**	

Supplementary Figure 97. HPLC spectra of **3a** (**CD-5** (Log *P* = 7.34), on water condition)

With CN-derivatives/in Dichloromethane

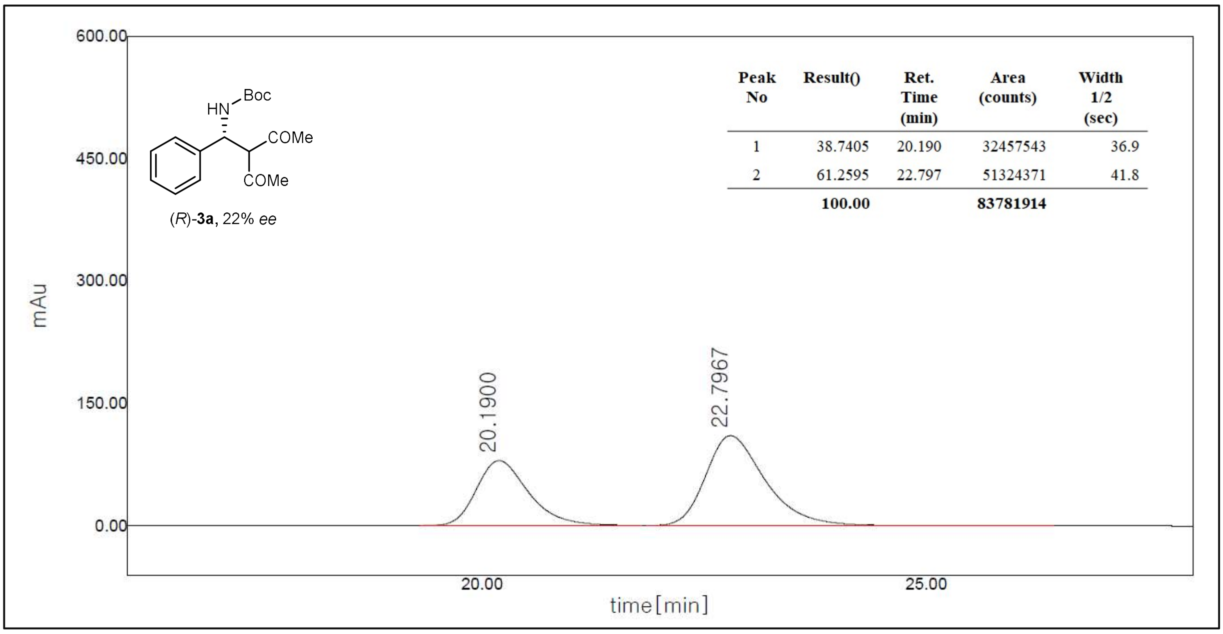

Peak No	Result()	Ret. Time (min)	Area (counts)	Width 1/2 (sec)
1	38.7405	20.190	32457543	36.9
2	61.2595	22.797	51324371	41.8
	**100.00**		**83781914**	

Supplementary Figure 98. HPLC spectra of **3a** (**CN-1** (Log *P* = 2.60), in dichloromethane)

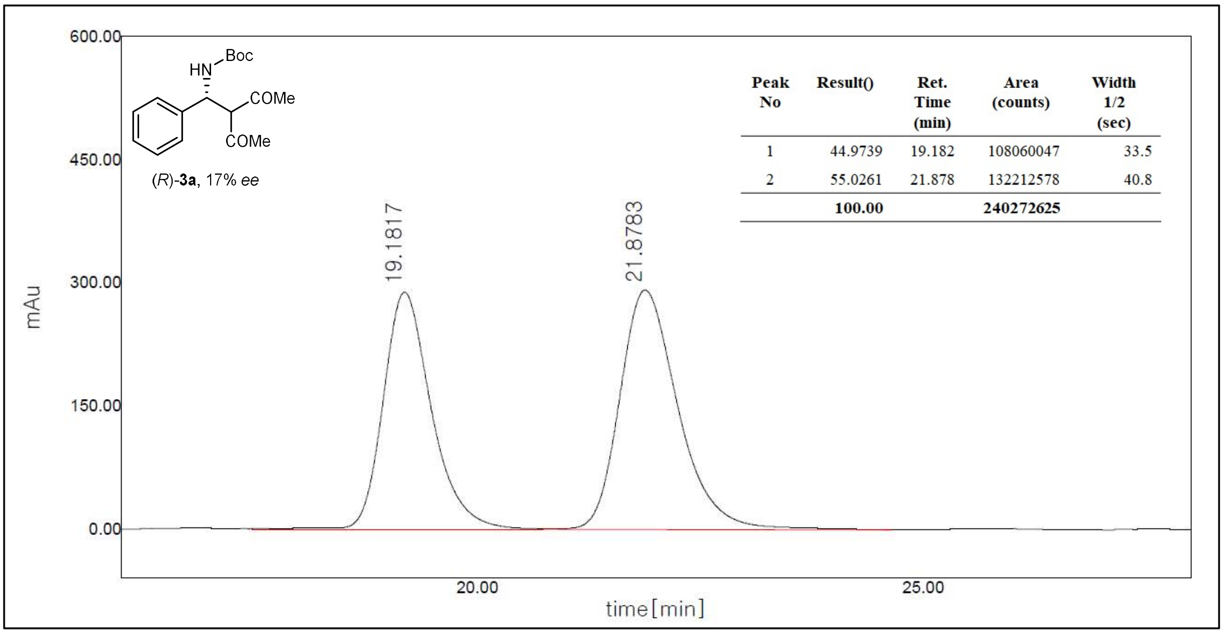

Supplementary Figure 99. HPLC spectra of **3a** (**CN-2** (Log *P* = 4.16), in dichloromethane)

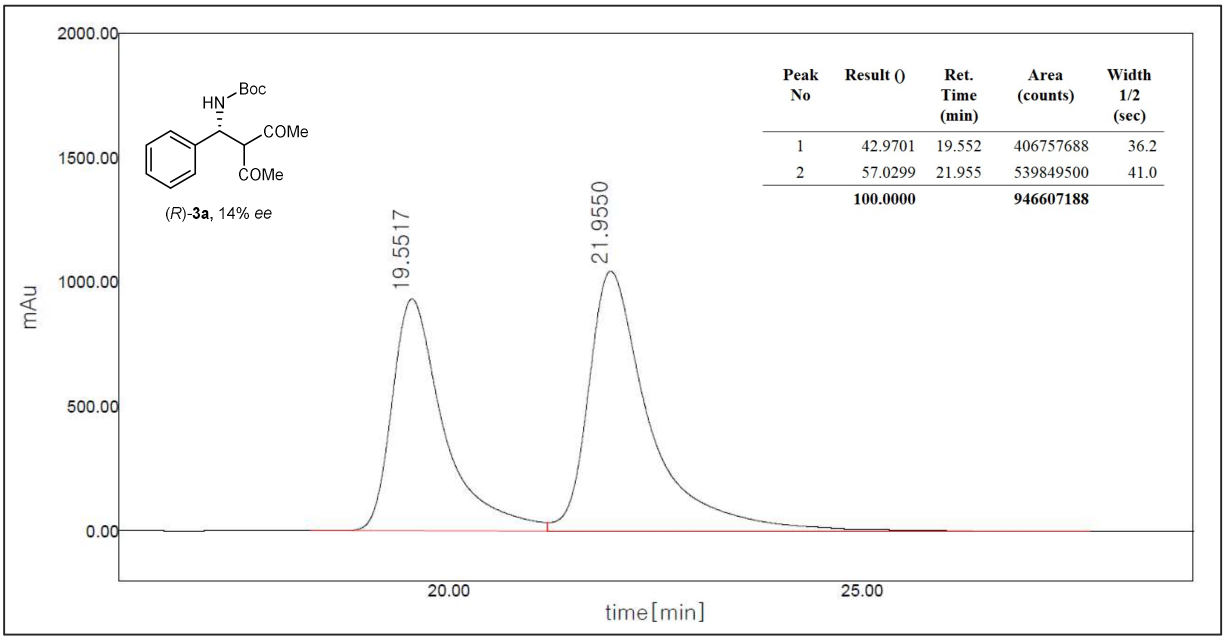

Supplementary Figure 100. HPLC spectra of **3a** (**CN-3** (Log *P* = 4.48), in dichloromethane)

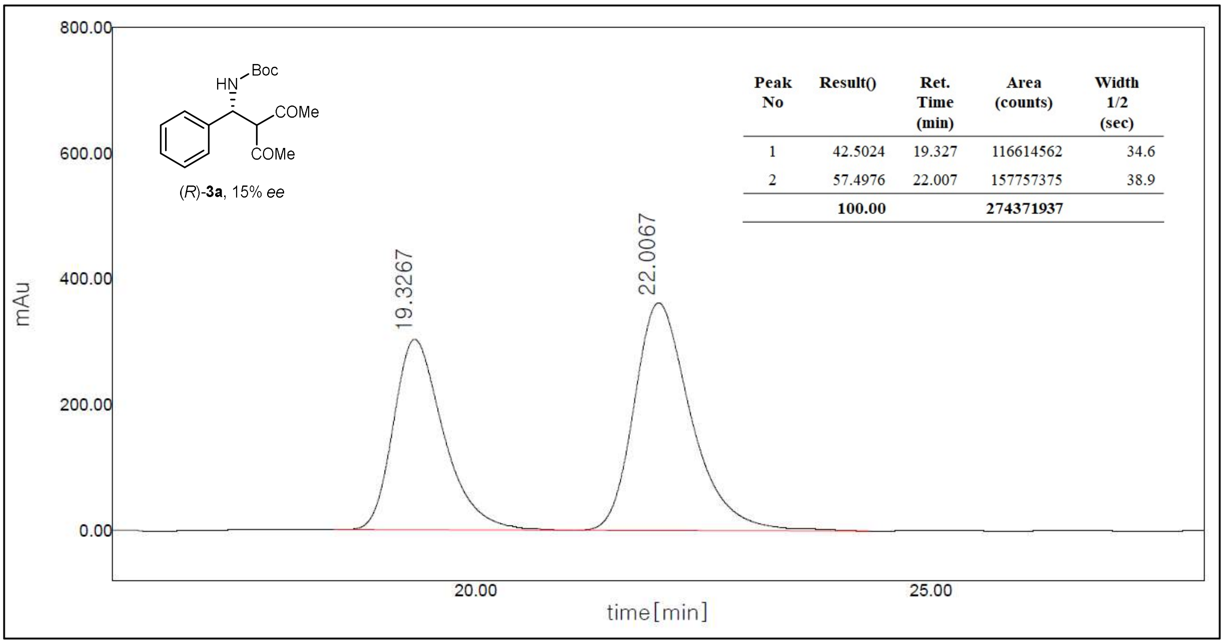

Peak No	Result()	Ret. Time (min)	Area (counts)	Width 1/2 (sec)
1	42.5024	19.327	116614562	34.6
2	57.4976	22.007	157757375	38.9
	**100.00**		**274371937**	

Supplementary Figure 101. HPLC spectra of **3a** (**CN-4** (Log *P* = 4.83), in dichloromethane)

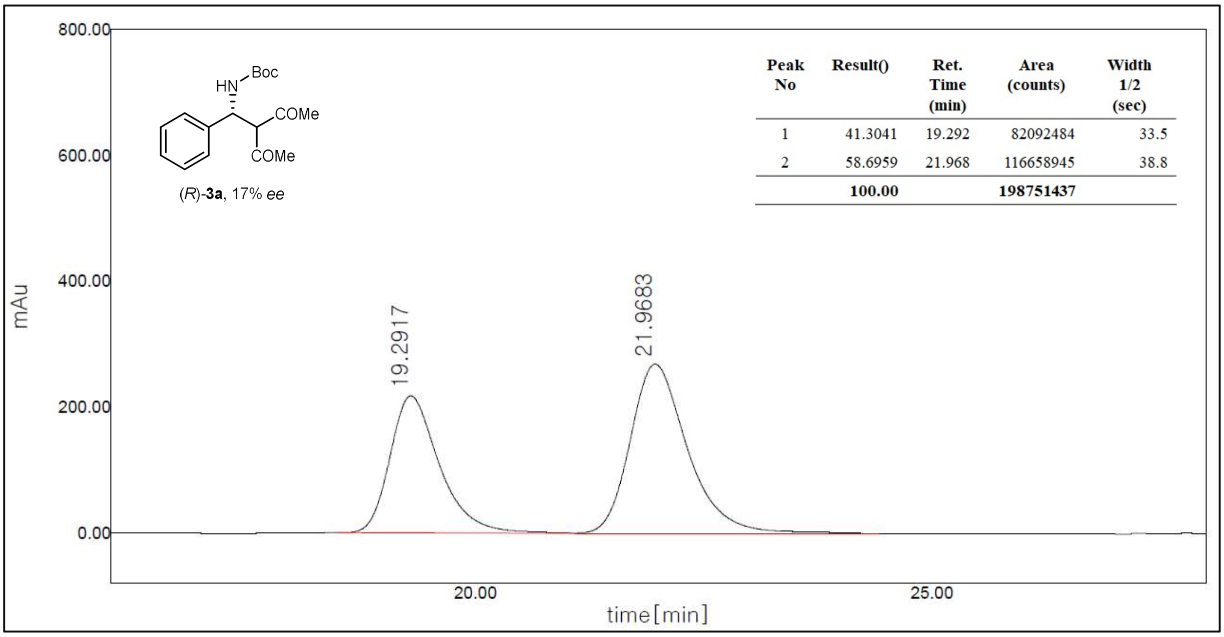

Peak No	Result()	Ret. Time (min)	Area (counts)	Width 1/2 (sec)
1	41.3041	19.292	82092484	33.5
2	58.6959	21.968	116658945	38.8
	**100.00**		**198751437**	

Supplementary Figure 102. HPLC spectra of **3a** (**CN-5** (Log *P* = 7.34), in dichloromethane)

With CD-derivatives/in Dichloromethane

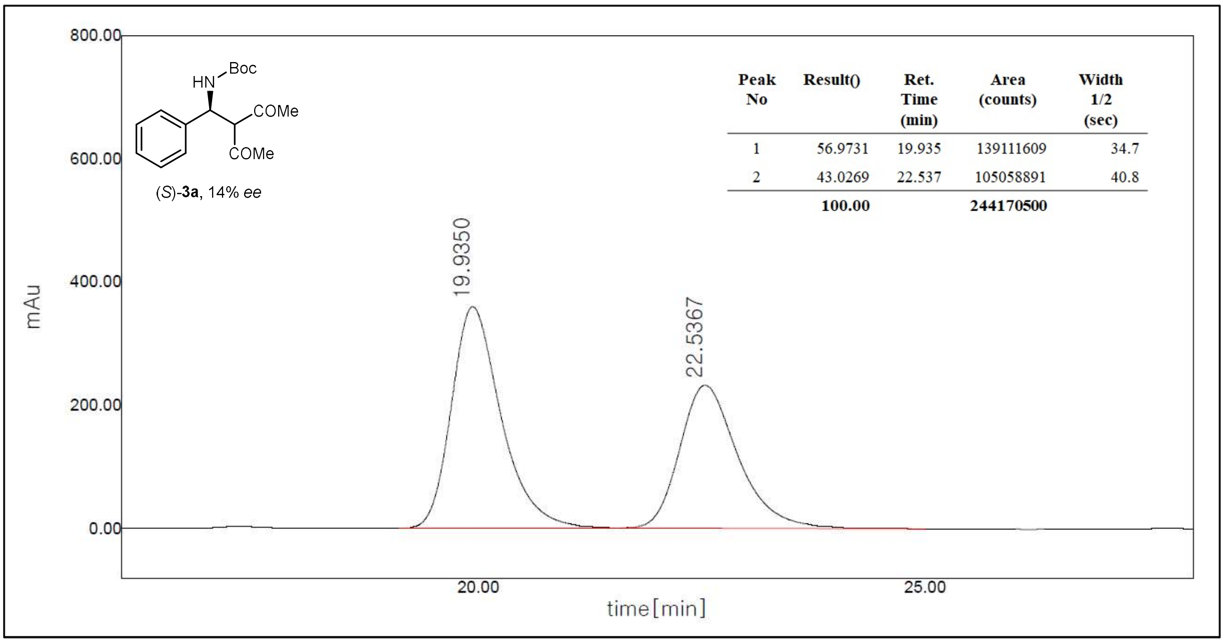

Peak No	Result()	Ret. Time (min)	Area (counts)	Width 1/2 (sec)
1	56.9731	19.935	139111609	34.7
2	43.0269	22.537	105058891	40.8
	**100.00**		**244170500**	

Supplementary Figure 103. HPLC spectra of **3a** (**CD-1** (Log *P* = 2.60), in dichloromethane)

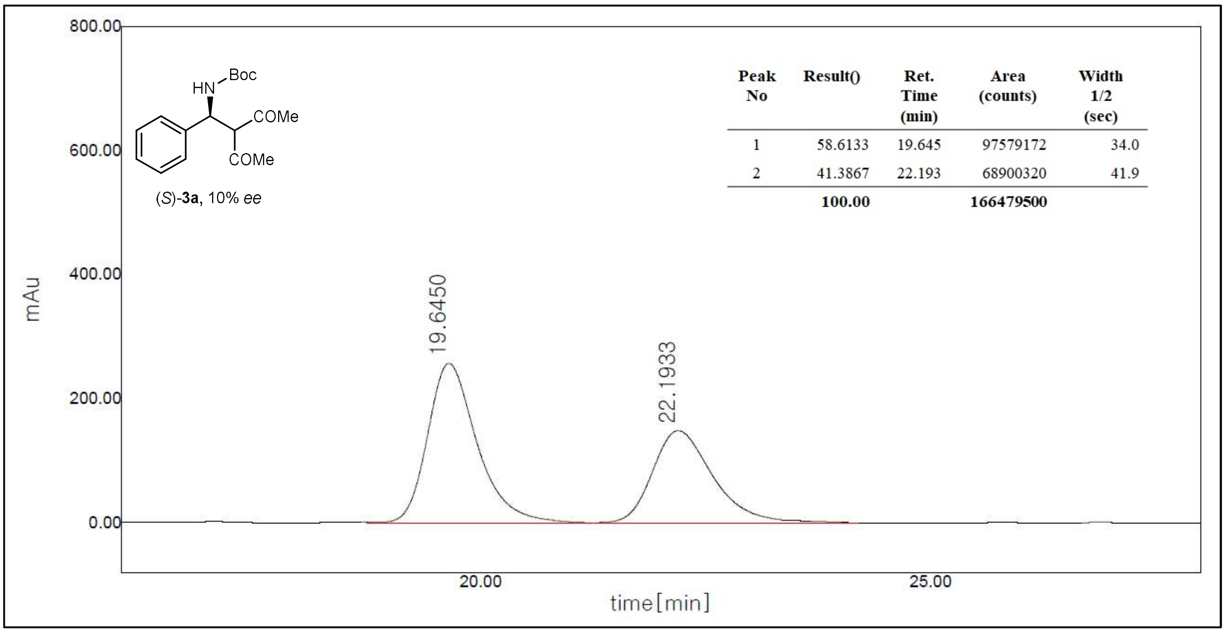

Peak No	Result()	Ret. Time (min)	Area (counts)	Width 1/2 (sec)
1	58.6133	19.645	97579172	34.0
2	41.3867	22.193	68900320	41.9
	**100.00**		**166479500**	

Supplementary Figure 104. HPLC spectra of **3a** (**CD-2** (Log *P* = 4.16), in dichloromethane)

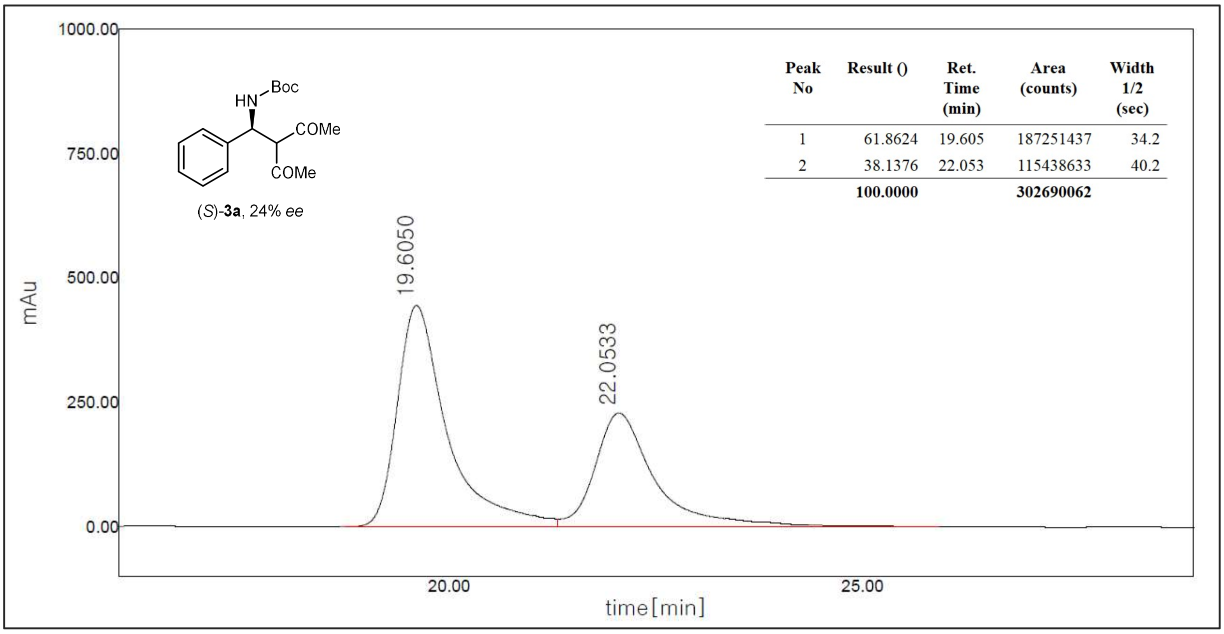

Peak No	Result ()	Ret. Time (min)	Area (counts)	Width 1/2 (sec)
1	61.8624	19.605	187251437	34.2
2	38.1376	22.053	115438633	40.2
	**100.0000**		**302690062**	

Supplementary Figure 105. HPLC spectra of **3a** (**CD-3** (Log *P* = 4.48), in dichloromethane)

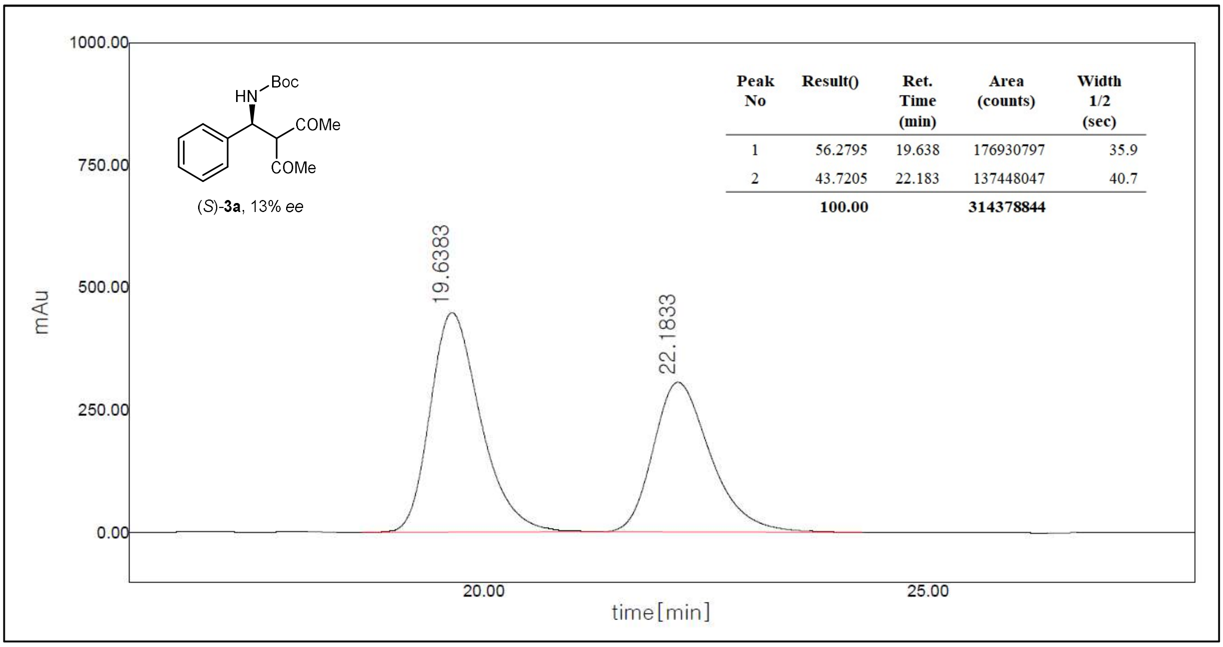

Peak No	Result()	Ret. Time (min)	Area (counts)	Width 1/2 (sec)
1	56.2795	19.638	176930797	35.9
2	43.7205	22.183	137448047	40.7
	**100.00**		**314378844**	

Supplementary Figure 106. HPLC spectra of **3a** (**CD-4** (Log *P* = 4.83), in dichloromethane)

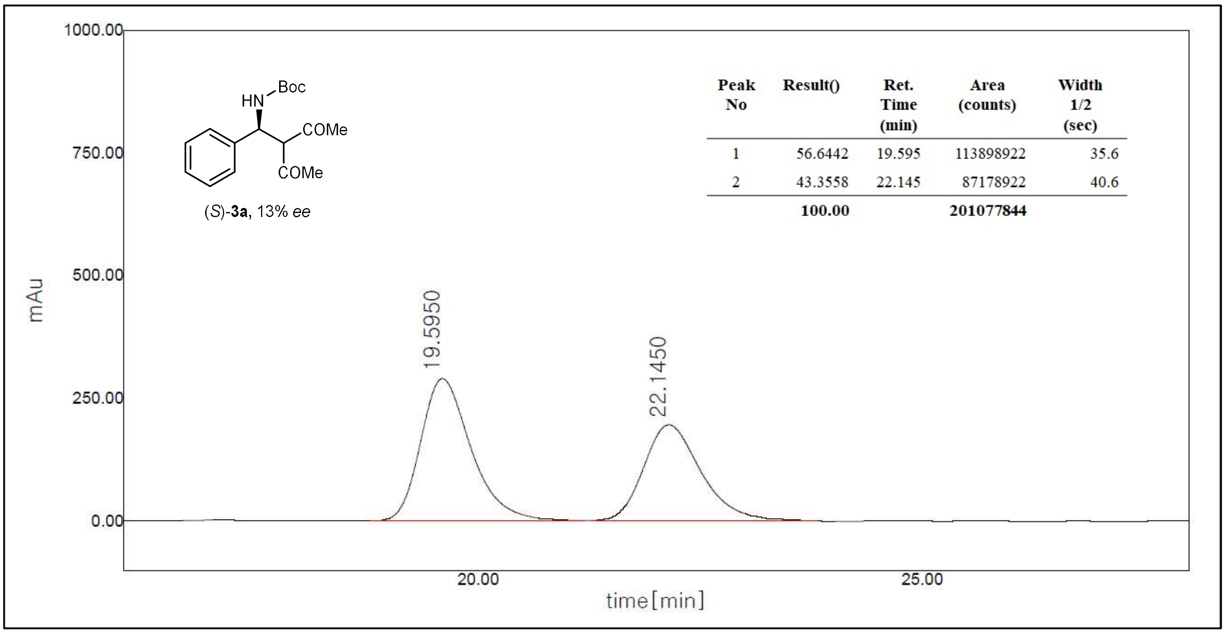

Supplementary Figure 107. HPLC spectra of **3a** (**CD-5** (Log *P* = 7.34), in dichloromethane)

HPLC spectra of Supplementary Table 2

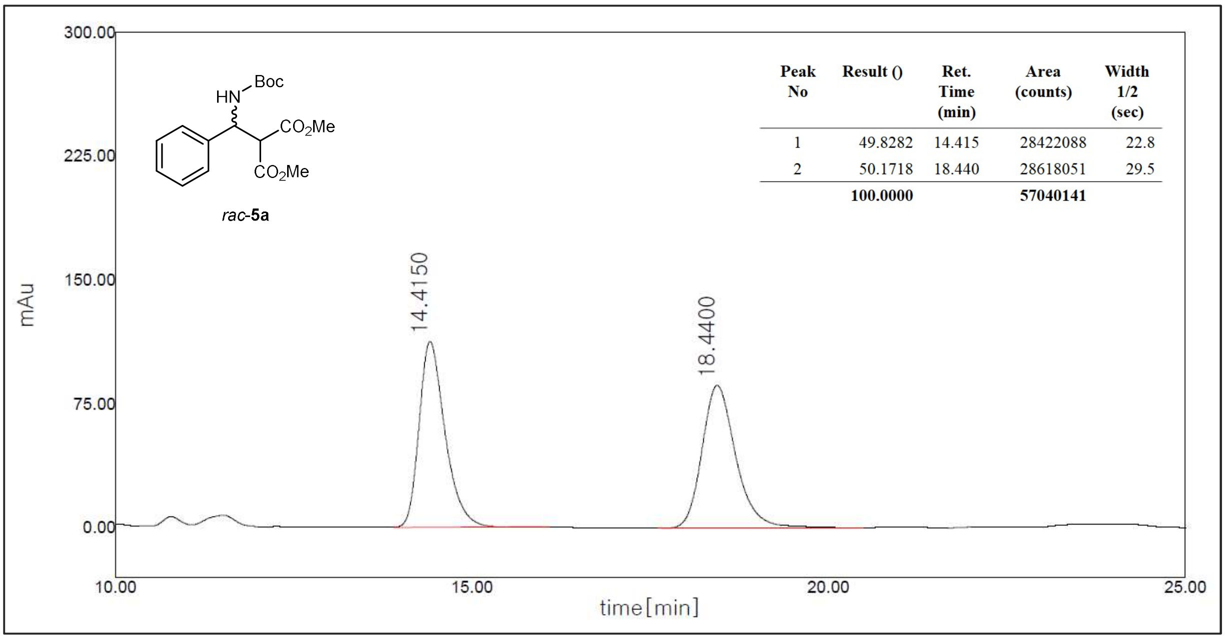

Peak No	Result ()	Ret. Time (min)	Area (counts)	Width 1/2 (sec)
1	49.8282	14.415	28422088	22.8
2	50.1718	18.440	28618051	29.5
	**100.0000**		**57040141**	

Supplementary Figure 108. HPLC spectra of *rac*-**5a**

With CN-derivatives/on water

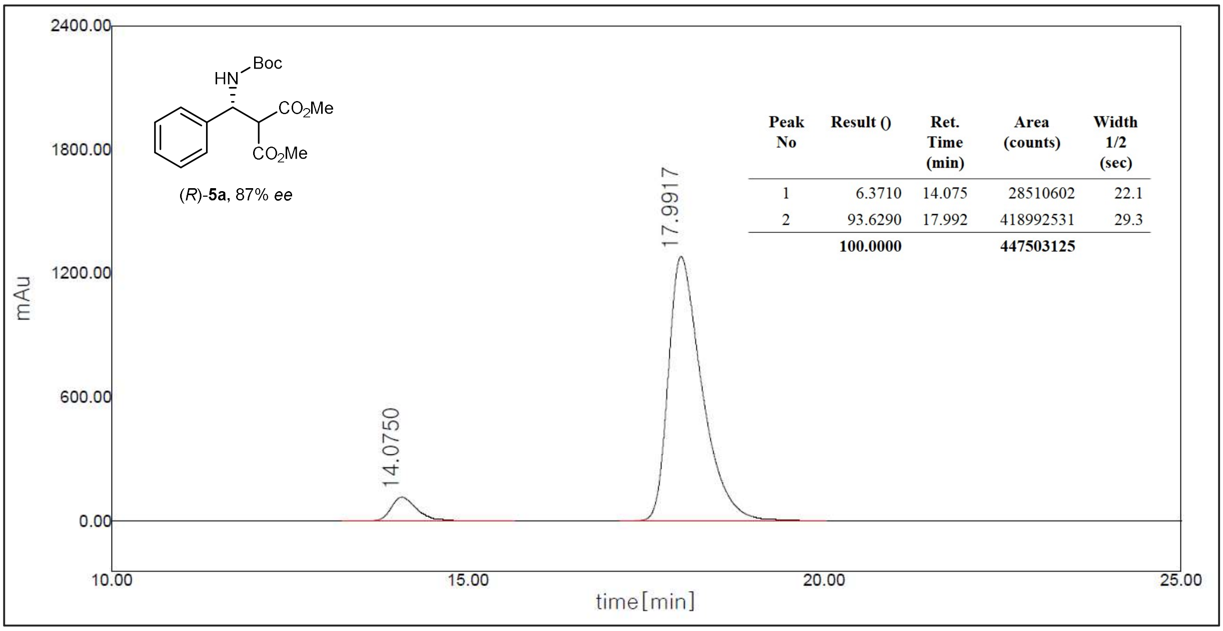

Peak No	Result ()	Ret. Time (min)	Area (counts)	Width 1/2 (sec)
1	6.3710	14.075	28510602	22.1
2	93.6290	17.992	418992531	29.3
	**100.0000**		**447503125**	

Supplementary Figure 109. HPLC spectra of **5a** (**CN-1** (Log P = 2.60), on water condition)

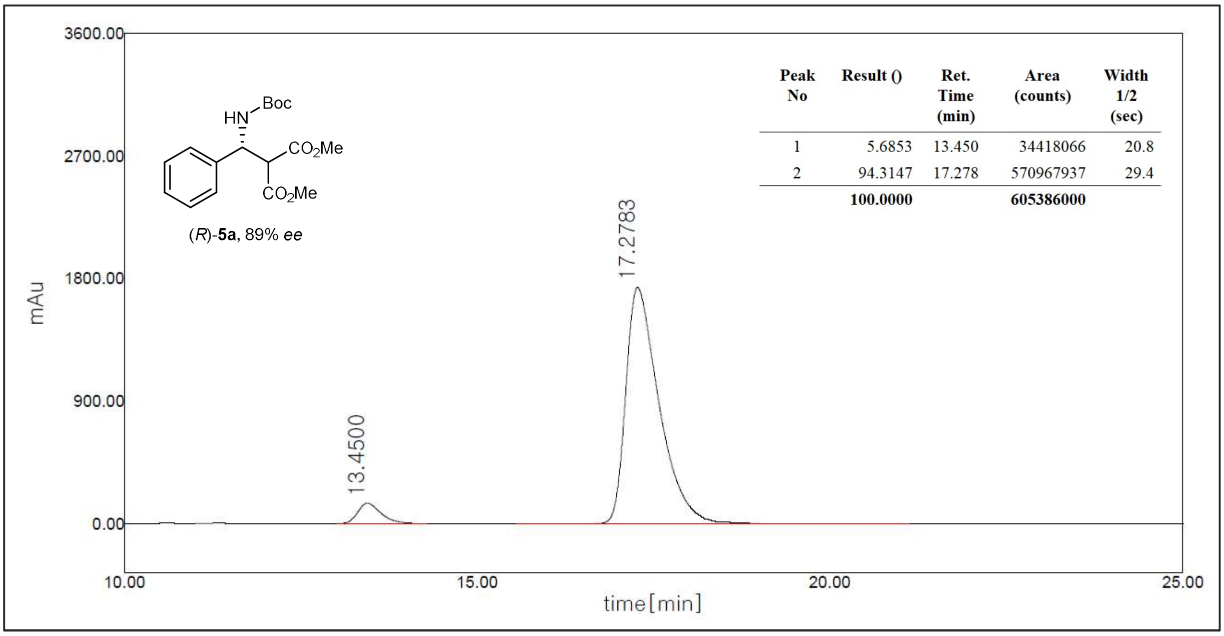

Peak No	Result ()	Ret. Time (min)	Area (counts)	Width 1/2 (sec)
1	5.6853	13.450	34418066	20.8
2	94.3147	17.278	570967937	29.4
	**100.0000**		**605386000**	

Supplementary Figure 110. HPLC spectra of **5a** (**CN-2** (Log *P* = 4.16), on water condition)

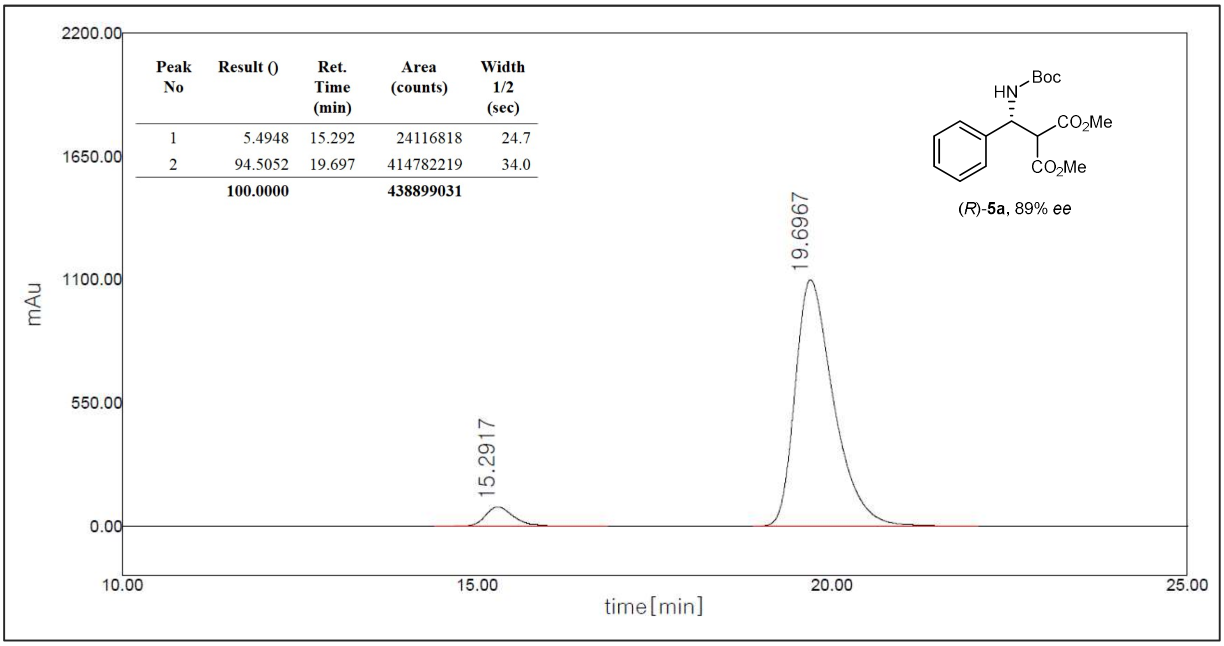

Peak No	Result ()	Ret. Time (min)	Area (counts)	Width 1/2 (sec)
1	5.4948	15.292	24116818	24.7
2	94.5052	19.697	414782219	34.0
	**100.0000**		**438899031**	

Supplementary Figure 111. HPLC spectra of **5a** (**CN-3** (Log *P* = 4.48), on water condition)

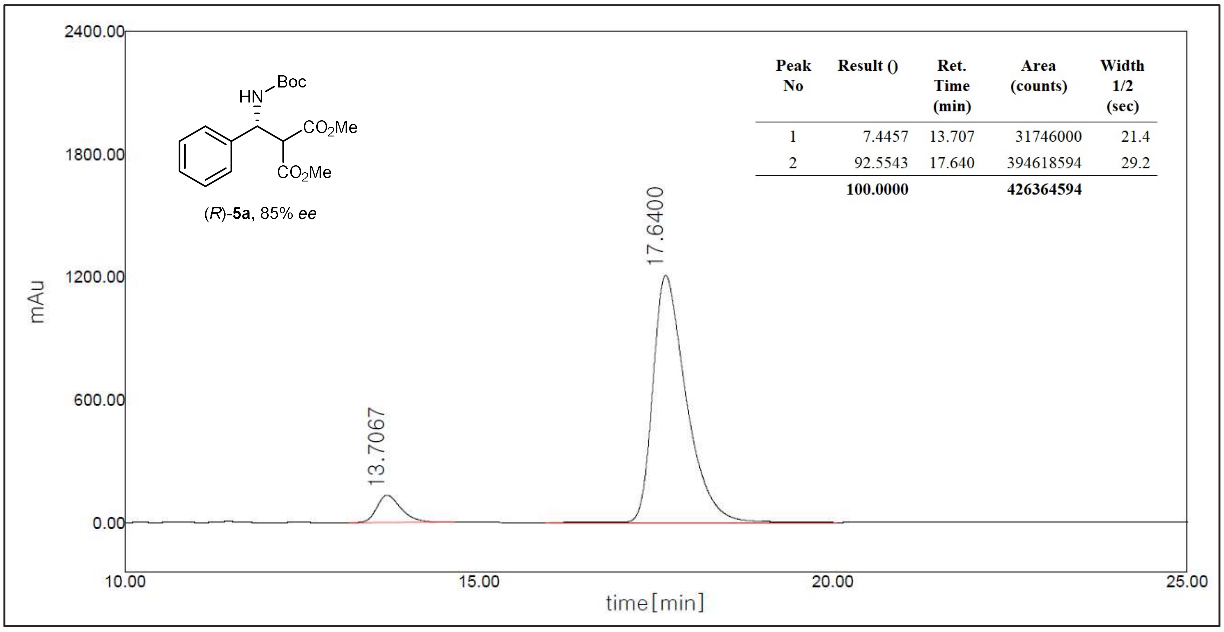

Peak No	Result ()	Ret. Time (min)	Area (counts)	Width 1/2 (sec)
1	7.4457	13.707	31746000	21.4
2	92.5543	17.640	394618594	29.2
	**100.0000**		**426364594**	

Supplementary Figure 112. HPLC spectra of **5a** (**CN-4** (Log *P* = 4.83), on water condition)

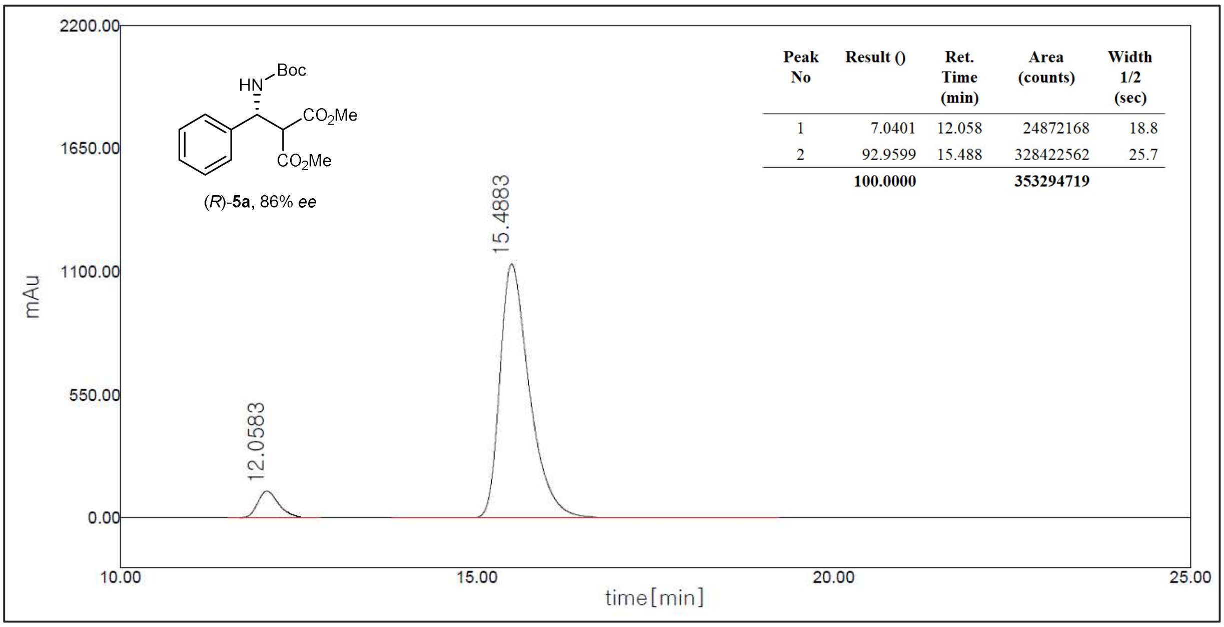

Peak No	Result ()	Ret. Time (min)	Area (counts)	Width 1/2 (sec)
1	7.0401	12.058	24872168	18.8
2	92.9599	15.488	328422562	25.7
	**100.0000**		**353294719**	

Supplementary Figure 113. HPLC spectra of **5a** (**CN-5** (Log *P* = 7.34), on water condition)

With CD-derivatives/on water

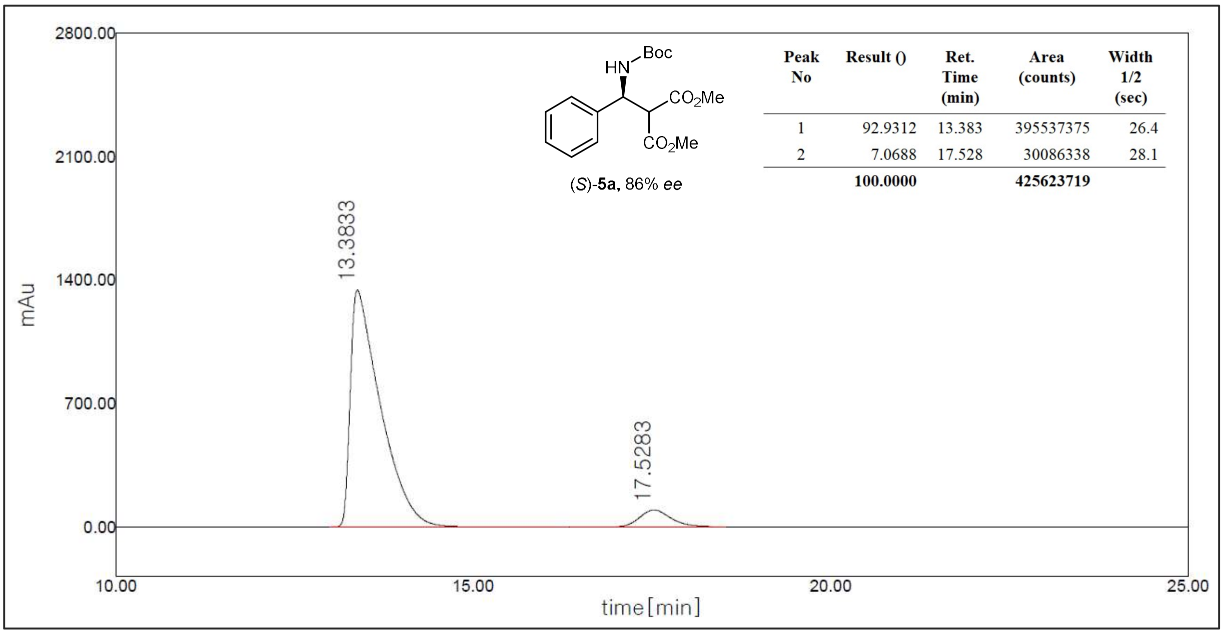

Peak No	Result ()	Ret. Time (min)	Area (counts)	Width 1/2 (sec)
1	92.9312	13.383	395537375	26.4
2	7.0688	17.528	30086338	28.1
	**100.0000**		**425623719**	

Supplementary Figure 114. HPLC spectra of **5a** (**CD-1** (Log *P* = 2.60), on water condition)

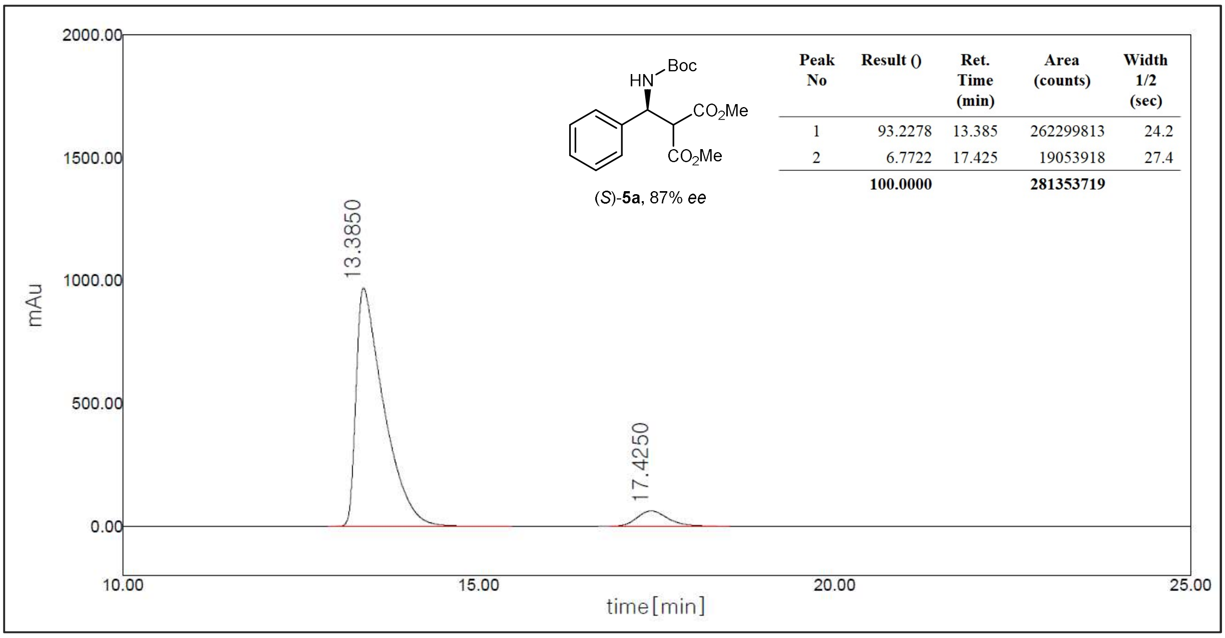

Peak No	Result ()	Ret. Time (min)	Area (counts)	Width 1/2 (sec)
1	93.2278	13.385	262299813	24.2
2	6.7722	17.425	19053918	27.4
	**100.0000**		**281353719**	

Supplementary Figure 115. HPLC spectra of **5a** (**CD-2** (Log *P* = 4.16), on water condition)

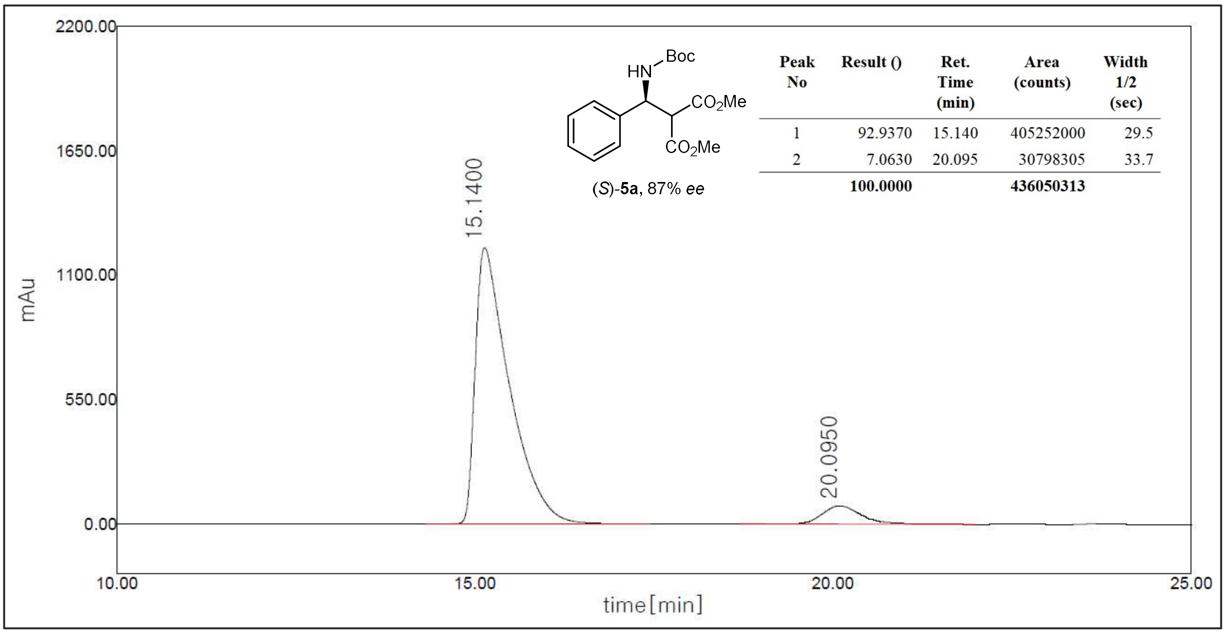

Peak No	Result ()	Ret. Time (min)	Area (counts)	Width 1/2 (sec)
1	92.9370	15.140	405252000	29.5
2	7.0630	20.095	30798305	33.7
	**100.0000**		**436050313**	

Supplementary Figure 116. HPLC spectra of **5a** (**CD-3** (Log *P* = 4.48), on water condition)

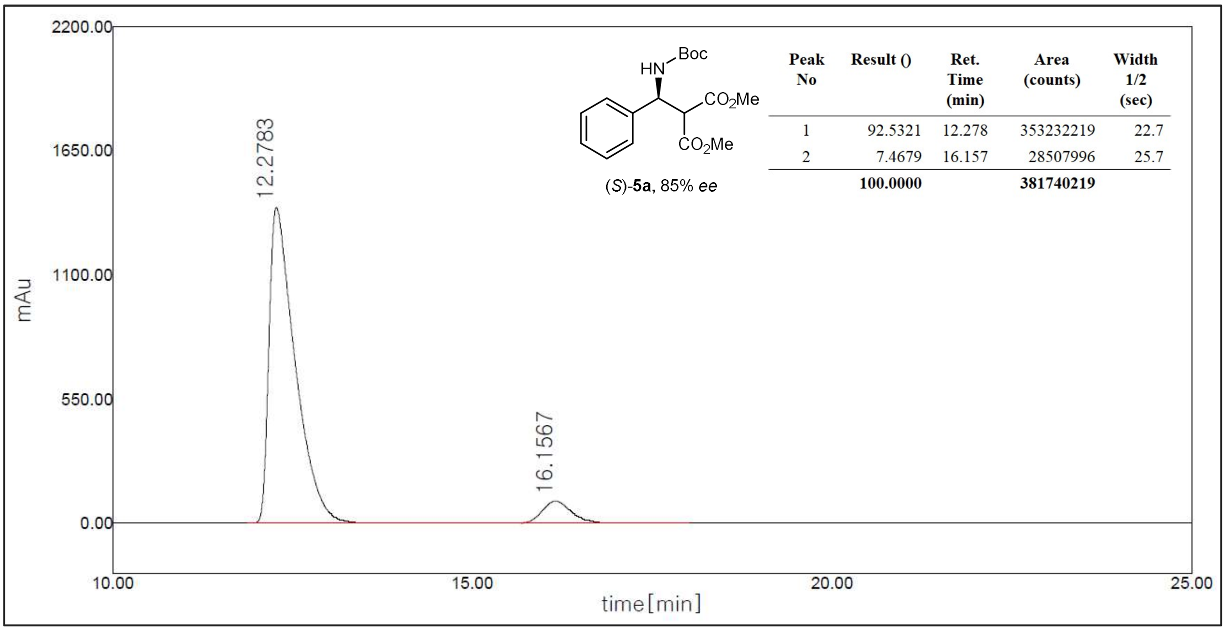

Peak No	Result ()	Ret. Time (min)	Area (counts)	Width 1/2 (sec)
1	92.5321	12.278	353232219	22.7
2	7.4679	16.157	28507996	25.7
	**100.0000**		**381740219**	

Supplementary Figure 117. HPLC spectra of **5a** (**CD-4** (Log *P* = 4.83), on water condition)

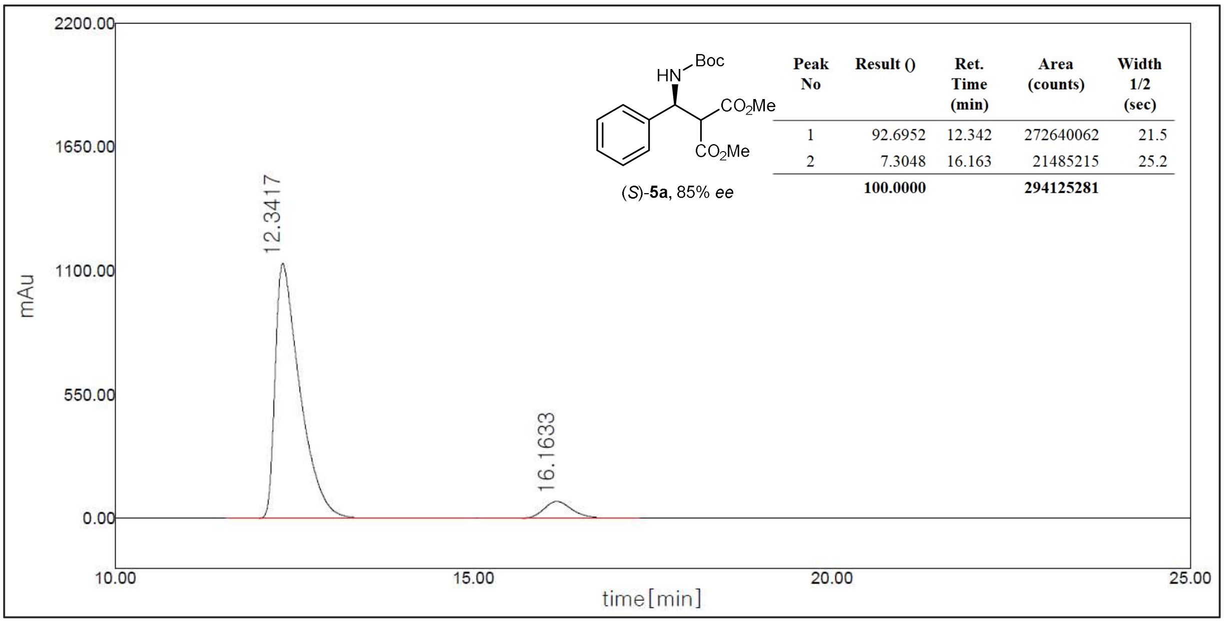

Peak No	Result ()	Ret. Time (min)	Area (counts)	Width 1/2 (sec)
1	92.6952	12.342	272640062	21.5
2	7.3048	16.163	21485215	25.2
	**100.0000**		**294125281**	

Supplementary Figure 118. HPLC spectra of **5a** (**CD-5** (Log *P* = 7.34), on water condition)

With CN-derivatives/in dichloromethane

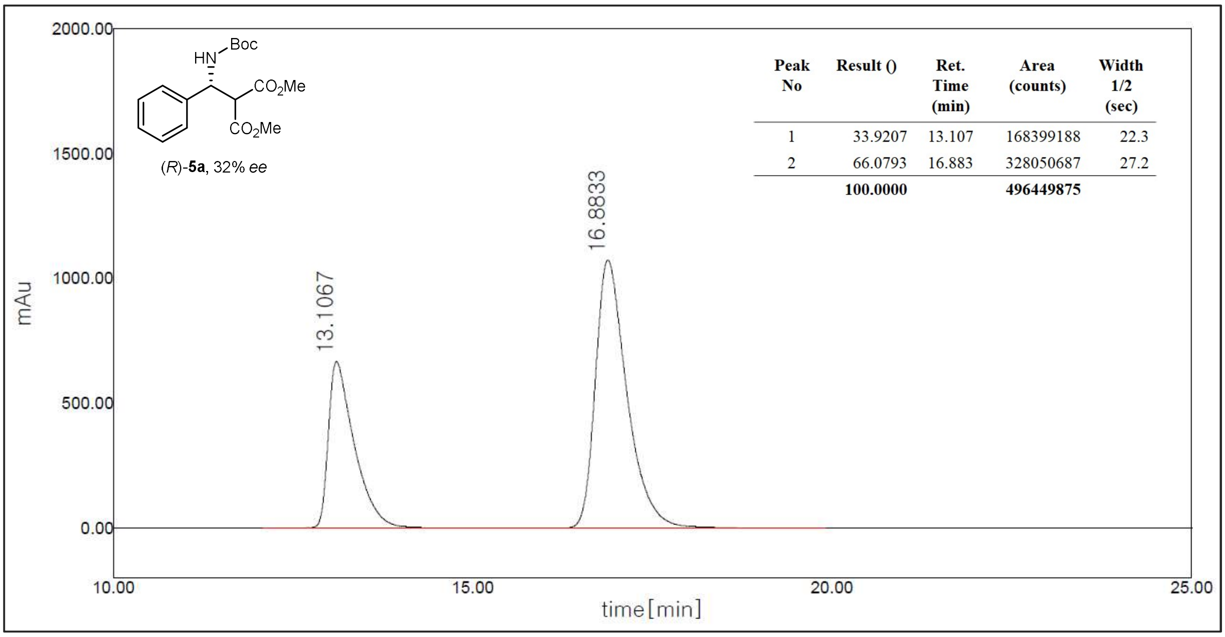

Peak No	Result ()	Ret. Time (min)	Area (counts)	Width 1/2 (sec)
1	33.9207	13.107	168399188	22.3
2	66.0793	16.883	328050687	27.2
	**100.0000**		**496449875**	

Supplementary Figure 119. HPLC spectra of **5a** (**CN-1** (Log *P* = 2.60), in dichloromethane)

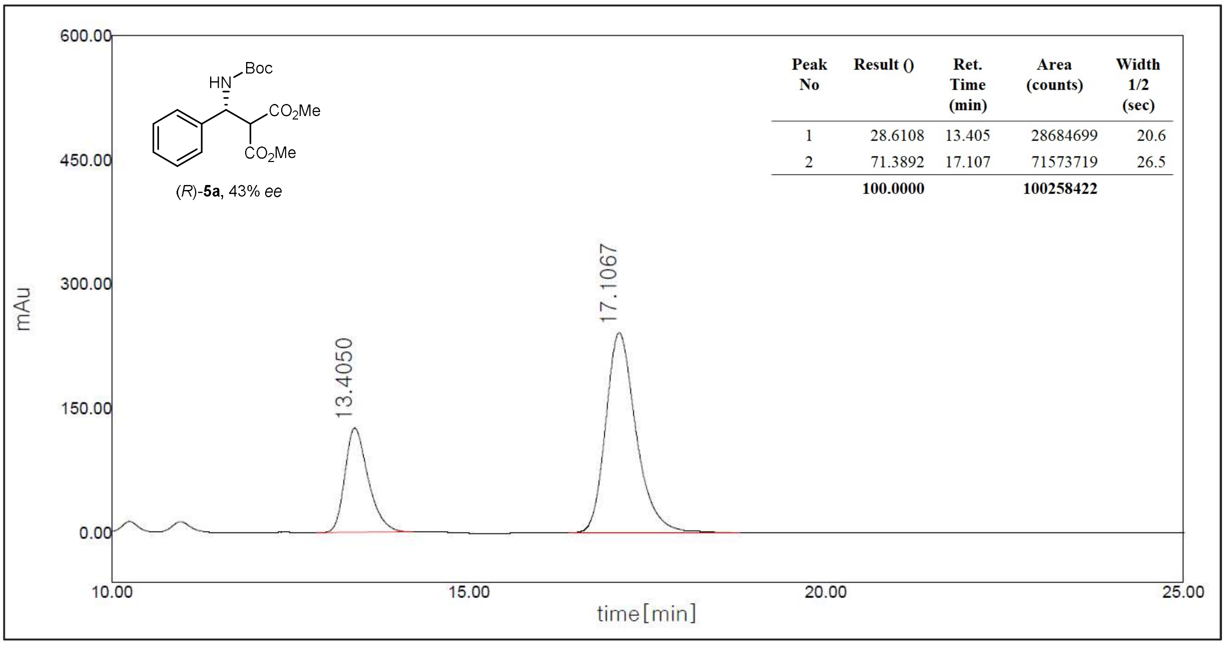

Peak No	Result ()	Ret. Time (min)	Area (counts)	Width 1/2 (sec)
1	28.6108	13.405	28684699	20.6
2	71.3892	17.107	71573719	26.5
	**100.0000**		**100258422**	

Supplementary Figure 120. HPLC spectra of **5a** (**CN-2** (Log *P* = 4.16), in dichloromethane)

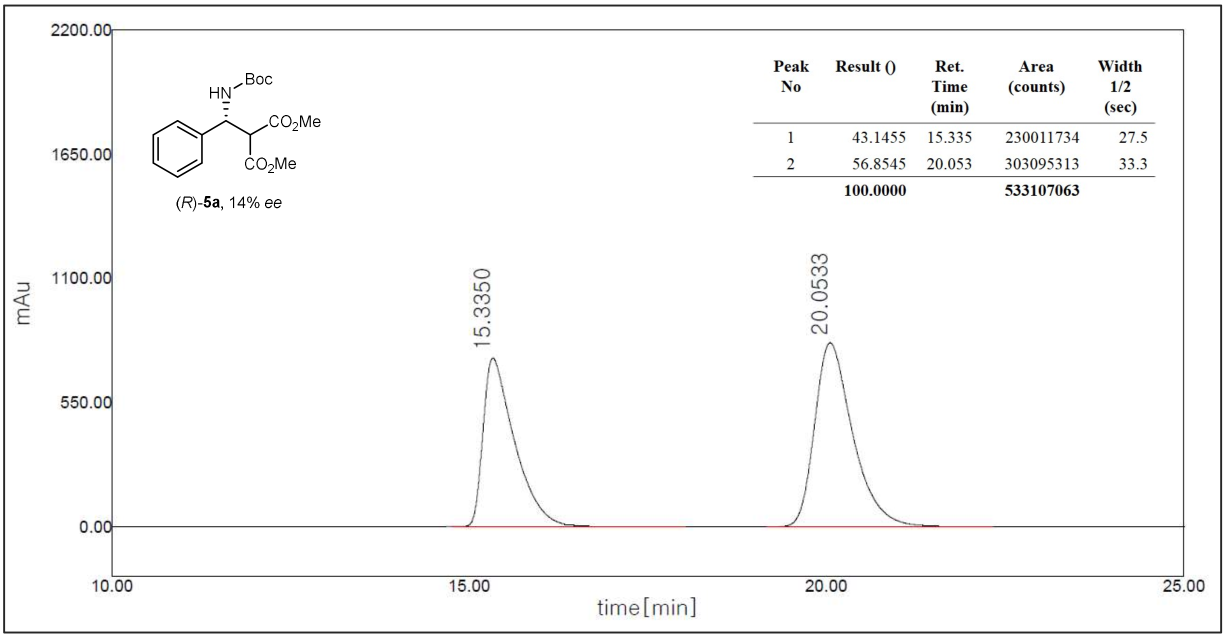

Peak No	Result ()	Ret. Time (min)	Area (counts)	Width 1/2 (sec)
1	43.1455	15.335	230011734	27.5
2	56.8545	20.053	303095313	33.3
	**100.0000**		**533107063**	

Supplementary Figure 121. HPLC spectra of **5a** (**CN-3** (Log *P* = 4.48), in dichloromethane)

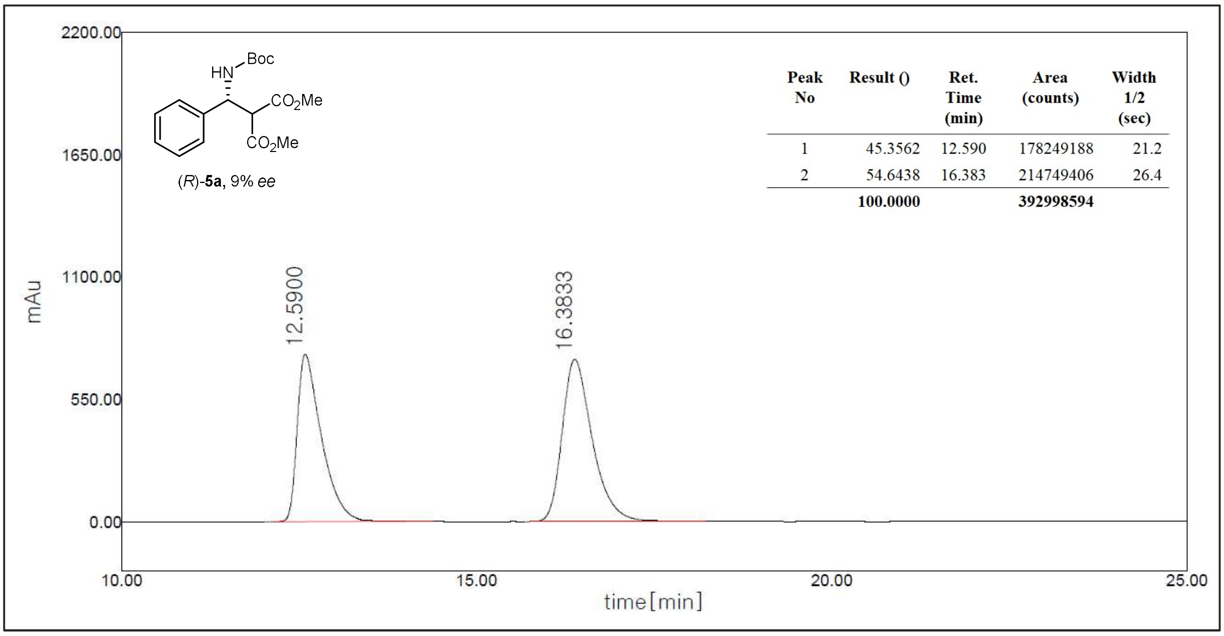

Supplementary Figure 122. HPLC spectra of **5a** (**CN-4** (Log *P* = 4.83), in dichloromethane)

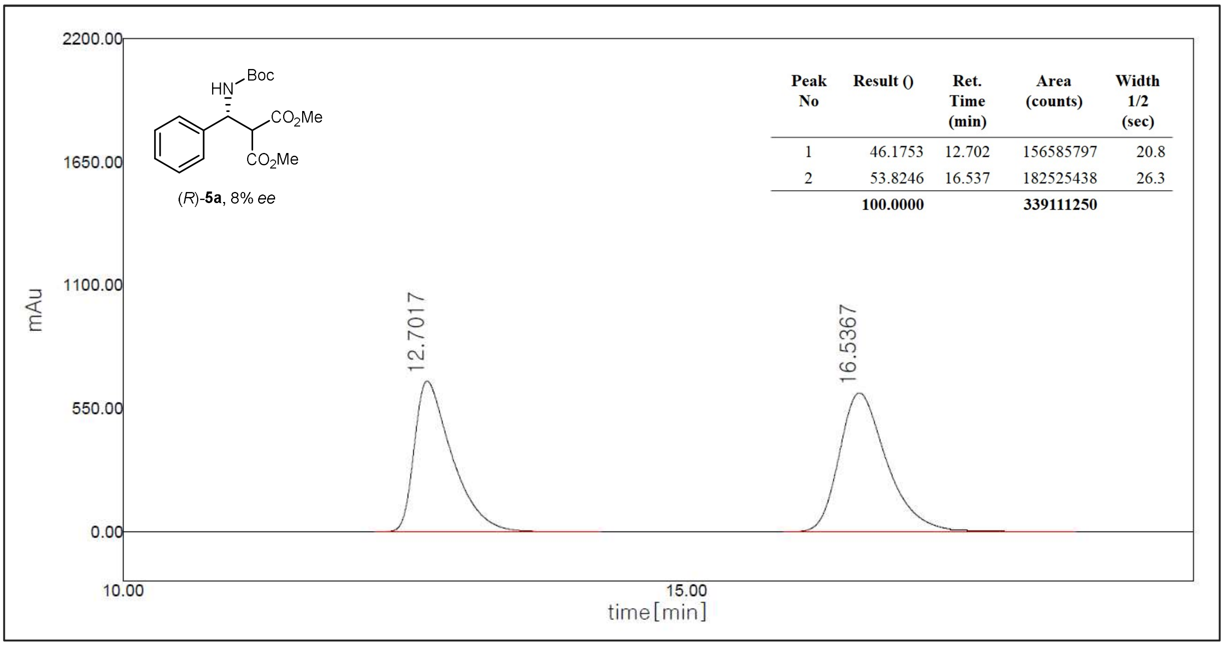

Supplementary Figure 123. HPLC spectra of **5a** (**CN-5** (Log *P* = 7.34), in dichloromethane)

With CD-derivatives/in dichloromethane

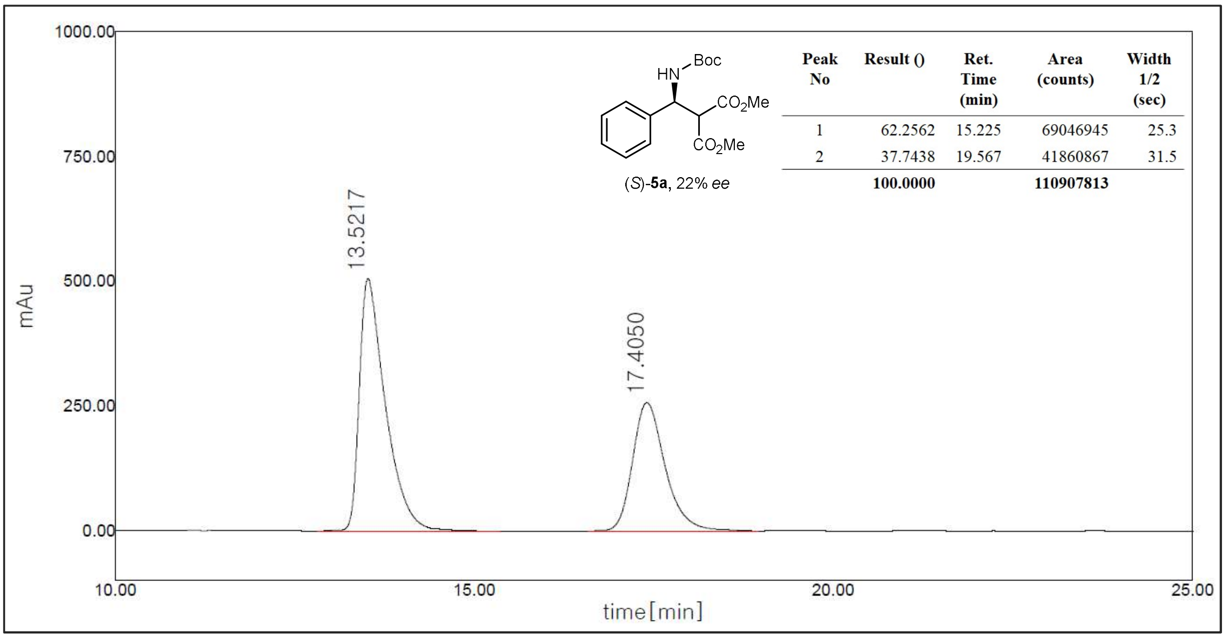

Peak No	Result ()	Ret. Time (min)	Area (counts)	Width 1/2 (sec)
1	62.2562	15.225	69046945	25.3
2	37.7438	19.567	41860867	31.5
	**100.0000**		**110907813**	

Supplementary Figure 124. HPLC spectra of **5a** (**CD-1** (Log *P* = 2.60), in dichloromethane)

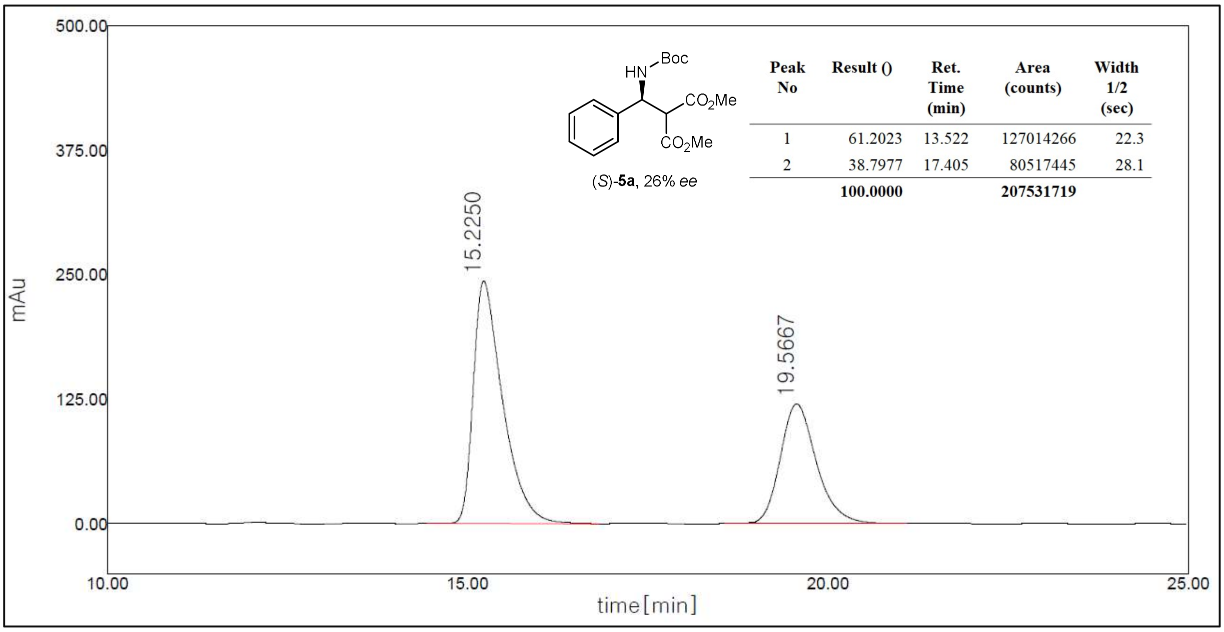

Peak No	Result ()	Ret. Time (min)	Area (counts)	Width 1/2 (sec)
1	61.2023	13.522	127014266	22.3
2	38.7977	17.405	80517445	28.1
	**100.0000**		**207531719**	

Supplementary Figure 125. HPLC spectra of **5a** (**CD-2** (Log *P* = 4.16), in dichloromethane)

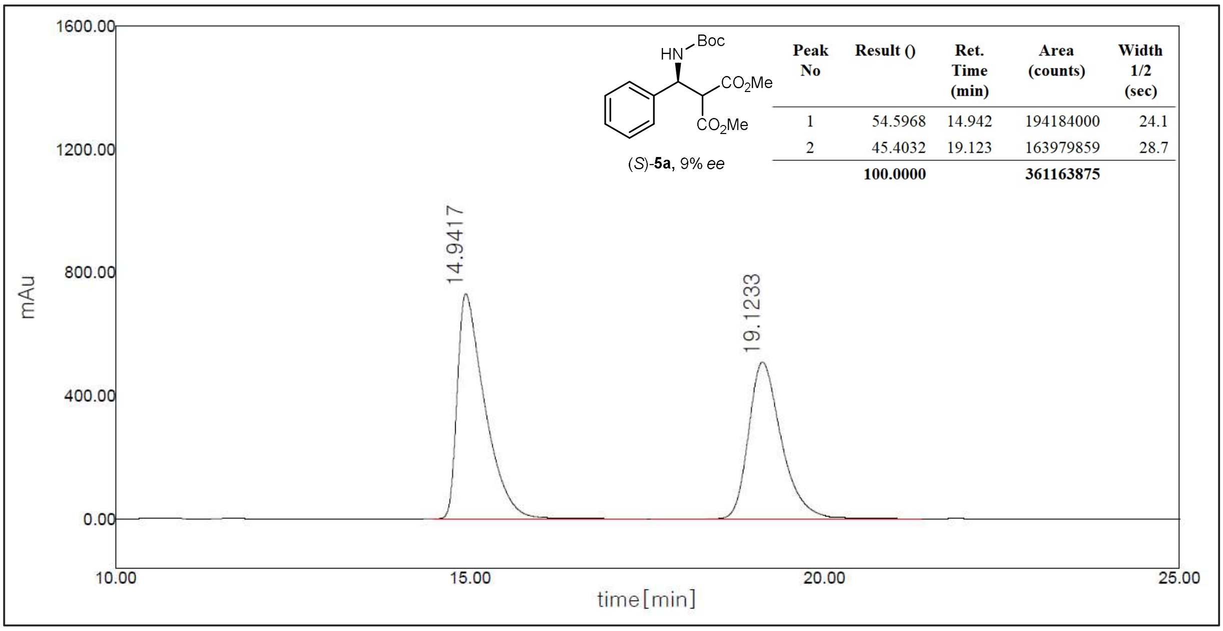

Supplementary Figure 126. HPLC spectra of **5a** (**CD-3** (Log *P* = 4.48), in dichloromethane)

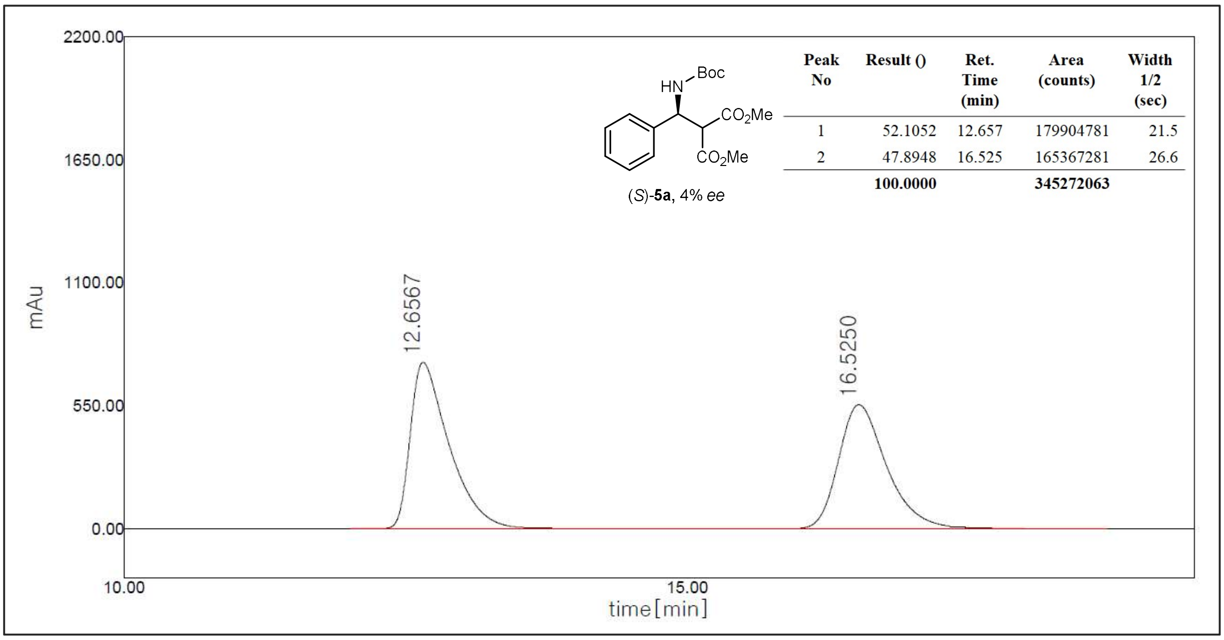

Supplementary Figure 127. HPLC spectra of **5a** (**CD-4** (Log *P* = 4.83), in dichloromethane)

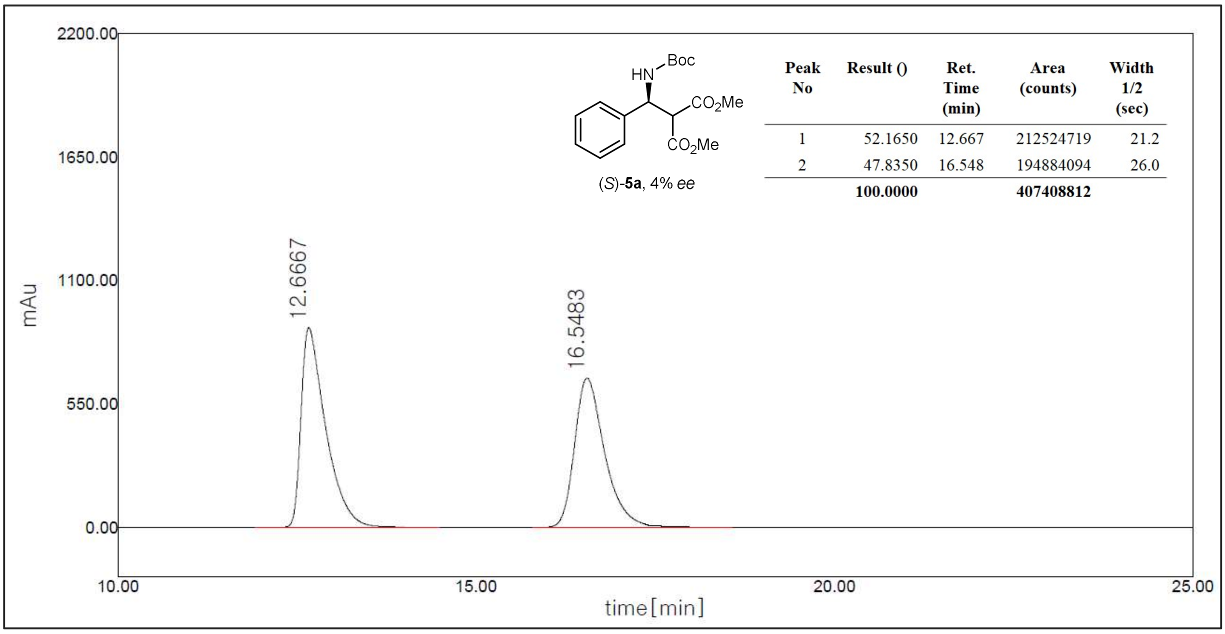

Peak No	Result ()	Ret. Time (min)	Area (counts)	Width 1/2 (sec)
1	52.1650	12.667	212524719	21.2
2	47.8350	16.548	194884094	26.0
	**100.0000**		**407408812**	

Supplementary Figure 128. HPLC spectra of **5a** (**CD-5** (Log P = 7.34), in dichloromethane)

HPLC spectra of Fig. 2c

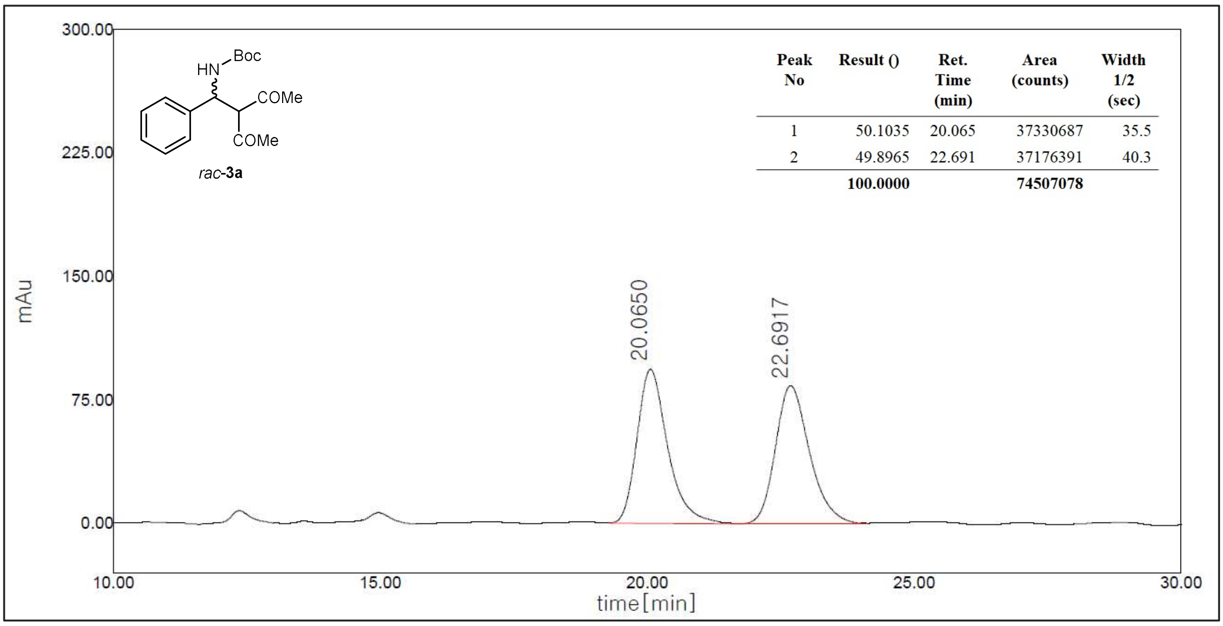

Peak No	Result ()	Ret. Time (min)	Area (counts)	Width 1/2 (sec)
1	50.1035	20.065	37330687	35.5
2	49.8965	22.691	37176391	40.3
	**100.0000**		**74507078**	

Supplementary Figure 129. HPLC spectra of *rac*-**3a**

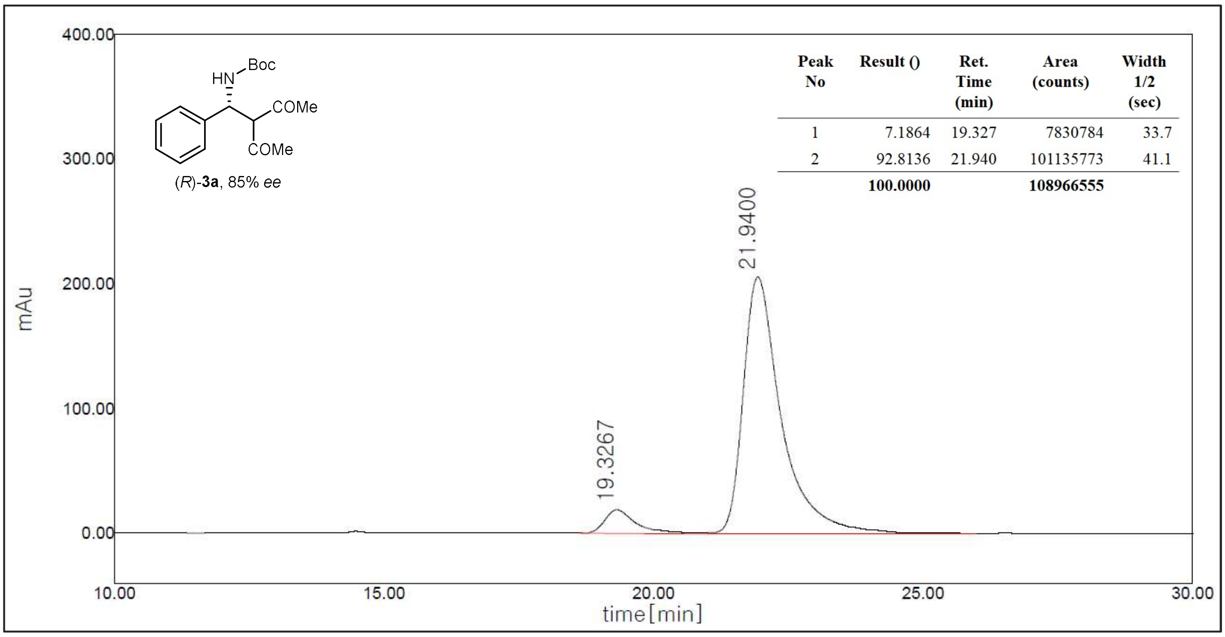

Peak No	Result ()	Ret. Time (min)	Area (counts)	Width 1/2 (sec)
1	7.1864	19.327	7830784	33.7
2	92.8136	21.940	101135773	41.1
	**100.0000**		**108966555**	

Supplementary Figure 130. HPLC spectra of **3a** (200 rpm)

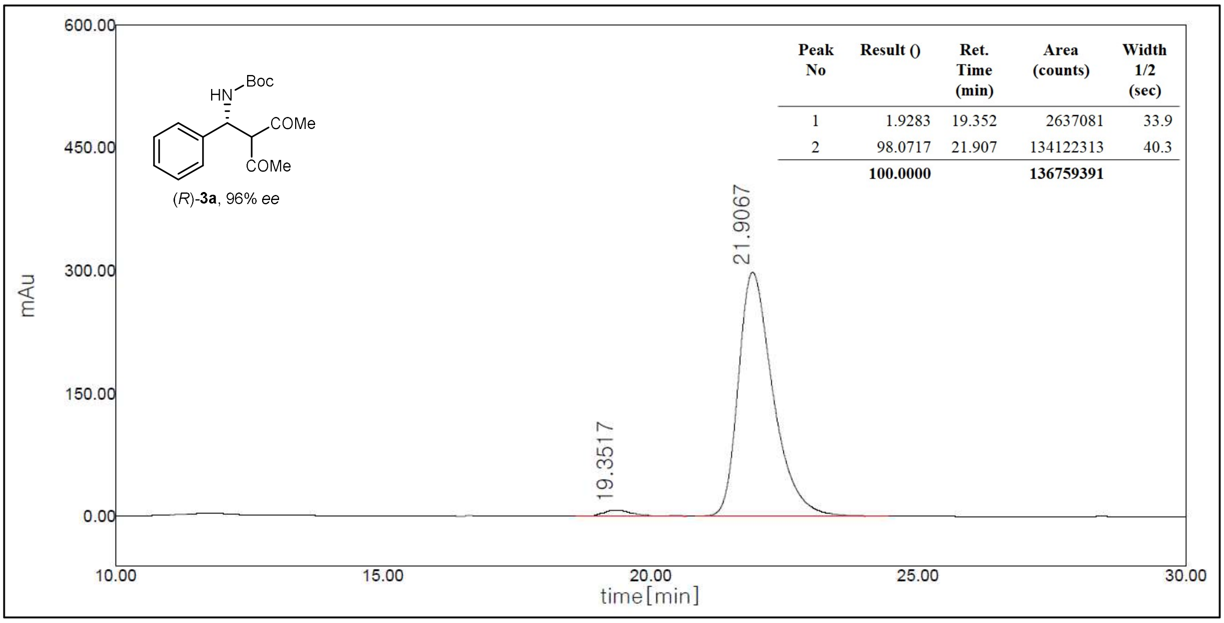

Peak No	Result ()	Ret. Time (min)	Area (counts)	Width 1/2 (sec)
1	1.9283	19.352	2637081	33.9
2	98.0717	21.907	134122313	40.3
	**100.0000**		**136759391**	

Supplementary Figure 131. HPLC spectra of **3a** (600 rpm)

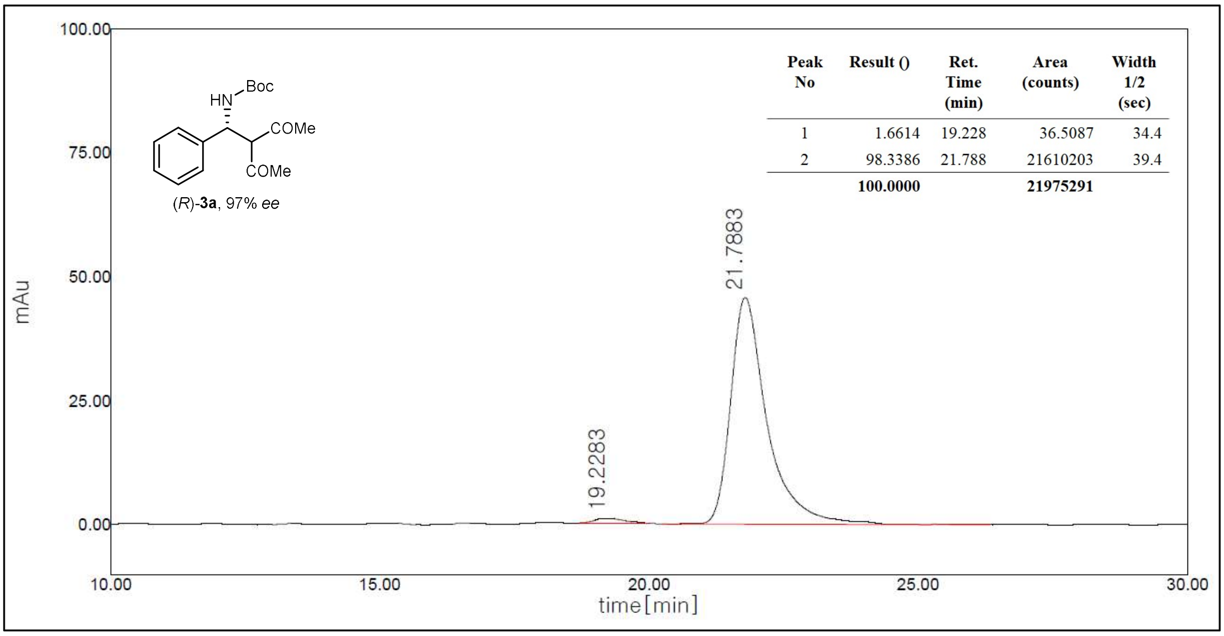

Peak No	Result ()	Ret. Time (min)	Area (counts)	Width 1/2 (sec)
1	1.6614	19.228	36.5087	34.4
2	98.3386	21.788	21610203	39.4
	**100.0000**		**21975291**	

Supplementary Figure 132. HPLC spectra of **3a** (1150 rpm)

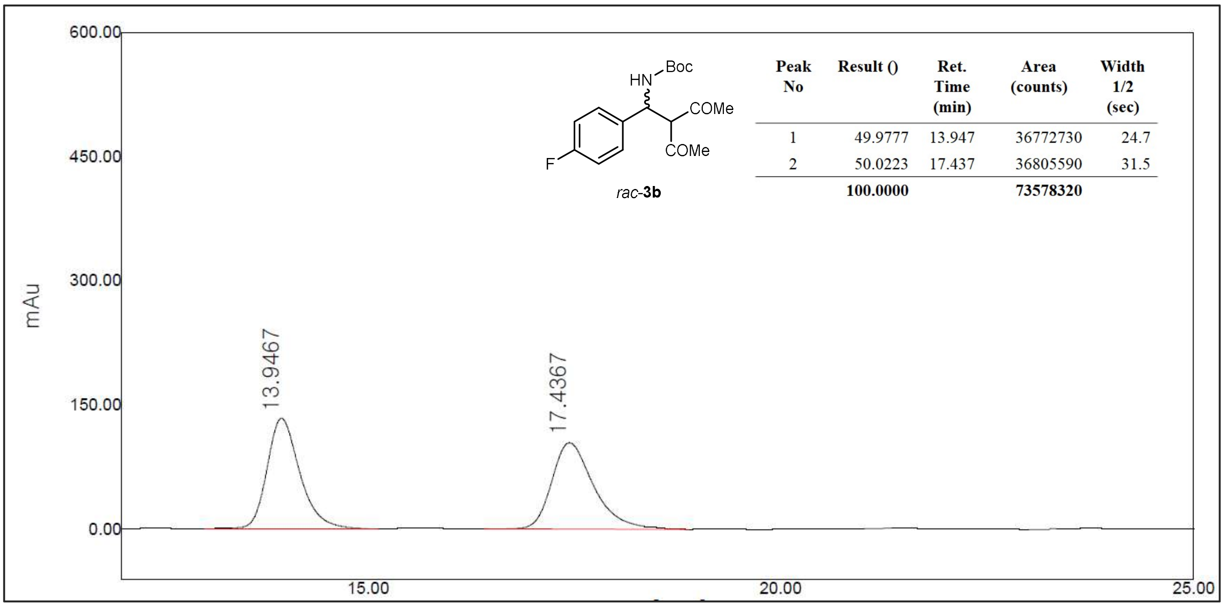

Peak No	Result ()	Ret. Time (min)	Area (counts)	Width 1/2 (sec)
1	49.9777	13.947	36772730	24.7
2	50.0223	17.437	36805590	31.5
	**100.0000**		**73578320**	

Supplementary Figure 133. HPLC spectra of *rac*-**3b**

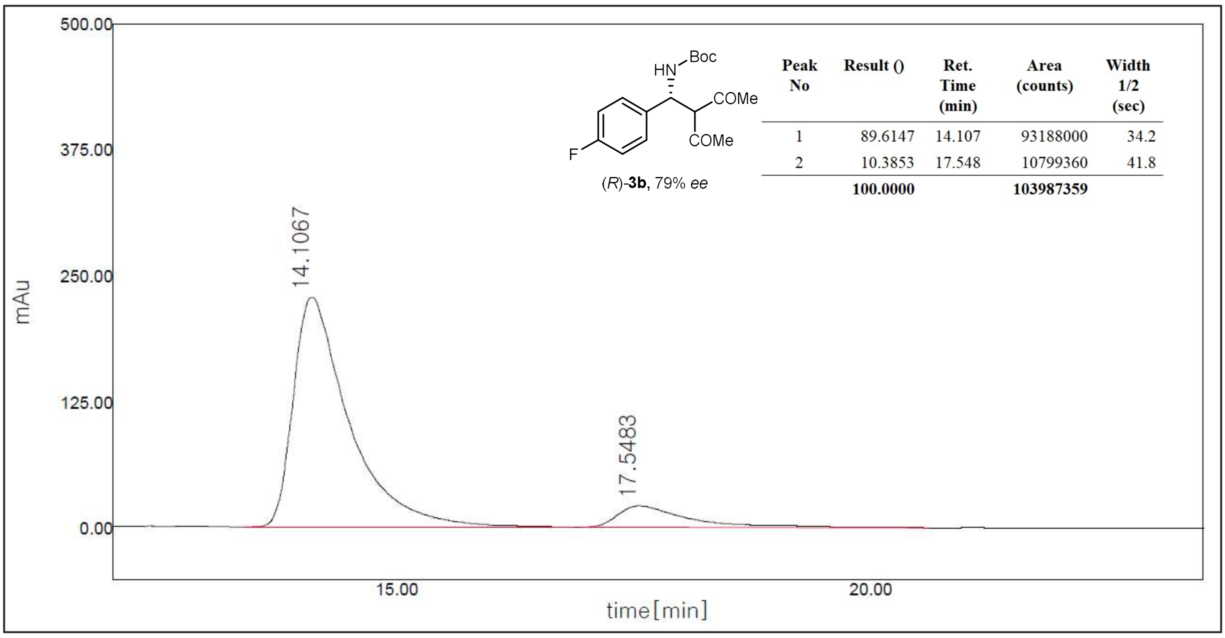

Peak No	Result ()	Ret. Time (min)	Area (counts)	Width 1/2 (sec)
1	89.6147	14.107	93188000	34.2
2	10.3853	17.548	10799360	41.8
	**100.0000**		**103987359**	

Supplementary Figure 134. HPLC spectra of **3b** (200 rpm)

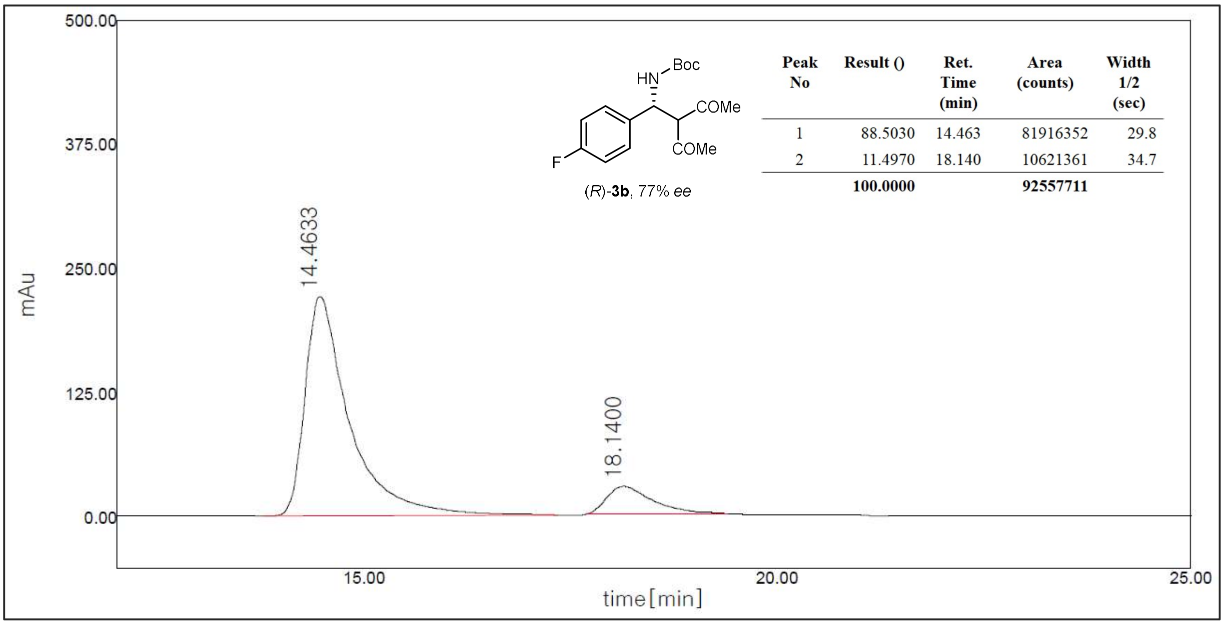

Peak No	Result ()	Ret. Time (min)	Area (counts)	Width 1/2 (sec)
1	88.5030	14.463	81916352	29.8
2	11.4970	18.140	10621361	34.7
	**100.0000**		**92557711**	

Supplementary Figure 135. HPLC spectra of **3b** (600 rpm)

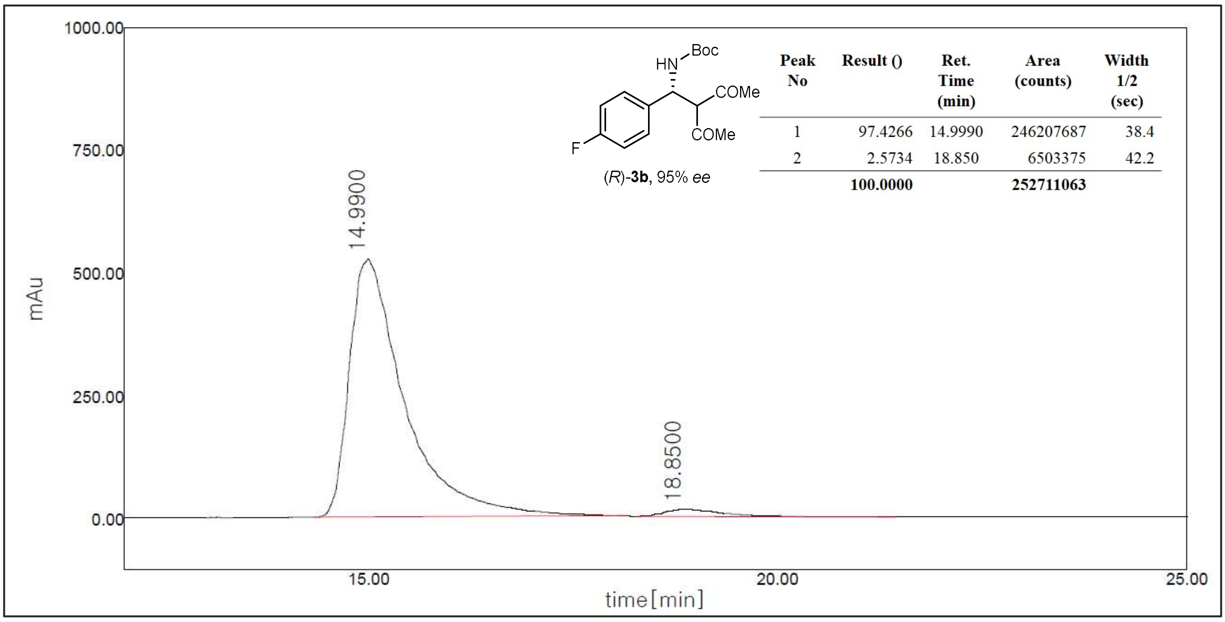

Peak No	Result ()	Ret. Time (min)	Area (counts)	Width 1/2 (sec)
1	97.4266	14.9990	246207687	38.4
2	2.5734	18.850	6503375	42.2
	**100.0000**		**252711063**	

Supplementary Figure 136. HPLC spectra of **3b** (1150 rpm)

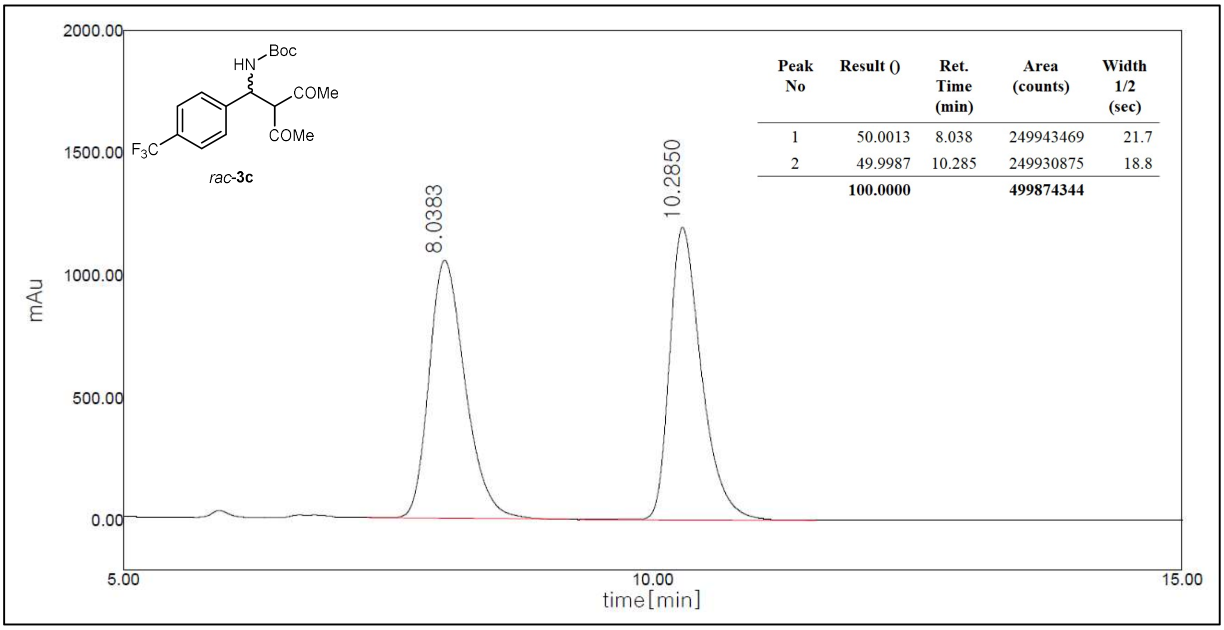

Peak No	Result ()	Ret. Time (min)	Area (counts)	Width 1/2 (sec)
1	50.0013	8.038	249943469	21.7
2	49.9987	10.285	249930875	18.8
	**100.0000**		**499874344**	

Supplementary Figure 137. HPLC spectra of *rac*-**3c**

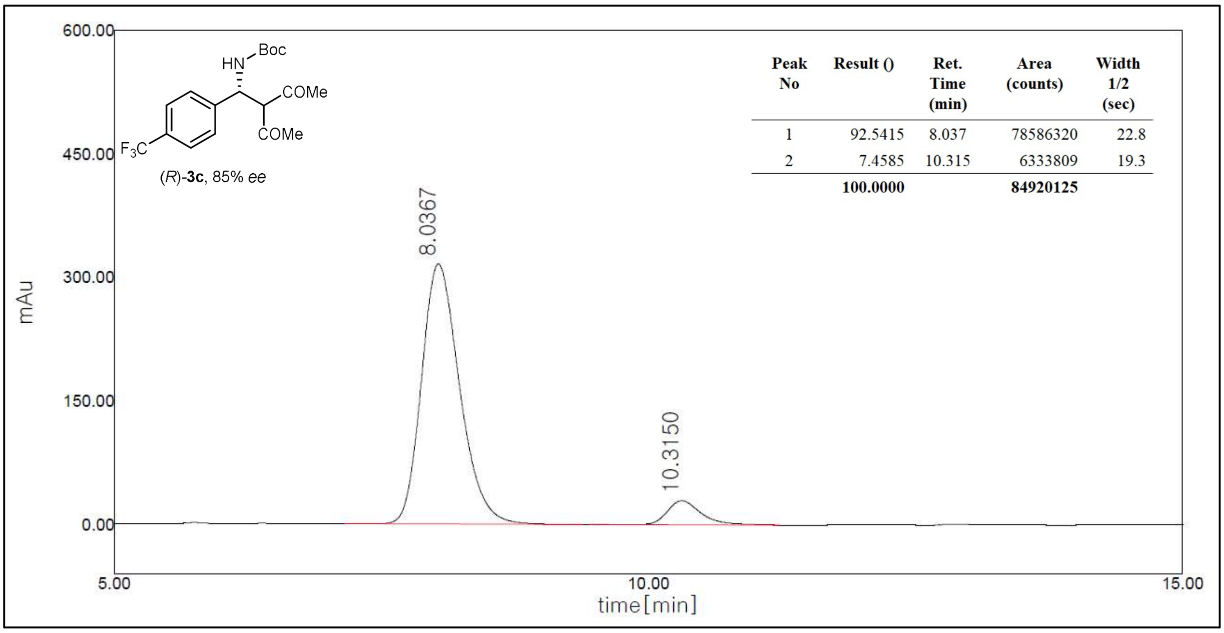

Peak No	Result ()	Ret. Time (min)	Area (counts)	Width 1/2 (sec)
1	92.5415	8.037	78586320	22.8
2	7.4585	10.315	6333809	19.3
	**100.0000**		**84920125**	

Supplementary Figure 138. HPLC spectra of **3c** (200 rpm)

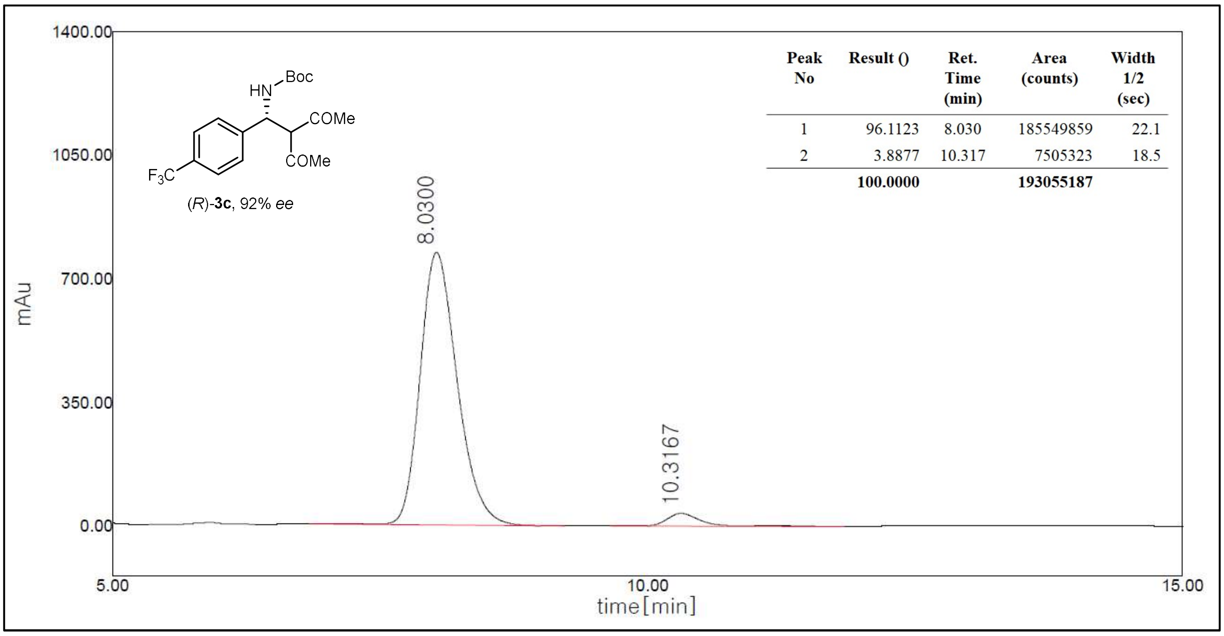

Supplementary Figure 139. HPLC spectra of **3c** (600 rpm)

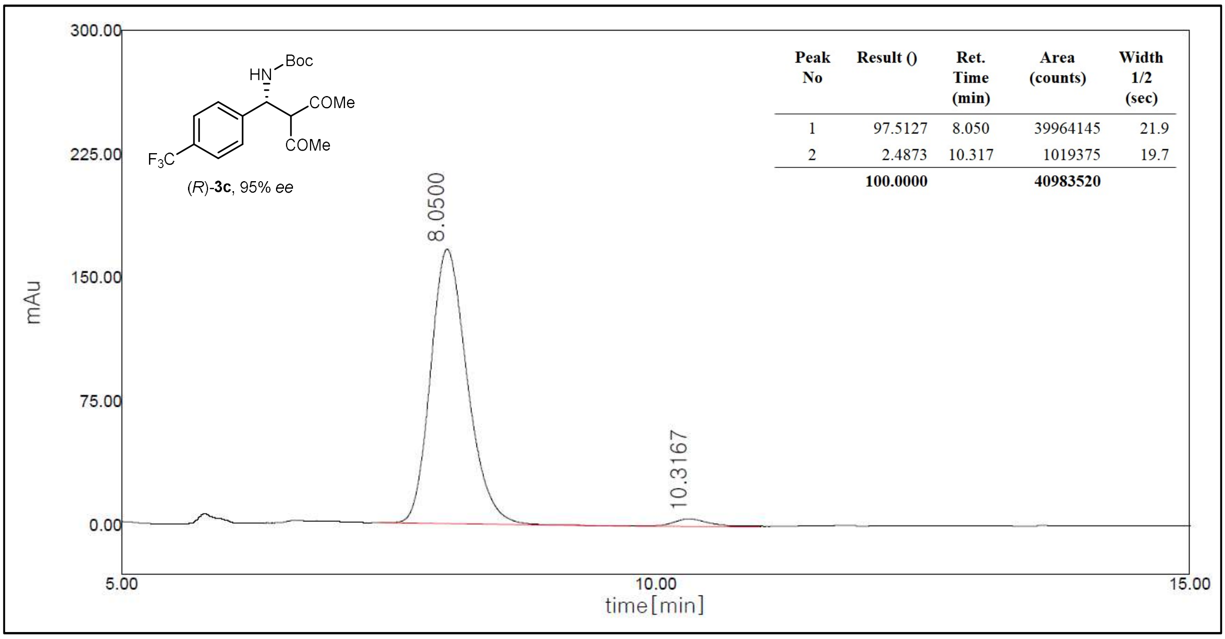

Supplementary Figure 140. HPLC spectra of **3c** (1150 rpm)

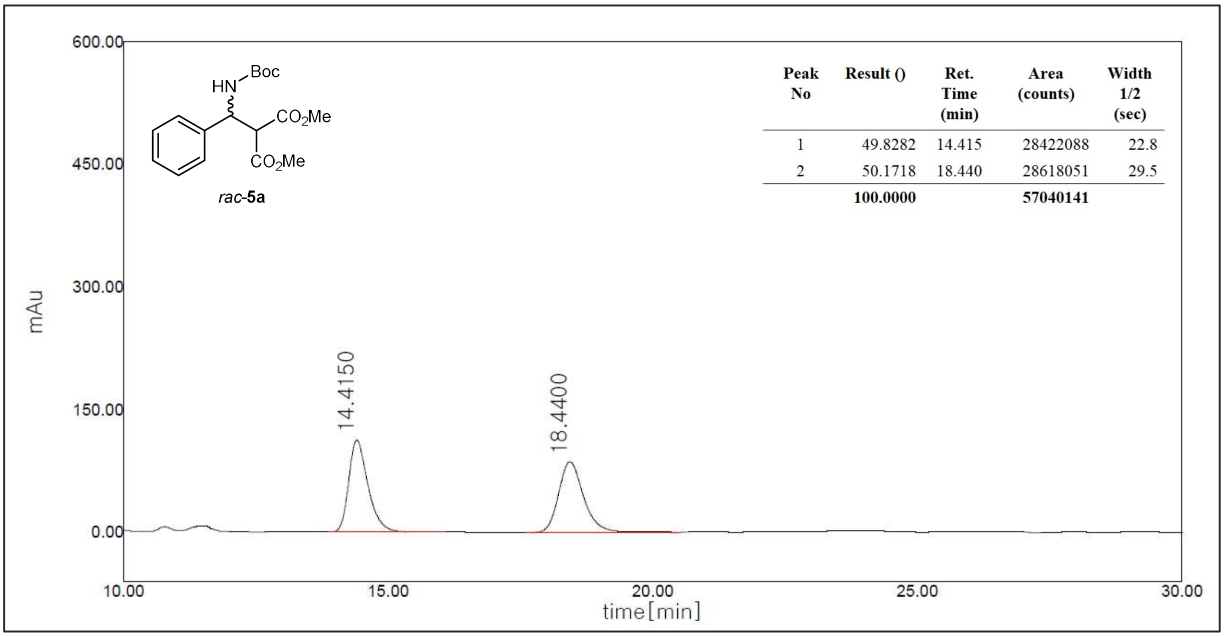

Peak No	Result ()	Ret. Time (min)	Area (counts)	Width 1/2 (sec)
1	49.8282	14.415	28422088	22.8
2	50.1718	18.440	28618051	29.5
	**100.0000**		**57040141**	

Supplementary Figure 141. HPLC spectra of *rac*-**5a**

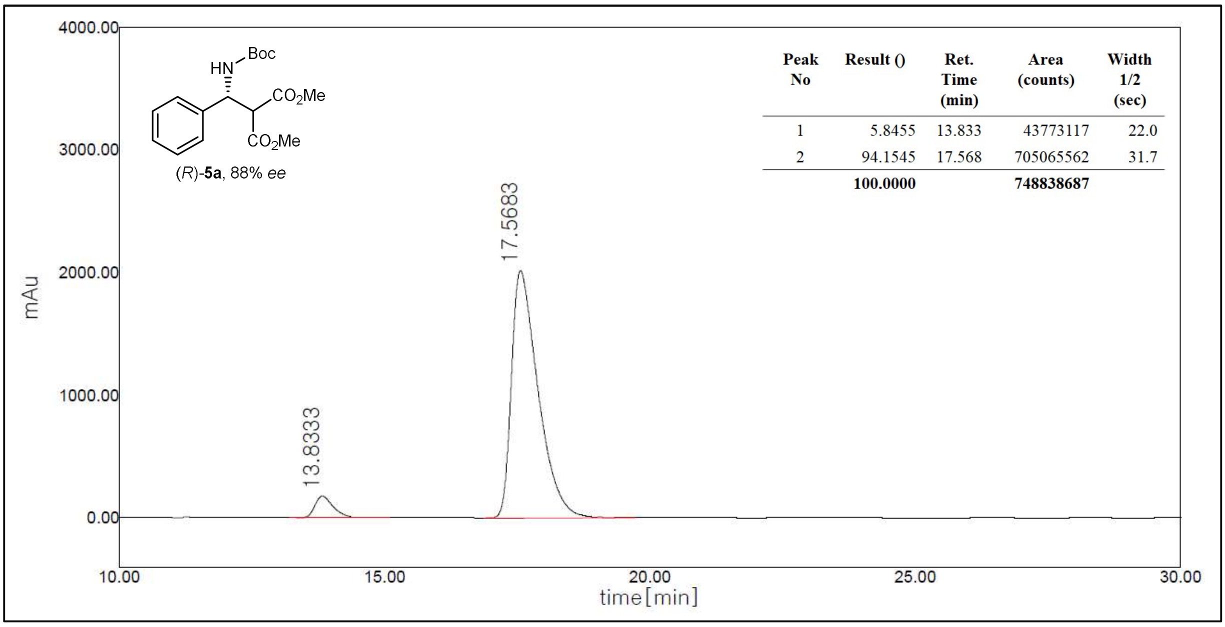

Peak No	Result ()	Ret. Time (min)	Area (counts)	Width 1/2 (sec)
1	5.8455	13.833	43773117	22.0
2	94.1545	17.568	705065562	31.7
	**100.0000**		**748838687**	

Supplementary Figure 142. HPLC spectra of **5a** (200 rpm)

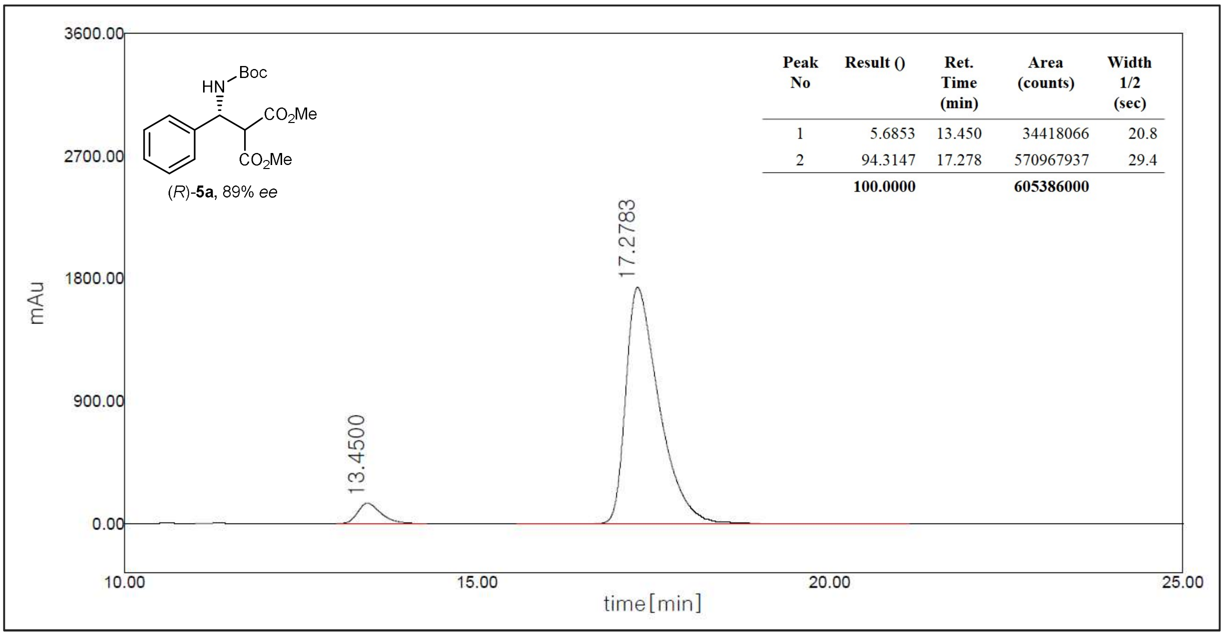

Supplementary Figure 143. HPLC spectra of **5a** (600 rpm)

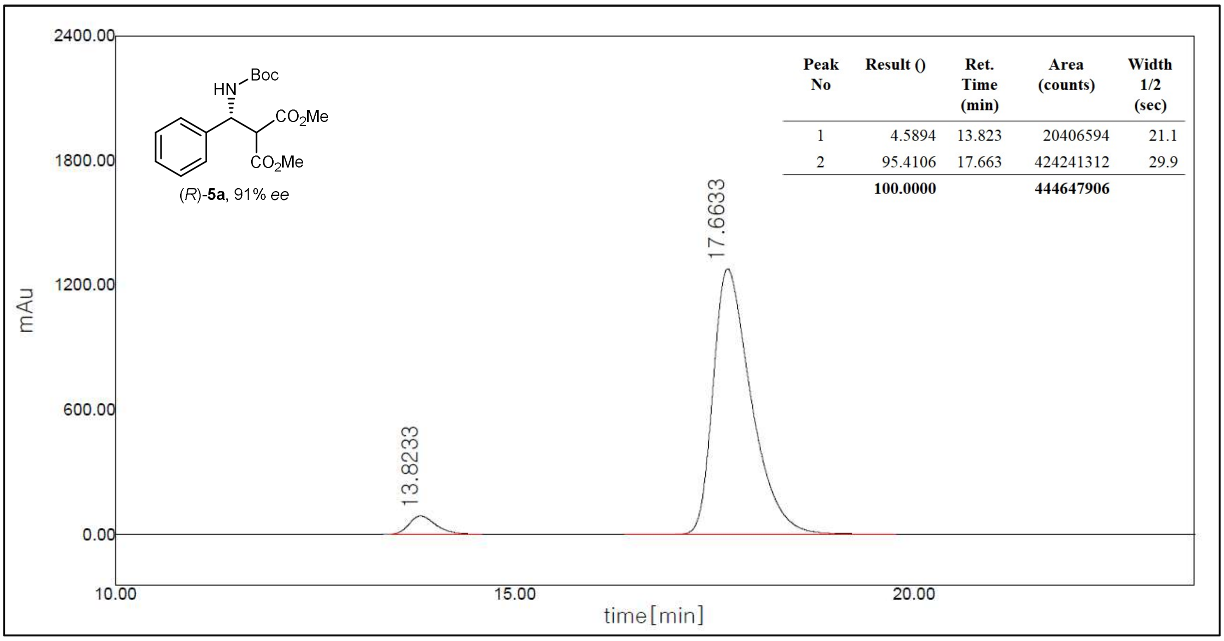

Supplementary Figure 144. HPLC spectra of **5a** (1150 rpm)

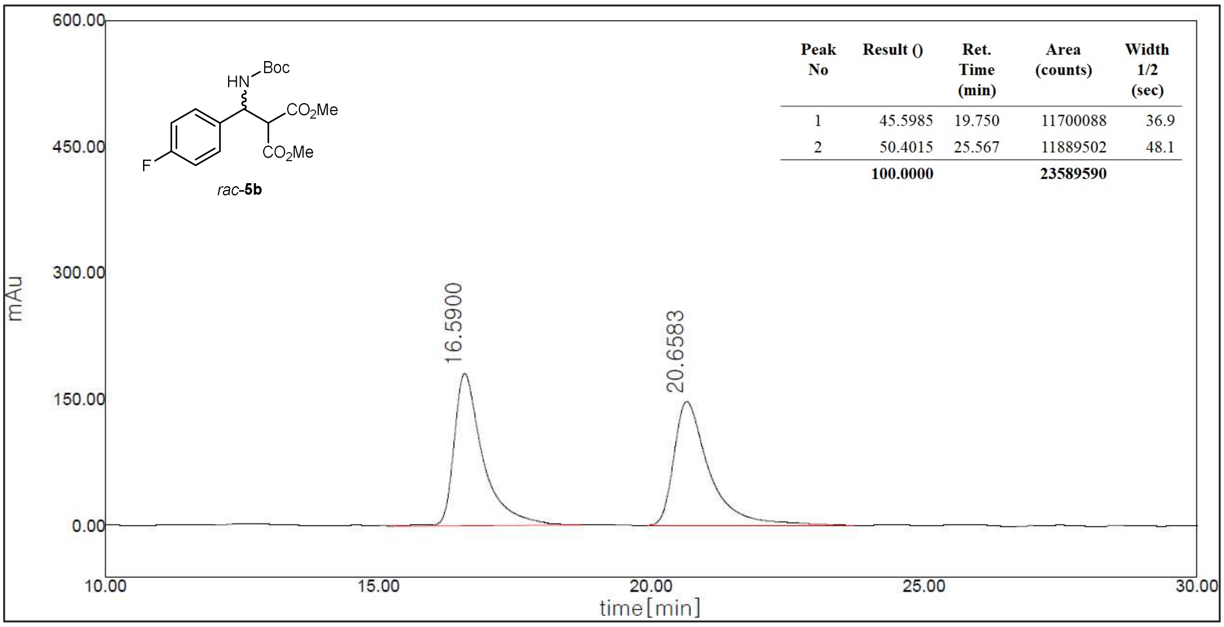

Peak No	Result ()	Ret. Time (min)	Area (counts)	Width 1/2 (sec)
1	45.5985	19.750	11700088	36.9
2	50.4015	25.567	11889502	48.1
	**100.0000**		**23589590**	

Supplementary Figure 145. HPLC spectra of *rac*-**5b**

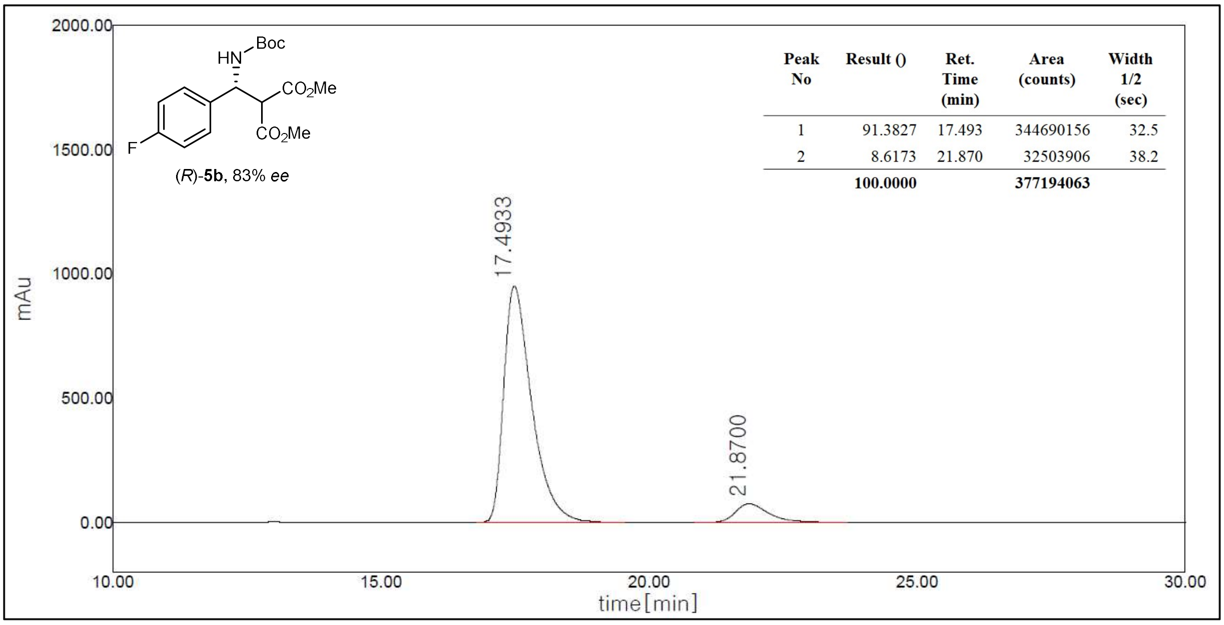

Peak No	Result ()	Ret. Time (min)	Area (counts)	Width 1/2 (sec)
1	91.3827	17.493	344690156	32.5
2	8.6173	21.870	32503906	38.2
	**100.0000**		**377194063**	

Supplementary Figure 146. HPLC spectra of **5b** (200 rpm)

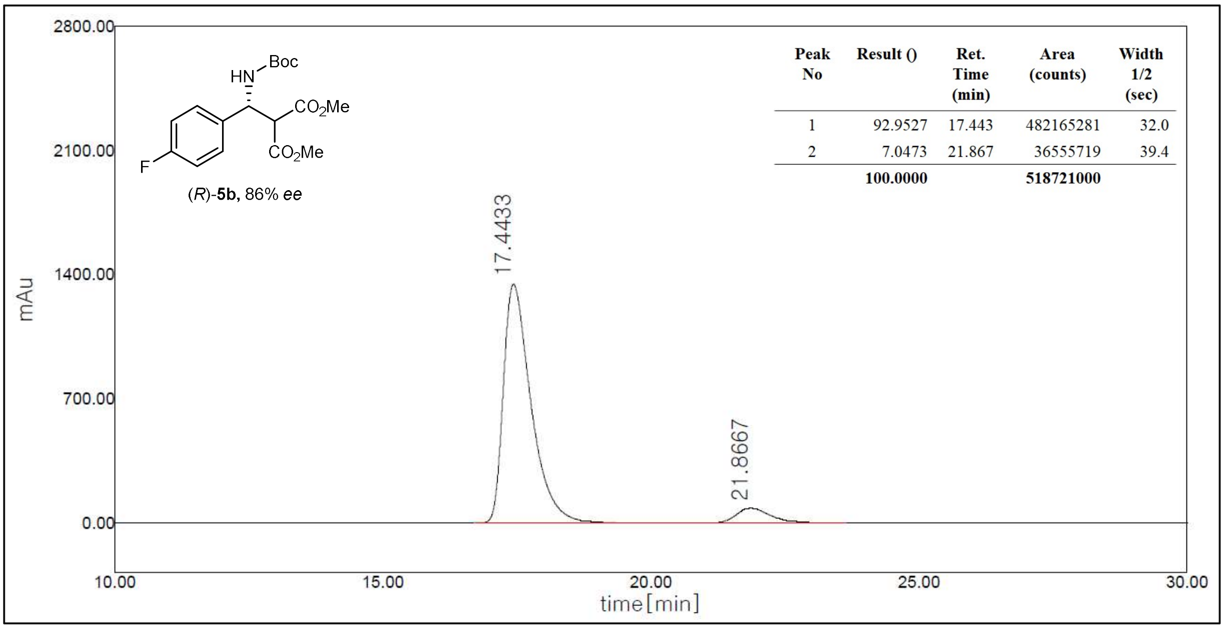

Peak No	Result ()	Ret. Time (min)	Area (counts)	Width 1/2 (sec)
1	92.9527	17.443	482165281	32.0
2	7.0473	21.867	36555719	39.4
	**100.0000**		**518721000**	

Supplementary Figure 147. HPLC spectra of **5b** (600 rpm)

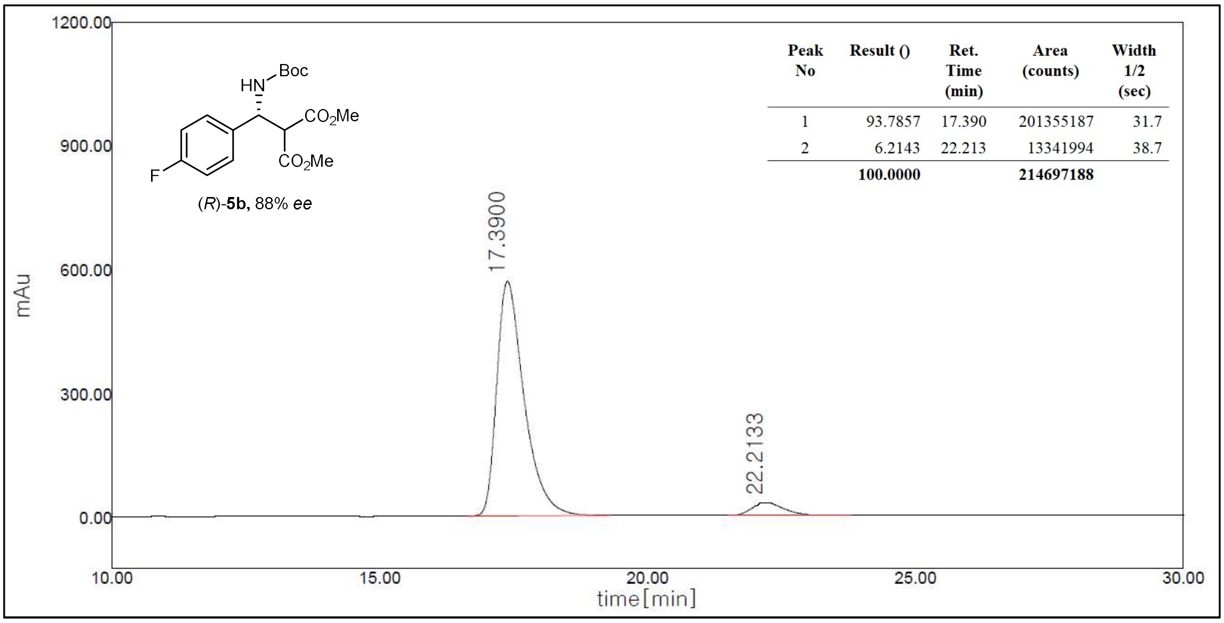

Peak No	Result ()	Ret. Time (min)	Area (counts)	Width 1/2 (sec)
1	93.7857	17.390	201355187	31.7
2	6.2143	22.213	13341994	38.7
	**100.0000**		**214697188**	

Supplementary Figure 148. HPLC spectra of **5b** (1150 rpm)

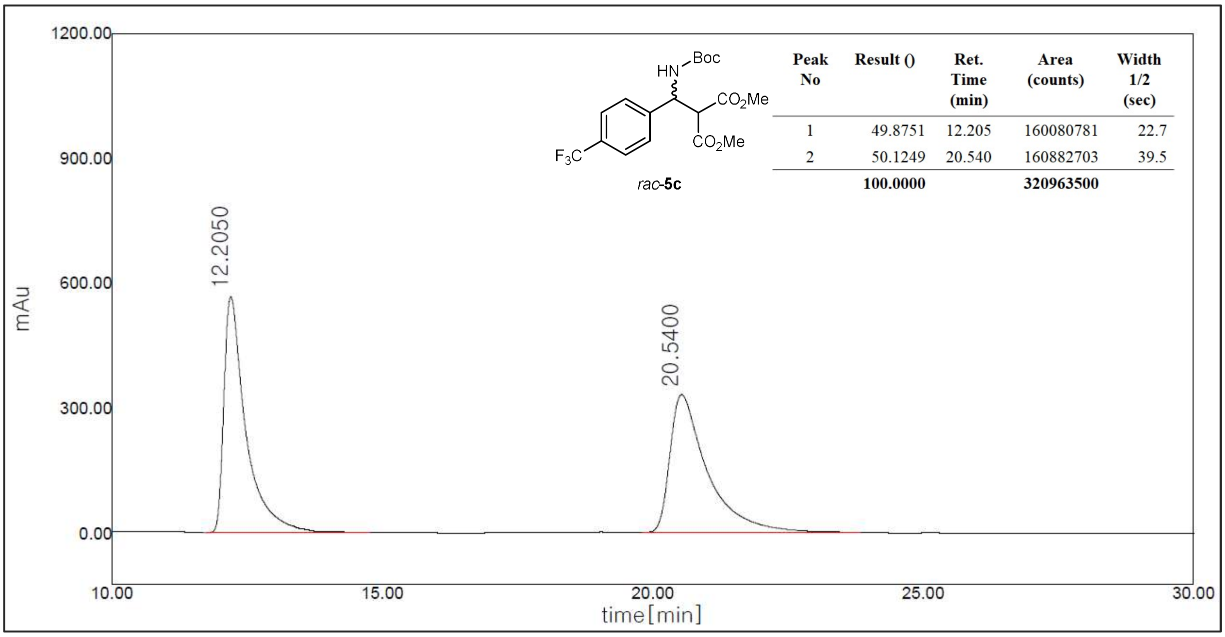

Peak No	Result ()	Ret. Time (min)	Area (counts)	Width 1/2 (sec)
1	49.8751	12.205	160080781	22.7
2	50.1249	20.540	160882703	39.5
	**100.0000**		**320963500**	

Supplementary Figure 149. HPLC spectra of *rac*-**5c**

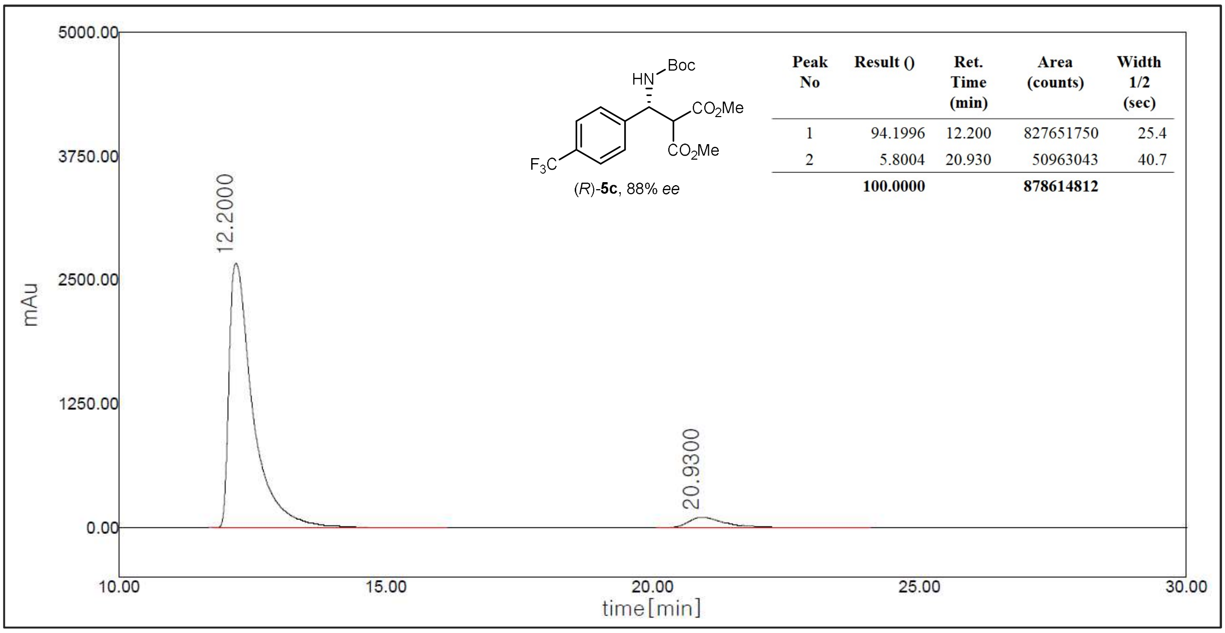

Peak No	Result ()	Ret. Time (min)	Area (counts)	Width 1/2 (sec)
1	94.1996	12.200	827651750	25.4
2	5.8004	20.930	50963043	40.7
	**100.0000**		**878614812**	

Supplementary Figure 150. HPLC spectra of **5c** (200 rpm)

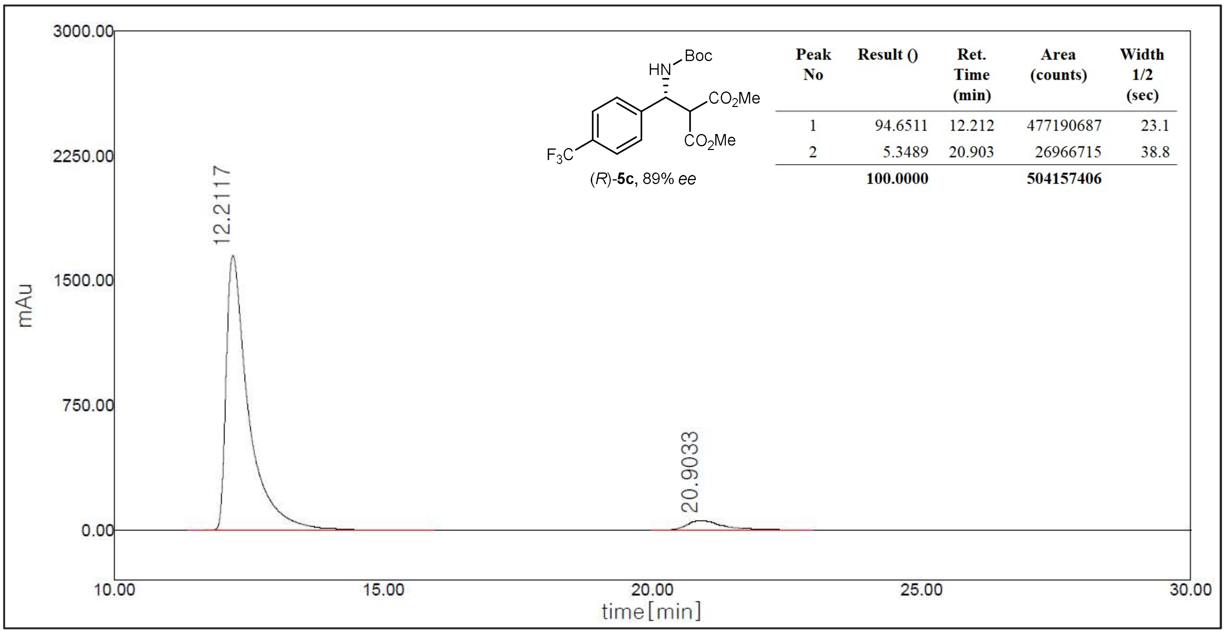

Peak No	Result ()	Ret. Time (min)	Area (counts)	Width 1/2 (sec)
1	94.6511	12.212	477190687	23.1
2	5.3489	20.903	26966715	38.8
	**100.0000**		**504157406**	

Supplementary Figure 151. HPLC spectra of **5c** (600 rpm)

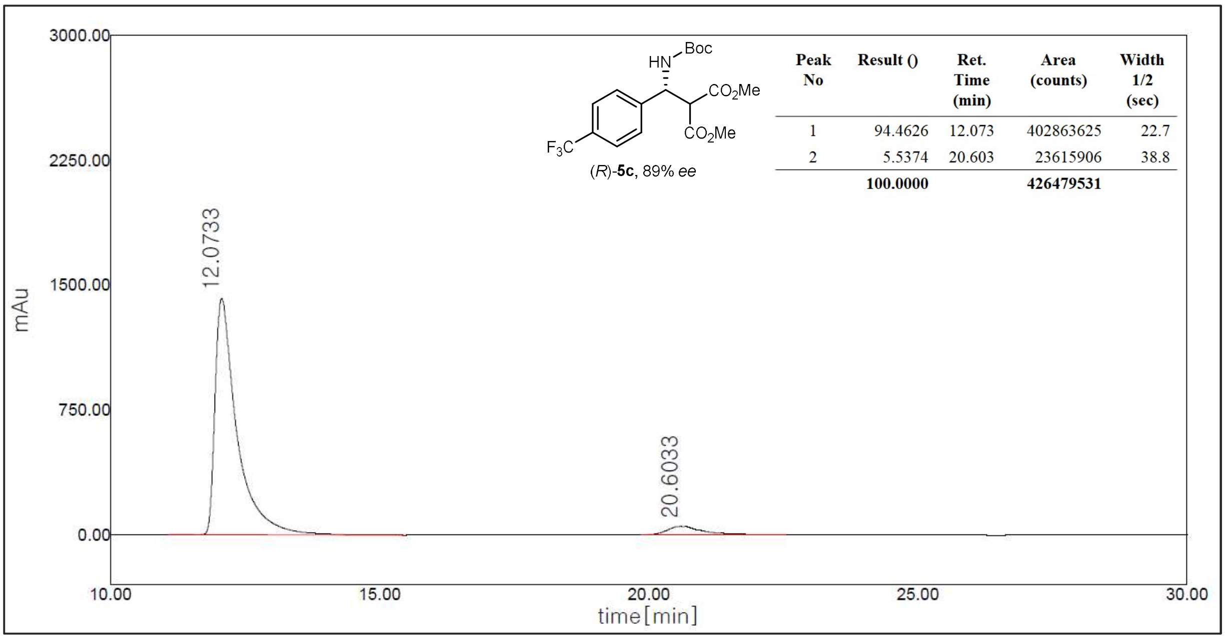

Peak No	Result ()	Ret. Time (min)	Area (counts)	Width 1/2 (sec)
1	94.4626	12.073	402863625	22.7
2	5.5374	20.603	23615906	38.8
	**100.0000**		**426479531**	

Supplementary Figure 152. HPLC spectra of **5c** (1150 rpm)

HPLC spectra of Figure 3c

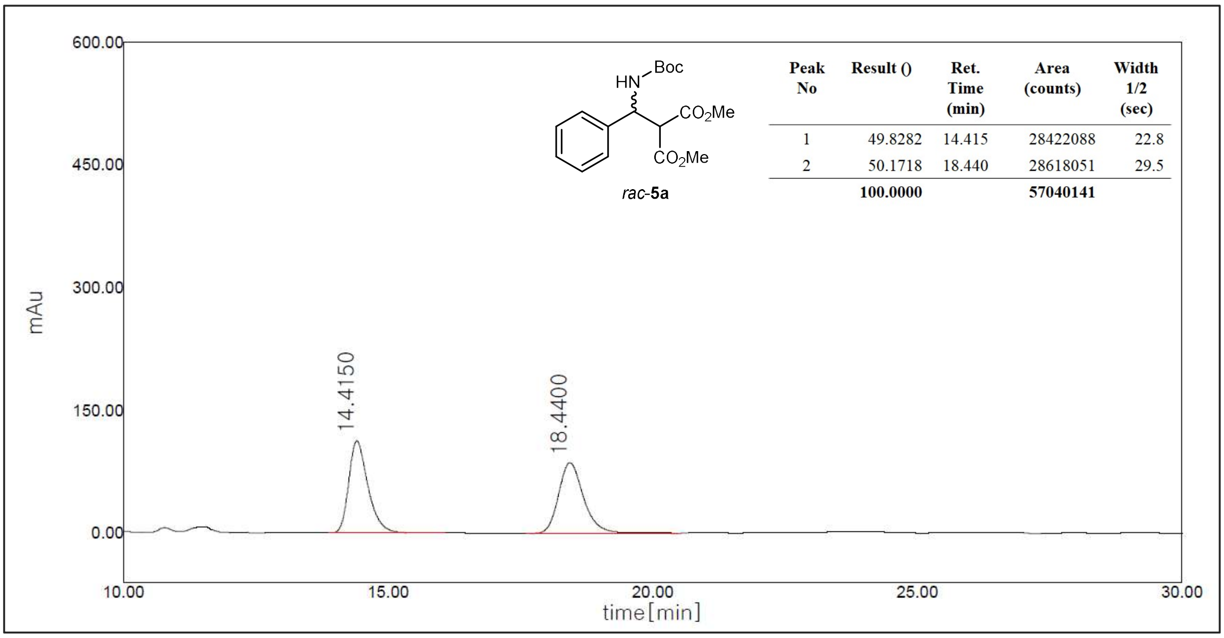

Peak No	Result ()	Ret. Time (min)	Area (counts)	Width 1/2 (sec)
1	49.8282	14.415	28422088	22.8
2	50.1718	18.440	28618051	29.5
	**100.0000**		**57040141**	

Supplementary Figure 153. HPLC spectra of *rac*-**5a**

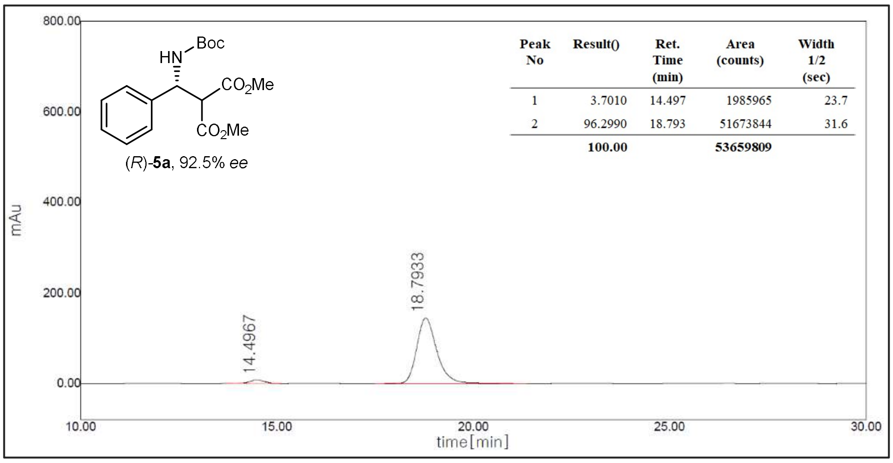

Peak No	Result()	Ret. Time (min)	Area (counts)	Width 1/2 (sec)
1	3.7010	14.497	1985965	23.7
2	96.2990	18.793	51673844	31.6
	**100.00**		**53659809**	

Supplementary Figure 154. HPLC spectra of **5a** (**CN-2**, ID = 250 μm, Qw/Qo = 20:1)

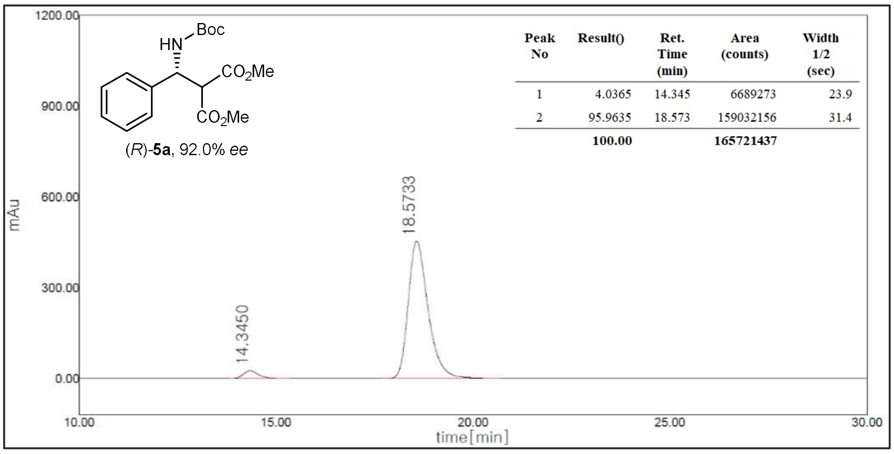

Peak No	Result()	Ret. Time (min)	Area (counts)	Width 1/2 (sec)
1	4.0365	14.345	6689273	23.9
2	95.9635	18.573	159032156	31.4
	**100.00**		**165721437**	

Supplementary Figure 155. HPLC spectra of **5a** (**CN-2**, ID = 250 μm, Qw/Qo = 20:20)

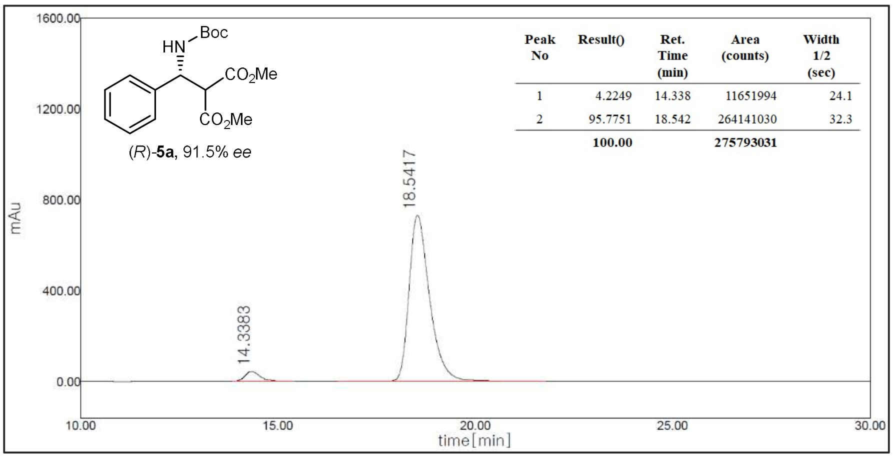

Peak No	Result()	Ret. Time (min)	Area (counts)	Width 1/2 (sec)
1	4.2249	14.338	11651994	24.1
2	95.7751	18.542	264141030	32.3
	**100.00**		**275793031**	

Supplementary Figure 156. HPLC spectra of **5a** (**CN-2**, ID = 250 μm, Qw/Qo = 1:20)

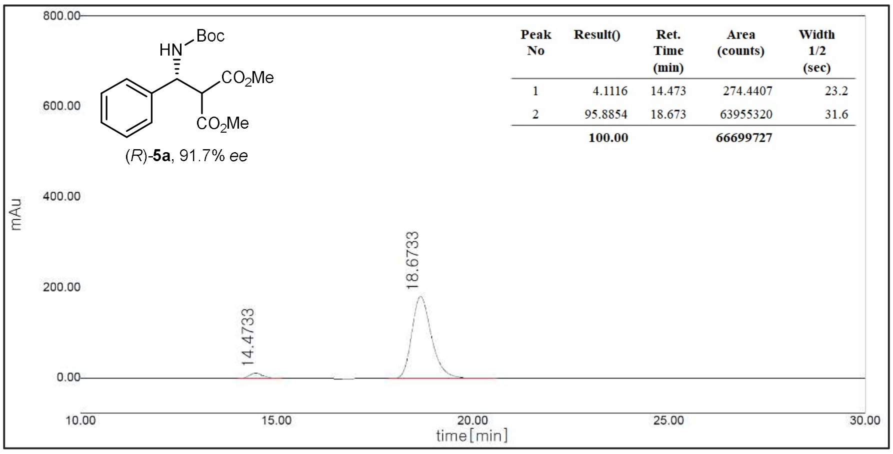

Peak No	Result()	Ret. Time (min)	Area (counts)	Width 1/2 (sec)
1	4.1116	14.473	274.4407	23.2
2	95.8854	18.673	63955320	31.6
	**100.00**		**66699727**	

Supplementary Figure 157. HPLC spectra of **5a** (**CN-2**, ID = 500 μm, Qw/Qo = 20:1)

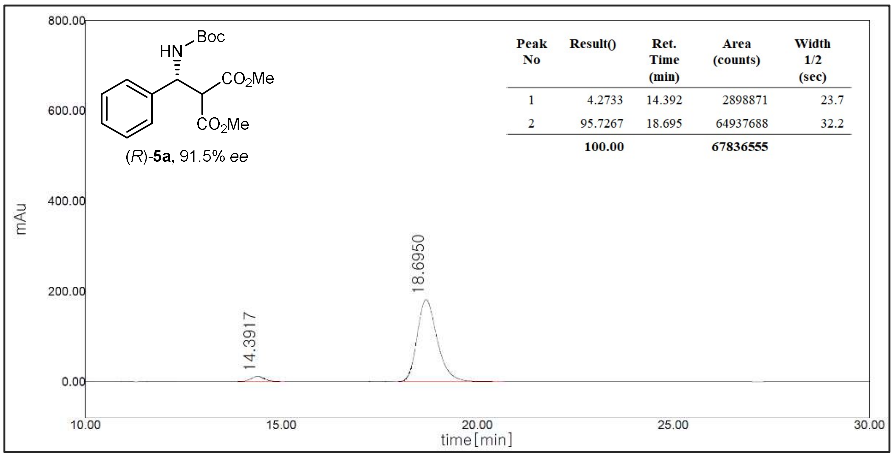

Peak No	Result()	Ret. Time (min)	Area (counts)	Width 1/2 (sec)
1	4.2733	14.392	2898871	23.7
2	95.7267	18.695	64937688	32.2
	**100.00**		**67836555**	

Supplementary Figure 158. HPLC spectra of **5a** (**CN-2**, ID = 500 μm, Qw/Qo = 20:20)

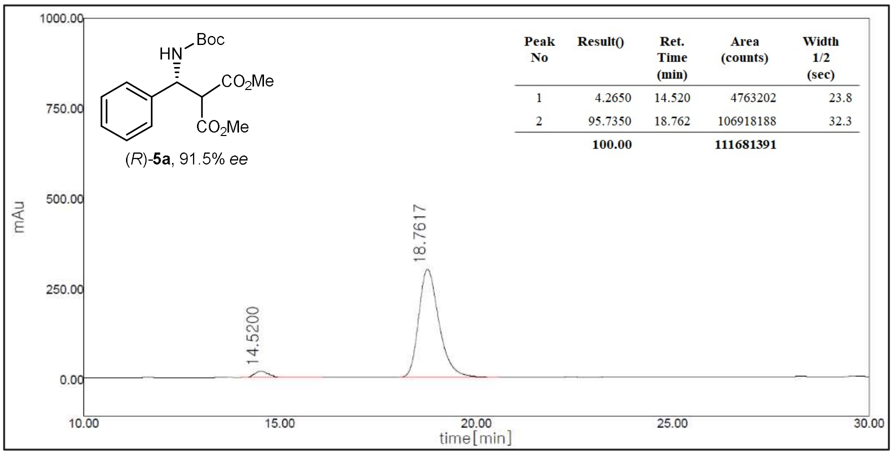

Peak No	Result()	Ret. Time (min)	Area (counts)	Width 1/2 (sec)
1	4.2650	14.520	4763202	23.8
2	95.7350	18.762	106918188	32.3
	**100.00**		**111681391**	

Supplementary Figure 159. HPLC spectra of **5a** (**CN-2**, ID = 500 μm, Qw/Qo = 1:20)

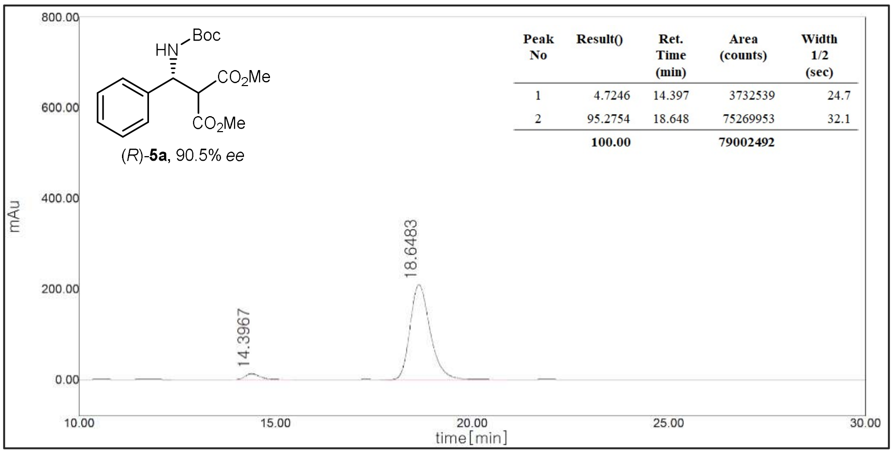

Supplementary Figure 160. HPLC spectra of **5a** (**CN-2**, ID = 1000 μm, Qw/Qo = 20:1)

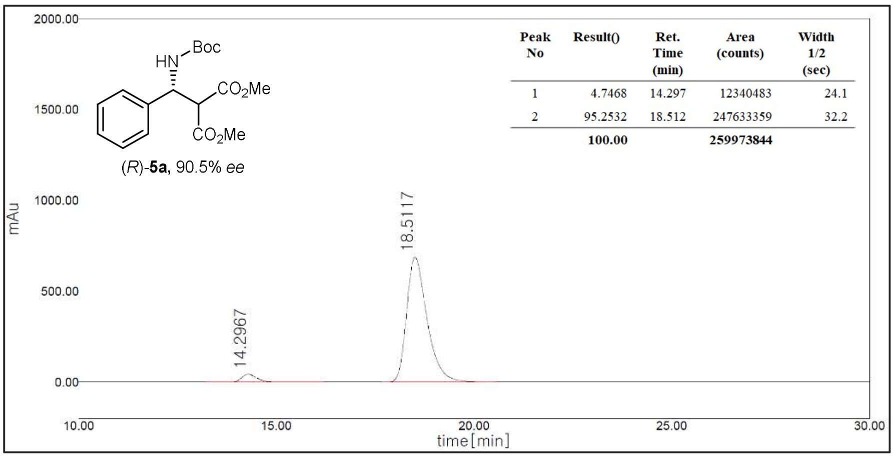

Supplementary Figure 161. HPLC spectra of **5a** (**CN-2**, ID = 1000 μm, Qw/Qo = 20:20)

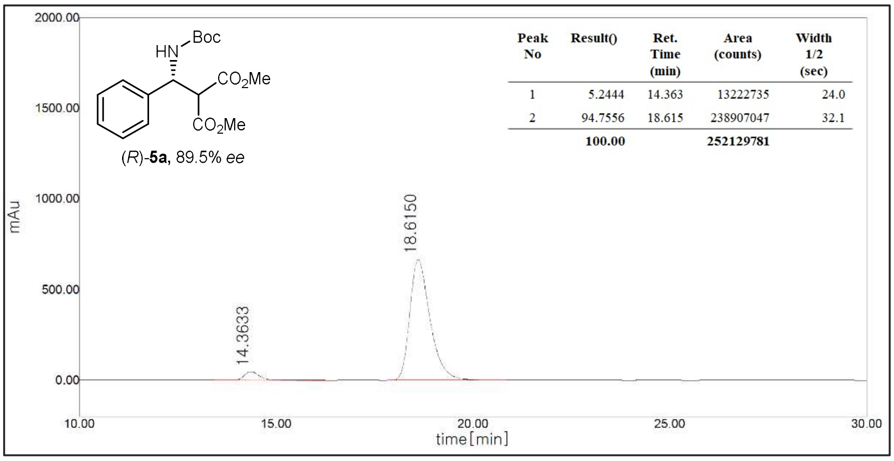

Supplementary Figure 162. HPLC spectra of **5a** (**CN-2**, ID = 1000 μm, Qw/Qo = 1:20)

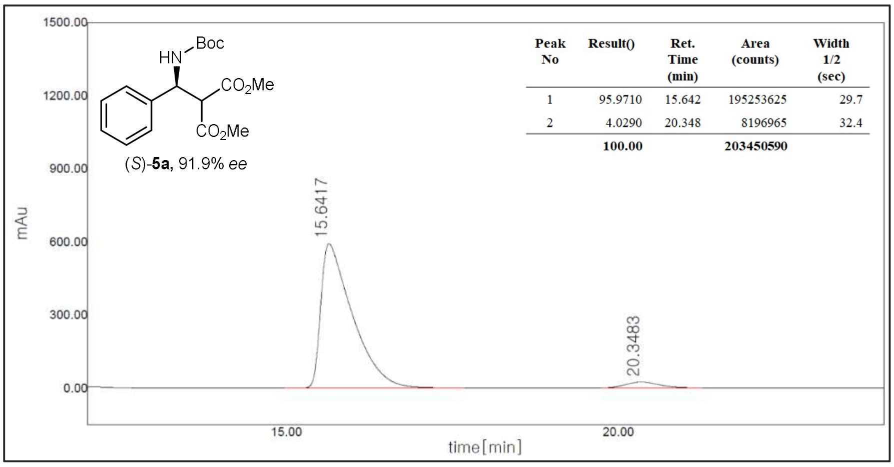

Supplementary Figure 163. HPLC spectra of **5a** (**CD-2**, ID = 250 µm, Qw/Qo = 20:1)

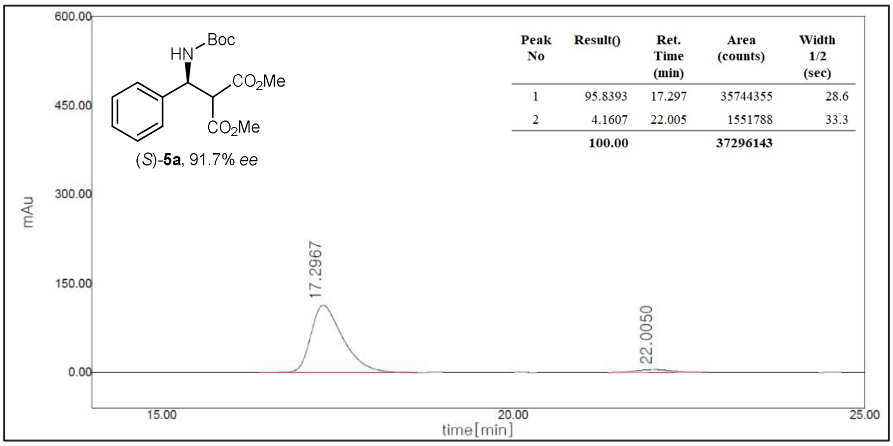

Supplementary Figure 164. HPLC spectra of **5a** (**CD-2**, ID = 250 µm, Qw/Qo = 20:20)

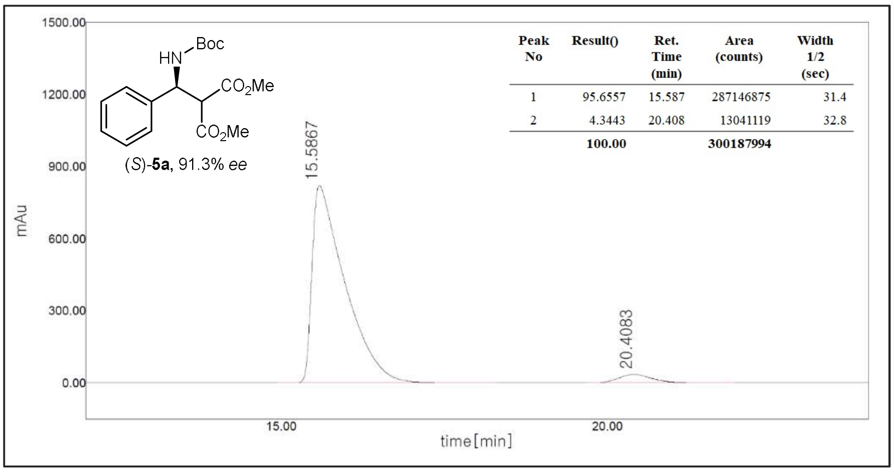

Supplementary Figure 165. HPLC spectra of **5a** (**CD-2**, ID = 250 µm, Qw/Qo = 1:20)

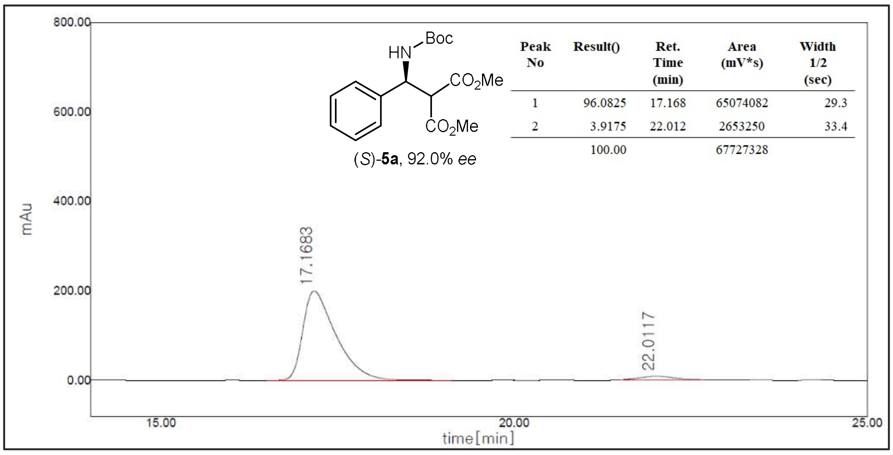

Supplementary Figure 166. HPLC spectra of **5a** (**CD-2**, ID = 500 µm, Qw/Qo = 20:1)

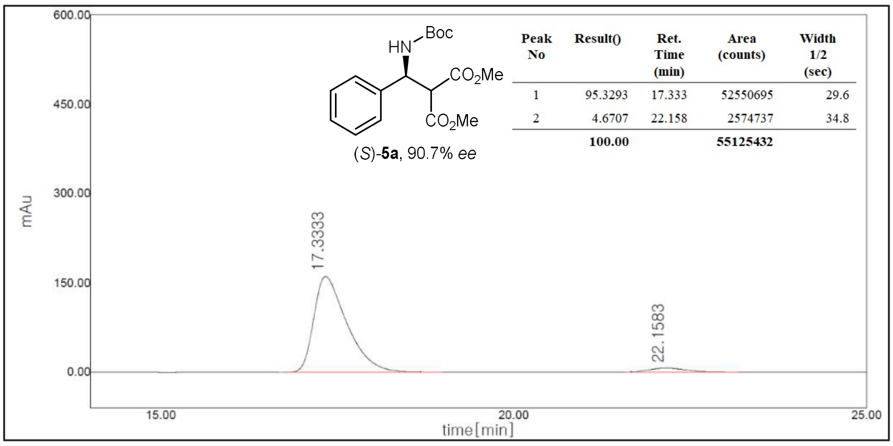

Supplementary Figure 167. HPLC spectra of **5a** (**CD-2**, ID = 500 µm, Qw/Qo = 20:20)

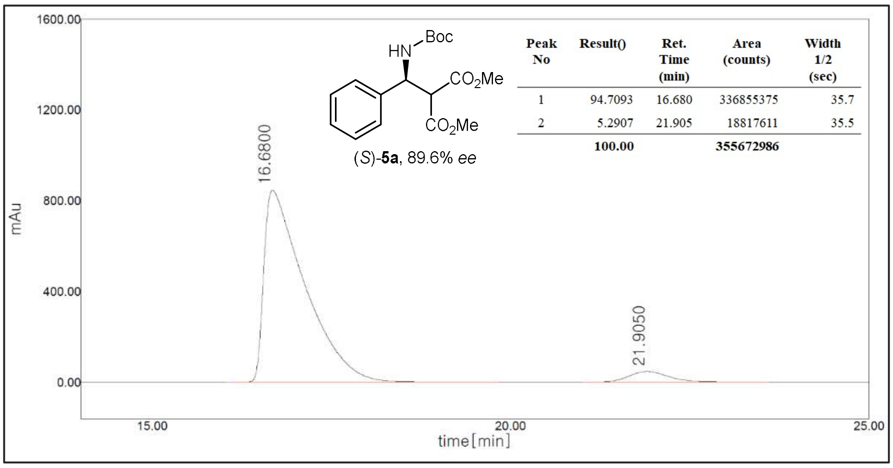

Supplementary Figure 168. HPLC spectra of **5a** (**CD-2**, ID = 500 µm, Qw/Qo = 1:20)

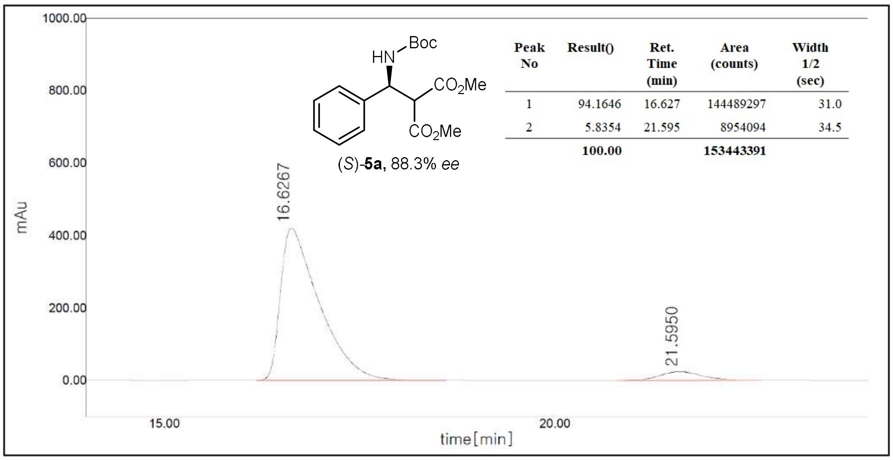

Peak No	Result()	Ret. Time (min)	Area (counts)	Width 1/2 (sec)
1	94.1646	16.627	144489297	31.0
2	5.8354	21.595	8954094	34.5
	100.00		153443391	

Supplementary Figure 169. HPLC spectra of **5a** (**CD-2**, ID = 1000 μm, Qw/Qo = 20:1)

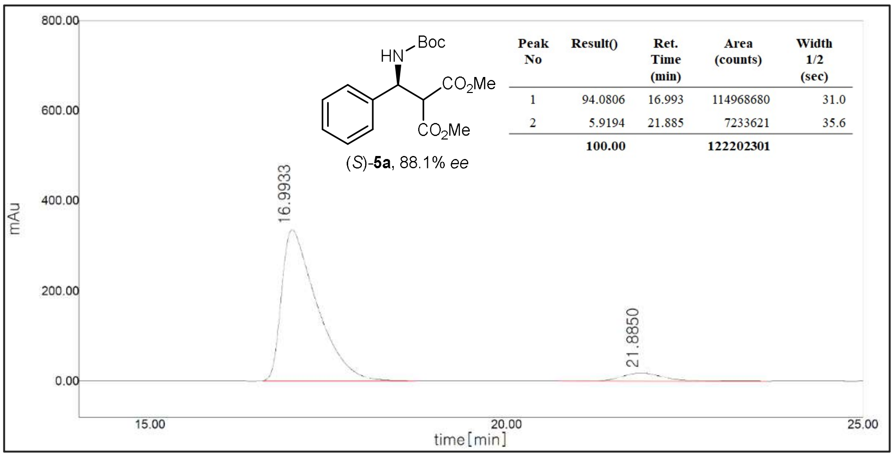

Peak No	Result()	Ret. Time (min)	Area (counts)	Width 1/2 (sec)
1	94.0806	16.993	114968680	31.0
2	5.9194	21.885	7233621	35.6
	100.00		122202301	

Supplementary Figure 170. HPLC spectra of **5a** (**CD-2**, ID = 1000 μm, Qw/Qo = 20:20)

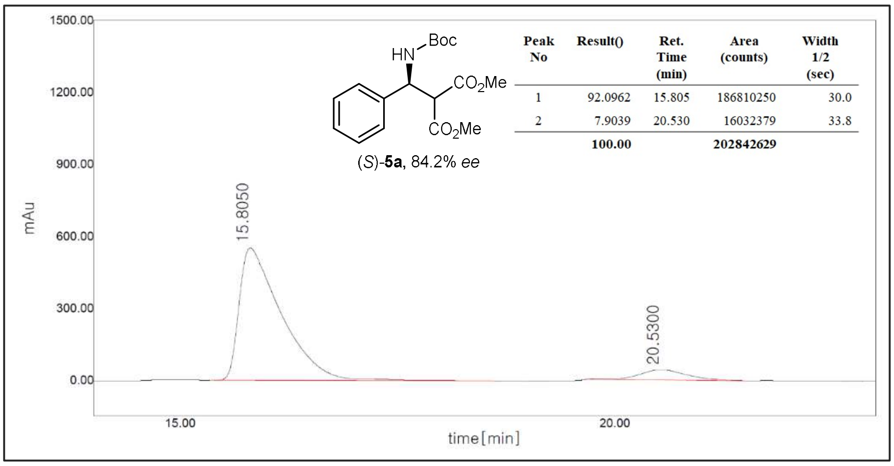

Peak No	Result()	Ret. Time (min)	Area (counts)	Width 1/2 (sec)
1	92.0962	15.805	186810250	30.0
2	7.9039	20.530	16032379	33.8
	100.00		202842629	

Supplementary Figure 171. HPLC spectra of **5a** (**CD-2**, ID = 1000 μm, Qw/Qo = 1:20)

HPLC spectra of Supplementary Table 3

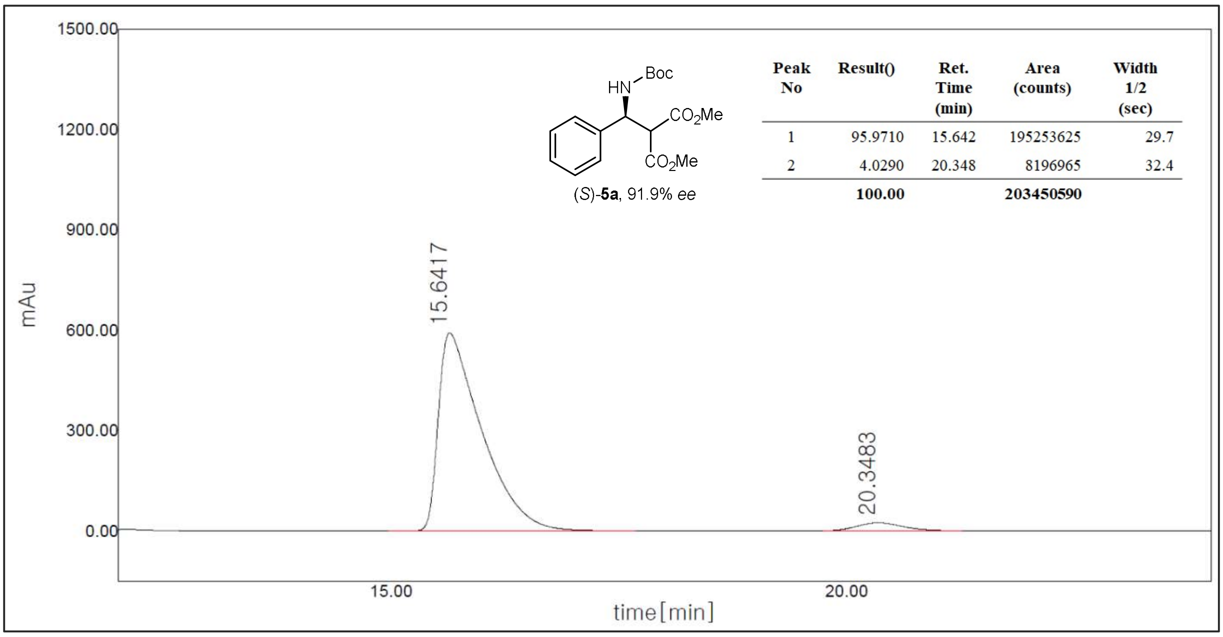

Peak No	Result()	Ret. Time (min)	Area (counts)	Width 1/2 (sec)
1	95.9710	15.642	195253625	29.7
2	4.0290	20.348	8196965	32.4
	**100.00**		**203450590**	

Supplementary Figure 172. HPLC spectra of Supplementary Table 3-Entry 1

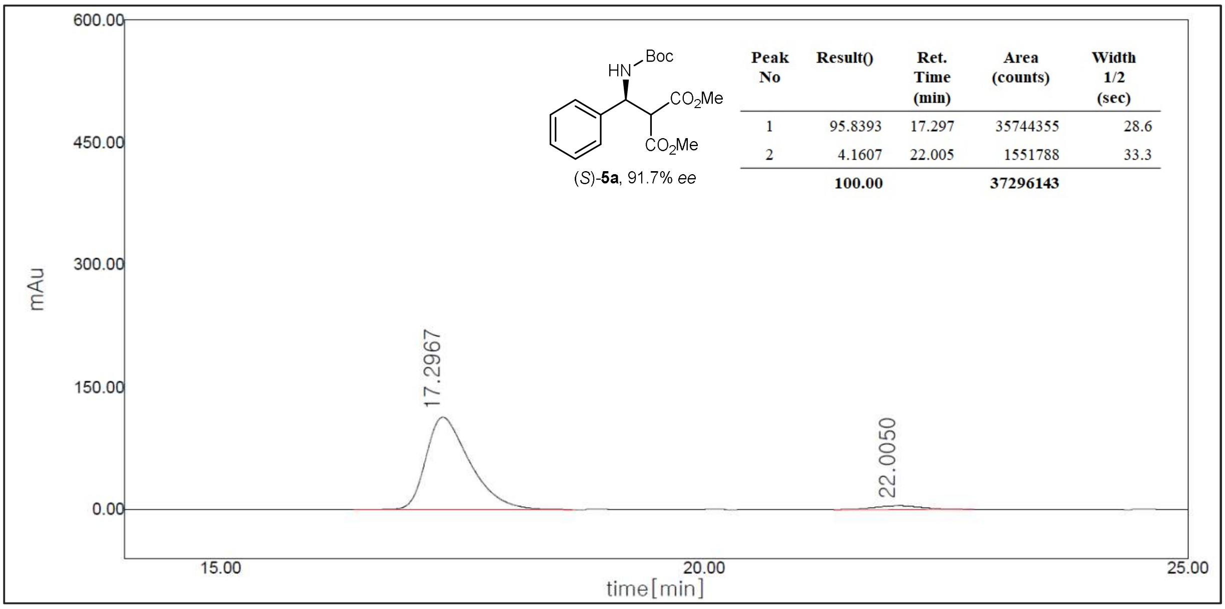

Peak No	Result()	Ret. Time (min)	Area (counts)	Width 1/2 (sec)
1	95.8393	17.297	35744355	28.6
2	4.1607	22.005	1551788	33.3
	**100.00**		**37296143**	

Supplementary Figure 173. HPLC spectra of Supplementary Table 3-Entry 2

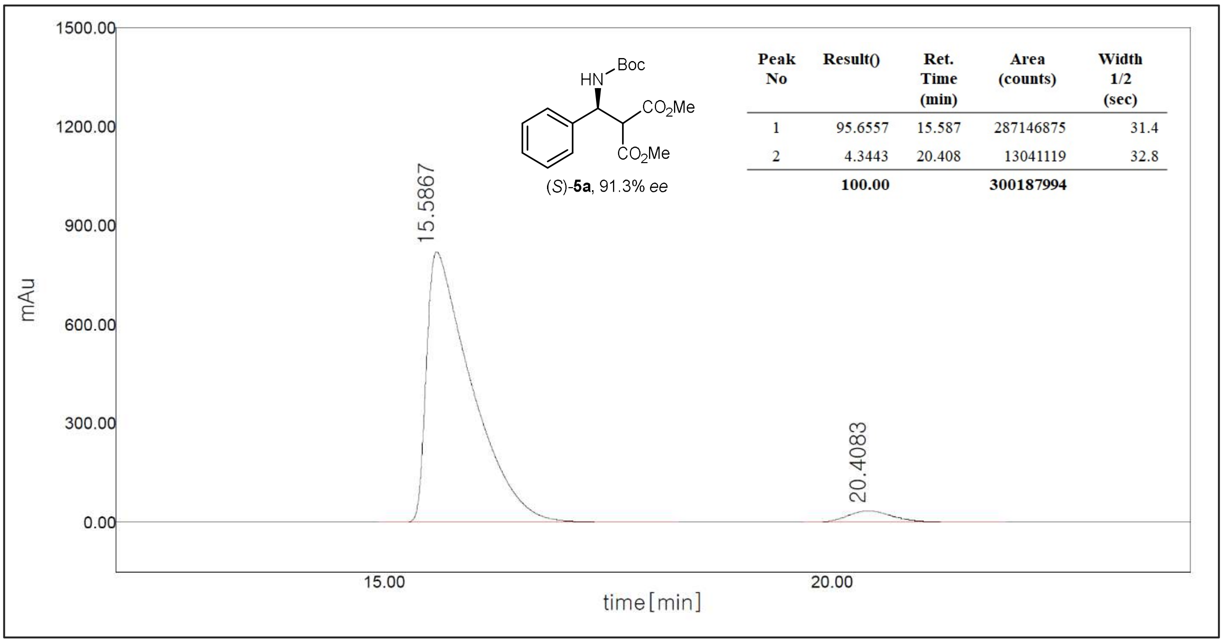

Peak No	Result()	Ret. Time (min)	Area (counts)	Width 1/2 (sec)
1	95.6557	15.587	287146875	31.4
2	4.3443	20.408	13041119	32.8
	**100.00**		**300187994**	

Supplementary Figure 174. HPLC spectra of Supplementary Table 3-Entry 3

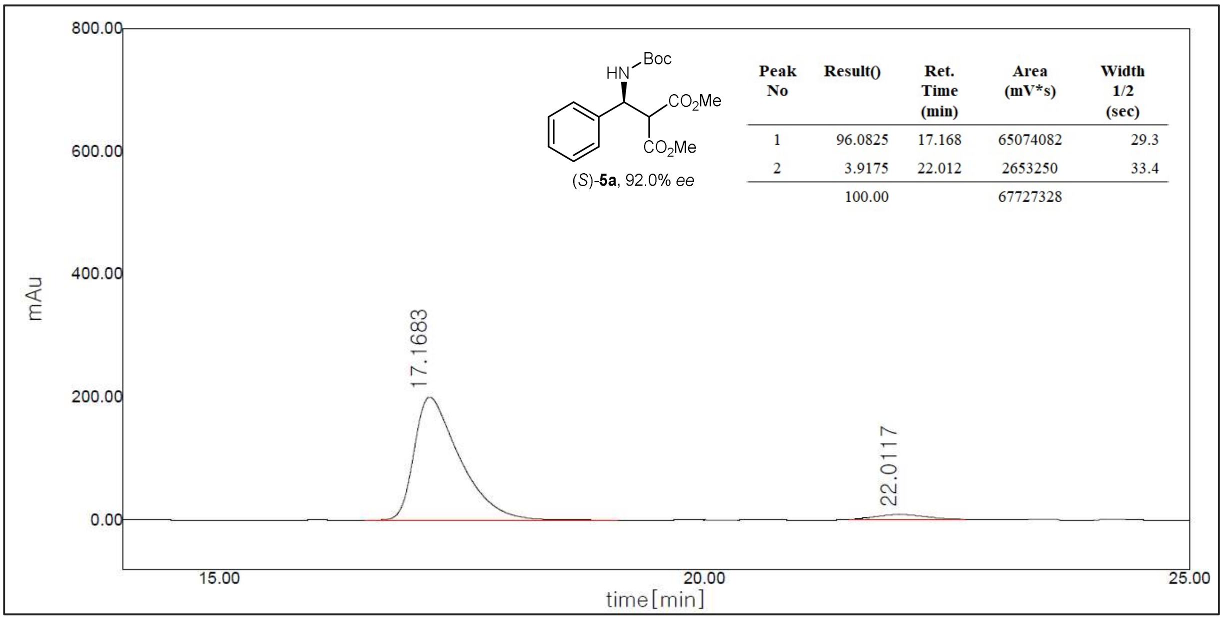

Peak No	Result()	Ret. Time (min)	Area (mV*s)	Width 1/2 (sec)
1	96.0825	17.168	65074082	29.3
2	3.9175	22.012	2653250	33.4
	100.00		67727328	

Supplementary Figure 175. HPLC spectra of Supplementary Table 3-Entry 4

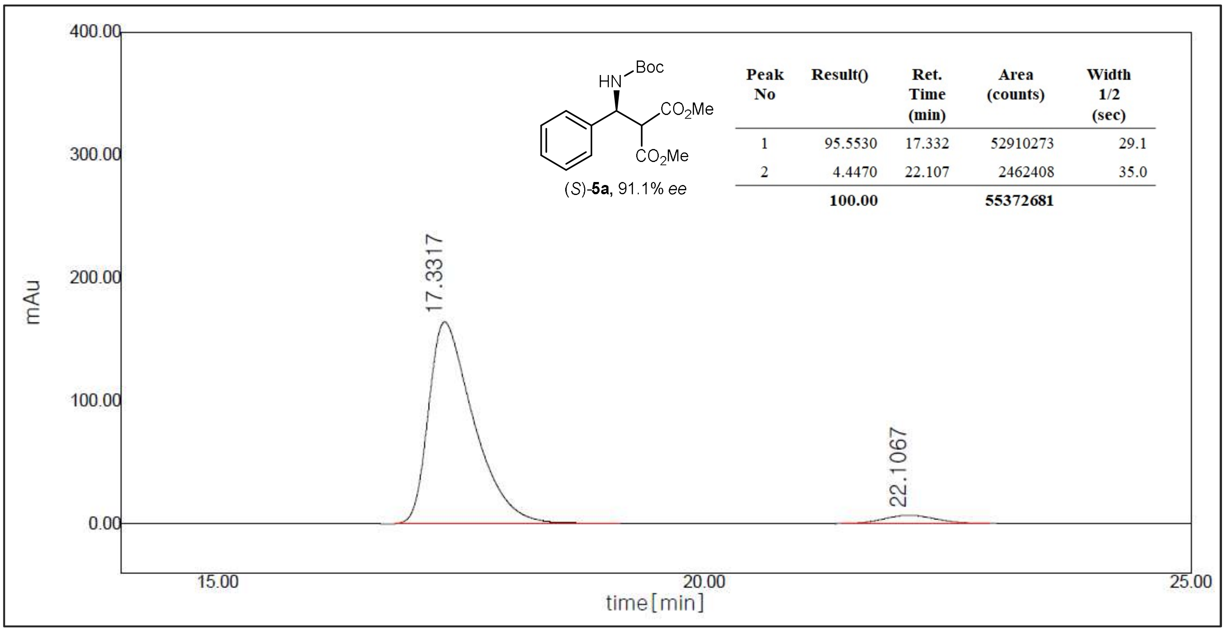

Peak No	Result()	Ret. Time (min)	Area (counts)	Width 1/2 (sec)
1	95.5530	17.332	52910273	29.1
2	4.4470	22.107	2462408	35.0
	**100.00**		**55372681**	

Supplementary Figure 176. HPLC spectra of Supplementary Table 3-Entry 5

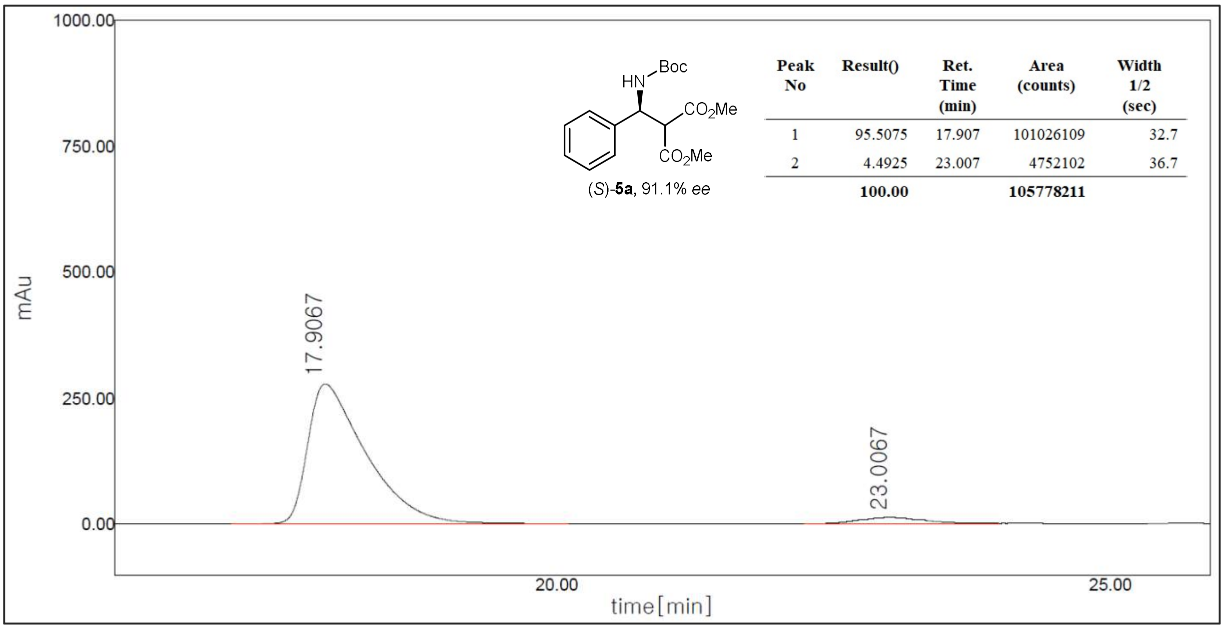

Peak No	Result()	Ret. Time (min)	Area (counts)	Width 1/2 (sec)
1	95.5075	17.907	101026109	32.7
2	4.4925	23.007	4752102	36.7
	**100.00**		**105778211**	

Supplementary Figure 177. HPLC spectra of Supplementary Table 3-Entry 6

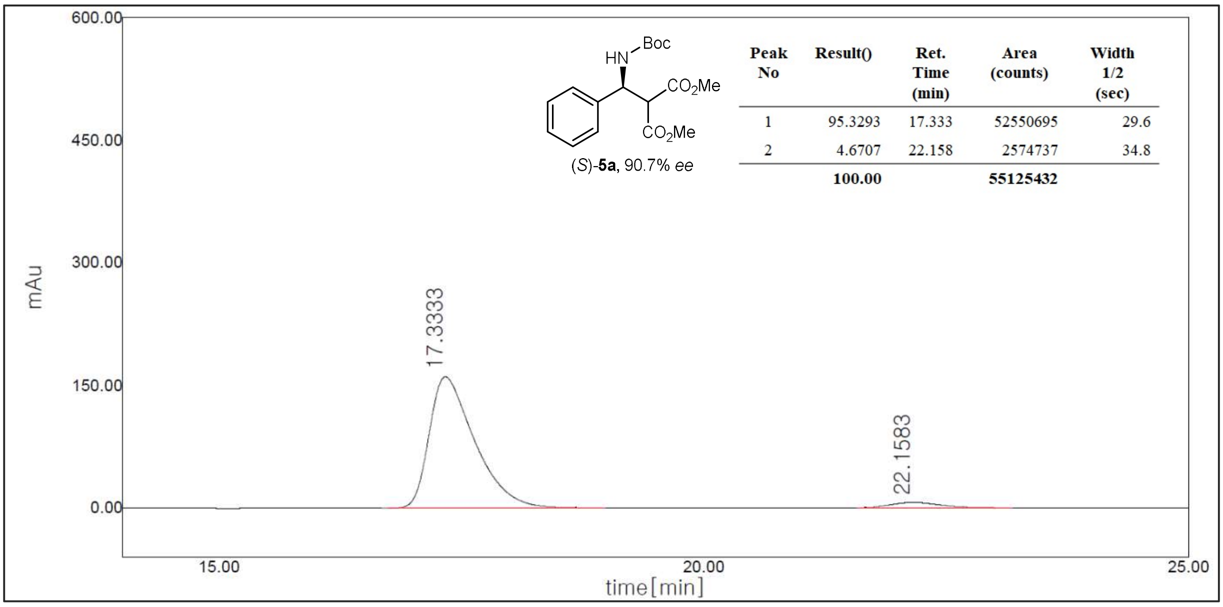

Peak No	Result()	Ret. Time (min)	Area (counts)	Width 1/2 (sec)
1	95.3293	17.333	52550695	29.6
2	4.6707	22.158	2574737	34.8
	**100.00**		**55125432**	

Supplementary Figure 178. HPLC spectra of Supplementary Table 3-Entry 7

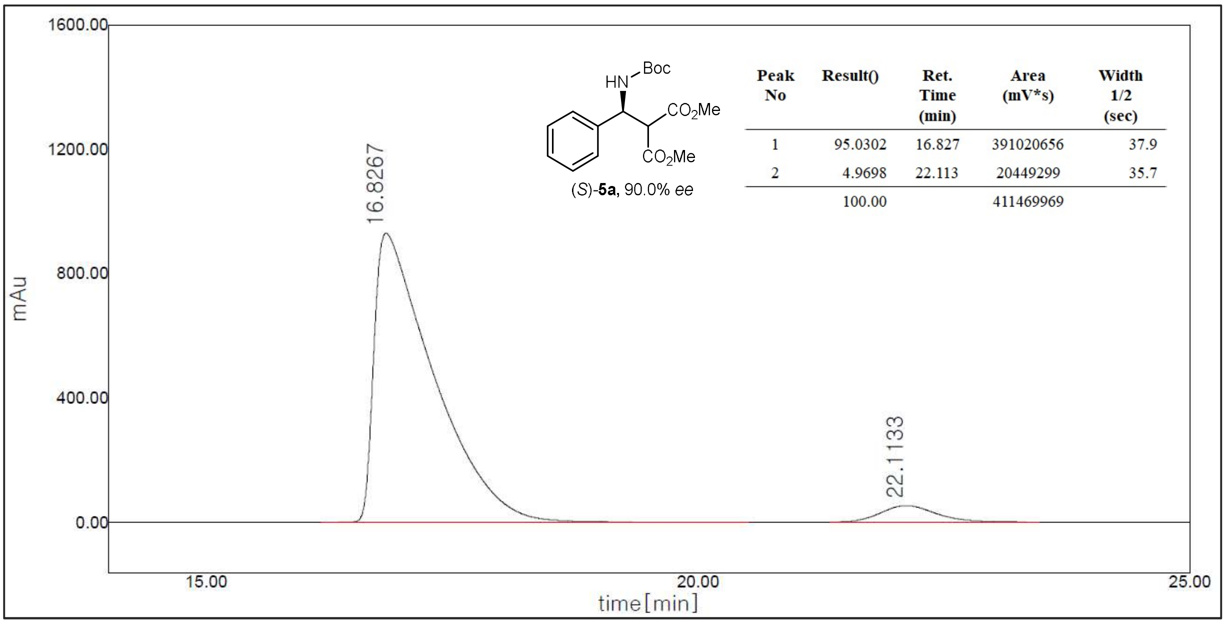

Peak No	Result()	Ret. Time (min)	Area (mV*s)	Width 1/2 (sec)
1	95.0302	16.827	391020656	37.9
2	4.9698	22.113	20449299	35.7
	100.00		411469969	

Supplementary Figure 179. HPLC spectra of Supplementary Table 3-Entry 8

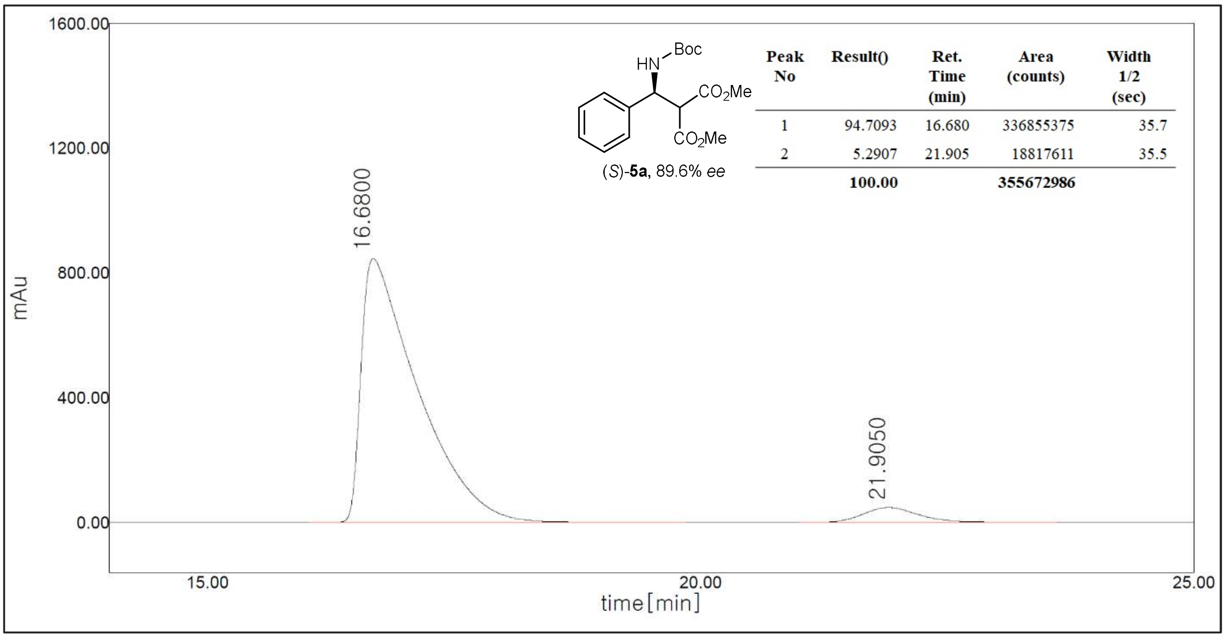

Peak No	Result()	Ret. Time (min)	Area (counts)	Width 1/2 (sec)
1	94.7093	16.680	336855375	35.7
2	5.2907	21.905	18817611	35.5
	100.00		355672986	

Supplementary Figure 180. HPLC spectra of Supplementary Table 3-Entry 9

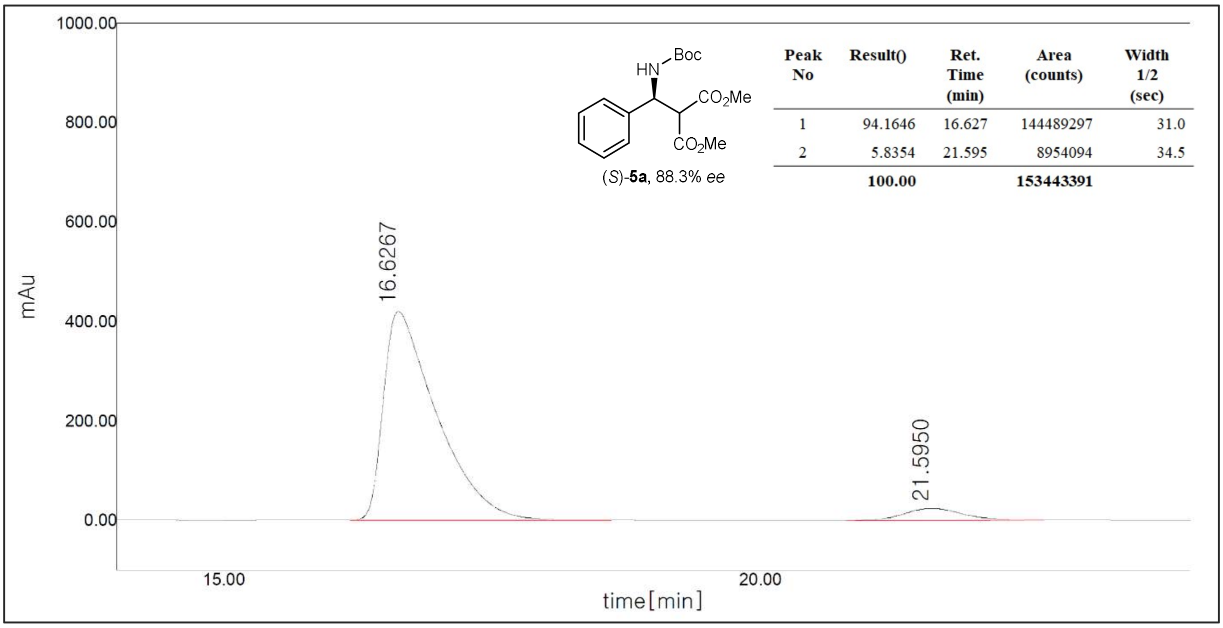

Peak No	Result()	Ret. Time (min)	Area (counts)	Width 1/2 (sec)
1	94.1646	16.627	144489297	31.0
2	5.8354	21.595	8954094	34.5
	100.00		153443391	

Supplementary Figure 181. HPLC spectra of Supplementary Table 3-Entry 10

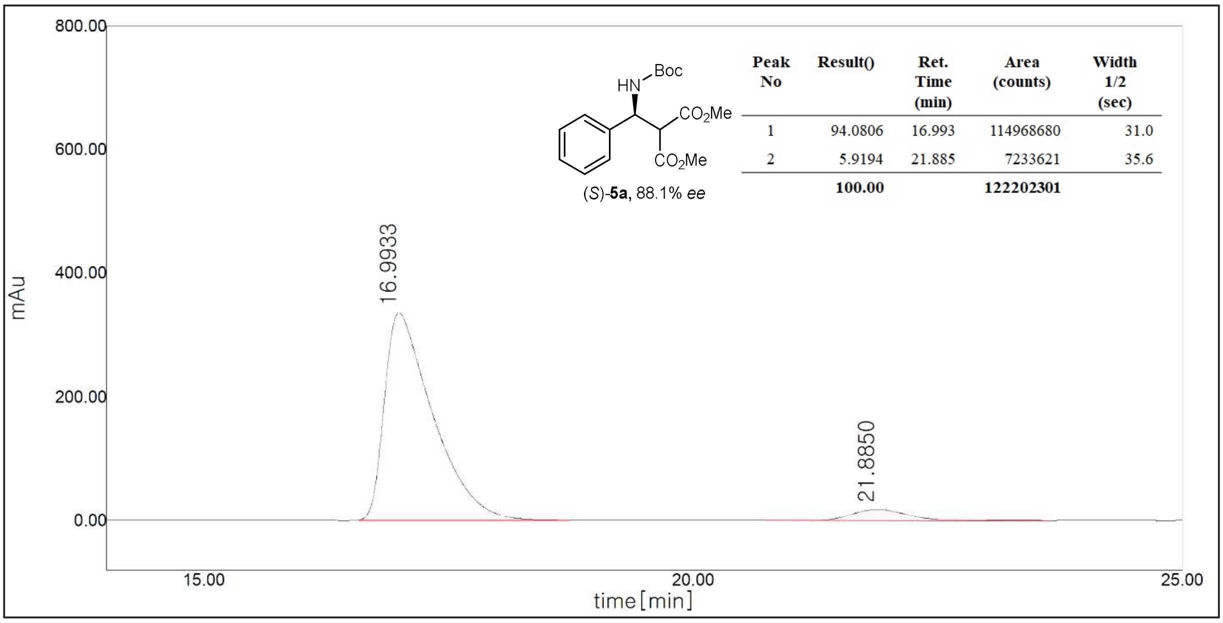

Peak No	Result()	Ret. Time (min)	Area (counts)	Width 1/2 (sec)
1	94.0806	16.993	114968680	31.0
2	5.9194	21.885	7233621	35.6
	**100.00**		**122202301**	

Supplementary Figure 182. HPLC spectra of Supplementary Table 3-Entry 11

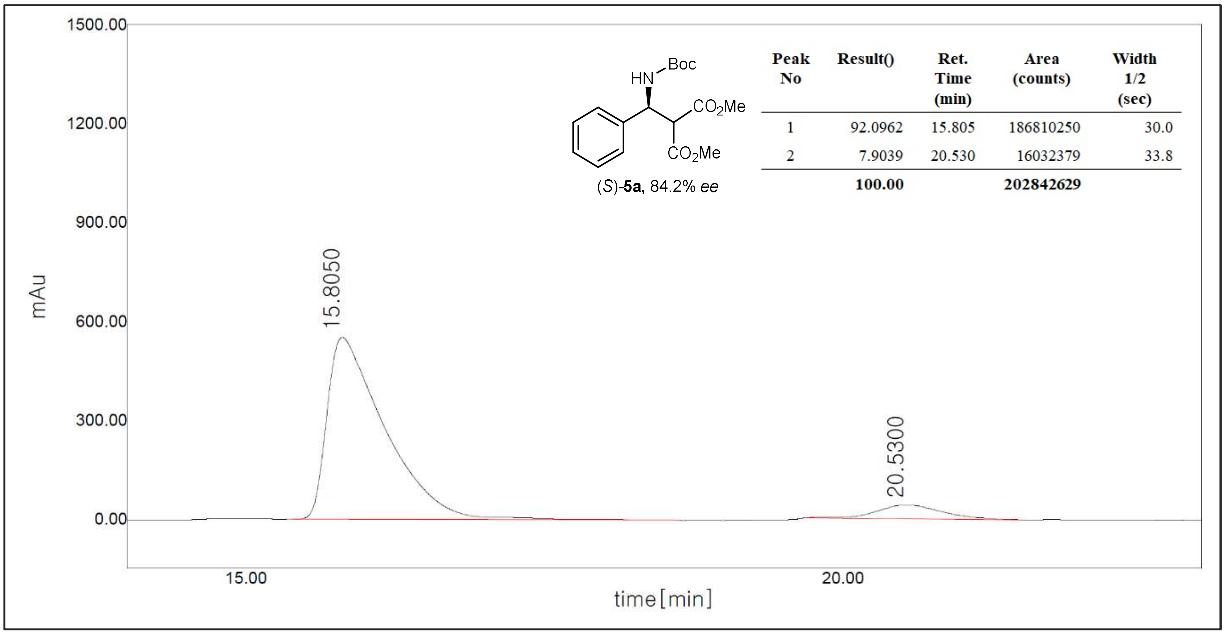

Peak No	Result()	Ret. Time (min)	Area (counts)	Width 1/2 (sec)
1	92.0962	15.805	186810250	30.0
2	7.9039	20.530	16032379	33.8
	**100.00**		**202842629**	

Supplementary Figure 183. HPLC spectra of Supplementary Table 3-Entry 12

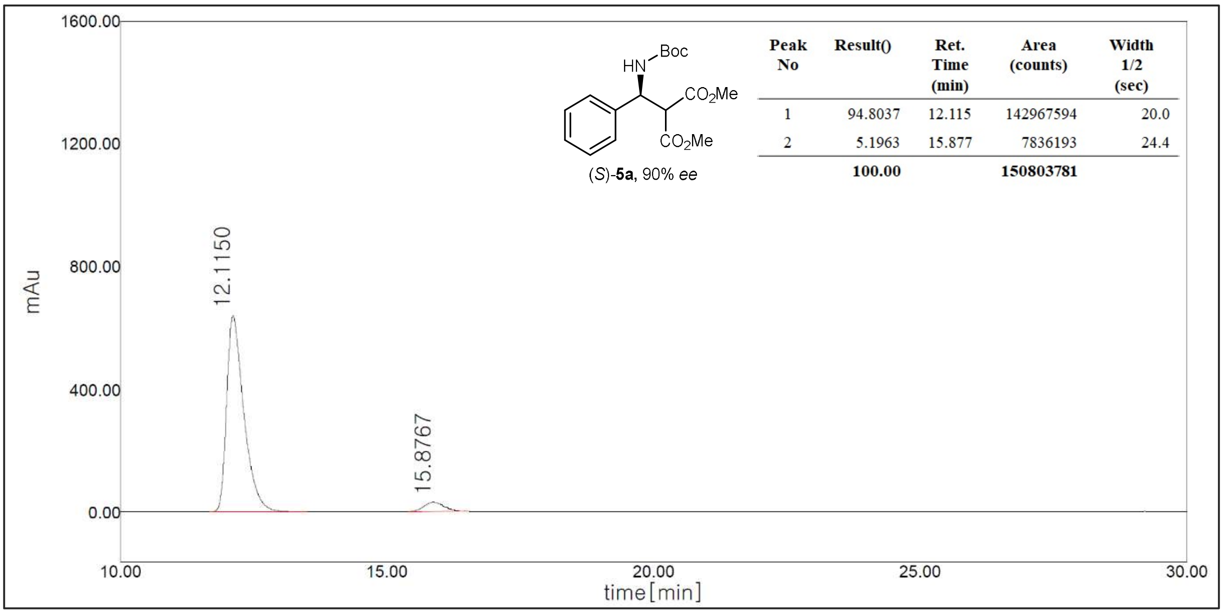

Peak No	Result()	Ret. Time (min)	Area (counts)	Width 1/2 (sec)
1	94.8037	12.115	142967594	20.0
2	5.1963	15.877	7836193	24.4
	**100.00**		**150803781**	

Supplementary Figure 184. HPLC spectra of Supplementary Table 3-Entry 13

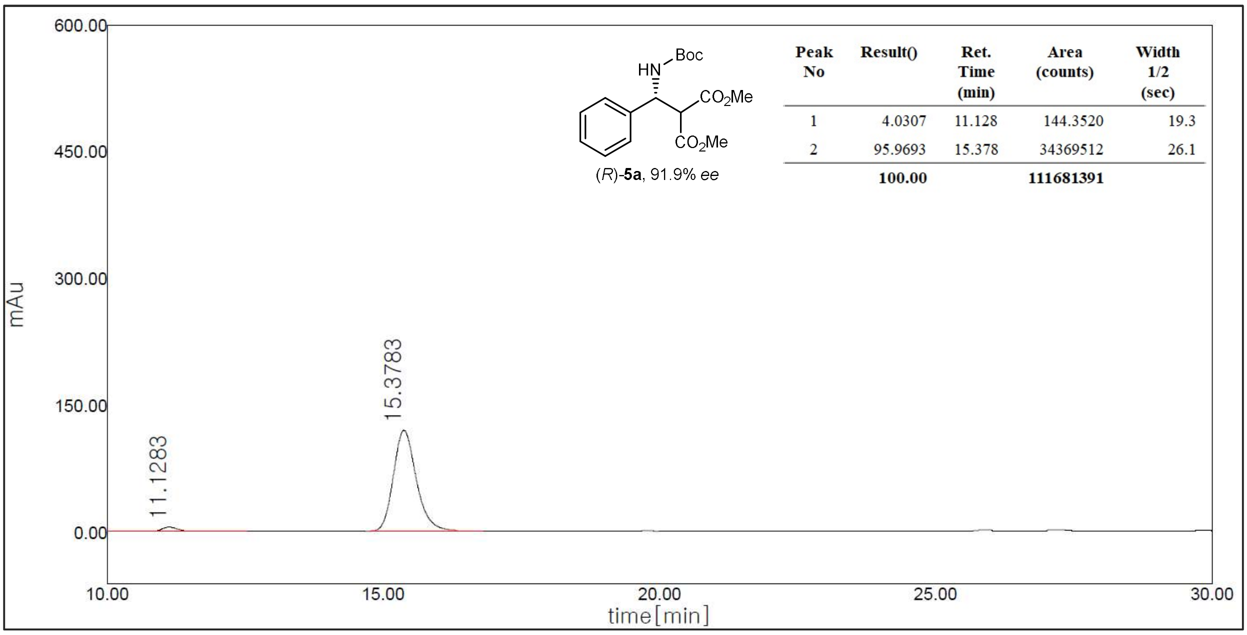

Peak No	Result()	Ret. Time (min)	Area (counts)	Width 1/2 (sec)
1	4.0307	11.128	144.3520	19.3
2	95.9693	15.378	34369512	26.1
	**100.00**		**111681391**	

Supplementary Figure 185. HPLC spectra of Supplementary Table 3-Entry 14

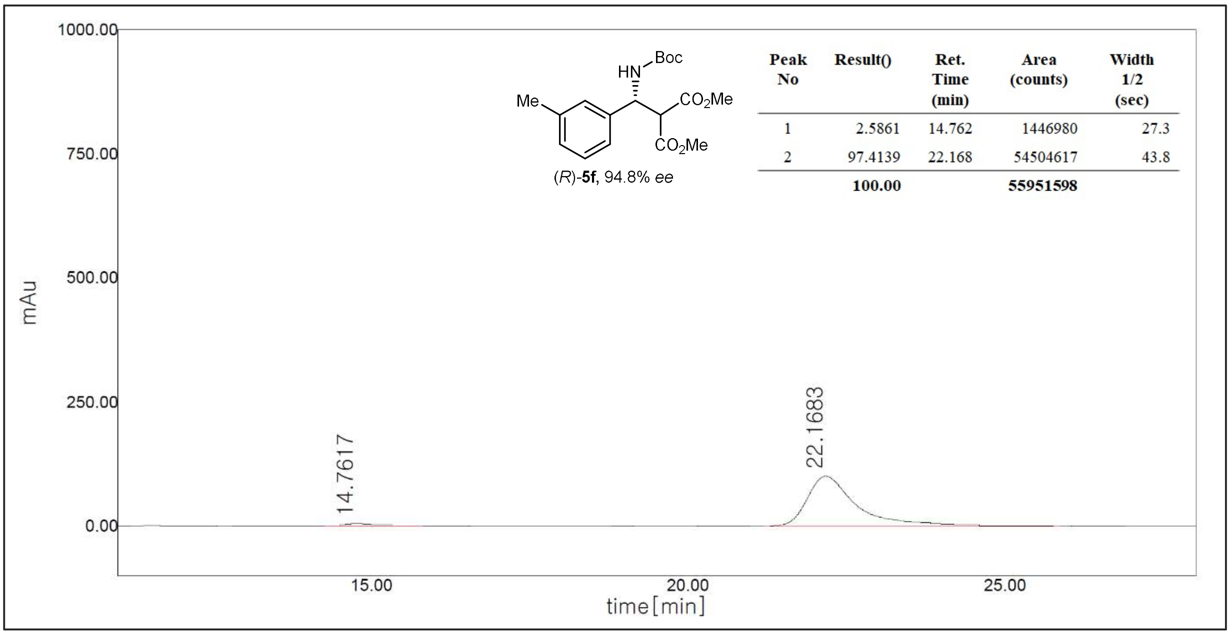

Peak No	Result()	Ret. Time (min)	Area (counts)	Width 1/2 (sec)
1	2.5861	14.762	1446980	27.3
2	97.4139	22.168	54504617	43.8
	**100.00**		**55951598**	

Supplementary Figure 186. HPLC spectra of Supplementary Table 3-Entry 15

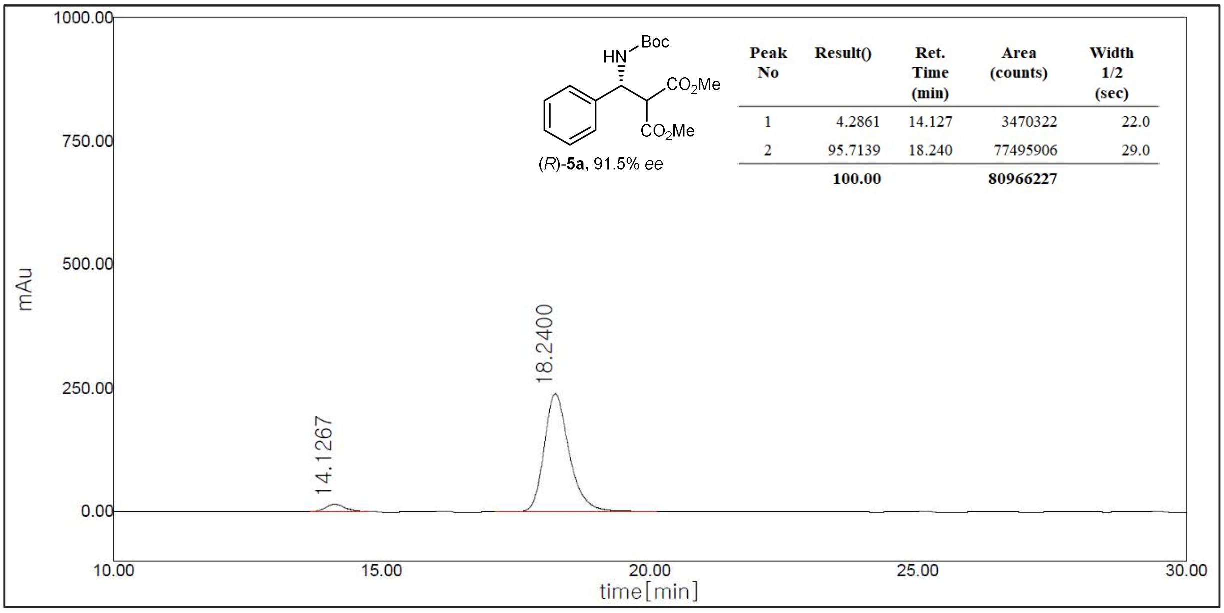

Peak No	Result()	Ret. Time (min)	Area (counts)	Width 1/2 (sec)
1	4.2861	14.127	3470322	22.0
2	95.7139	18.240	77495906	29.0
	**100.00**		**80966227**	

Supplementary Figure 187. HPLC spectra of Supplementary Table 3-Entry 16

HPLC spectra of Fig. 4a

High pressure effect on enantioselectivity under biphasic microfluidic conditions using a BPR.

5 bar (75 psi)

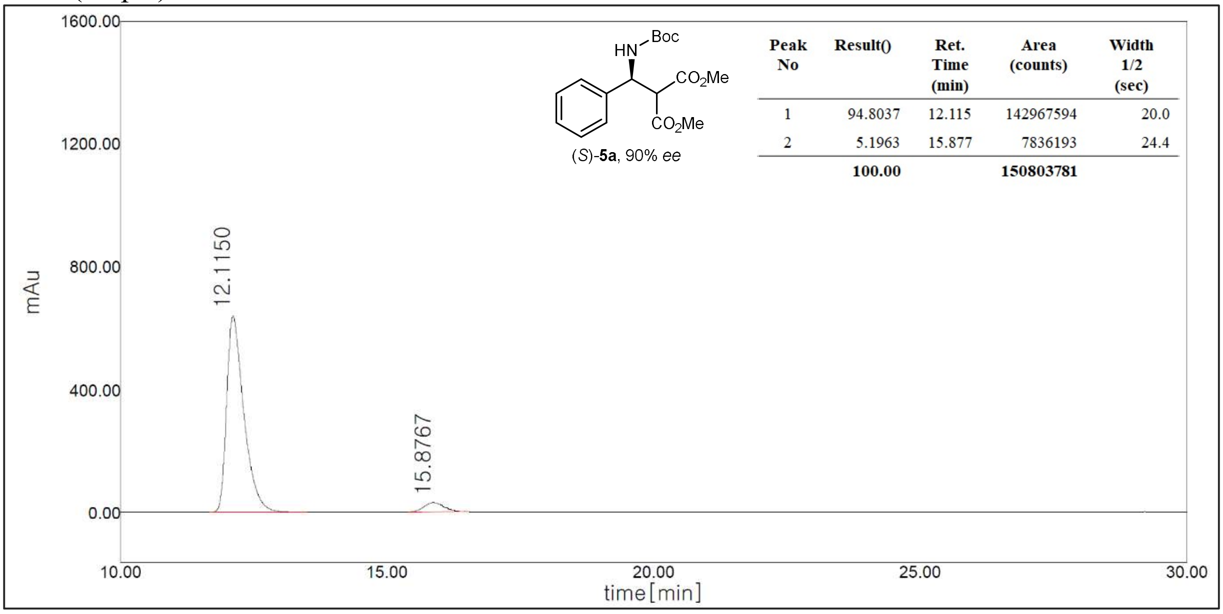

Peak No	Result()	Ret. Time (min)	Area (counts)	Width 1/2 (sec)
1	94.8037	12.115	142967594	20.0
2	5.1963	15.877	7836193	24.4
	**100.00**		**150803781**	

Supplementary Figure 188. HPLC spectra of Fig. 4a

HPLC spectra of Fig. 4b

High pressure effect on catalytic Mannich reaction.

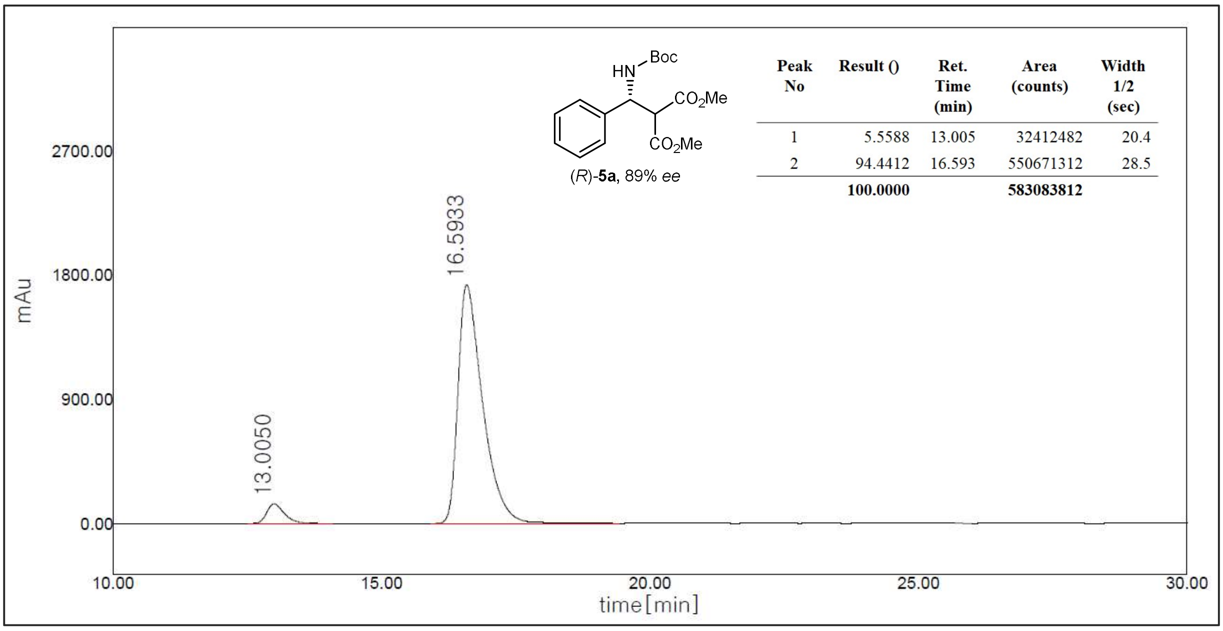

Peak No	Result ()	Ret. Time (min)	Area (counts)	Width 1/2 (sec)
1	5.5588	13.005	32412482	20.4
2	94.4412	16.593	550671312	28.5
	**100.0000**		**583083812**	

Supplementary Figure 189. HPLC spectra of Fig. 4b, **5a**

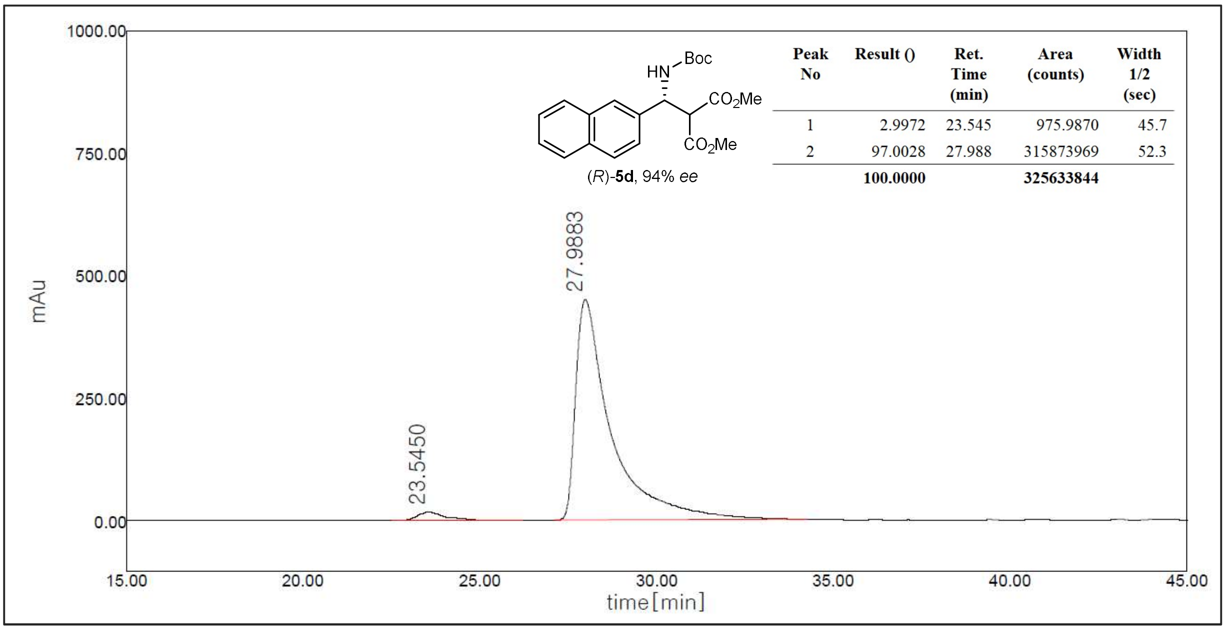

Peak No	Result ()	Ret. Time (min)	Area (counts)	Width 1/2 (sec)
1	2.9972	23.545	975.9870	45.7
2	97.0028	27.988	315873969	52.3
	**100.0000**		**325633844**	

Supplementary Figure 190. HPLC spectra of Fig. 4b, **5d**

HPLC spectra of Fig. 5a

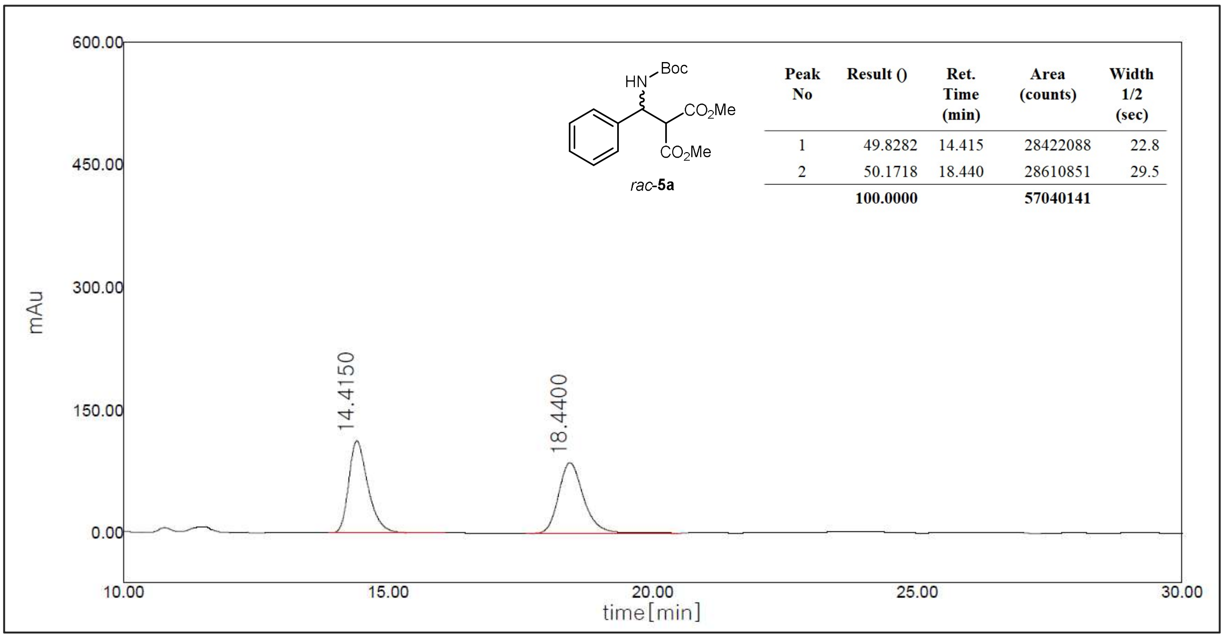

Peak No	Result ()	Ret. Time (min)	Area (counts)	Width 1/2 (sec)
1	49.8282	14.415	28422088	22.8
2	50.1718	18.440	28610851	29.5
	**100.0000**		**57040141**	

Supplementary Figure 191. HPLC spectra of *rac*-**5a**

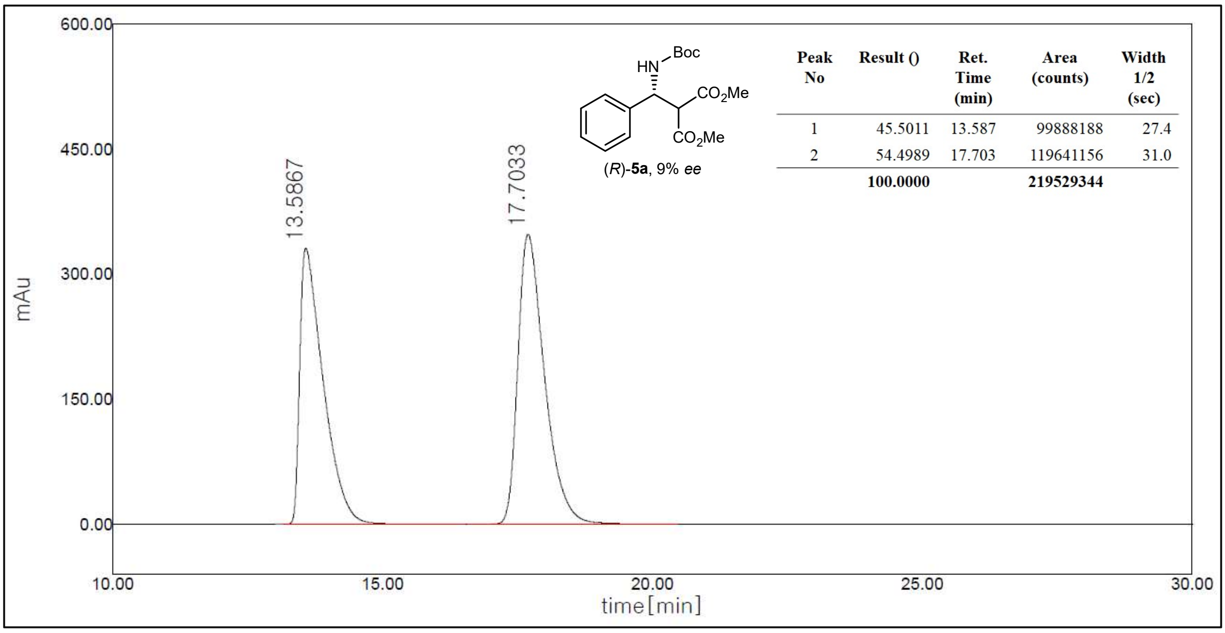

Peak No	Result ()	Ret. Time (min)	Area (counts)	Width 1/2 (sec)
1	45.5011	13.587	99888188	27.4
2	54.4989	17.703	119641156	31.0
	**100.0000**		**219529344**	

Supplementary Figure 192. HPLC spectra of **5a** (condition *a*)

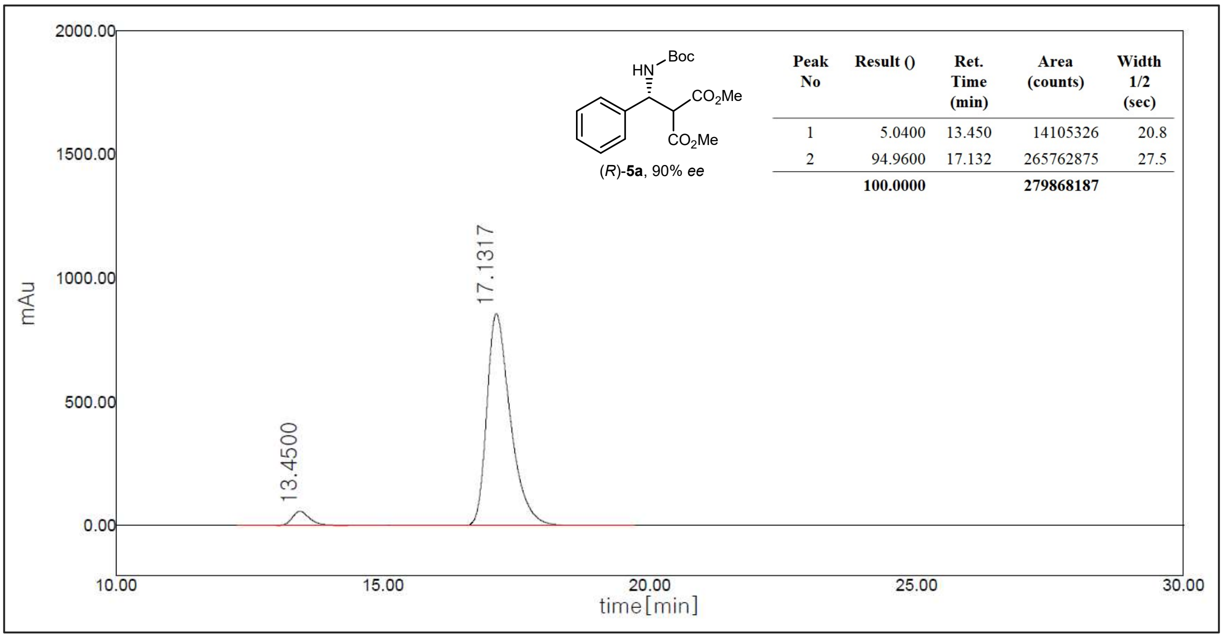

Peak No	Result ()	Ret. Time (min)	Area (counts)	Width 1/2 (sec)
1	5.0400	13.450	14105326	20.8
2	94.9600	17.132	265762875	27.5
	**100.0000**		**279868187**	

Supplementary Figure 193. HPLC spectra of **5a** (condition *b*)

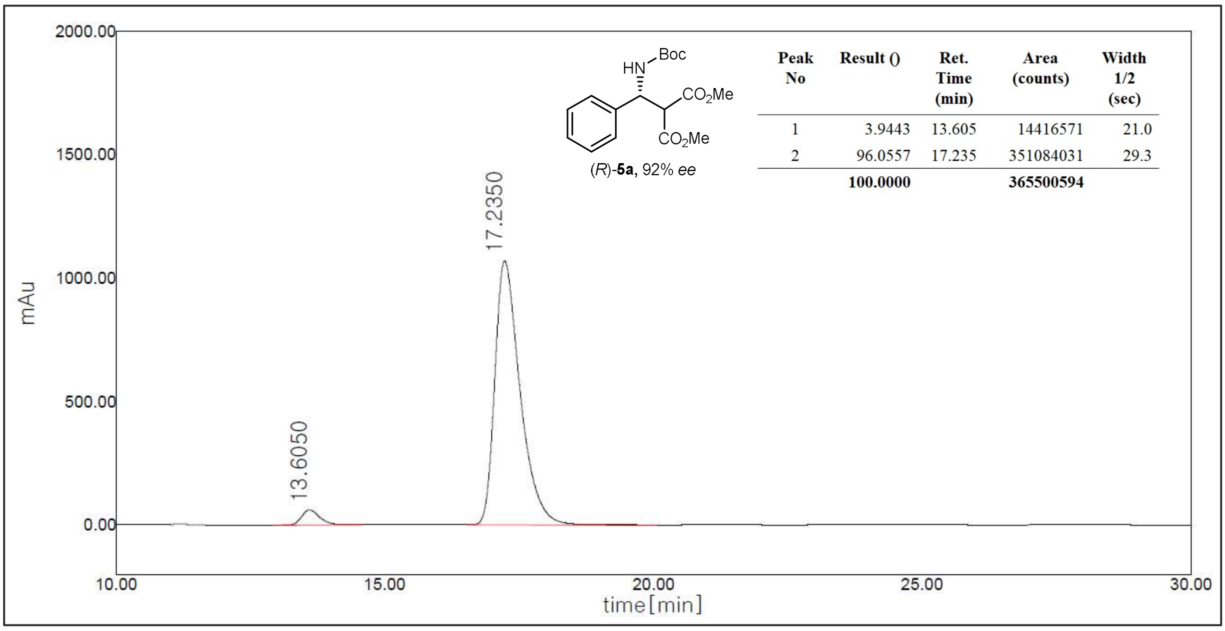

Peak No	Result ()	Ret. Time (min)	Area (counts)	Width 1/2 (sec)
1	3.9443	13.605	14416571	21.0
2	96.0557	17.235	351084031	29.3
	**100.0000**		**365500594**	

Supplementary Figure 194. HPLC spectra of **5a** (condition ***c-1***)

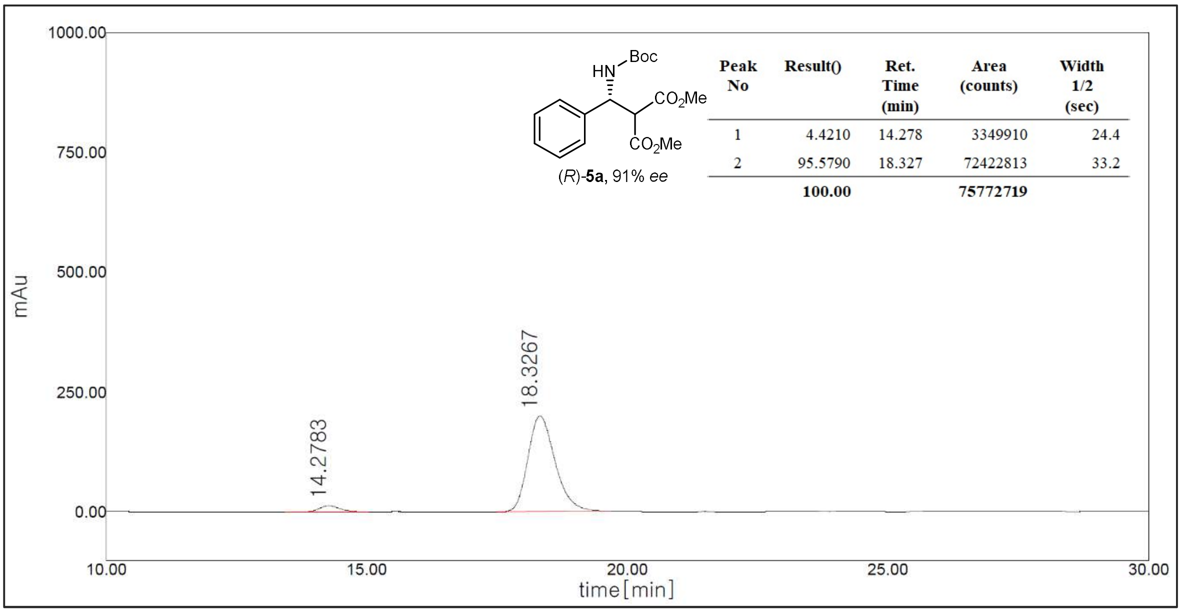

Peak No	Result()	Ret. Time (min)	Area (counts)	Width 1/2 (sec)
1	4.4210	14.278	3349910	24.4
2	95.5790	18.327	72422813	33.2
	**100.00**		**75772719**	

Supplementary Figure 195. HPLC spectra of **5a** (condition ***c-2***)

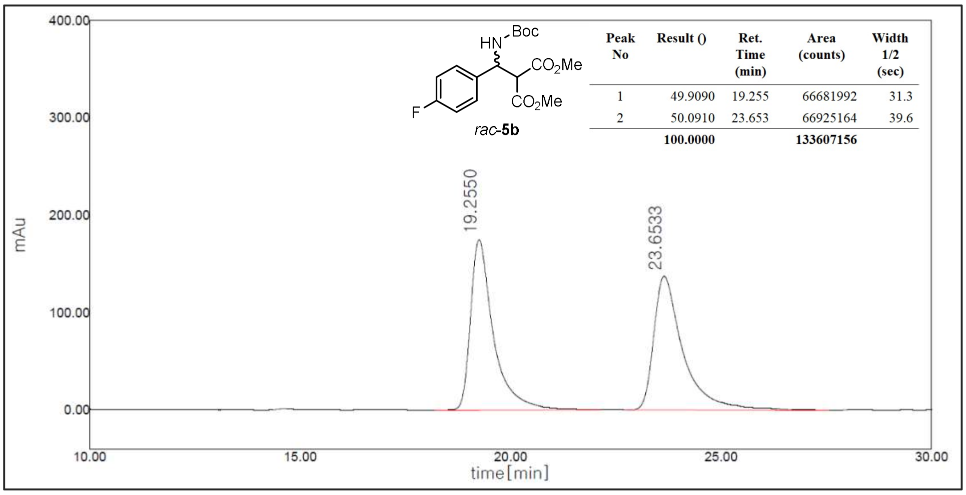

Peak No	Result ()	Ret. Time (min)	Area (counts)	Width 1/2 (sec)
1	49.9090	19.255	66681992	31.3
2	50.0910	23.653	66925164	39.6
	**100.0000**		**133607156**	

Supplementary Figure 196. HPLC spectra of *rac*-**5b**

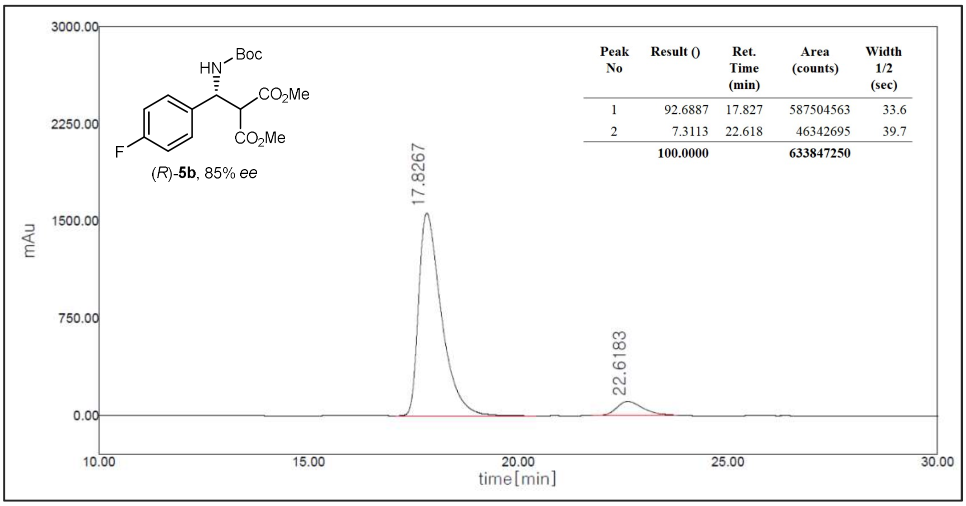

Peak No	Result ()	Ret. Time (min)	Area (counts)	Width 1/2 (sec)
1	92.6887	17.827	587504563	33.6
2	7.3113	22.618	46342695	39.7
	**100.0000**		**633847250**	

Supplementary Figure 197. HPLC spectra of **5b** (condition ***b***)

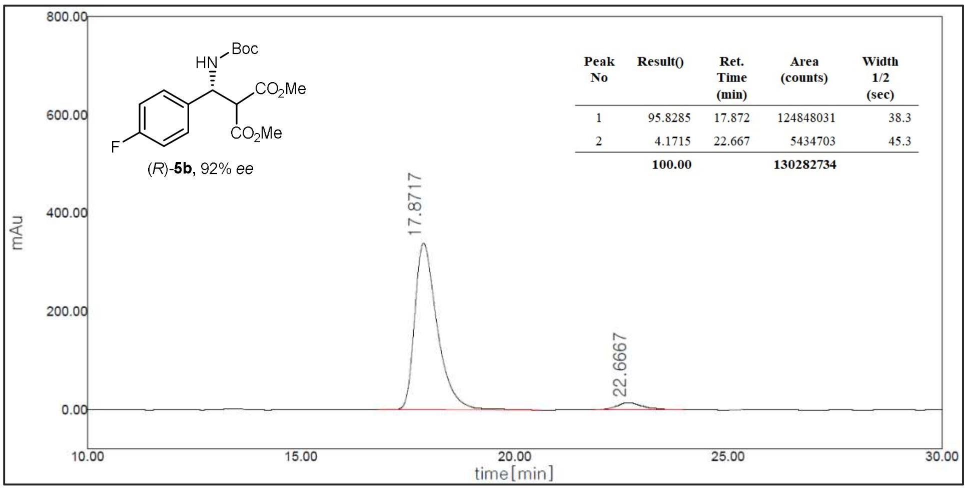

Peak No	Result()	Ret. Time (min)	Area (counts)	Width 1/2 (sec)
1	95.8285	17.872	124848031	38.3
2	4.1715	22.667	5434703	45.3
	**100.00**		**130282734**	

Supplementary Figure 198. HPLC spectra of **5b** (condition ***c-1***)

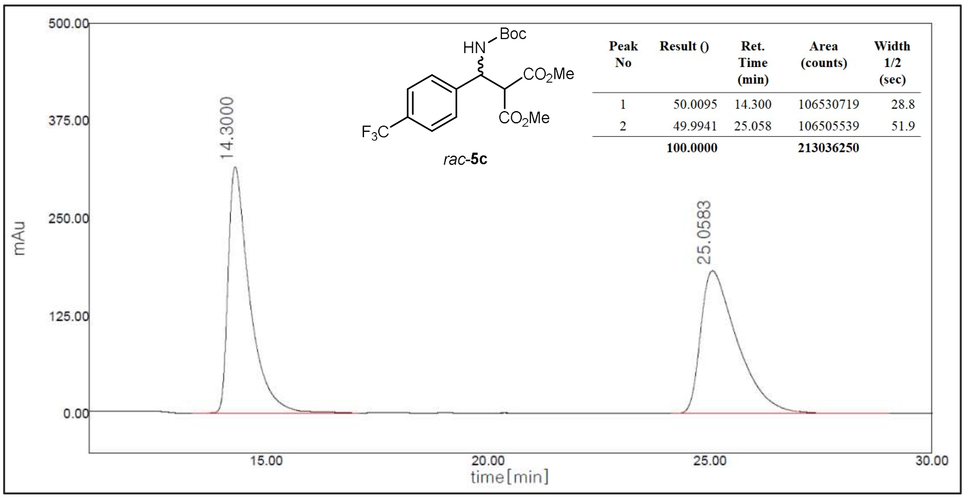

Peak No	Result ()	Ret. Time (min)	Area (counts)	Width 1/2 (sec)
1	50.0095	14.300	106530719	28.8
2	49.9941	25.058	106505539	51.9
	**100.0000**		**213036250**	

Supplementary Figure 199. HPLC spectra of *rac*-**5c**

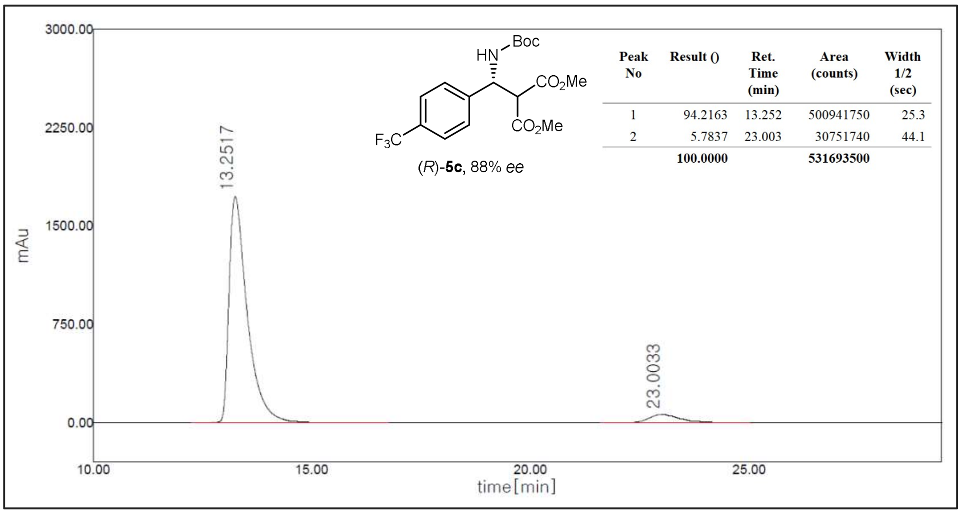

Peak No	Result ()	Ret. Time (min)	Area (counts)	Width 1/2 (sec)
1	94.2163	13.252	500941750	25.3
2	5.7837	23.003	30751740	44.1
	**100.0000**		**531693500**	

Supplementary Figure 200. HPLC spectra of **5c** (condition ***b***)

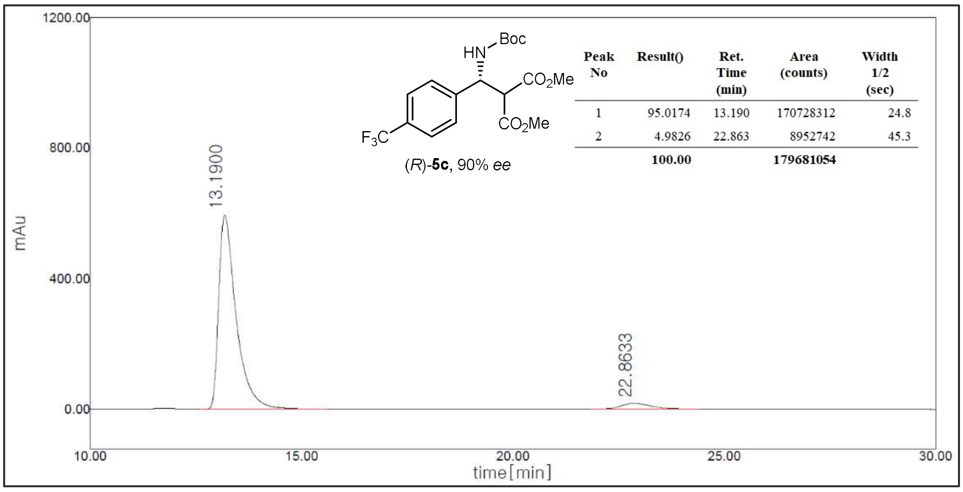

Peak No	Result()	Ret. Time (min)	Area (counts)	Width 1/2 (sec)
1	95.0174	13.190	170728312	24.8
2	4.9826	22.863	8952742	45.3
	**100.00**		**179681054**	

Supplementary Figure 201. HPLC spectra of **5c** (condition ***c-1***)

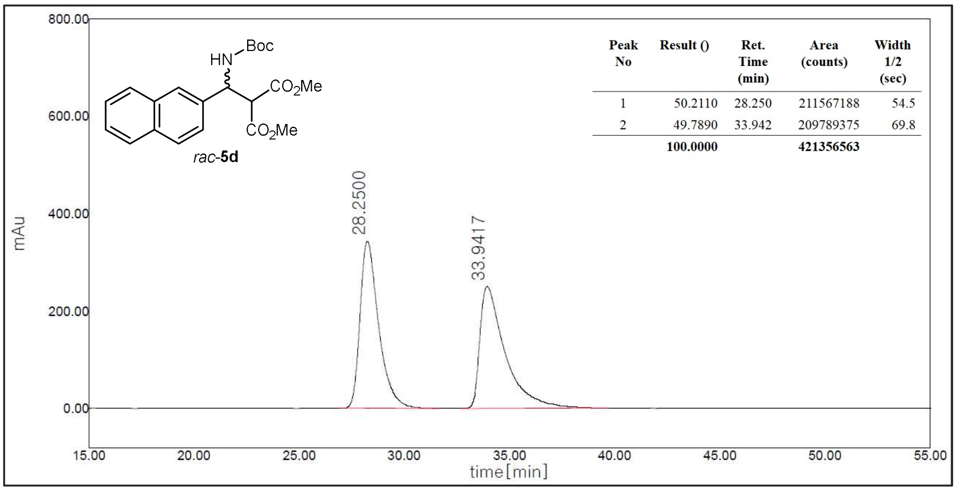

Peak No	Result ()	Ret. Time (min)	Area (counts)	Width 1/2 (sec)
1	50.2110	28.250	211567188	54.5
2	49.7890	33.942	209789375	69.8
	**100.0000**		**421356563**	

Supplementary Figure 202. HPLC spectra of *rac*-**5d**

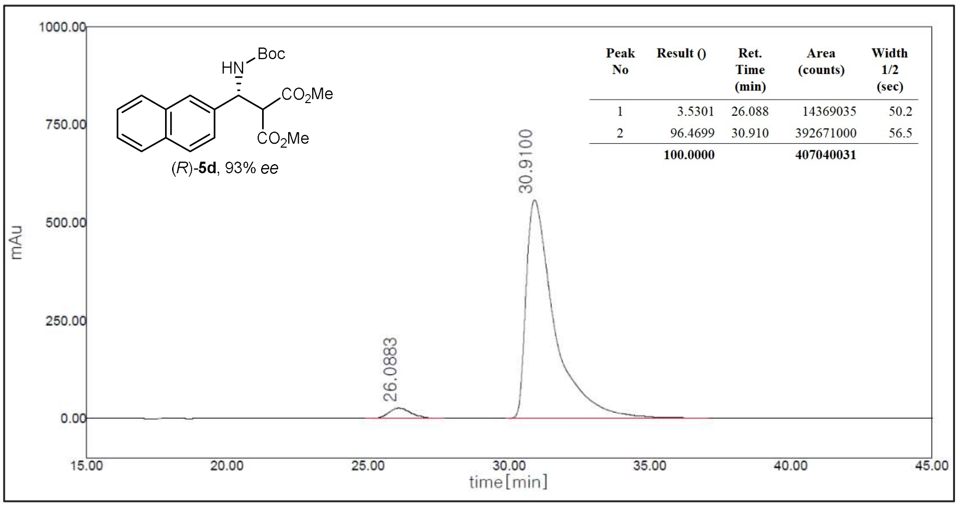

Peak No	Result ()	Ret. Time (min)	Area (counts)	Width 1/2 (sec)
1	3.5301	26.088	14369035	50.2
2	96.4699	30.910	392671000	56.5
	**100.0000**		**407040031**	

Supplementary Figure 203. HPLC spectra of **5d** (condition ***b***)

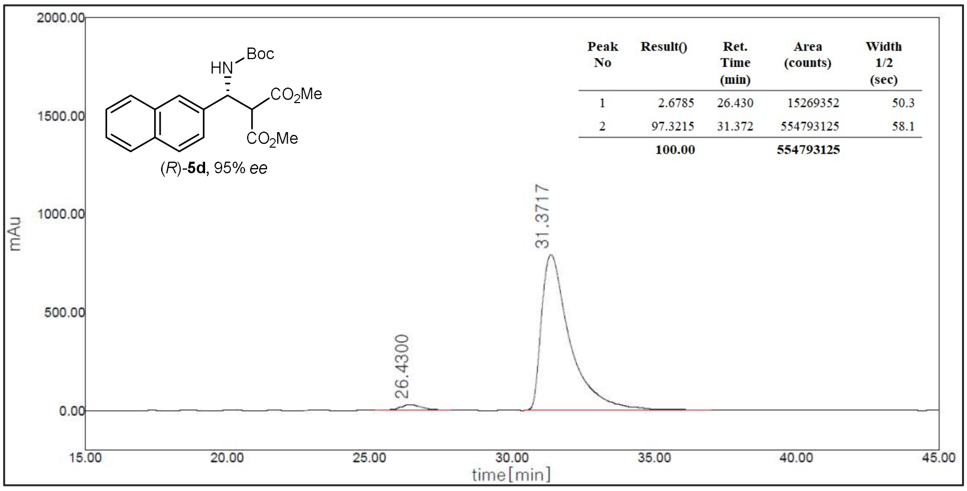

Peak No	Result()	Ret. Time (min)	Area (counts)	Width 1/2 (sec)
1	2.6785	26.430	15269352	50.3
2	97.3215	31.372	554793125	58.1
	**100.00**		**554793125**	

Supplementary Figure 204. HPLC spectra of **5d** (condition ***c-1***)

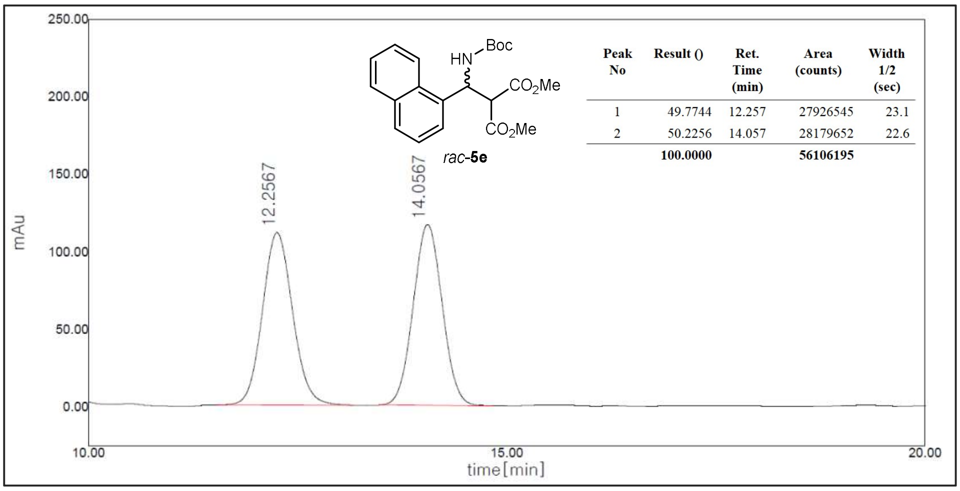

Peak No	Result ()	Ret. Time (min)	Area (counts)	Width 1/2 (sec)
1	49.7744	12.257	27926545	23.1
2	50.2256	14.057	28179652	22.6
	**100.0000**		**56106195**	

Supplementary Figure 205. HPLC spectra of *rac*-**5e**

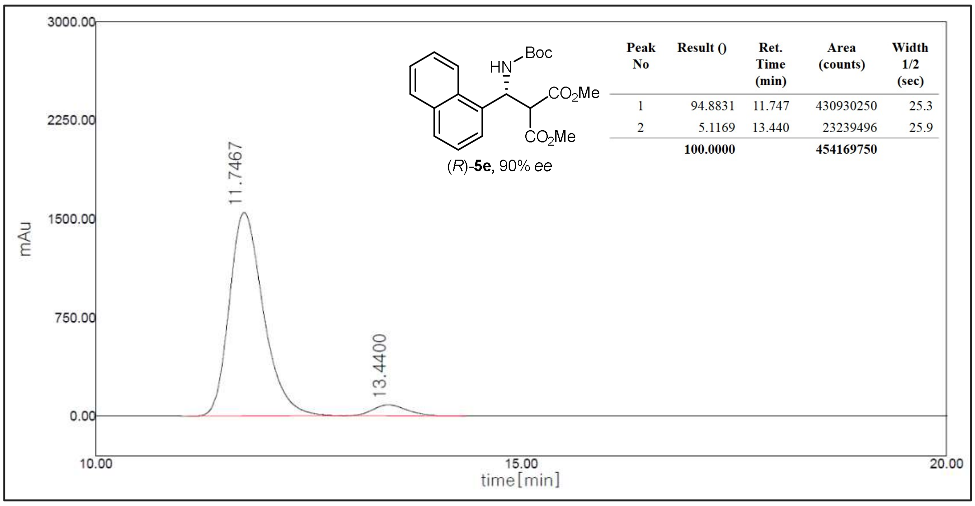

Peak No	Result ()	Ret. Time (min)	Area (counts)	Width 1/2 (sec)
1	94.8831	11.747	430930250	25.3
2	5.1169	13.440	23239496	25.9
	**100.0000**		**454169750**	

Supplementary Figure 206. HPLC spectra of **5e** (condition ***b***)

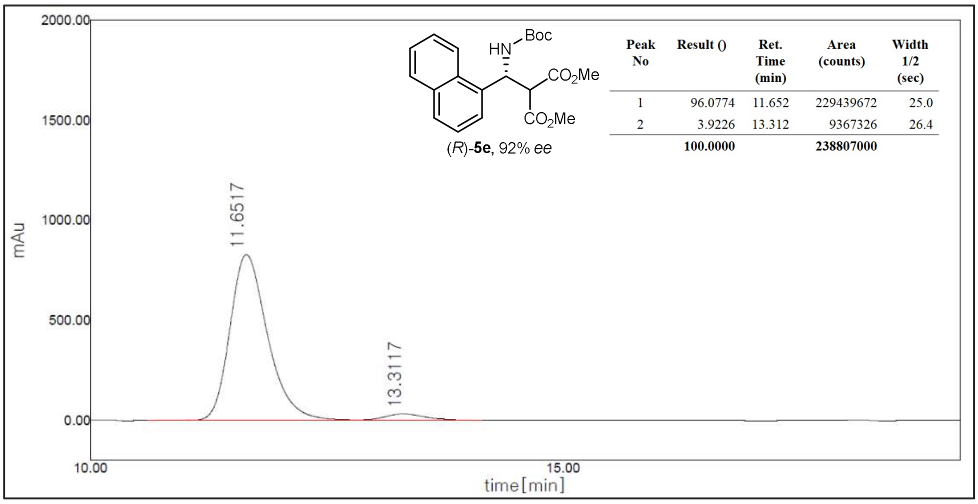

Peak No	Result ()	Ret. Time (min)	Area (counts)	Width 1/2 (sec)
1	96.0774	11.652	229439672	25.0
2	3.9226	13.312	9367326	26.4
	**100.0000**		**238807000**	

Supplementary Figure 207. HPLC spectra of **5e** (condition ***c-1***)

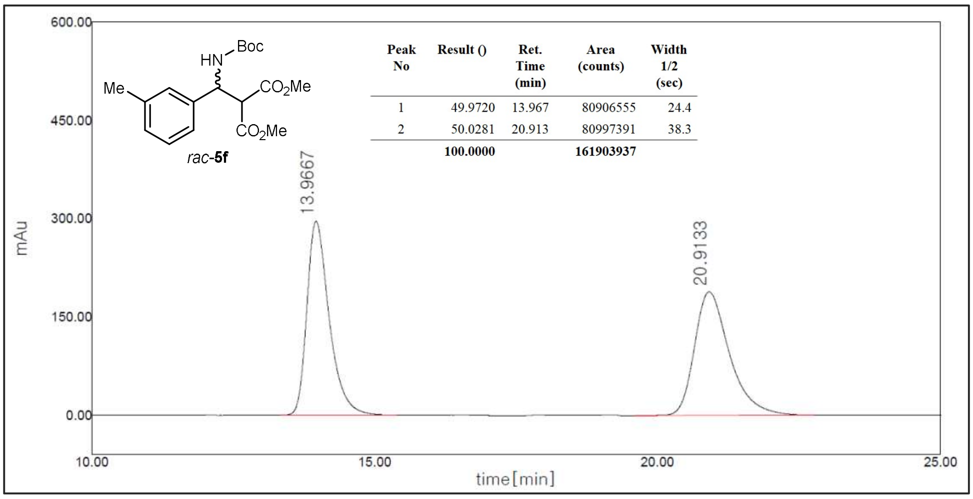

Peak No	Result ()	Ret. Time (min)	Area (counts)	Width 1/2 (sec)
1	49.9720	13.967	80906555	24.4
2	50.0281	20.913	80997391	38.3
	**100.0000**		**161903937**	

Supplementary Figure 208. HPLC spectra of *rac*-**5f**

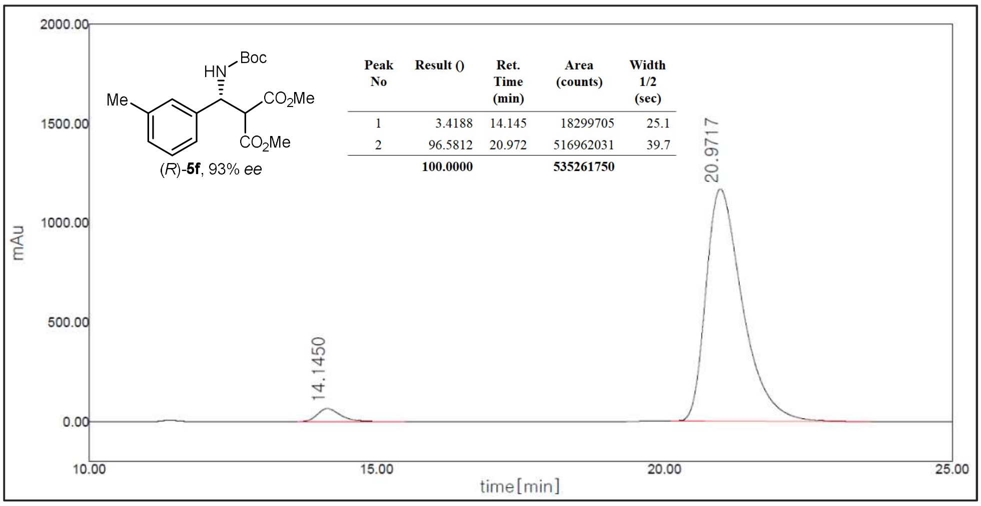

Peak No	Result ()	Ret. Time (min)	Area (counts)	Width 1/2 (sec)
1	3.4188	14.145	18299705	25.1
2	96.5812	20.972	516962031	39.7
	**100.0000**		**535261750**	

Supplementary Figure 209. HPLC spectra of **5f** (condition ***b***)

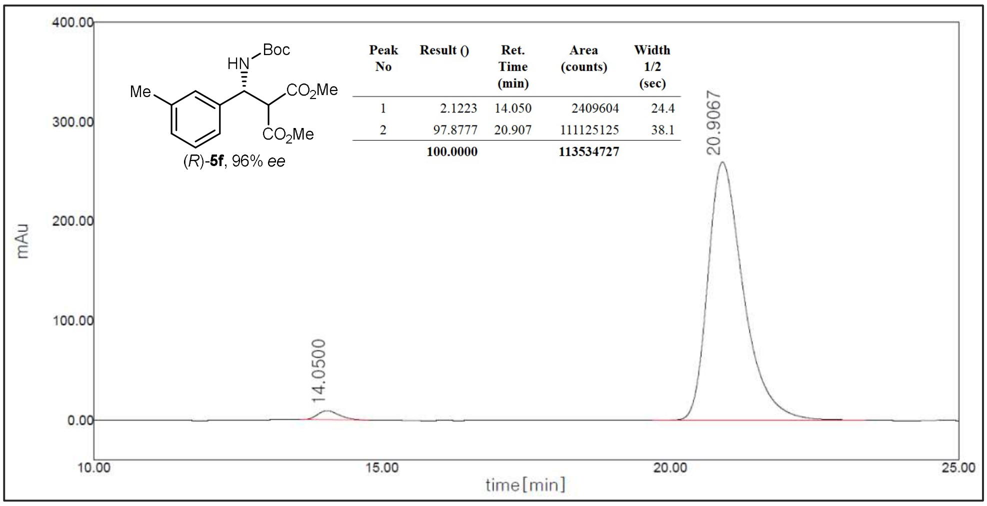

Peak No	Result ()	Ret. Time (min)	Area (counts)	Width 1/2 (sec)
1	2.1223	14.050	2409604	24.4
2	97.8777	20.907	111125125	38.1
	**100.0000**		**113534727**	

Supplementary Figure 210. HPLC spectra of **5f** (condition ***c-1***)

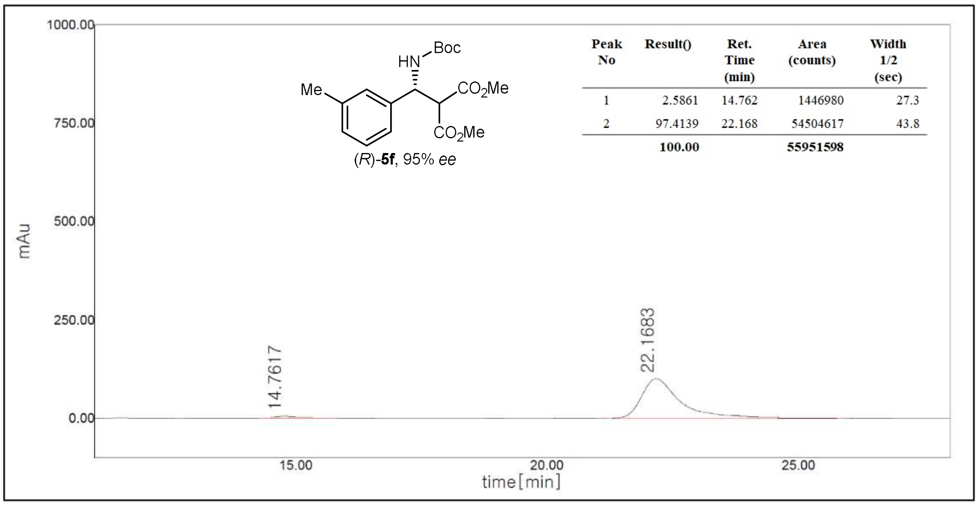

Peak No	Result()	Ret. Time (min)	Area (counts)	Width 1/2 (sec)
1	2.5861	14.762	1446980	27.3
2	97.4139	22.168	54504617	43.8
	**100.00**		**55951598**	

Supplementary Figure 211. HPLC spectra of **5f** (condition ***c-2***)

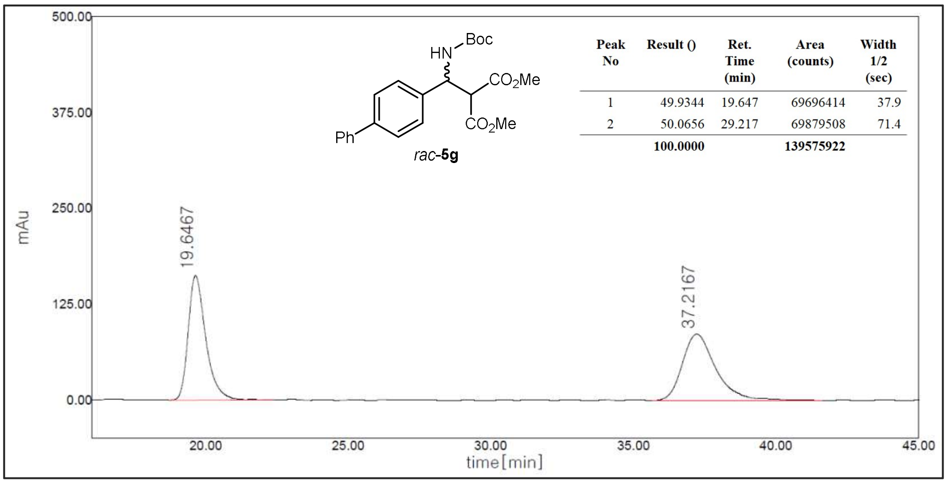

Peak No	Result ()	Ret. Time (min)	Area (counts)	Width 1/2 (sec)
1	49.9344	19.647	69696414	37.9
2	50.0656	29.217	69879508	71.4
	**100.0000**		**139575922**	

Supplementary Figure 212. HPLC spectra of *rac*-**5g**

condition ***b***

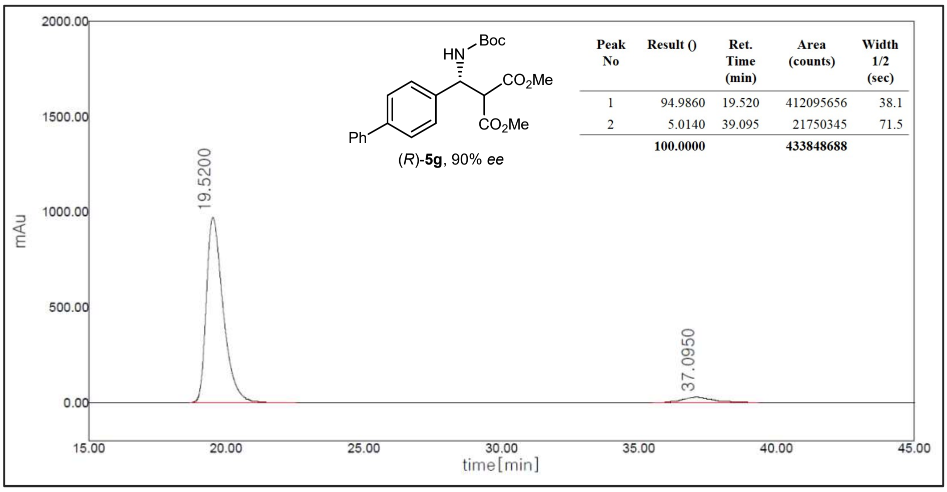

Peak No	Result ()	Ret. Time (min)	Area (counts)	Width 1/2 (sec)
1	94.9860	19.520	412095656	38.1
2	5.0140	39.095	21750345	71.5
	**100.0000**		**433848688**	

Supplementary Figure 213. HPLC spectra of **5g** (condition ***b***)

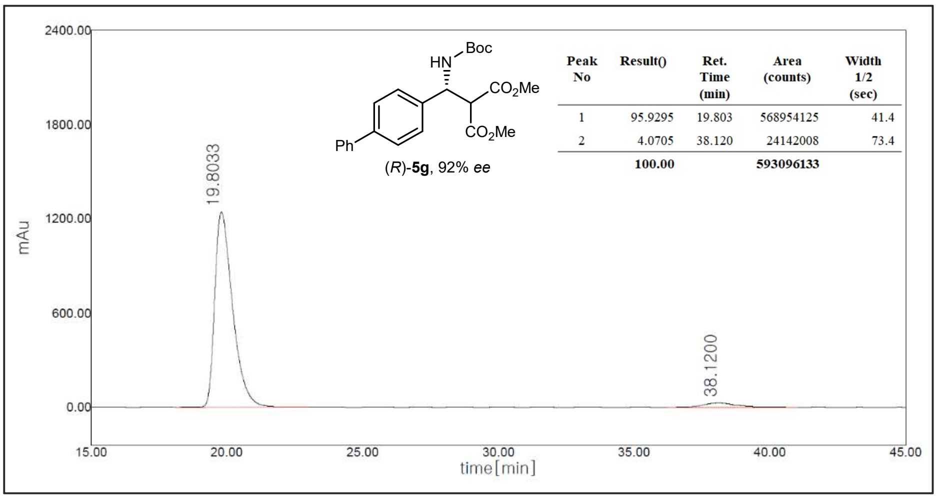

Peak No	Result()	Ret. Time (min)	Area (counts)	Width 1/2 (sec)
1	95.9295	19.803	568954125	41.4
2	4.0705	38.120	24142008	73.4
	**100.00**		**593096133**	

Supplementary Figure 214. HPLC spectra of **5g** (condition ***c-1***)

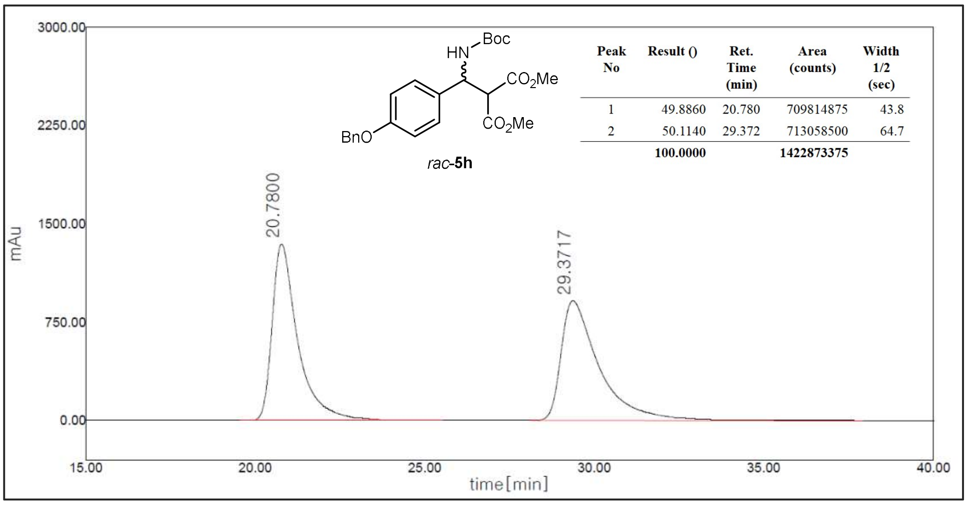

Supplementary Figure 215. HPLC spectra of *rac*-**5h**

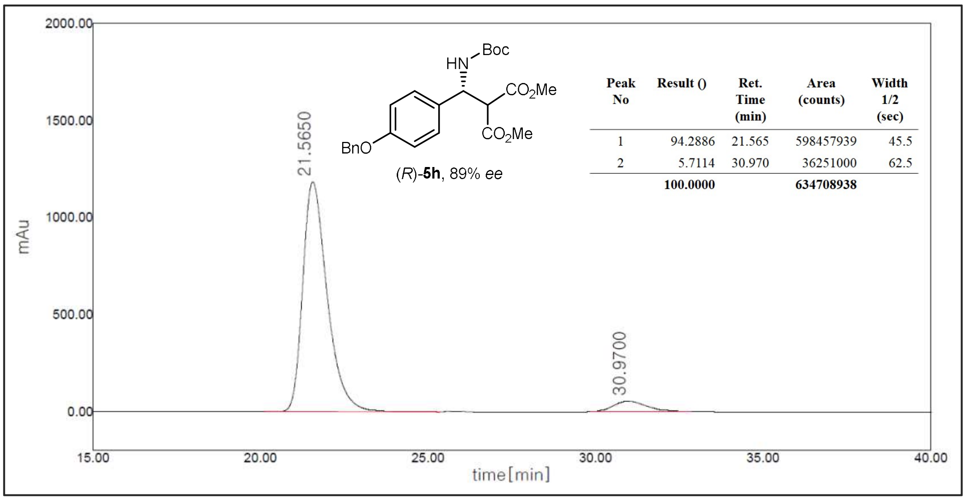

Supplementary Figure 216. HPLC spectra of **5h** (condition ***b***)

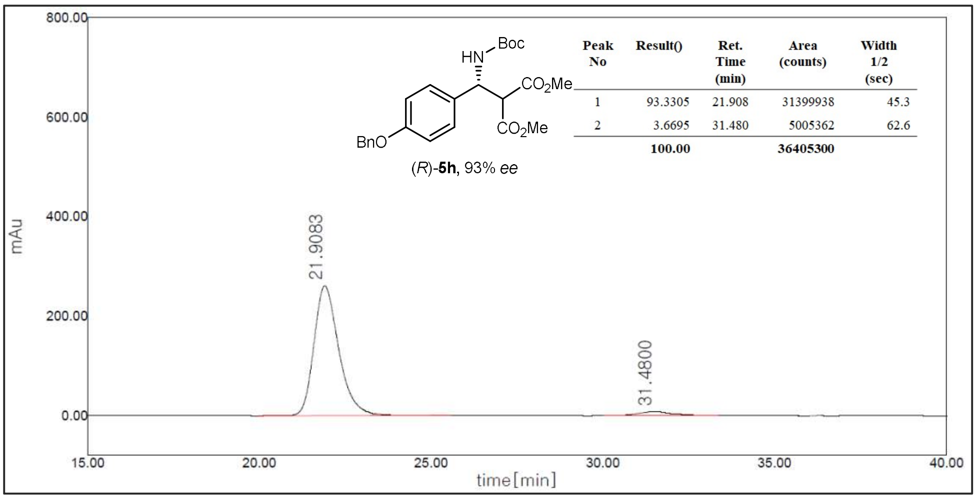

Supplementary Figure 217. HPLC spectra of **5h** (condition ***c-1***)

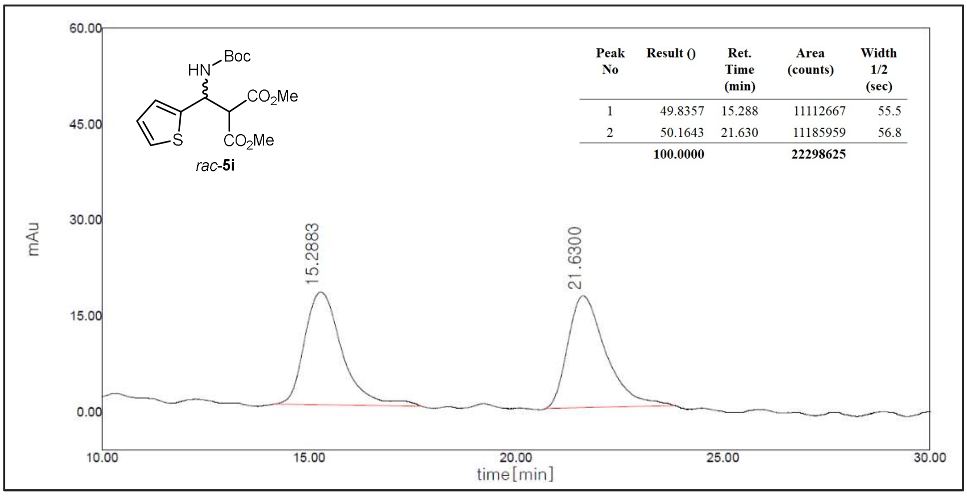

Peak No	Result ()	Ret. Time (min)	Area (counts)	Width 1/2 (sec)
1	49.8357	15.288	11112667	55.5
2	50.1643	21.630	11185959	56.8
	**100.0000**		**22298625**	

Supplementary Figure 218. HPLC spectra of *rac*-**5i**

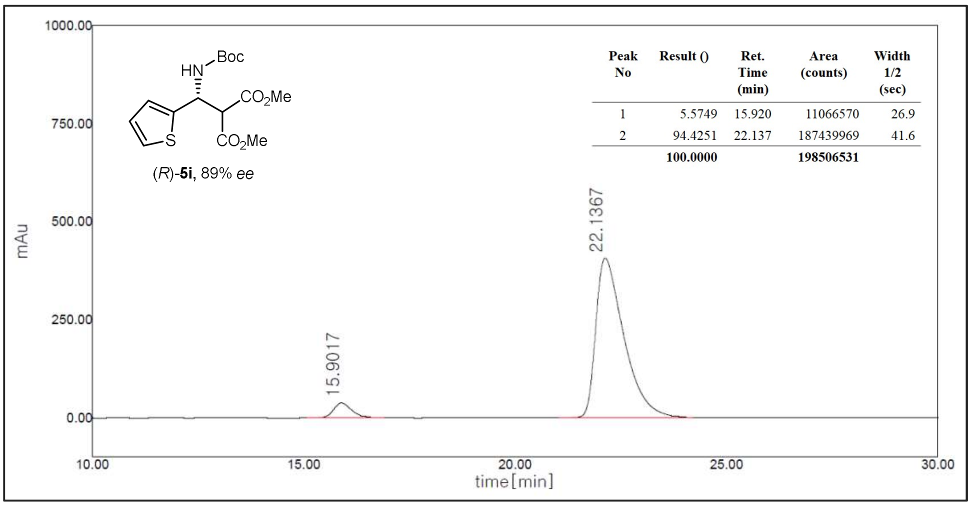

Peak No	Result ()	Ret. Time (min)	Area (counts)	Width 1/2 (sec)
1	5.5749	15.920	11066570	26.9
2	94.4251	22.137	187439969	41.6
	**100.0000**		**198506531**	

Supplementary Figure 219. HPLC spectra of **5i** (condition ***b***)

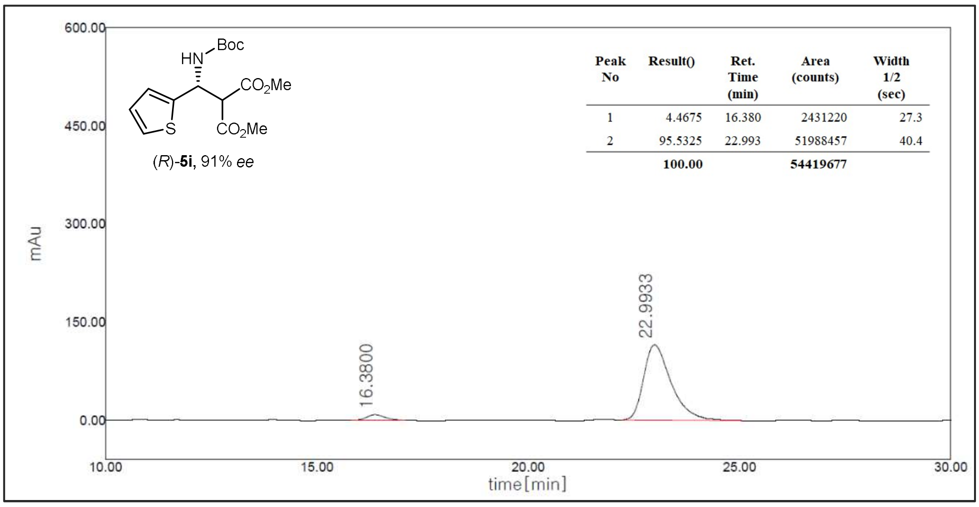

Peak No	Result()	Ret. Time (min)	Area (counts)	Width 1/2 (sec)
1	4.4675	16.380	2431220	27.3
2	95.5325	22.993	51988457	40.4
	**100.00**		**54419677**	

Supplementary Figure 220. HPLC spectra of **5i** (condition ***c-1***)

HPLC spectra of Fig. 5b

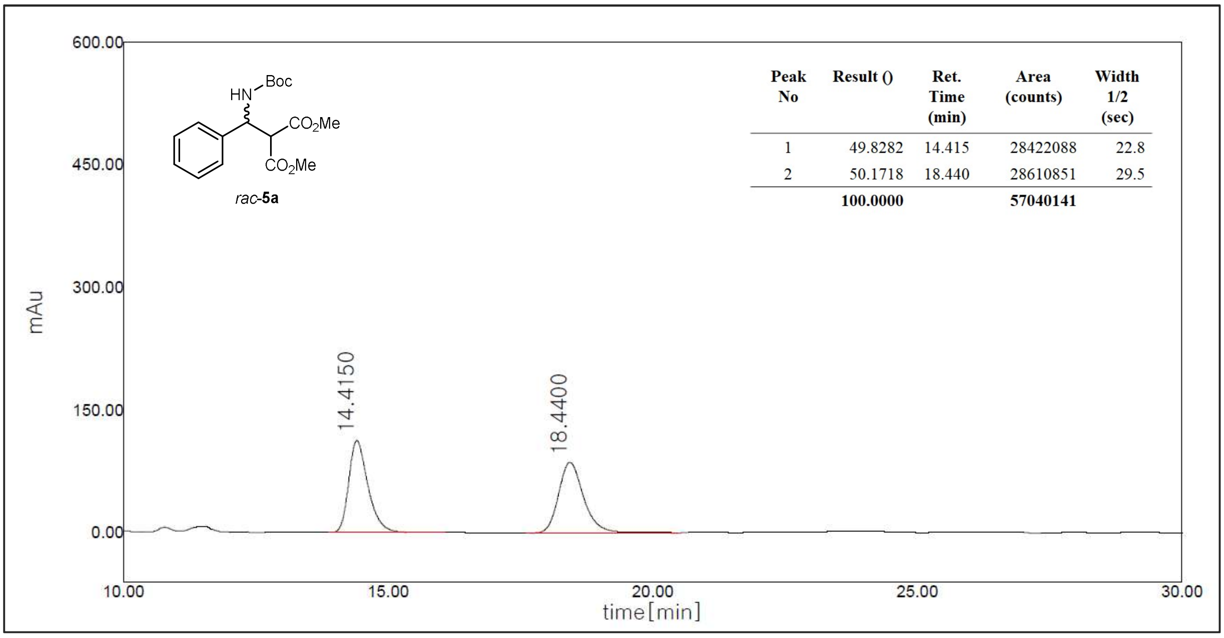

Peak No	Result ()	Ret. Time (min)	Area (counts)	Width 1/2 (sec)
1	49.8282	14.415	28422088	22.8
2	50.1718	18.440	28610851	29.5
	**100.0000**		**57040141**	

Supplementary Figure 221. HPLC spectra of *rac*-**5a**

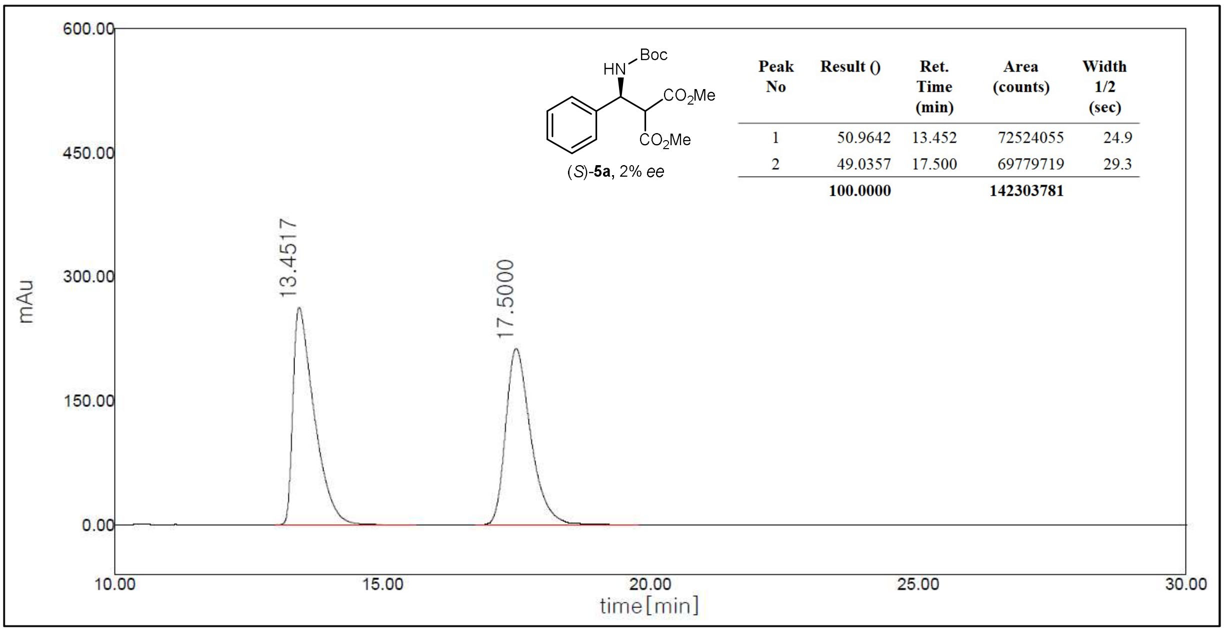

Peak No	Result ()	Ret. Time (min)	Area (counts)	Width 1/2 (sec)
1	50.9642	13.452	72524055	24.9
2	49.0357	17.500	69779719	29.3
	**100.0000**		**142303781**	

Supplementary Figure 222. HPLC spectra of **5a** (condition ***a***)

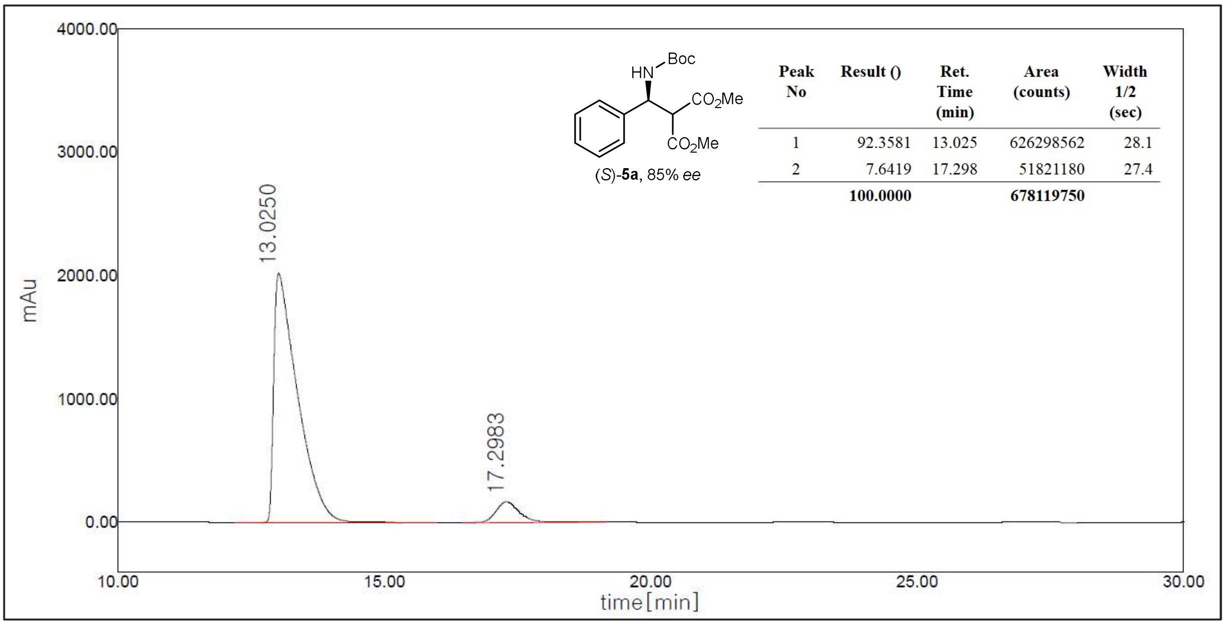

Peak No	Result ()	Ret. Time (min)	Area (counts)	Width 1/2 (sec)
1	92.3581	13.025	626298562	28.1
2	7.6419	17.298	51821180	27.4
	**100.0000**		**678119750**	

Supplementary Figure 223. HPLC spectra of **5a** (condition ***b***)

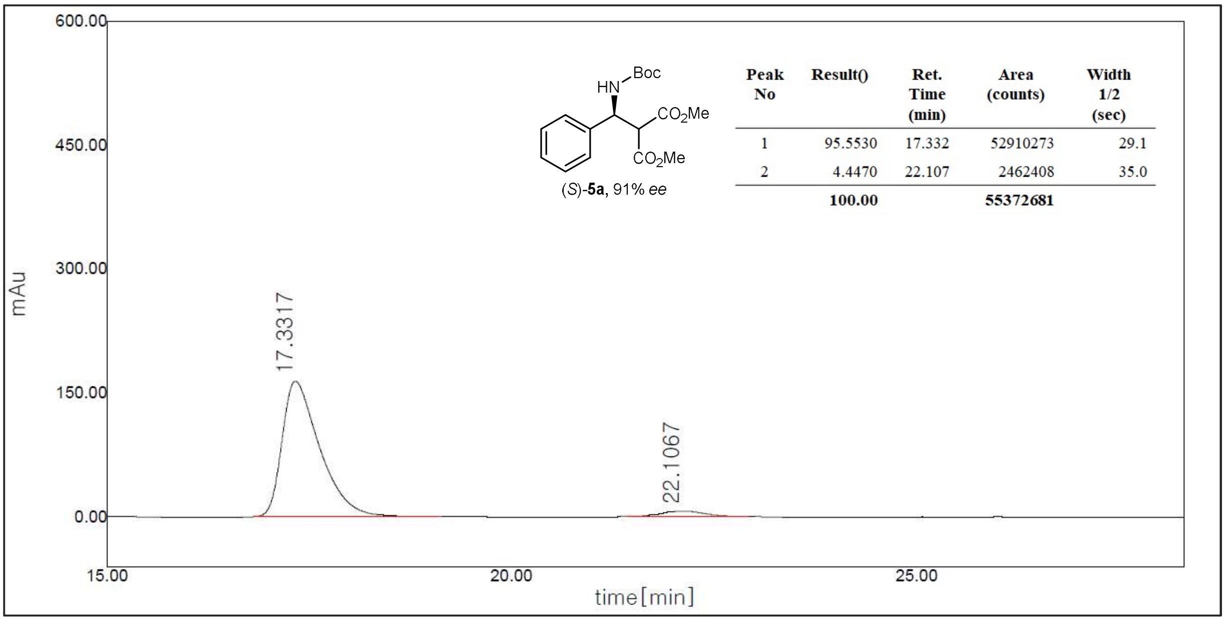

Peak No	Result()	Ret. Time (min)	Area (counts)	Width 1/2 (sec)
1	95.5530	17.332	52910273	29.1
2	4.4470	22.107	2462408	35.0
	**100.00**		**55372681**	

Supplementary Figure 224. HPLC spectra of **5a** (condition ***c-1***)

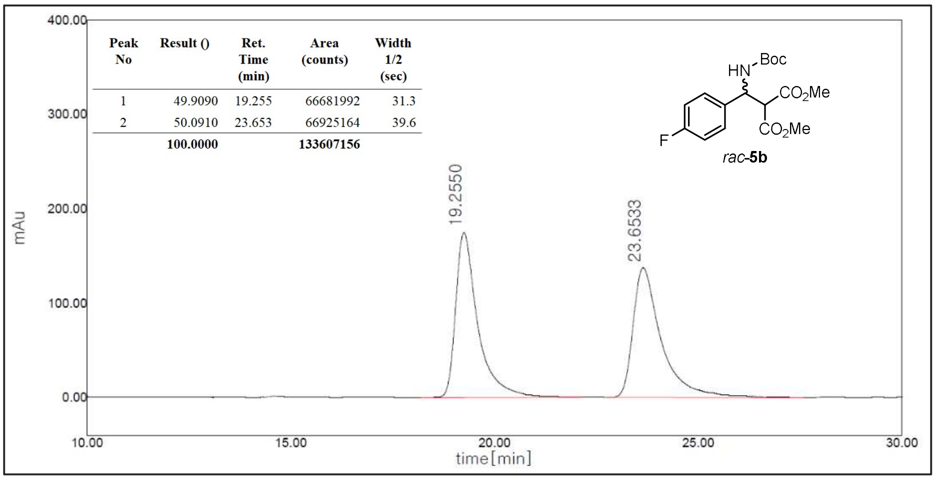

Peak No	Result ()	Ret. Time (min)	Area (counts)	Width 1/2 (sec)
1	49.9090	19.255	66681992	31.3
2	50.0910	23.653	66925164	39.6
	**100.0000**		**133607156**	

Supplementary Figure 225. HPLC spectra of *rac*-**5b**

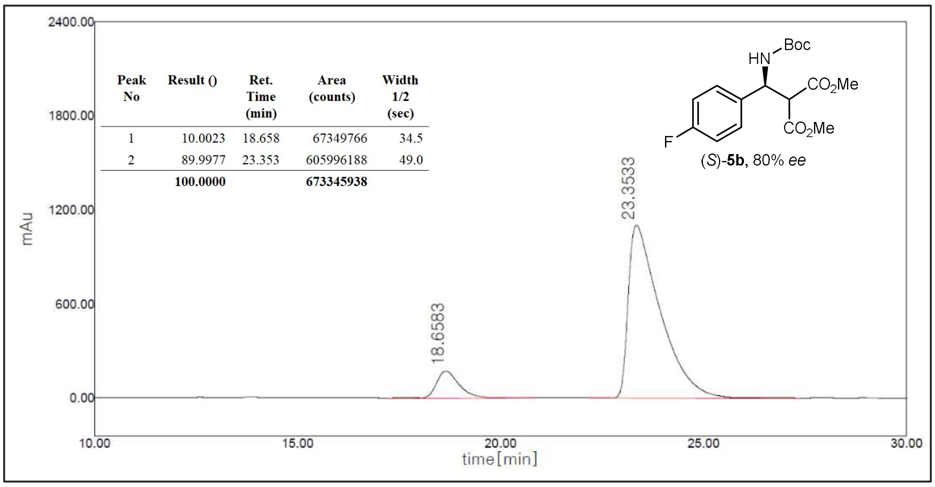

Peak No	Result ()	Ret. Time (min)	Area (counts)	Width 1/2 (sec)
1	10.0023	18.658	67349766	34.5
2	89.9977	23.353	605996188	49.0
	**100.0000**		**673345938**	

Supplementary Figure 226. HPLC spectra of **5b** (condition ***b***)

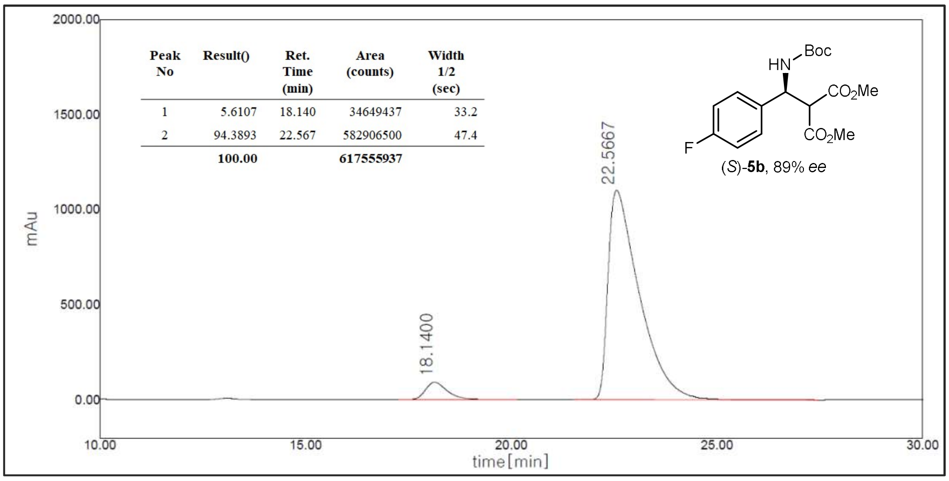

Peak No	Result()	Ret. Time (min)	Area (counts)	Width 1/2 (sec)
1	5.6107	18.140	34649437	33.2
2	94.3893	22.567	582906500	47.4
	**100.00**		**617555937**	

Supplementary Figure 227. HPLC spectra of **5b** (condition ***c-1***)

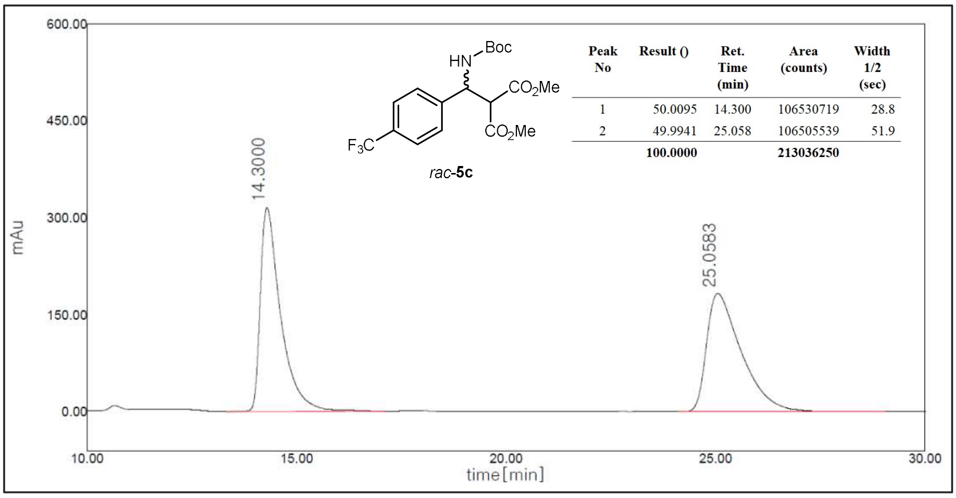

Peak No	Result ()	Ret. Time (min)	Area (counts)	Width 1/2 (sec)
1	50.0095	14.300	106530719	28.8
2	49.9941	25.058	106505539	51.9
	**100.0000**		**213036250**	

Supplementary Figure 228. HPLC spectra of *rac*-**5c**

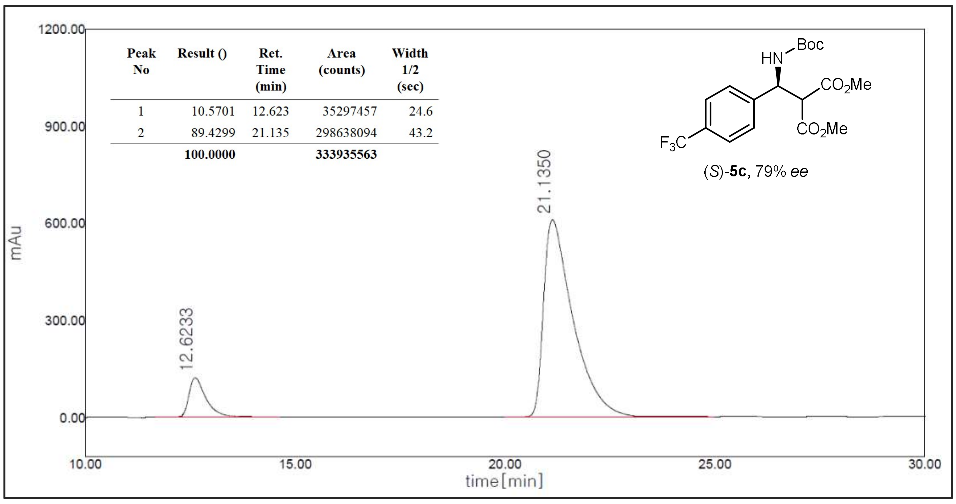

Peak No	Result ()	Ret. Time (min)	Area (counts)	Width 1/2 (sec)
1	10.5701	12.623	35297457	24.6
2	89.4299	21.135	298638094	43.2
	**100.0000**		**333935563**	

Supplementary Figure 229. HPLC spectra of **5c** (condition ***b***)

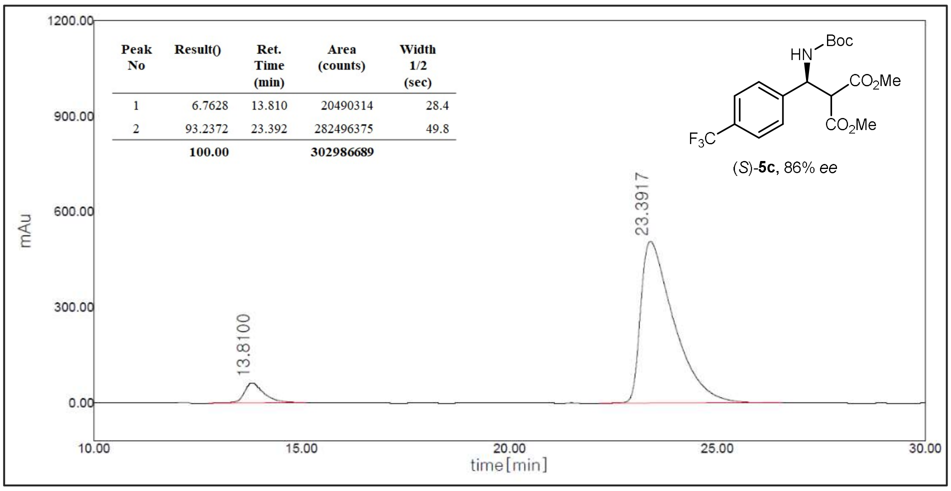

Peak No	Result()	Ret. Time (min)	Area (counts)	Width 1/2 (sec)
1	6.7628	13.810	20490314	28.4
2	93.2372	23.392	282496375	49.8
	**100.00**		**302986689**	

Supplementary Figure 230. HPLC spectra of **5c** (condition ***c-1***)

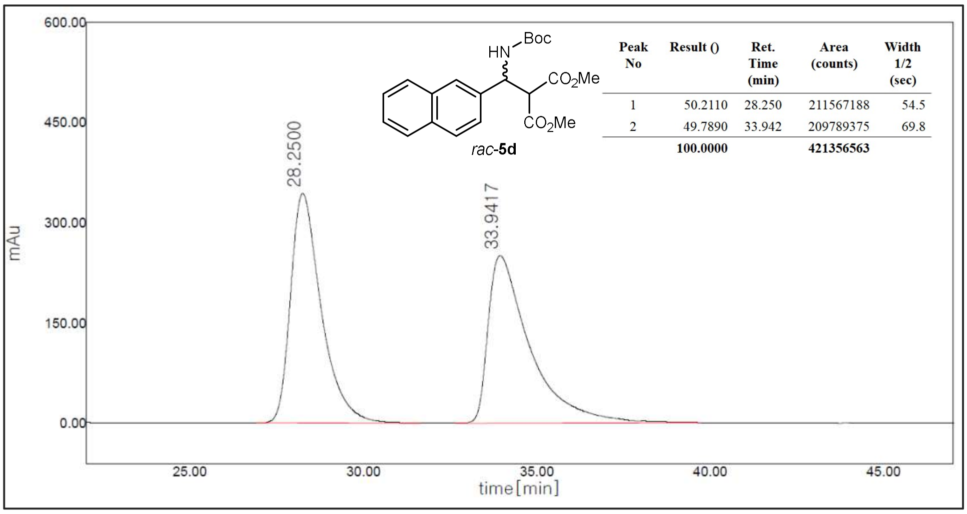

Peak No	Result ()	Ret. Time (min)	Area (counts)	Width 1/2 (sec)
1	50.2110	28.250	211567188	54.5
2	49.7890	33.942	209789375	69.8
	**100.0000**		**421356563**	

Supplementary Figure 231. HPLC spectra of *rac*-**5d**

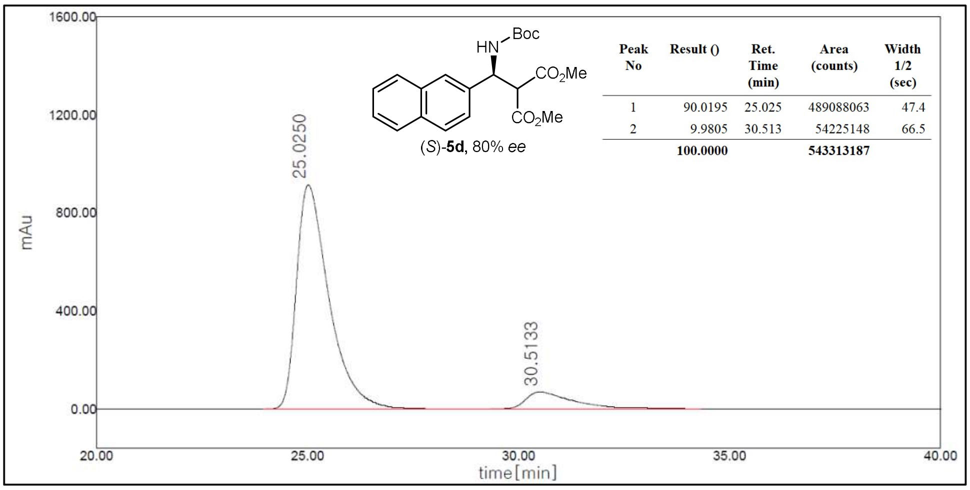

Peak No	Result ()	Ret. Time (min)	Area (counts)	Width 1/2 (sec)
1	90.0195	25.025	489088063	47.4
2	9.9805	30.513	54225148	66.5
	**100.0000**		**543313187**	

Supplementary Figure 232. HPLC spectra of **5d** (condition ***b***)

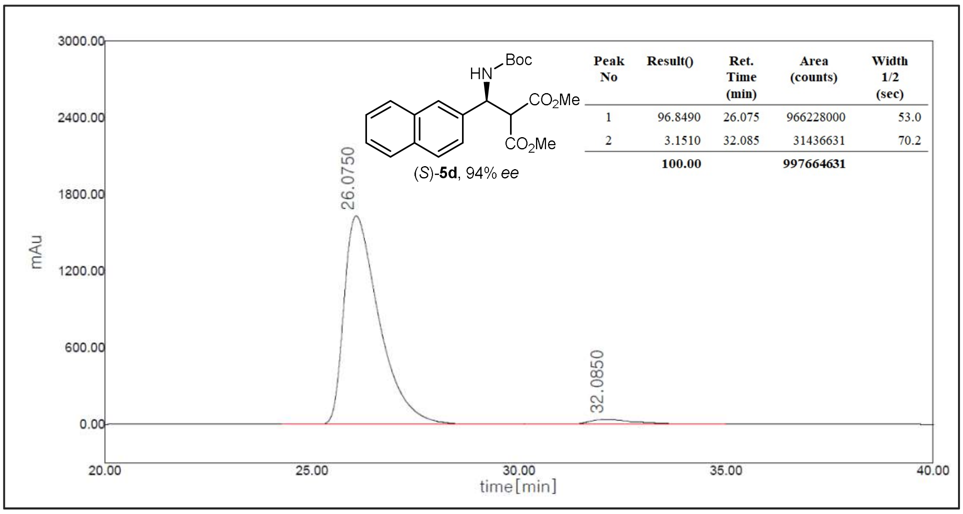

Peak No	Result()	Ret. Time (min)	Area (counts)	Width 1/2 (sec)
1	96.8490	26.075	966228000	53.0
2	3.1510	32.085	31436631	70.2
	**100.00**		**997664631**	

Supplementary Figure 233. HPLC spectra of **5d** (condition ***c-1***)

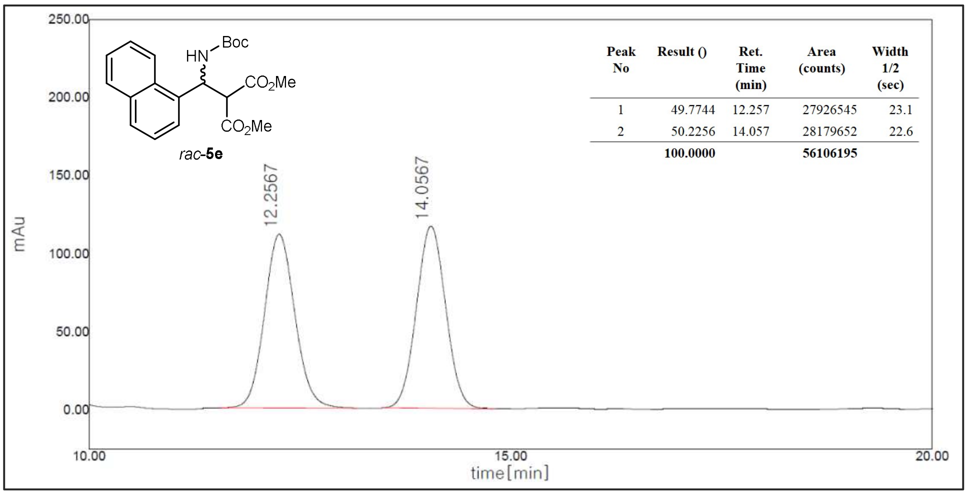

Peak No	Result ()	Ret. Time (min)	Area (counts)	Width 1/2 (sec)
1	49.7744	12.257	27926545	23.1
2	50.2256	14.057	28179652	22.6
	**100.0000**		**56106195**	

Supplementary Figure 234. HPLC spectra of *rac*-**5e**

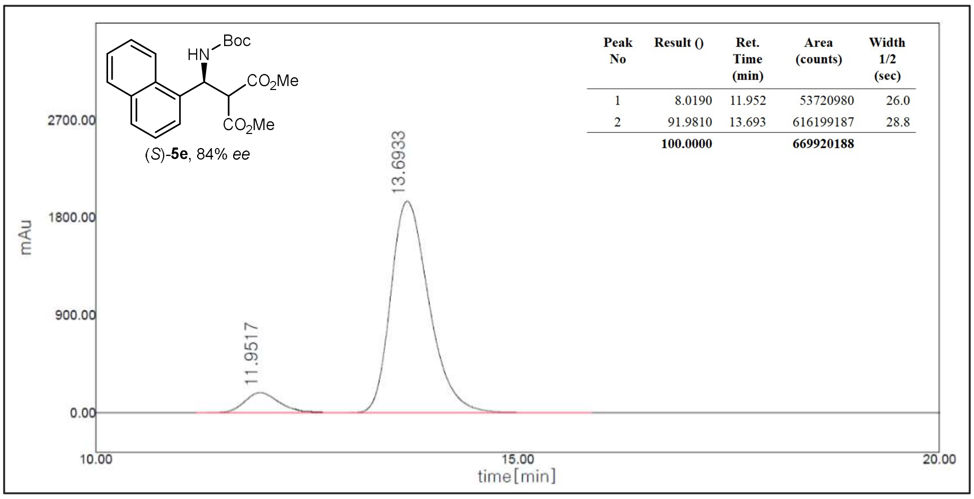

Peak No	Result ()	Ret. Time (min)	Area (counts)	Width 1/2 (sec)
1	8.0190	11.952	53720980	26.0
2	91.9810	13.693	616199187	28.8
	**100.0000**		**669920188**	

Supplementary Figure 235. HPLC spectra of **5e** (condition ***b***)

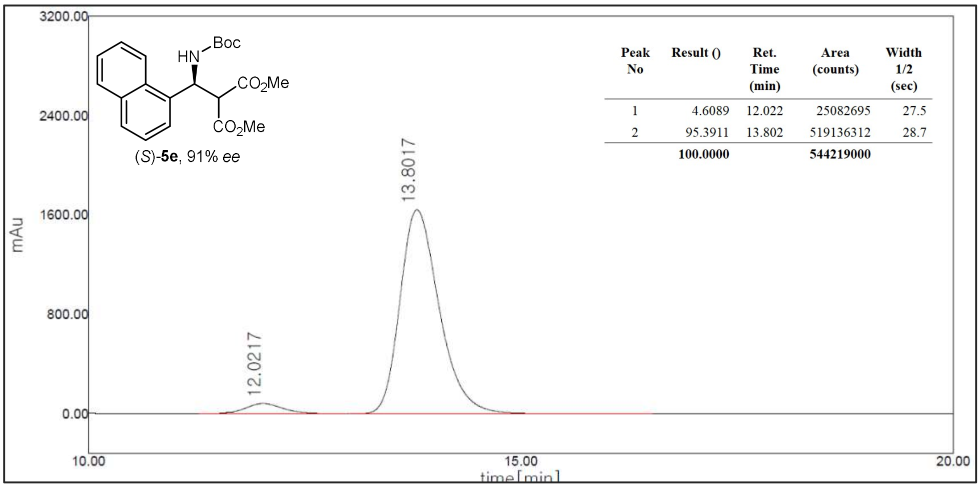

Peak No	Result ()	Ret. Time (min)	Area (counts)	Width 1/2 (sec)
1	4.6089	12.022	25082695	27.5
2	95.3911	13.802	519136312	28.7
	**100.0000**		**544219000**	

Supplementary Figure 236. HPLC spectra of **5e** (condition ***c-1***)

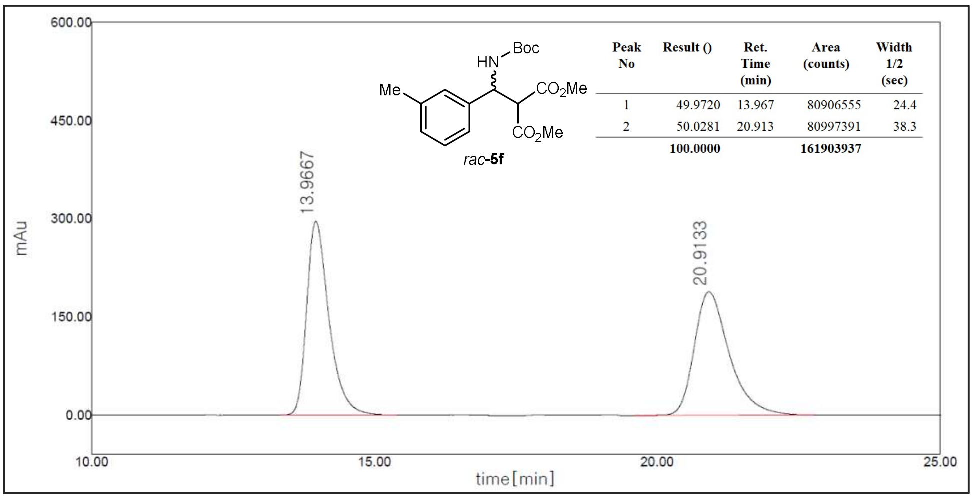

Peak No	Result ()	Ret. Time (min)	Area (counts)	Width 1/2 (sec)
1	49.9720	13.967	80906555	24.4
2	50.0281	20.913	80997391	38.3
	**100.0000**		**161903937**	

Supplementary Figure 237. HPLC spectra of *rac*-**5f**

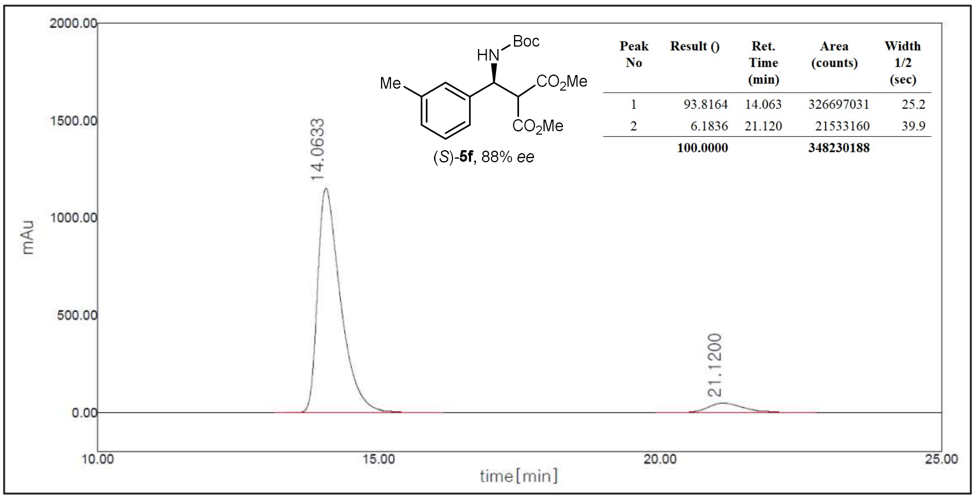

Peak No	Result ()	Ret. Time (min)	Area (counts)	Width 1/2 (sec)
1	93.8164	14.063	326697031	25.2
2	6.1836	21.120	21533160	39.9
	**100.0000**		**348230188**	

Supplementary Figure 238. HPLC spectra of **5f** (condition ***b***)

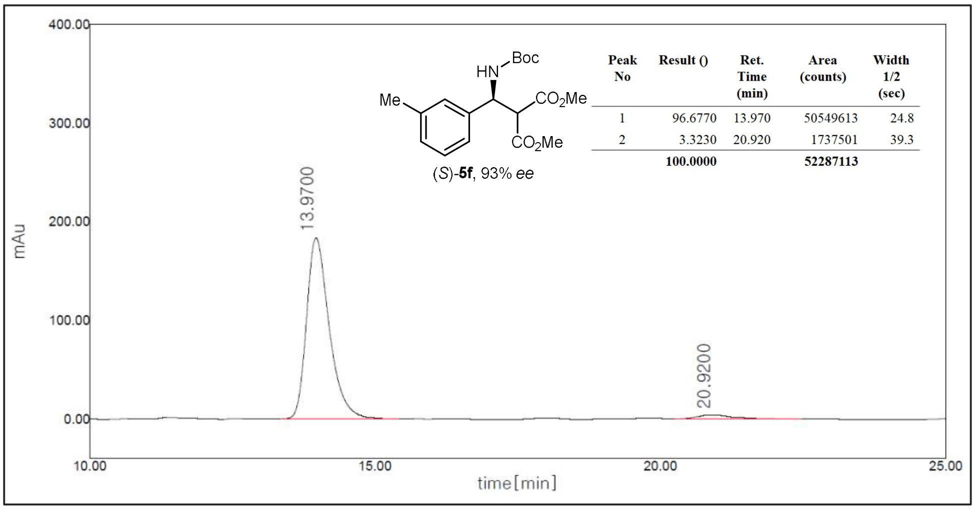

Peak No	Result ()	Ret. Time (min)	Area (counts)	Width 1/2 (sec)
1	96.6770	13.970	50549613	24.8
2	3.3230	20.920	1737501	39.3
	**100.0000**		**52287113**	

Supplementary Figure 239. HPLC spectra of **5f** (condition ***c-1***)

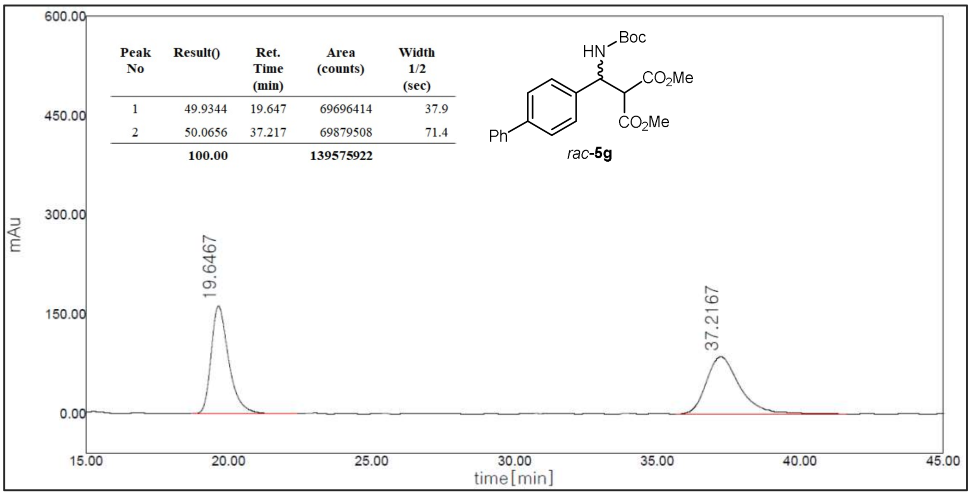

Peak No	Result()	Ret. Time (min)	Area (counts)	Width 1/2 (sec)
1	49.9344	19.647	69696414	37.9
2	50.0656	37.217	69879508	71.4
	**100.00**		**139575922**	

Supplementary Figure 240. HPLC spectra of *rac*-**5g**

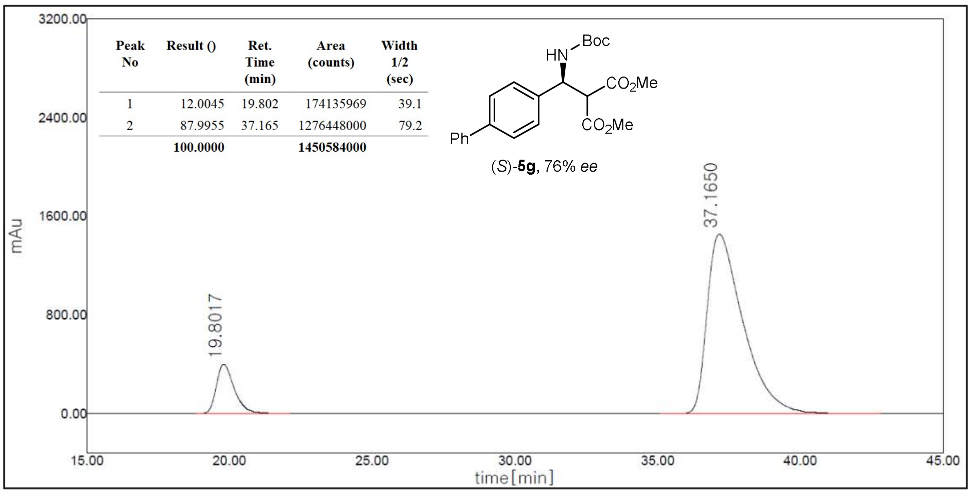

Peak No	Result ()	Ret. Time (min)	Area (counts)	Width 1/2 (sec)
1	12.0045	19.802	174135969	39.1
2	87.9955	37.165	1276448000	79.2
	**100.0000**		**1450584000**	

Supplementary Figure 241. HPLC spectra of *rac*-**5g**

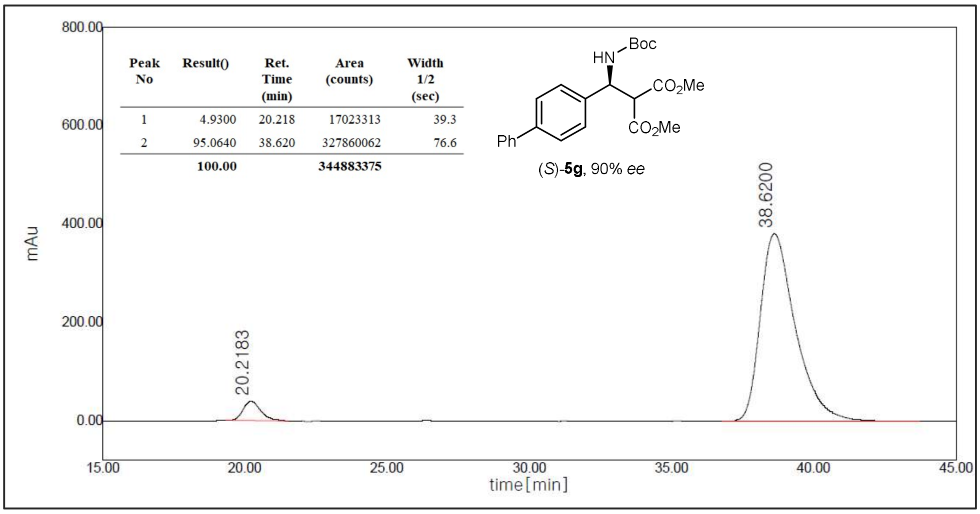

Peak No	Result()	Ret. Time (min)	Area (counts)	Width 1/2 (sec)
1	4.9300	20.218	17023313	39.3
2	95.0640	38.620	327860062	76.6
	**100.00**		**344883375**	

Supplementary Figure 242. HPLC spectra of **5g** (condition ***c-1***)

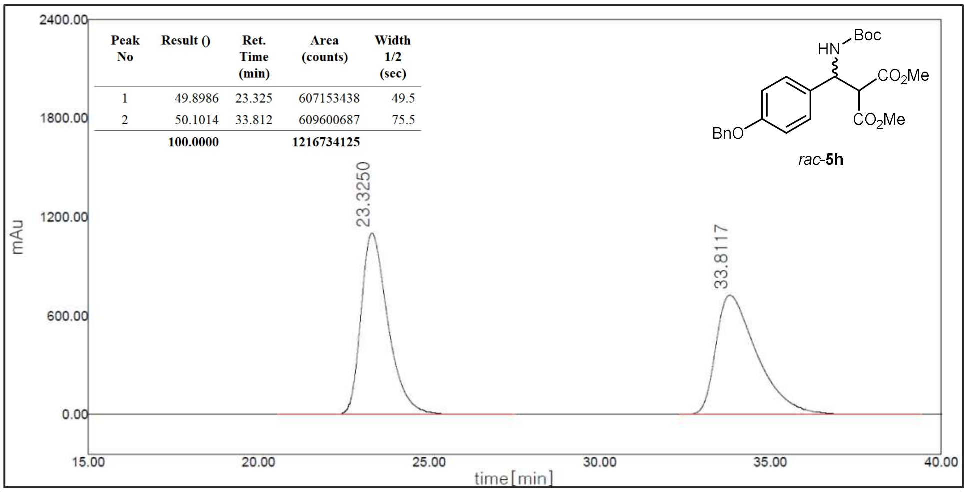

Supplementary Figure 243. HPLC spectra of *rac*-**5h**

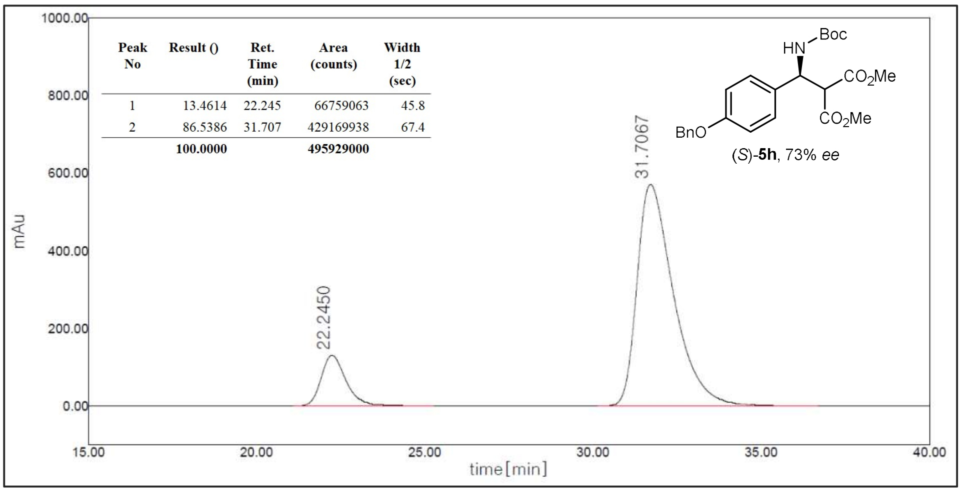

Supplementary Figure 244. HPLC spectra of **5h** (condition ***b***)

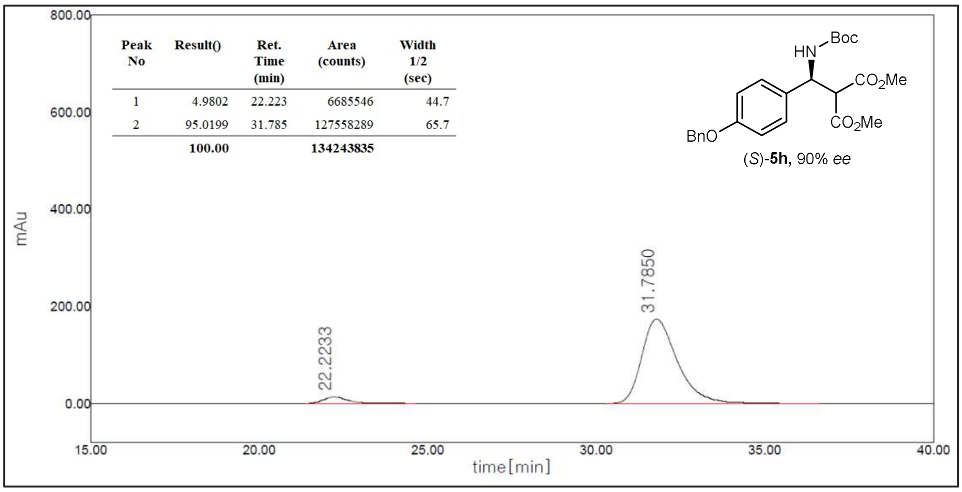

Supplementary Figure 245. HPLC spectra of **5h** (condition ***c-1***)

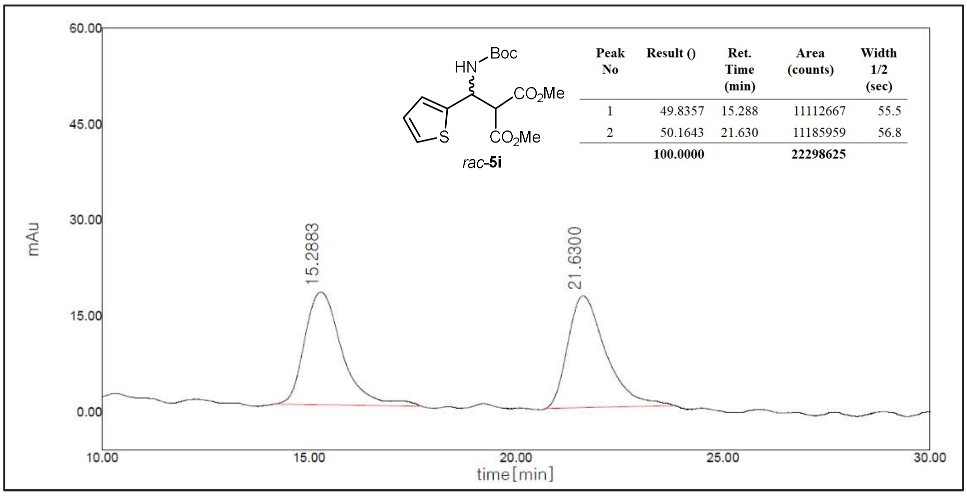

Peak No	Result ()	Ret. Time (min)	Area (counts)	Width 1/2 (sec)
1	49.8357	15.288	11112667	55.5
2	50.1643	21.630	11185959	56.8
	**100.0000**		**22298625**	

Supplementary Figure 246. HPLC spectra of *rac*-**5i**

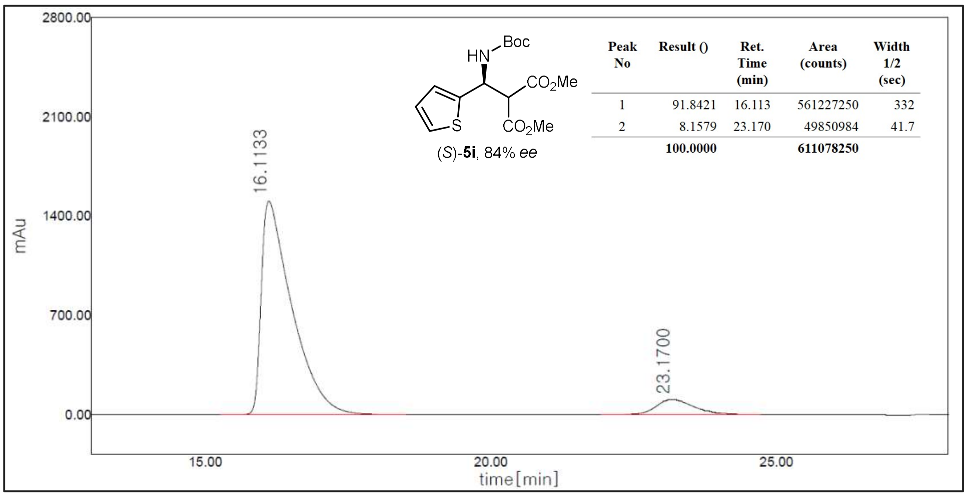

Peak No	Result ()	Ret. Time (min)	Area (counts)	Width 1/2 (sec)
1	91.8421	16.113	561227250	332
2	8.1579	23.170	49850984	41.7
	**100.0000**		**611078250**	

Supplementary Figure 247. HPLC spectra of **5i** (condition ***b***)

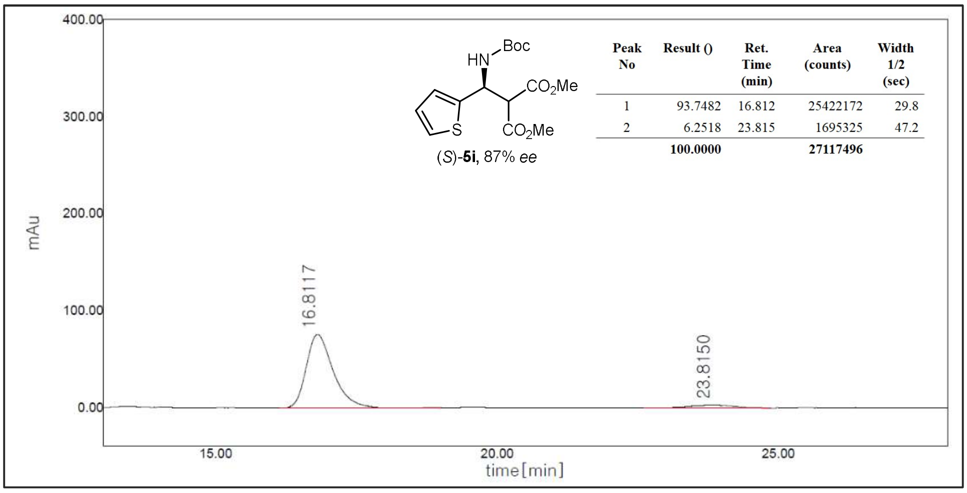

Peak No	Result ()	Ret. Time (min)	Area (counts)	Width 1/2 (sec)
1	93.7482	16.812	25422172	29.8
2	6.2518	23.815	1695325	47.2
	**100.0000**		**27117496**	

Supplementary Figure 248. HPLC spectra of **5i** (condition ***c-1***)

Supplementary References

1. Lou, S., Dai, P. & Schaus, S. E. Asymmetric Mannich reaction of dicarbonyl compounds with α-amido sulfones catalyzed by cinchona alkaloids and synthesis of chiral dihydropyrimidones. *J. Org. Chem.* ***72***, 9998−10008 (2007).
2. Uraguchi, D. & Terada, M. Chiral Brønsted acid-catalyzed direct Mannich reactions via electrophilic activation. *J. Am. Chem. Soc.* ***126***, 5356−5357 (2004).
3. Sohtome, Y., Tanaka¸ S., Takada, K., Yamaguchi, T. & Nagasawa, K. Solvent-dependent enantiodivergent Mannich-yype reaction: Utilizing a conformationally flexible guanidine/bisthiourea organocatalyst. *Angew. Chem. Int. Ed.* ***49***, 9254−9257 (2010).
4. Hatano, M., Horibe, T. & Ishihara, K. Magnesium(II)-binaphtholate as a practical chiral catalyst for the enantioselective direct Mannich-type reaction with malonates. *Org. Lett.* ***12***, 3502–3505 (2010).
5. Neuvonen, A. J., Földes, T., Madarász, Á., Pápai, I. & Pihko, P. M., Organocatalysts fold to generate an active site pocket for the Mannich reaction. *ACS Catal.* *7*, 3284−3294 (2017).
6. Hayashi, Y., Tsuboi, W., Shoji, M. & Suzuki, N. Application of high pressure induced by water-freezing to the direct catalytic asymmetric three-component List−Barbas−Mannich reaction. *J. Am. Chem. Soc.* **125**, 11208−11209 (2003).
